# Access to *P*-chiral *sec*- and *tert*-phosphine oxides enabled by Le-Phos-catalyzed asymmetric kinetic resolution†

**DOI:** 10.1039/d0sc04041j

**Published:** 2020-09-02

**Authors:** Haile Qiu, Qiang Dai, Jiafeng He, Wenbo Li, Junliang Zhang

**Affiliations:** Shanghai Key Laboratory of Green Chemistry and Chemical Processes, East China Normal University Shanghai P. R. China jlzhang@chem.ecnu.edu.cn; Department of Chemistry, Fudan University 2005 Songhu Road Shanghai 200438 P. R. China junliangzhang@fudan.edu.cn

## Abstract

The synthesis of *P*-stereogenic building blocks is extremely difficult. Herein we report an efficient kinetic resolution of secondary phosphine oxides *via* a Le-Phos-catalyzed asymmetric allylation reaction with Morita–Baylis–Hillman carbonates. This method provides facile access to enantioenriched secondary and tertiary *P*-chiral phosphine oxides with broad substrate scope, both of which could serve as *P*-stereogenic synthons, and can be rapidly incorporated into a given scaffold bearing a *P*-stereocenter. The highly desirable late stage modifications demonstrate the practicability of our method and can be a critical contribution to obtaining optimal *P*-chiral catalysts and ligands.

## Abstract 2

Herein we report an efficient kinetic resolution of secondary phosphine oxides *via* a Le-Phos-catalyzed asymmetric allylation reaction with Morita–Baylis–Hillman carbonates.

## Graphical abstract

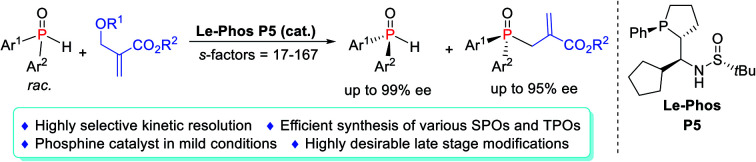

## Introduction

**Scheme 1:**
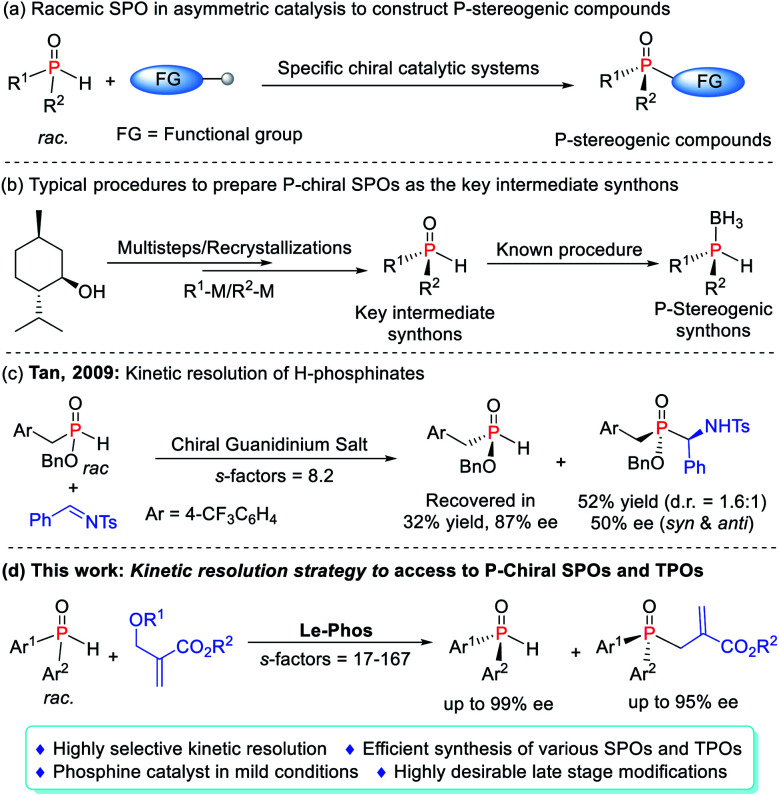
Application of the SPOs. (a) Catalytic asymmetric synthesis of P-chiral TPOs. (b) Construction and application of P-chiral SPOs. (c) Kinetic resolution of H-phosphinates. (d) This work: access to P-Chiral SPOs and TPOs.

*P*-Stereogenic phosphines represent a class of highly efficient ligands or catalysts as their stereocenters and active centers converge at one point and have been used in countless catalytic processes.^1^ However, the less availability and synthetic challenges are the hurdles for exploration of these privileged compounds and thus restrict their further applications.^2^ In this context, during the past two decades, catalytic asymmetric synthesis of *P*-stereogenic phosphines, as a more efficient alternative, has been an intensive field of research.^3–5^ The direct cross-coupling of secondary phosphines^3^ or secondary phosphine oxides (SPOs)^4^ with a variety of specific partners provided a more straightforward approach to access *P*-chiral compounds with diverse functional groups (Scheme 1a). The toxicity and liability of the former, however, largely deterred them from being used in many laboratories.^6^ As a promising alternative, SPOs were bench stable, less toxic, and odorless. Apart from the widespread use of their racemates in asymmetric catalysis to construct *P*-stereogenic compounds, their chiral version also served as a key intermediate synthon that could be rapidly incorporated into a given ligand scaffold (Scheme 1b).^7^

On the other hand, specific asymmetric catalytic systems usually achieved single transformation and were hardly used as general systems to construct multiple types of *P*-chiral compounds. Moreover, they often required an excess amount of SPOs to secure better enantioselectivities and yields due to the difficult interconversion of both enantiomers in the reaction system.^4*a*–*c*^ Although some studies have attempted to use this feature to achieve a standard kinetic resolution (KR) of SPOs in order to obtain *P*-chiral tertiary phosphine oxides (TPOs) as well as enantioenriched SPOs, the elusive degree of racemization of SPOs made the process very challenging.^4*b*–*d*^ In 2009, the Tan group achieved the kinetic separation of H-phosphinates in the phospha-Mannich reaction, obtaining enantiomerically pure H-phosphinates in 87% ee as only one example (Scheme 1c).^8^ To date, *P*-chiral SPOs were typically prepared using enantiopure starting materials, and chiral auxiliaries, or by recrystallization from the racemates.^9^ Very little attention has been paid to the acquisition of *P*-chiral SPOs by kinetic resolution, which still remains in its infancy. Herein we report a highly efficient kinetic resolution of SPOs *via* a Le-Phos-catalyzed asymmetric allylation reaction with Morita–Baylis–Hillman (MBH) carbonates (Scheme 1d). The developed process holds some promise as a potentially straightforward route to enantioenriched *P*-chiral SPOs as well as TPOs, both of which could serve as *P*-stereogenic synthons, and would undoubtedly spur on more detailed studies of their chemistry and synthetic utility.

## Results and discussion

Our initial investigation was carried out by using Boc-protected MBH adduct **2a** (0.5 equiv.) and racemic mesityl(phenyl)phosphine oxide **1a** in the presence of multifunctional chiral phosphine catalyst **P1** (Wei-Phos, 10 mol%) in toluene (Table 1, entry 1).^10^ The reaction gave the desired allylation product **3aa** in 34% yield with 7% ee, along with recovered **1a** in 30% yield with 17% ee, correlating with an *s*-factor of 1.3. Then, Xiao-Phos **P2** was tested but a little better result was obtained (Table 1, entry 2).^11^ Gratifyingly, the use of (*R*_P_,*S*,*S*,*R*_S_)-Le-Phos **P3** and (*S*_P_,*R*,*S*,*R*_S_)-Le-Phos **P4** as the catalysts, especially the latter one increased the ees of both **3aa** and recovered (*R*)-**1a** (Table 1, entries 3 and 4). This result indicated that the configuration of the catalyst backbone has a remarkable impact on the enantioselectivities of the products.

**Table 1:** Optimization of kinetic resolution of SPOs^a^

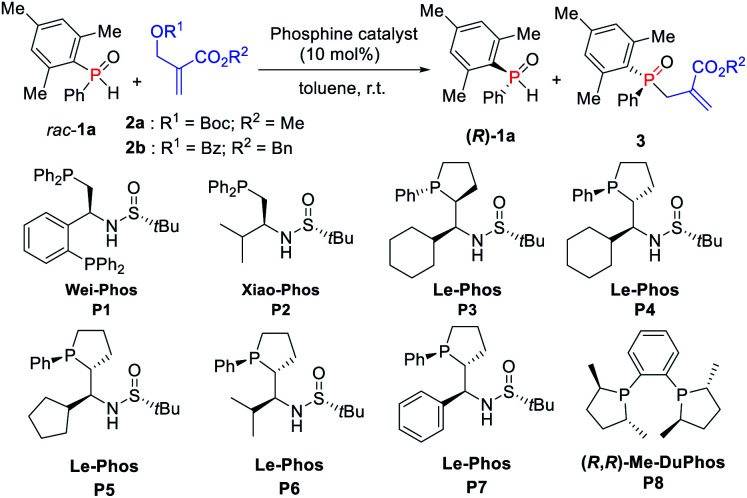				
Entry	Cat.	Recovery of **1a**	**3**	*s* factor
1	**P1**	30%, 17% ee	34%, 7% ee	1.3
2	**P2**	38%, −26% ee	36%, −21% ee	1.9
3	**P3**	42%, 10% ee	44%,10% ee	1.3
4	**P4**	30%, 86% ee	32%, 48% ee	7.4
5	**P5**	40%, 78% ee	42%, 63% ee	10.4
6	**P6**	41%, 64% ee	43%, 55% ee	6.5
7	**P7**	73%, −6% ee	18%, −35% ee	2.2
8^b^	**P5**	40%, 82% ee	42%, 81% ee	24.1
9^b^^,^^c^	**P5**	42%, 79% ee	40%, 79% ee	20.3
10^b^^,^^d^	**P5**	40%, 75% ee	40%, 78% ee	18.2
11^b^^,^^e^	**P5**	35%, 88% ee	38%, 78% ee	23.4
12^b^^,^^f^	**P5**	36%, 89% ee	44%, 75% ee	20.5
13^b^^,^^e^^,^^g^	**P5**	—	Trace	—
14^b^^,^^f^^,^^g^	**P5**	37%, 93% ee	44%, 86% ee	44.4
15^b^^,^^f^^,^^h^^,^^i^	**P5**	45%, 89% ee	43%, 90% ee	57
16^b^^,^^f^^,^^h^^,^^i^	**P8**	65%, 17% ee	20%, 66% ee	5.7

a All reactions were performed using **1a** (0.2 mmol), **2** (0.1 mmol) and an organocatalyst (10 mol%) in 1.5 mL of solvent at r.t. All yields were determined by ^1^H NMR analysis of the crude mixture and CH_2_Br_2_ as the internal standard. Enantiomeric excesses were determined by HPLC. *C* (calculated conversion) = ee_SM_/(ee_SM_ + ee_PR_). *s* (selectivity) = ln[(1 − *C*)(1 − ee_SM_)]/In[(1 − *C*) (1 + ee_SM_)].

b **2b** instead of **2a**.

c PhCO_2_H (50 mol%) used as an additive.

d PhOH (20 mol%) used as an additive.

e Mesitylene instead of toluene.

f *t*BuPh instead of toluene.

g At 10 °C.

h At 0 °C.

i *rac*-**1a** (0.205 mol) was used.

Encouraged by this finding, several modified chiral phosphine catalysts (*S*_P_,*R*,*S*,*R*_S_)-Le-Phos **P5–P7** with the variation of the substituent *R* at the center of the chiral sulfinamide were then tested.^12^ The enantioselectivity of **3aa** could be improved to 63% along with increased yields of **3aa** and recovered (*R*)-**1a** when the phosphine catalyst was switched to (*S*_P_,*R*,*S*,*R*_S_)-Le-Phos **P5** (*R* = cyclopentyl) (Table 1, entry 5). However, isopropyl- and phenyl-substituted **P6** and **P7** resulted in lower enantioselectivities (Table 1, entries 6 and 7). More importantly, when the benzoyl protected MBH adduct **2b** instead of **2a** was used under the catalysis of **P5** in toluene at room temperature, the product **3ab** was obtained in 42% yield with 81% ee and (*R*)-**1a** was recovered in 40% yield with 82% ee, further improving the *s*-factor to 24.1 (Table 1, entry 8). The additives such as PhCO_2_H and PhOH have no obvious effect on both yield and enantioselectivity (Table 1, entries 9 and 10). Further solvent screening revealed that the kinetic resolution of secondary phosphine oxide *rac*-**1a** afforded the allylation product **3ab** in better yields and recovered (*R*)-**1a** in higher ee by using *tert*-butylbenzene as solvent in a shorter reaction time (Table 1, entry 12). Lowering the reaction temperature to 10 °C improved the ees of **3ab** and (*R*)-**1a** to 86% and 93% for 8 h, respectively (Table 1, entry 14). It is noteworthy that only a trace amount of the corresponding product was detected with the use of mesitylene as the solvent at 10 °C (Table 1, entry 13). Fortunately, the enantioselectivity of the desired product **3ab** could be further improved to 90% by lowering the reaction temperature to 0 °C and increasing the equivalent of *rac*-**1a** to 2.05 (Table 1, entry 15). Lowering the temperature also largely improved the *s*-value to 57. We also tried out the classical chiral phosphine catalyst (*R*,*R*)-Me-DuPhos (**P8**), but unfortunately the ee value and *s*-value were lower compared with those of **P5** (Table 1, entry 16).

**Scheme 2:**
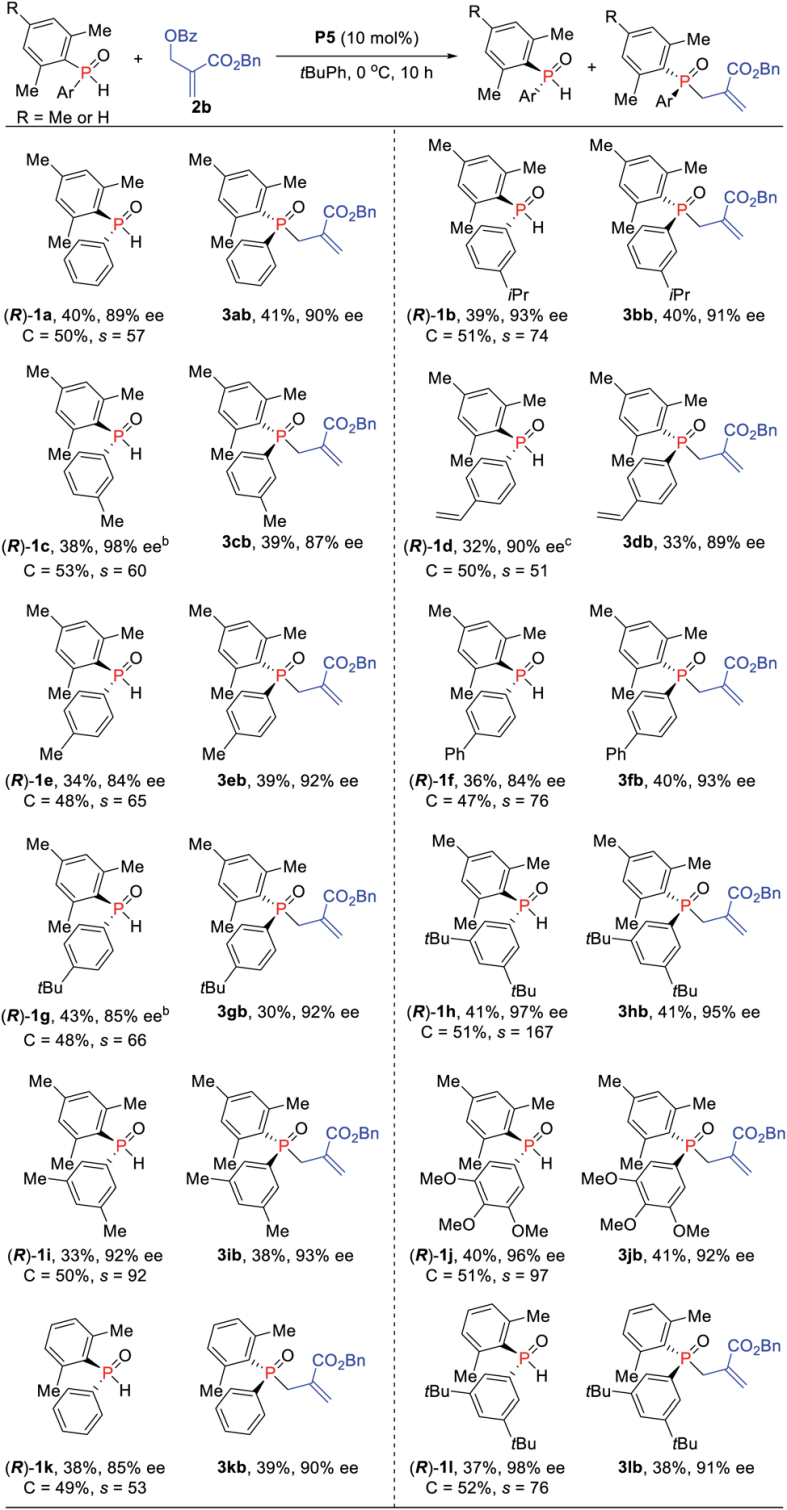
Asymmetric allylic alkylation of SPOs and MBH carbonates. ^*a*^*rac*-**SPO** (0.205 mmol), **2b** (0.1 mmol), and **P5** (10 mol%) in 1.5 mL of *t*BuPh for 10 h at 0 °C. Isolated yields. ^*b*^*rac*-**SPO** (0.21 mol) was used. ^*c*^*rac*-**SPO** (0.22 mol) was used.

Under the optimized reaction conditions, the scope of the kinetic resolution reaction of SPOs was examined by performing asymmetric allylic alkylation in the presence of (*S*_P_,*R*,*S*,*R*_S_)-Le-Phos **P5** (Scheme 2). The steric hindrance of the mesityl group of SPOs has a significant impact on the reaction reactivity and enantioselectivity.^13^ Substrates SPOs with iPr or Me at the *meta*-position of another aromatic ring afforded the corresponding tertiary phosphine oxides **3bb** and **3cb** in 39–40% yields with 87–91% ees, and secondary phosphine oxides **1b** and **1c** were recovered in 38–39% yields with 93–98% ees. A series of SPOs with a *para*-substituted aryl group were well tolerated in this reaction, producing the corresponding allylation products **3db–3gb** in good yields (30–40%) with high enantioselectivities (89–93% ees). The chiral diarylphosphine oxides (*R*)-**1d–1g** could be recovered in 32–43% yields with 84–90% ees, and *s* factors were in the range of 51–76. Multi-substituted aromatic groups such as 3,5-di-*tert*-butylphenyl, 3,5-dimethylphenyl and 3,4,5-trimethoxylphenyl were also compatible, and both the alkylation products and the recovered SPOs were formed in good to excellent enantioselectivities (*s* factors = 92–167).^14^ Fortunately, by the variation of the mesityl group to the 2,5-dimethylphenyl group, the allylic alkylation kinetic resolution reactions gave comparable yields and enantioselectivities ((*R*)-**1k** and (*R*)-**1l**: 37–38% yields, 85–98% ees; **3kb** and **3lb**: 38–39% yields, 90–91% ees; *s* factors = 53–76). The absolute configuration of recovered SPO **1l** was unambiguously confirmed as *R* by X-ray crystallographic analysis.^15^

**Scheme 3:**
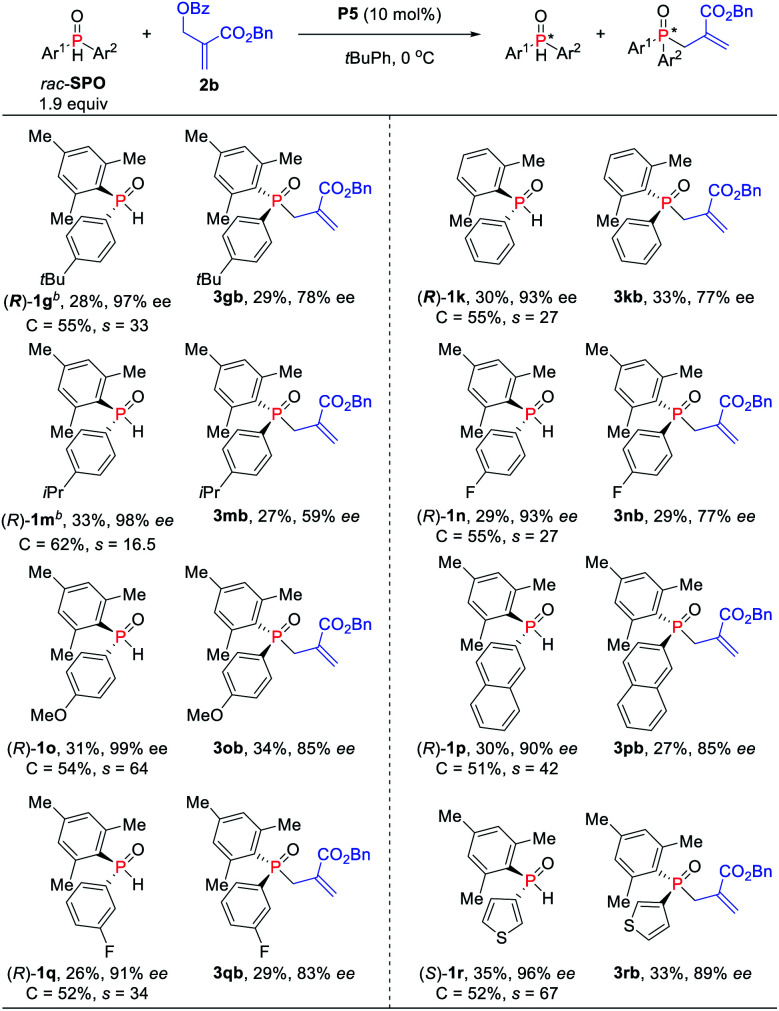
Asymmetric allylic alkylation of SPOs and MBH carbonates. ^*a*^Isolated yields based on the amount of SPOs. ^*b*^*rac*-**SPO** (0.20 mol) was used.

To further increase the enantioselectivity of SPOs, the reaction conditions were modified by reducing the amount of SPOs (Scheme 3). Indeed, the kinetic resolution reaction of SPOs **1g** and **1k** provided higher ees with relatively lower *s* factors (27–33). Moreover, electron-withdrawing and electron-donating aryl groups on the phosphine atom were all tolerated and up to 99% ee was obtained (*s* factors = 17–64). The naphthyl-substituted phosphine oxide **1p** was also kinetically well resolved in 30% yield with 90% ee. The heteroaryl-substituted substrate was compatible with the standard reaction conditions, producing *P*-stereogenic diaryl phosphine oxide in 35% yield and 96% ee, and achieving the corresponding TPO **3rb** in 33% yield and 89% ee (*s* factors = 67).

**Scheme 4:**
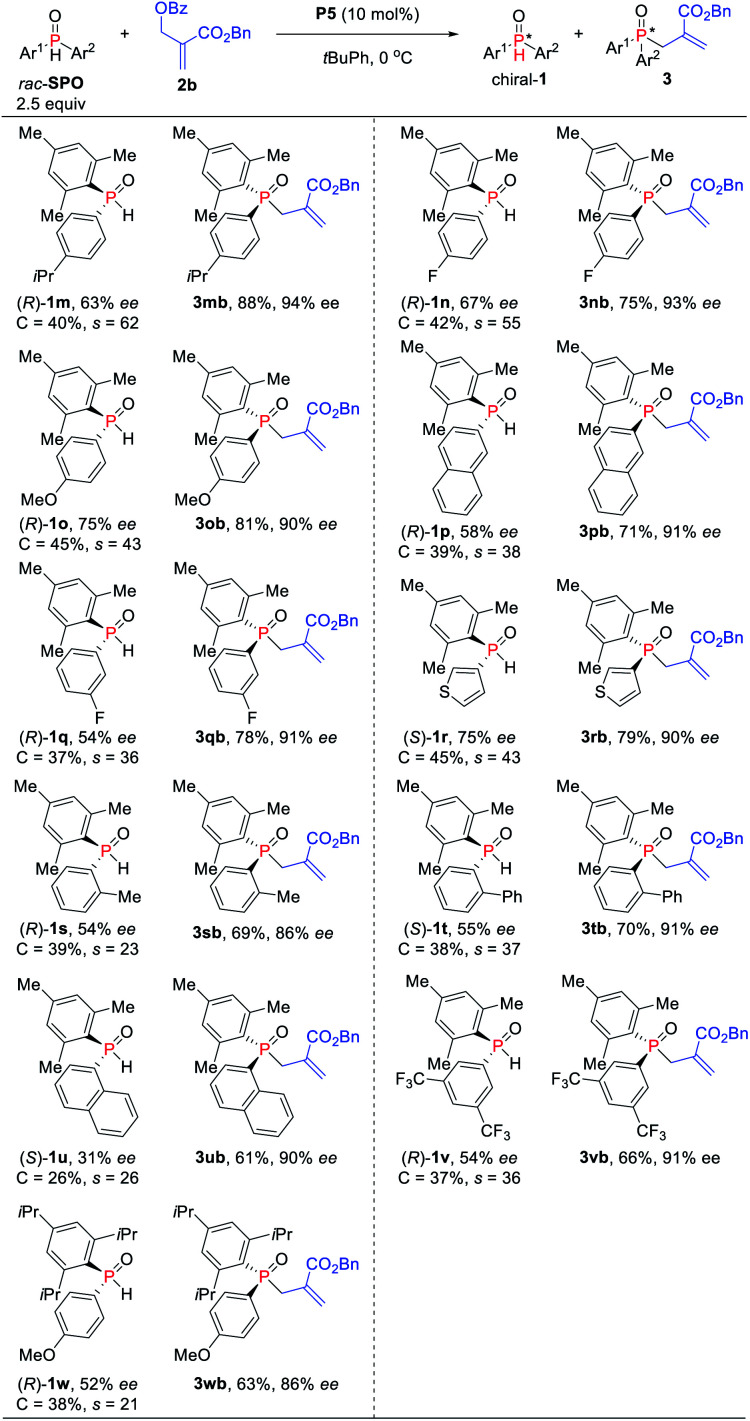
Phosphine-catalyzed asymmetric allylic alkylation of secondary phosphine oxides. ^*a*^Isolated yields of **3** based on the amount of **2b**.

Alternatively, in order to obtain tertiary phosphine oxides with good ee, the asymmetric kinetic resolution of SPOs was further investigated with slightly adjusting the ratio of SPOs to MBH carbonates (2.5 : 1) (Scheme 4). Substrates bearing an *ortho*-, *meta*- or *para*-substituted aryl on the phosphorus center could be accommodated for the reaction, furnishing the corresponding TPOs in 63–83% yields with 86–94% ees (*s*-factors = 23–38). Electron-withdrawing and -donating substituents on the aromatic ring of SPOs have little influence on the reaction yield and enantioselectivity. Naphthyl and thienyl on the SPOs were also compatible with the allylic alkylation process, and good yields with high enantioselectivities were obtained for **3pb** (71%, 91% ee), **3ub** (70%, 90% ee) and **3rb** (79%, 90% ee), respectively. More hindered SPO was found to be less efficient compared to the mesityl-substituted SPO under these conditions (**3ob***vs.***3wb**).

**Scheme 5:**
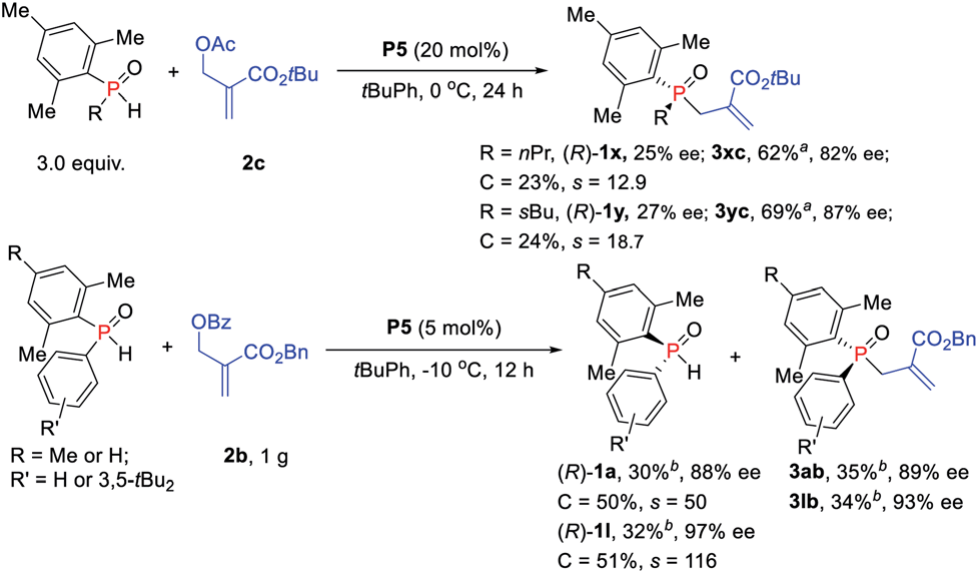
Phosphine-catalyzed asymmetric allylic alkylation of secondary phosphine oxides. ^*a*^Isolated yields based on the amount of **2c**. ^*b*^Isolated yields based on the amount of SPOs.

The reactions of more challenging alkyl-substituted phosphine oxides and MBH carbonate **2c** were carried out with 20 mol% catalyst **P5**, which proceeded well to afford the desired TPOs **3xc–3yc** in moderate yields with 82–87% ees (Scheme 5). To probe the efficiency of the currently studied kinetic resolution strategy in the synthesis of chiral SPOs and TPOs, gram scale reactions were investigated. To our delight, the desired products TPOs (**3ab** and **3lb**) and recovered SPOs ((*R*)-**1a** and (*R*)-**1l**) were obtained without any loss of enantioselectivity even with only 5 mol% catalyst **P5**.

**Scheme 6:**
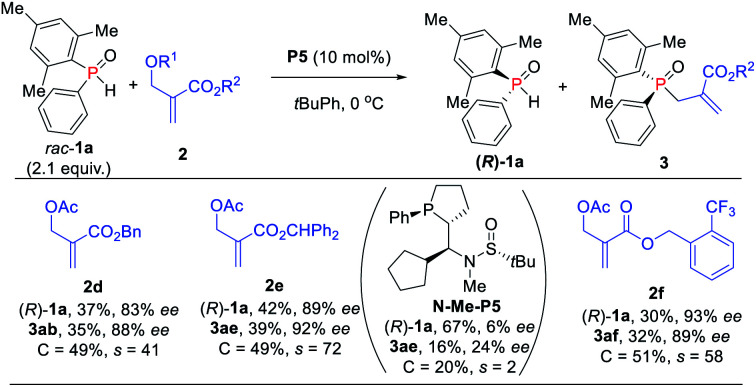
Substrate scope of MBH carbonate substrates.

Further investigation of the substrate scope was focused on MBH carbonate substrates **2** (Scheme 6). The Ac-protected MBH carbonates **2d** afforded the target products **3ab** in 35% yield with 88% ee, and unreacted **1a** was recovered in 37% yield with 83% ee. The benzyl substituted MBH carbonates obtained the corresponding TPOs **3ae–3af** in moderate to excellent yields (32–39%) with excellent enantioselectivities (89–92% ees). The chiral diarylphosphine oxides were also compatible. It is also worth mentioning that we achieved **3ae** in 16% yield with 24% ee *via N*-methylated chiral phosphine catalyst *N*-Me-**P5**, thus indicating that the hydrogen-bonding interaction might indeed play an important role in the catalytic activity and enantioselectivity control.

**Scheme 7:**
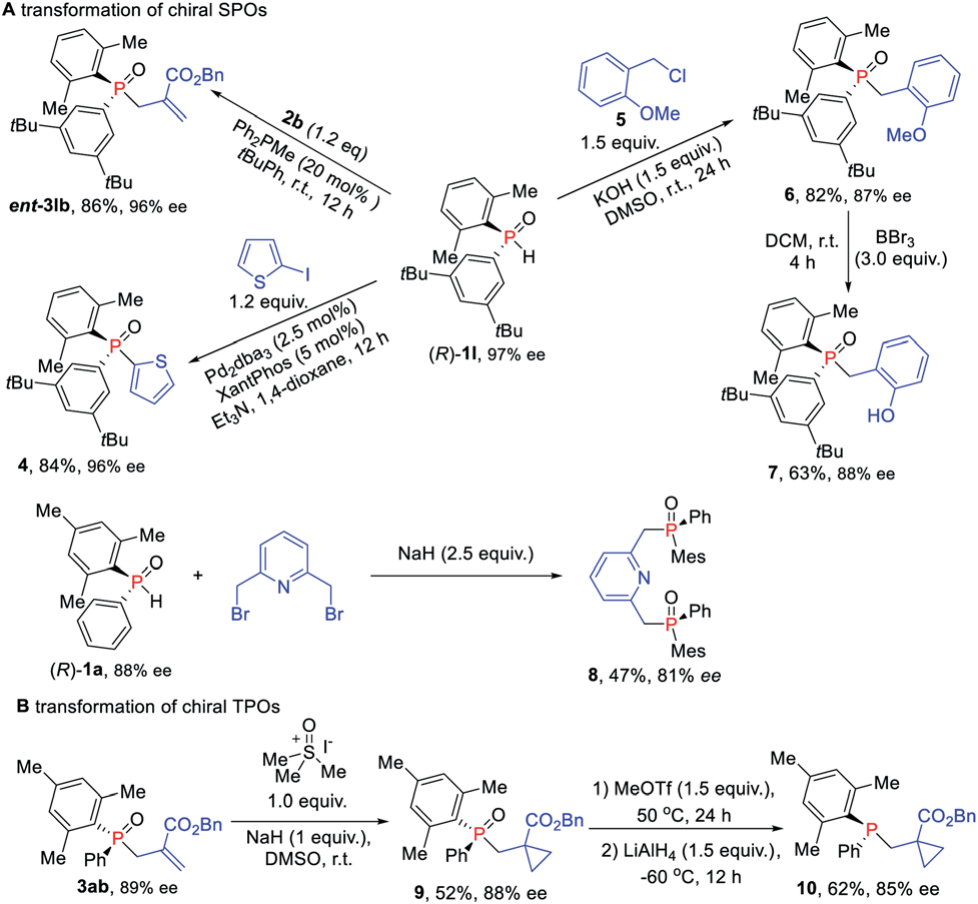
(A) Transformations of chiral SPOs. (B) Transformations of TPOs.

Given their potential of synthetic versatility, various transformations of chiral SPOs were conducted. The chiral SPO **1l** with an *R* configuration at the phosphorus atom underwent an allylic alkylation reaction with MBH carbonate **2b** to provide the enantiomer of TPO **3lb**, that is, *ent*-**3lb** in 86% yield and 96% ee (Scheme 7A). Further cross-coupling of (*R*)-**1l** with 2-iodothiophene under the catalysis of palladium and xantphos afforded the enantioenriched triaryl *P*-stereogenic oxide **4** in 84% yield with 96% ee.^16^ The direct alkylation of (*R*)-**1l** with benzyl chloride in the presence of KOH, furnished the novel TPO **6** in 82% yield with slight erosion of ee to 87% ee.^17^ When (*R*)-**1** was used for this transformation, the corresponding products were obtained without loss ofenantioselectivity.^18^ Then the demethylation of TPO **6** in the presence of BBr_3_ furnished the catalyst **7**, which has potential application in the asymmetric catalytic Mitsunobu reaction.^19^ We also utilized (*R*)-**1a** to synthesize chiral pincer-type ligand **8** in 47% yield with 81% ee. Moreover, we demonstrated the utility of these enantioenriched TPOs through further transformations of the *P*-chiral scaffold. Cyclopropanantion of this enantiopure TPO **3ab** was carried out and the cyclopropane product **9** was converted into *P*-stereogenic phosphine **10** without loss of chirality (Scheme 7B).^16,20^

## Conclusions

In summary, we have developed a novel type of bifunctional chiral sulfinamide cyclic phosphine catalyst Le-Phos, which can be easily prepared in gram scale from inexpensive commercially available starting materials in short steps. (*S*_P_,*R*,*S*,*R*_S_)-Le-Phos has shown excellent performance in the enantioselective γ-addition reactions of various N-centered nucleophiles to γ-substituted allenoates, acquiring a series of γ-addition adducts in high yields with up to 98% ees and excellent regioselectivity and diastereoselectivity under mild conditions. Its prominent characteristics are general substrate scope, mild reaction conditions, good yields, high enantioselectivities, ease of scale-up to gram scale, and further synthetic transformations of the products. Further explorations of Le-Phos as an organocatalyst and chiral ligand of transition metals in asymmetric catalysis are currently underway in our group and will be reported in due course.

## Conflicts of interest

There are no conflicts to declare.

## Supplementary Material

SC-011-D0SC04041J-s001

SC-011-D0SC04041J-s002
