# Supplementary material for: Access to P-chiral sec- and tert-phosphine oxides enabled by Le-Phos-catalyzed asymmetric kinetic resolution
Source: Chem Sci. 2020 Sep 2;11(36):9983–8. doi: 10.1039/d0sc04041j (PMC8162192; doi:10.1039/d0sc04041j)
Supplement: SC-011-D0SC04041J-s001 [file SC-011-D0SC04041J-s001.pdf]

Electronic Supplementary Material (ESI) for Chemical Science.
This journal is © The Royal Society of Chemistry 2020

Supporting Information

Access to P-Chiral *sec*- and *tert*-Phosphine Oxides Enabled by Le-Phos-Catalyzed Asymmetric Kinetic Resolution

Haile Qiu, Qiang Dai, Jiafeng He, Wenbo Li and Junliang Zhang*

Shanghai Key Laboratory of Green Chemistry and Chemical Processes, School of Chemistry and Molecular Engineering, East China Normal University, 3663 N. Zhongshan Road, Shanghai 200062, P. R. China;

Department of Chemistry, Fudan University, 2005 Songhu Road, Shanghai 200438, P.R. China

Email: jlzhang@chem.ecnu.edu.cn; junliangzhang@fudan.edu.cn

1. General Information……………………………………………………………………….S1

2. Optimization of reaction conditions……………………………………………………….S2

3. General procedure for the synthesis of secondary phosphine oxides (SPOs)…………………. S4

4. General procedure for the synthesis of chiral SPOs and the corresponding tertiary phosphine oxides (TPOs)……………………………………………………………………………….... S14

5. Transformation of chiral SPOs and TPOs……………………………………………….S50

6. The X-ray structure of compound (*R*)-**1l**………………………………………………...S61

7. References………………………………………………………………………………….S64

8. NMR spectra……………………………………………………………………………….S65

1. General Information:

Unless otherwise noted, all reactions were carried out under a argon atmosphere; materials obtained from commercial suppliers were used directly without further purification. The [±]D was recorded using PolAAr 3005 High Accuracy Polarimeter. ^{1}H NMR spectra, ^{13}C NMR spectra, and ^{31}P NMR spectra were recorded on a Bruker 400 MHz spectrometer in $CDCl_3$. NMR experiments are reported in δ units, parts per million (ppm), and were referenced to $CDCl_3$ (δ 7.26 or 77.0 ppm) as the internal standard. The data is being reported as (s = singlet, d = doublet, dd = doublet of doublet, t = triplet, m = multiplet or unresolved, br = broad signal, coupling constant(s) in Hz, integration). Trichloromethane ($CHCl_3$), carbon tetrachlorid, dichloromethane, dichloroethane and acetonitrile were freshly distilled from CaH_2; tetrahydrofuran (THF), toluene and ether were dried with sodium benzophenone and distilled before use; Reactions were monitored by thin layer chromatography (TLC) using silicycle pre-coated silica gel plates. Flash column chromatography was performed on silica gel 60 (particle size 300-400 mesh ASTM, purchased from Yantai, China) and eluted with petroleum ether/ethyl acetate.

2. Optimization of reaction conditions:

2.1 Table S-1: Screening of catalysts.[a]

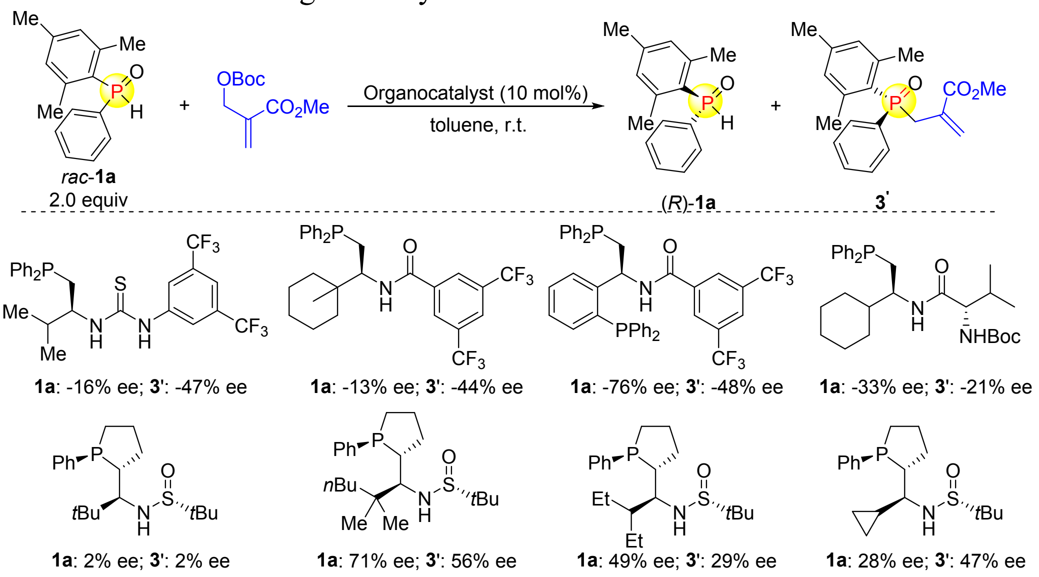

2.2 Table S-2: Screening of MBH carbonates.[a]

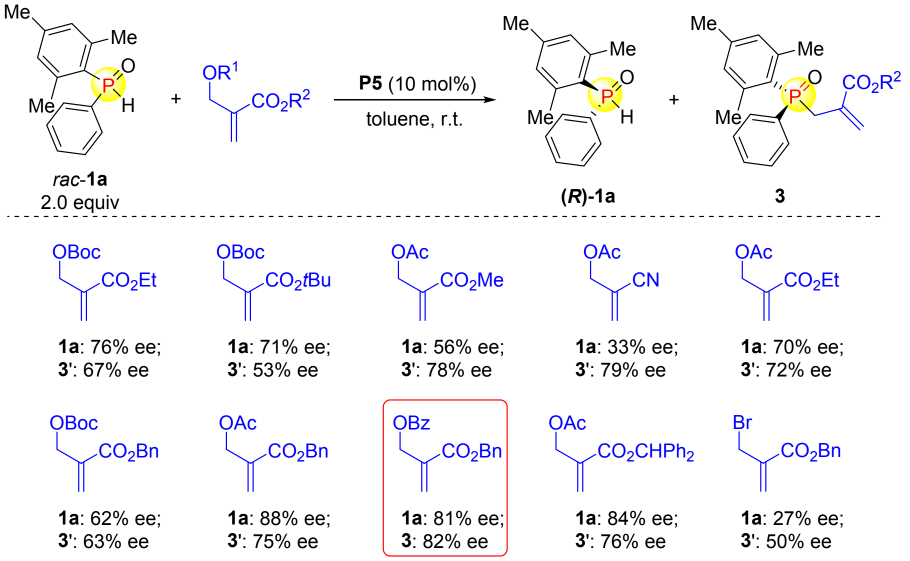

2.3 Table S-3: Experimental comparison of several SPOs.[a]

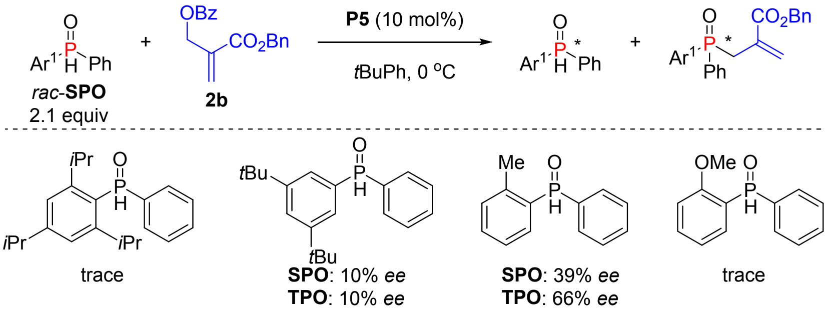

2.4 Table S-4: Experimental comparison of solvent.[a]

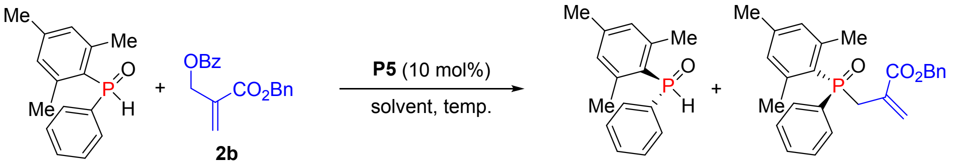

Entry	Solvent	Temp. (°C)	T（h）	Recovery of **1a**	**3ab**	*s* factor
1	1,4-dioxane	r.t.	12	41%, 72% *ee*	37%, 70% *ee*	12
2	Et_2O	r.t.	12	39%, 78% *ee*	40%, 76% *ee*	17.2
3	DCE	r.t.	12	38%, 64% *ee*	37%, 58% *ee*	7.1
4	mesitylene	r.t.	12	35%, 88% *ee*	38%, 78% *ee*	23.4
5	*t*BuPh	r.t.	4	36%, 89% *ee*	44%, 75% *ee*	20.5
6	mesitylene	10	24 h		trace	
7[b]	*t*BuPh	10	8	37%, 93% *ee*	44%, 86% *ee*	44.4
8[b]	*t*BuPh	0	12	92% *ee*	88% *ee*	49
9[c]	*t*BuPh	0	12	86% *ee*	92% *ee*	69
10[d]	*t*BuPh	0	12	77% *ee*	95% *ee*	30

[a]All yields are determined by ^{1}H NMR analysis of the crude mixture. Enantiomeric excesses are determined by HPLC. C (calculated conversion) = $ee_{SM}/(ee_{SM} + ee_{PR})$, *s* (selectivity) = $In[(1 - C)(1 - ee_{SM})]/In[(1 - C)(1 + ee_{SM})]$. [b]*rac*-**SPO** (0.20 mol) was used. [c]*rac*-**SPO** (0.21 mol) was used. [d]*rac*-**SPO** (0.23 mol) was used.

3. General procedure for the synthesis of secondary phosphine oxides (SPOs).

General procedure A[1]:

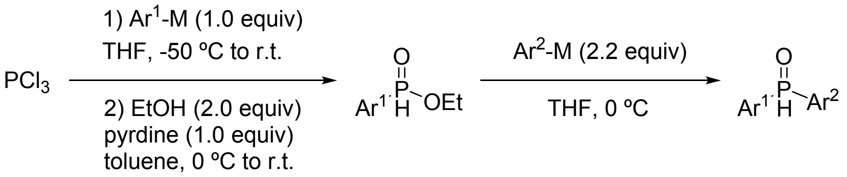

A 500 mL round-bottomed flask equipped with a magnetic stirrer under argon atmosphere was charged with phosphorus trichloride (17.5 mL, 200 mmol) in 100 mL THF and cooled to -50 °C, the appropriate organometallic reagent (1.0 equiv.) in THF was added dropwise at -50 °C over 30 mins. The reaction was sirred at -50 °C for 4 h and then warmed to room temperature for another 1 h, then concentrated in vacuo. A 250 mL round-bottomed flask equipped with a magnetic stirrer under air atmosphere was charged with the crude residue in 100 mL toluene, a solution of EtOH (18.4 g, 400 mmol) and pyridine (16.1 mL, 200 mmol) was added dropwise over 30 mins. The reaction was sirred at 0 °C for 0.5 h and then warmed to room temperature for 2 hour Water (100 mL) was then added and the aqueous phase was then extracted with EtOAc (3 × 50 mL). The combined organic fractions were dried over anhydrous Na_2SO_4, concentrated in vacuo, and the crude residue purified by column chromatography to afford phosphonate.

A 50 mL round-bottomed flask equipped with a magnetic stirrer under argon atmosphere was charged with the appropriate organometallic reagent (2.2 equiv) in THF and cooled to 0 °C, ethyl phosphinate (10.0 mmol) in THF (20 mL) was added dropwise at 0 °C over 10 mins. The reaction was stirred at 0 °C for 4 h then quenched with sat. aq. NH_4Cl solution. The aqueous phase was then extracted with EtOAc (3 × 20 mL). The combined organic fractions were dried over anhydrous Na_2SO_4, concentrated in vacuo, and the crude residue purified by column chromatography to afford the desired secondary phosphine oxide.

General procedure B[1]:

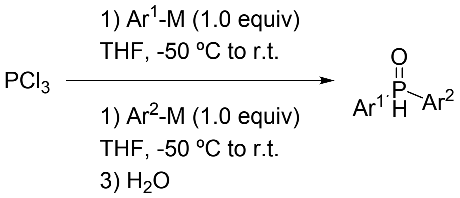

A 250 mL round-bottomed flask equipped with a magnetic stirrer under argon atmosphere was charged with phosphorus trichloride (1.8 mL, 20 mmol) in 100 mL THF and cooled to -50 °C, the appropriate organometallic reagent (1.0 equiv.) in THF was added dropwise at -50 °C over 30 mins. The reaction was sirred at -50 °C for 4 h and then warmed to room temperature for another 1 h. Then the flask was placed at -50 °C. the appropriate organometallic reagent (1.0 equiv.) in THF was added dropwise at -50 °C over 30 mins. The reaction was sirred at -50 °C for 1 h and then warmed to room temperature for 4 h, then cooled to -50 °C. Deoxygenated water (10 mL) was added dropwise at 0 °C over 5 mins. The reaction was sirred at room temperature for 1 h. Water (20 mL) was then added and the aqueous phase was then extracted with EtOAc (3 × 10 mL). The combined organic fractions were dried over anhydrous Na_2SO_4, concentrated in vacuo, and the crude residue purified by column chromatography to afford the desired secondary phosphine oxide.

3.1 mesityl(phenyl)phosphine oxide (1a)

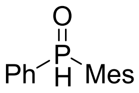

1a

1a was prepared as a white solid following general procedure A. ^{1}H NMR (400 MHz, $CDCl_3$) δ 8.55 (d, J = 483.0 Hz, 1H), 7.66 – 7.58 (m, 2H), 7.56 – 7.49 (m, 1H), 7.49 – 7.42 (m, 2H), 6.91 (d, J = 3.9 Hz, 2H), 2.45 (s, 6H), 2.31 (s, 3H). ^{31}P NMR (162 MHz, $CDCl_3$) δ 9.86. ^{13}C NMR (101 MHz, $CDCl_3$) δ 142.78 (d, J = 2.3 Hz), 142.08 (d, J = 10.0 Hz), 132.26 (d, J = 99.0 Hz), 131.87 (d, J = 2.9 Hz), 130.49 (d, J = 11.2 Hz), 130.28 (d, J = 10.5 Hz), 128.77 (d, J = 12.6 Hz), 124.32 (d, J = 102.9 Hz), 21.44 (d, J = 8.5 Hz), 21.28 (d, J = 0.9 Hz). HRMS (ESI) calcd. For $C_{15}H_{17}NaOP$ $[M+Na]^+$: 267.0909, found: 267.0917.

3.2 (3-isopropylphenyl)(mesityl)phosphine oxide (1b).

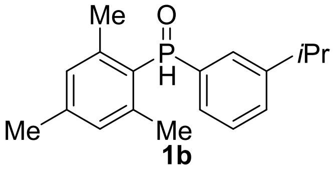

1b

1b was prepared as a white solid following general procedure A. ^{1}H NMR (400 MHz, $CDCl_3$) δ 8.53 (d, J = 481.7 Hz, 1H), 7.63 (d, J = 13.8 Hz, 1H), 7.40 – 7.26 (m, 3H), 6.90 (d, J = 3.7 Hz, 2H), 2.99 – 2.87 (m, 1H), 2.45 (s, 6H), 2.30 (s, 3H), 1.23 (d, J = 7.0 Hz, 6H). ^{31}P NMR (162 MHz, $CDCl_3$) δ 10.35. ^{13}C NMR (101 MHz, $CDCl_3$) δ 149.67 (d, J = 11.6 Hz), 142.62 (d, J = 2.4 Hz), 142.06 (d, J = 9.9 Hz), 132.15 (d, J = 98.7 Hz), 130.24 (d, J = 10.5 Hz), 130.01 (d, J = 2.9 Hz), 128.95 (d, J = 10.4 Hz), 128.73 (d, J = 13.5 Hz), 127.54 (d, J = 12.3 Hz), 124.54 (d, J = 102.4 Hz), 34.09, 23.79 (d, J = 3.5 Hz), 21.44 (dd, J = 8.3, 2.5 Hz), 21.25 (d, J = 2.3 Hz). HRMS (ESI) calcd. For $C_{18}H_{23}NaOP$ $[M+Na]^+$: 309.1379, found: 309.1371.

3.3 mesityl(*m*-tolyl)phosphine oxide (1c).

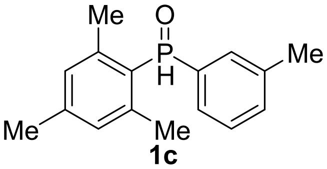

1c

1c was prepared as a white solid following general procedure A. ^{1}H NMR (500 MHz, $CDCl_3$) δ 8.53 (d, J = 481.6 Hz, 1H), 7.52 (d, J = 13.6 Hz, 1H), 7.39 – 7.33 (m, 1H), 7.33 – 7.30 (m, 2H), 6.90 (d, J = 3.9 Hz, 2H), 2.47 (s, 6H), 2.36 (s, 3H), 2.30 (s, 3H). ^{31}P NMR (202 MHz, $CDCl_3$) δ 9.93. ^{13}C NMR (126 MHz, $CDCl_3$) δ 142.42 (d, J = 2.3 Hz), 141.78 (d, J = 10.0 Hz), 138.44 (d, J = 12.4 Hz), 132.47 (d, J = 3.0 Hz), 131.87 (d, J = 98.7 Hz), 130.73 (d, J = 10.8 Hz), 130.02 (d, J = 10.4 Hz), 128.43 (d, J = 13.4 Hz), 127.13 (d, J = 11.6 Hz), 124.23 (d, J = 102.5 Hz), 21.25, 21.14 (d, J = 10.0 Hz), 21.03 (d, J = 0.7 Hz). HRMS (ESI) calcd. For $C_{16}H_{19}NaOP$ $[M+Na]^+$: 281.1066, found: 281.1071.

3.4 mesityl(4-vinylphenyl)phosphine oxide (1d).

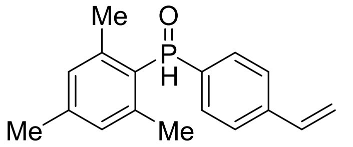

1d

1d was prepared as a white solid following general procedure B. ^{1}H NMR (500 MHz, $CDCl_3$) δ 8.49 (d, J = 483.3 Hz, 1H), 7.52 (dd, J = 13.0, 8.1 Hz, 2H), 7.40 (dd, J = 8.0, 1.8 Hz, 2H), 6.83 (d, J = 3.6 Hz, 2H), 6.64 (dd, J = 17.6, 10.9 Hz, 1H), 5.76 (d, J = 17.6 Hz, 1H), 5.28 (d, J = 10.9 Hz, 1H), 2.39 (s, 6H), 2.23 (s, 3H). ^{31}P NMR (202 MHz, $CDCl_3$) δ 9.55. ^{13}C NMR (126 MHz, $CDCl_3$) δ 142.53 (d, J = 2.3 Hz), 141.75 (d, J = 10.0 Hz), 140.74 (d, J = 2.9 Hz), 135.57 (d, J = 1.0 Hz), 130.52 (d, J = 11.4 Hz), 130.04 (d, J = 10.4 Hz), 126.24 (d, J = 13.0 Hz), 124.05 (d, J = 102.9 Hz), 116.21, 21.19 (d, J = 8.5 Hz), 21.02. HRMS (ESI) calcd. For $C_{17}H_{19}NaOP$ $[M+Na]^+$: 293.1066, found: 293.1069.

3.5 mesityl(p-tolyl)phosphine oxide (1e).

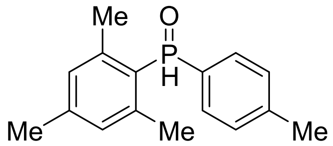

1e

1e was prepared as a white solid following general procedure A. ^{1}H NMR (400 MHz, $CDCl_3$) δ 8.53 (d, J = 482.1 Hz, 1H), 7.51 (dd, J = 13.1, 7.7 Hz, 2H), 7.26 (d, J = 7.4 Hz, 2H), 6.90 (d, J = 3.2 Hz, 2H), 2.45 (s, 6H), 2.39 (s, 3H), 2.31 (s, 3H). ^{31}P NMR (162 MHz, $CDCl_3$) δ 10.10. ^{13}C NMR (101 MHz, $CDCl_3$) δ 142.56 (d, J = 2.4 Hz), 142.35 (d, J = 3.0 Hz), 141.96 (d, J = 10.0 Hz), 130.44 (d, J = 11.6 Hz), 130.18 (d, J = 10.4 Hz), 129.47 (d, J = 13.0 Hz), 128.83 (d, J = 101.3 Hz), 125.33 (d, J = 69.7 Hz), 123.97, 21.52 (d, J = 1.0 Hz), 21.36 (d, J = 8.4 Hz), 21.21 (d, J = 0.5 Hz). HRMS (ESI) calcd. For $C_{16}H_{19}NaOP$ $[M+Na]^+$: 281.2102, found: 281.2106.

3.6 [1,1'-biphenyl]-4-yl(mesityl)phosphine oxide (1f).

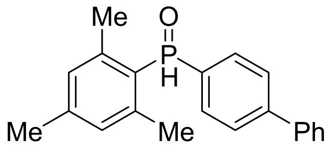

1f

1f was prepared as a white solid following general procedure B. ^{1}H NMR (400 MHz, $CDCl_3$) δ 8.61 (d, *J* = 483.3 Hz, 1H), 7.73 – 7.66 (m, 4H), 7.61 – 7.57 (m, 2H), 7.49 – 7.43 (m, 2H), 7.41 – 7.36 (m, 1H), 6.93 (d, *J* = 3.9 Hz, 2H), 2.50 (s, 6H), 2.33 (s, 3H). ^{31}P NMR (162 MHz, $CDCl_3$) δ 9.70. ^{13}C NMR (101 MHz, $CDCl_3$) δ 144.78 (d, *J* = 3.0 Hz), 142.86 (d, *J* = 2.4 Hz), 142.16 (d, *J* = 10.0 Hz), 139.93 (d, *J* = 0.9 Hz), 131.07 (d, *J* = 11.5 Hz), 130.37 (d, *J* = 10.4 Hz), 128.98, 128.18, 127.53 (d, *J* = 12.9 Hz), 127.27, 124.43 (d, *J* = 102.8 Hz), 21.56 (d, *J* = 8.5 Hz), 21.36 (d, *J* = 0.6 Hz). HRMS (ESI) calcd. For $C_{21}H_{22}OP$ $[M+H]^+$: 321.1403, found: 321.1400.

3.7 4-(*tert*-butyl)phenyl)(mesityl)phosphine oxide (1g).

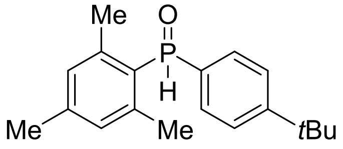

1g

1g was prepared as a white solid following general procedure B. ^{1}H NMR (500 MHz, $CDCl_3$) δ 8.54 (d, *J* = 481.8 Hz, 1H), 7.57 – 7.51 (m, 2H), 7.46 (m, 2H), 6.89 (d, *J* = 2.9 Hz, 2H), 2.46 (s, 6H), 2.29 (s, 3H), 1.30 (s, 9H). ^{31}P NMR (202 MHz, $CDCl_3$) δ 10.02. ^{13}C NMR (126 MHz, $CDCl_3$) δ 155.44 (d, *J* = 2.9 Hz), 142.56 (d, *J* = 2.3 Hz), 142.03 (d, *J* = 9.9 Hz), 130.37 (d, *J* = 11.5 Hz), 130.23 (d, *J* = 10.4 Hz), 128.90 (d, *J* = 101.2 Hz), 125.79 (d, *J* = 12.8 Hz), 124.53 (d, *J* = 102.5 Hz), 34.97, 31.07, 21.44 (d, *J* = 8.4 Hz), 21.26 (d, *J* = 0.5 Hz). HRMS (ESI) calcd. For $C_{19}H_{25}NaOP$ $[M+Na]^+$: 323.1535, found: 323.1539.

3.8 (3,5-di-tert-butylphenyl)(mesityl)phosphine oxide (1h).

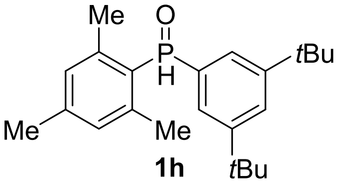

1h

1h was prepared as a white solid following general procedure B. H NMR (500 MHz, $CDCl_3$) δ 8.53 (d, *J* = 480.2 Hz, 1H), 7.56 (d, *J* = 1.4 Hz, 1H), 7.47 (d, *J* = 1.8 Hz, 1H), 7.44 (d, *J* = 1.7 Hz, 1H), 6.91 (d, *J* = 3.7 Hz, 2H), 2.48 (s, 6H), 2.32 (s, 3H), 1.28 (s, 18H). ^{31}P NMR (202 MHz, $CDCl_3$) δ 11.91. ^{13}C NMR (126 MHz, $CDCl_3$) δ 151.47 (d, *J* = 12.4 Hz), 142.44 (d, *J* = 2.4 Hz), 141.96 (d, *J* = 9.9 Hz), 131.29 (d, *J* = 99.4 Hz), 130.21 (d, *J* = 10.4 Hz), 129.44, 126.07 (d, *J* = 2.8 Hz), 124.48 (d, *J* = 12.1 Hz), 115.42, 35.00, 31.26, 21.51 (d, *J* = 8.2 Hz), 21.29. HRMS (ESI) calcd. For $C_{23}H_{33}NaOP$ $[M+Na]^+$: 379.2161, found: 379.2155.

3.9 (3,5-dimethylphenyl)(mesityl)phosphine oxide (1i).

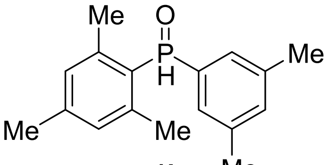

1i

1i was prepared as a white solid following general procedure B. ^{1}H NMR (400 MHz, $CDCl_3$) δ 8.50 (d, *J* = 481.2 Hz, 1H), 7.24 (d, *J* = 13.8 Hz, 2H), 7.13 (s, 1H), 6.90 (d, *J* = 3.6 Hz, 2H), 2.47 (s, 6H), 2.31 (s, 6H), 2.30 (s, 3H). ^{31}P NMR (162 MHz, $CDCl_3$) δ 10.25. ^{13}C NMR (101 MHz, $CDCl_3$) δ 142.34 (d, *J* = 2.3 Hz), 141.78 (d, *J* = 10.0 Hz), 138.32 (d, *J* = 13.3 Hz), 133.44 (d, *J* = 2.9 Hz), 131.71 (d, *J* = 98.6 Hz), 130.01 (d, *J* = 10.4 Hz), 127.71 (d, *J* = 11.2 Hz), 124.31 (d, *J* = 102.3 Hz), 21.23 (d, *J* = 8.4 Hz), 21.02, 20.95. HRMS (ESI) calcd. For $C_{17}H_{21}NaOP$ $[M+Na]^+$: 295.1222, found: 295.1229.

3.10 mesityl(3,4,5-trimethoxyphenyl)phosphine oxide (1j).

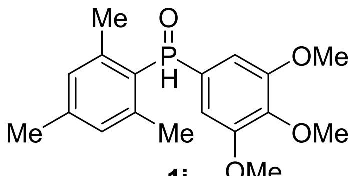

1j

1j was prepared as a white solid following general procedure B. ^{1}H NMR (400 MHz, $CDCl_3$) δ 8.49 (d, *J* = 484.7 Hz, 1H), 6.90 (d, *J* = 3.8 Hz, 2H), 6.80 (d, *J* = 14.6 Hz, 2H), 3.86 (s, 3H), 3.80 (s, 6H), 2.46 (s, 6H), 2.30 (s, 3H). ^{31}P NMR (162 MHz, $CDCl_3$) δ 10.10. ^{13}C NMR (101 MHz, $CDCl_3$) δ 153.67 (d, *J* = 18.2 Hz), 142.80 (d, *J* = 2.4 Hz), 142.04 (d, *J* = 10.0 Hz), 141.00 (d, *J* = 2.8 Hz), 130.27 (d, *J* = 10.4 Hz), 126.83 (d, *J* = 101.2 Hz), 124.09 (d, *J* = 103.2 Hz), 107.22 (d, *J* = 13.0 Hz), 60.86, 56.29 (d, *J* = 2.1 Hz), 29.19, 21.43 (dd, *J* = 8.4, 1.3 Hz), 21.27. HRMS (ESI) calcd. For $C_{18}H_{23}NaO_4P$ $[M+Na]^+$: 295.1222, found: 295.1229.

3.11 (2,6-dimethylphenyl)(phenyl)phosphine oxide (1k).

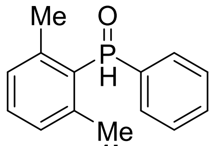

1k

1k was prepared as a white solid following general procedure B. ^{1}H NMR (500 MHz, $CDCl_3$) δ 8.57 (d, *J* = 484.6 Hz, 1H), 7.65 – 7.58 (m, 2H), 7.57 – 7.50 (m, 1H), 7.47 – 7.43 (m, 2H), 7.32 (t, *J* = 7.6 Hz, 1H), 7.07 (dd, *J* = 7.6, 4.2 Hz, 2H), 2.48 (s, 6H). ^{31}P NMR (202 MHz, $CDCl_3$) δ 9.85. ^{13}C NMR (126 MHz, $CDCl_3$) δ 142.06 (d, *J* = 9.7 Hz), 132.38 (d, *J* = 2.3 Hz), 132.00 (d, *J* = 3.0 Hz), 131.99 (d, *J* = 9.8 Hz), 130.42 (d, *J* = 11.3 Hz), 129.45 (d, *J* = 10.1 Hz), 128.82 (d, *J* = 12.6 Hz), 127.39 (d, *J* = 100.5 Hz), 21.52 (d, *J* = 8.6 Hz). HRMS (ESI) calcd. For $C_{14}H_{15}NaOP$ $[M+Na]^+$: 253.0753, found: 253.0760.

3.12 (3,5-di-tert-butylphenyl)(2,6-dimethylphenyl)phosphine oxide (1l).

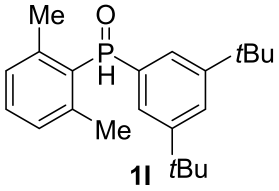

1l

1l was prepared as a white solid following general procedure B. ^{1}H NMR (400 MHz, $CDCl_3$) δ 8.58 (d, *J* = 482.0 Hz, 1H), 7.57 (s, 1H), 7.45 (dd, *J* = 14.4, 1.6 Hz, 2H), 7.31 (t, *J* = 7.6 Hz, 1H), 7.08 (dd, *J* = 7.4, 4.1 Hz, 2H), 2.52 (s, 6H), 1.27 (s, 18H). ^{31}P NMR (162 MHz, $CDCl_3$) δ 11.75. ^{13}C NMR (101 MHz, $CDCl_3$) δ 151.50 (d, *J* = 12.4 Hz), 141.96 (d, *J* = 9.5 Hz), 132.13 (d, *J* = 2.2 Hz), 130.96 (d, *J* = 99.1 Hz), 129.35 (d, *J* = 10.0 Hz), 127.85 (d, *J* = 99.4 Hz), 126.15 (d, *J* = 2.9 Hz), 124.44 (d, *J* = 12.2 Hz), 34.97, 31.21, 21.58 (d, *J* = 8.4 Hz). HRMS (ESI) calcd. For $C_{22}H_{31}NaOP$ $[M+Na]^+$: 365.2002, found: 365.2005.

3.13 (4-isopropylphenyl)(mesityl)phosphine oxide (1m).

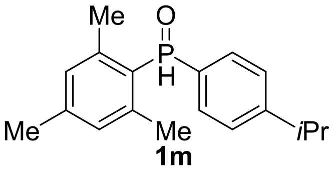

1m

1m was prepared as a white solid following general procedure B. ^{1}H NMR (400 MHz, $CDCl_3$) δ 8.53 (d, *J* = 482.1 Hz, 1H), 7.53 (dd, *J* = 13.2, 8.1 Hz, 2H), 7.29 (dd, *J* = 8.2, 2.5 Hz, 2H), 6.89 (d, *J* = 3.8 Hz, 2H), 3.00 – 2.85 (m, 1H), 2.45 (s, 6H), 2.29 (s, 3H), 1.23 (d, *J* = 6.9 Hz, 6H). ^{31}P NMR (162 MHz, $CDCl_3$) δ 10.11. ^{13}C NMR (101 MHz, $CDCl_3$) δ 153.14 (d, *J* = 2.9 Hz), 142.54 (d, *J* = 2.4 Hz), 141.98 (d, *J* = 9.9 Hz), 130.56 (d, *J* = 11.5 Hz), 130.18 (d, *J* = 10.4 Hz), 129.19 (d, *J* = 101.1 Hz), 126.92 (d, *J* = 12.9 Hz), 124.47 (d, *J* = 102.5 Hz), 34.11, 23.62 (d, *J* = 2.1 Hz), 21.39 (d, *J* = 8.4 Hz), 21.21 (d, *J* = 0.7 Hz). HRMS (ESI) calcd. For $C_{18}H_{23}NaOP$ $[M+Na]^+$: 309.2001, found: 309.2003.

3.14 (4-fluorophenyl)(mesityl)phosphine oxide (1n).

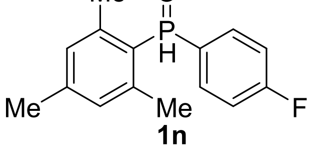

1n

1n was prepared as a white solid following general procedure B. ^{1}H NMR (500 MHz, $CDCl_3$) δ 9.18 – 7.91 (m, 1H), 7.75 – 7.55 (m, 2H), 7.22 – 7.09 (m, 2H), 6.90 (dd, *J* =

8.7, 3.2 Hz, 2H), 2.44 (dd, J = 8.0, 4.0 Hz, 6H), 2.30 (dd, J = 9.9, 4.9 Hz, 3H). ^{31}P NMR (202 MHz, $CDCl_3$) δ 8.69. ^{19}F NMR (376 MHz, $CDCl_3$) δ -106.68 (d, J = 1.6 Hz). ^{13}C NMR (126 MHz, $CDCl_3$) δ 165.04 (d, J = 253.2 Hz), 142.99, 142.06 (d, J = 10.0 Hz), 134.64 – 134.24 (m), 133.01 (dd, J = 12.6, 8.9 Hz), 130.39 (d, J = 10.5 Hz), 128.58 (d, J = 2.3 Hz), 127.78 (d, J = 2.9 Hz), 124.10 (d, J = 103.5 Hz), 116.16 (dt, J = 21.6, 13.2 Hz), 21.39 (d, J = 8.5 Hz), 21.29. HRMS (ESI) calcd. For $C_{15}H_{16}FNaOP$ $[M+Na]^+$: 285.0834, found: 285.0831.

3.15 mesityl(4-methoxyphenyl)phosphine oxide (1o)

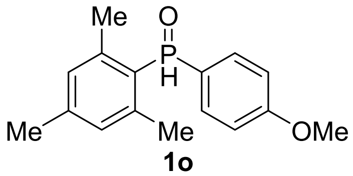

1o

1o was prepared as a white solid following general procedure B. ^{1}H NMR (400 MHz, $CDCl_3$) δ 8.53 (d, J = 483.4 Hz, 1H), 7.61 – 7.46 (m, 2H), 6.95 (d, J = 7.2 Hz, 2H), 6.89 (s, 2H), 3.81 (s, 3H), 2.45 (s, 6H), 2.30 (s, 3H). ^{31}P NMR (162 MHz, $CDCl_3$) δ 10.00. ^{13}C NMR (101 MHz, $CDCl_3$) δ 162.27 (d, J = 2.3 Hz), 142.34, 141.72 (d, J = 9.8 Hz), 132.10 (d, J = 12.5 Hz), 130.01 (d, J = 10.2 Hz), 124.23 (d, J = 102.8 Hz), 122.77 (d, J = 105.4 Hz), 114.16 (d, J = 13.6 Hz), 55.03 (d, J = 2.0 Hz), 21.09 (d, J = 8.0 Hz), 20.99. HRMS (ESI) calcd. For $C_{16}H_{19}NaO_2P$ $[M+Na]^+$: 297.1015, found: 297.1015.

3.16 mesityl(naphthalen-2-yl)phosphine oxide (1p).

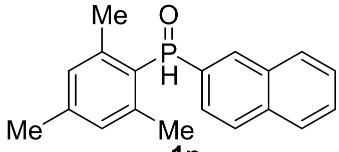

1p

1p was prepared as a white solid following general procedure B. ^{1}H NMR (500 MHz, $CDCl_3$) δ 8.67 (d, J = 482.5 Hz, 1H), 8.31 (d, J = 14.9 Hz, 1H), 7.94 – 7.83 (m, 3H), 7.62 – 7.53 (m, 2H), 7.53 – 7.46 (m, 1H), 6.92 (d, J = 3.2 Hz, 2H), 2.49 (s, 6H), 2.32 (s, 3H). ^{31}P NMR (202 MHz, $CDCl_3$) δ 9.52. ^{13}C NMR (126 MHz, $CDCl_3$) δ 142.81

(d, J = 2.3 Hz), 142.11 (d, J = 10.0 Hz), 134.71 (d, J = 2.4 Hz), 132.62 (d, J = 13.7 Hz), 132.58 (d, J = 9.8 Hz), 130.30 (d, J = 10.5 Hz), 129.34 (d, J = 99.1 Hz), 128.70, 128.59, 128.07, 127.84, 126.92, 124.96 (d, J = 12.9 Hz), 124.42 (d, J = 102.9 Hz), 21.48 (d, J = 8.4 Hz), 21.27 (d, J = 0.7 Hz). HRMS (ESI) calcd. For $C_{19}H_{19}NaOP$ $[M+Na]^+$: 317.1066, found: 317.1073.

3.17 (3-fluorophenyl)(mesityl)phosphine oxide (1q).

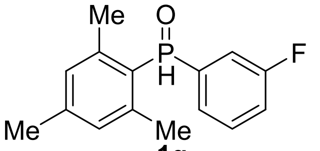

1q

1q was prepared as a white solid following general procedure B. ^1H NMR (500 MHz, $CDCl_3$) δ 8.55 (d, J = 486.6 Hz, 1H), 7.49 – 7.31 (m, 3H), 7.24 – 7.12 (m, 1H), 6.92 (d, J = 4.0 Hz, 2H), 2.47 (s, 6H), 2.31 (s, 3H). ^{31}P NMR (202 MHz, $CDCl_3$) δ 8.07 (d, J = 5.7 Hz). ^{19}F NMR (376 MHz, $CDCl_3$) δ -110.83 (d, J = 5.8 Hz). ^{13}C NMR (126 MHz, $CDCl_3$) δ 162.39 (dd, J = 250.6, 17.3 Hz), 142.84 (d, J = 2.4 Hz), 141.76 (d, J = 10.1 Hz), 134.88 (dd, J = 97.1, 5.4 Hz), 130.52 (dd, J = 14.5, 7.4 Hz), 130.10 (d, J = 10.6 Hz), 125.84 (dd, J = 10.7, 3.2 Hz), 123.49 (d, J = 103.8 Hz), 118.75 (dd, J = 21.2, 2.5 Hz), 117.07 (dd, J = 22.2, 11.9 Hz), 21.08 (d, J = 8.6 Hz), 20.96 (d, J = 0.6 Hz). HRMS (ESI) calcd. For $C_{15}H_{16}FNaOP$ $[M+Na]^+$: 285.0815, found: 285.0819.

3.18 mesityl(thiophen-3-yl)phosphine oxide (1r).

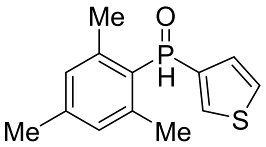

1r

1r was prepared as a brown solid following general procedure B. ^1H NMR (400 MHz, $CDCl_3$) δ 8.55 (d, J = 490.7 Hz, 1H), 7.73 (dd, J = 8.1, 2.1 Hz, 1H), 7.41 – 7.34 (m, 1H), 7.11 (dd, J = 6.7, 2.5 Hz, 1H), 6.86 (d, J = 3.8 Hz, 2H), 2.43 (s, 6H), 2.26 (s, 3H). ^{31}P NMR (162 MHz, $CDCl_3$) δ 2.44. ^{13}C NMR (101 MHz, $CDCl_3$) δ 142.59 (d, J = 2.3 Hz), 141.67 (d, J = 10.3 Hz), 133.70 (d, J = 15.1 Hz), 133.60 (d, J = 102.0 Hz),

130.17 (d, J = 10.6 Hz), 128.04 (d, J = 16.9 Hz), 127.67 (d, J = 15.5 Hz), 124.51 (d, J = 104.8 Hz), 21.10, 21.03 (d, J = 2.7 Hz). For $C_{13}H_{15}NaOPS$ $[M+Na]^+$: 273.0381, found: 285.0386.

4. General procedure for the synthesis of chiral SPOs and the corresponding tertiary phosphine oxides (TPOs):

General procedure A:

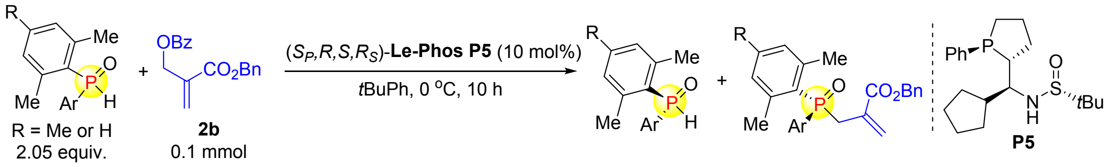

To a flame-dried glass tube with a magnetic stirring bar were added SPO (0.205 mmol) and (S_P, R, S, R_S)-**P5** (3.6 mg, 0.01 mmol), followed by the addition of *t*BuPh (1.5 mL). Then MBH carbonates **2b** (29.6 mg, 0.10 mmol) was slowly added via syringe at 0 °C under inert atmosphere. The reaction mixture was stirred for 10 h, and TLC show that the reaction was completed. Then H_2O_2 (1 drop, 30%) was added to the mixture. The mixture was directly purified by column chromatography on silica gel (petroleum ether/ethyl acetate = 1:1) to afford TPOs (yield was based on the amount of SPOs) and the corresponding chiral SPOs (yield of recovery was based on the amount of SPOs).

General procedure B:

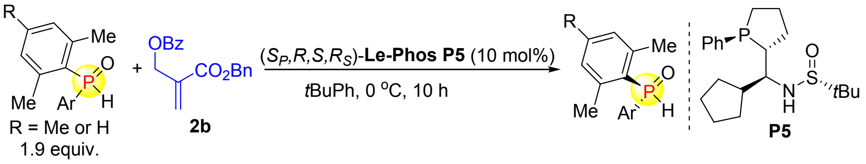

To a flame-dried glass tube with a magnetic stirring bar were added SPO (0.19 mmol) and (S_P, R, S, R_S)-**P5** (3.6 mg, 0.01 mmol), followed by the addition of *t*BuPh (1.5 mL). Then MBH carbonates **2b** (29.6 mg, 0.10 mmol) was slowly added via syringe at 0 °C under inert atmosphere. The reaction mixture was stirred for 10 h, and TLC show that the reaction was completed. Then H_2O_2 (1 drop, 30%) was added to the mixture. The mixture was directly purified by column chromatography on silica gel (petroleum

ether/ethyl acetate = 1:1) to afford the corresponding chiral SPOs (yield of recovery was based on the amount of SPOs).

General procedure C:

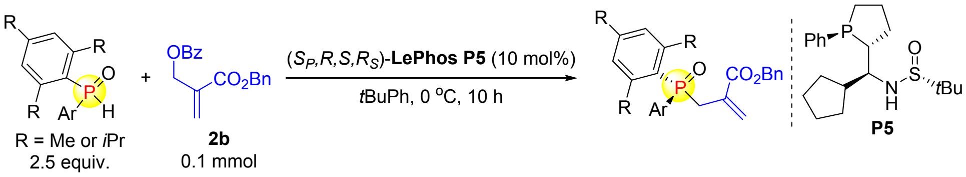

To a flame-dried glass tube with a magnetic stirring bar were added SPO (0.25 mmol) and (S_P, R, S, R_S)-**P5** (3.6 mg, 0.01 mmol), followed by the addition of *t*BuPh (1.5 mL). Then MBH carbonates **2b** (29.6 mg, 0.10 mmol) was slowly added via syringe at 0 °C under inert atmosphere. The reaction mixture was stirred for 10 h, and TLC show that the reaction was completed. Then H_2O_2 (1 drop, 30%) was added to the mixture. The mixture was directly purified by column chromatography on silica gel (petroleum ether/ethyl acetate = 1:1) to afford TPOs (yield was based on the amount of **2b**).

General procedure D:

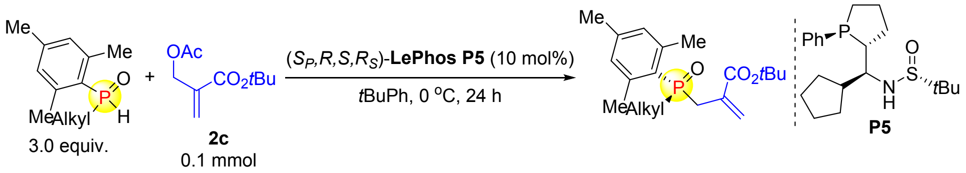

To a flame-dried glass tube with a magnetic stirring bar were added SPO (0.30 mmol) and (S_P, R, S, R_S)-**P5** (3.6 mg, 0.01 mmol), followed by the addition of *t*BuPh (1.5 mL). Then MBH carbonates **2c** (29.6 mg, 0.10 mmol) was slowly added via syringe at 0 °C under inert atmosphere. The reaction mixture was stirred for 24 h, and TLC show that the reaction was completed. Then H_2O_2 (1 drop, 30%) was added to the mixture. The mixture was directly purified by column chromatography on silica gel (petroleum ether/ethyl acetate = 1:1) to afford TPOs (yield was based on the amount of **2c**).

4.1 (*R*)-mesityl(phenyl)phosphine oxide ((*R*)-1a)

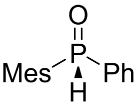

(*R*)-**1a**

(*R*)-**1a** (20.0 mg, 40%) was prepared following general procedure A. $[\alpha]^{20}_{D}$ = 0.22 (*c* 0.5, $CHCl_3$); Enantiomeric excess: 89%, determined by HPLC (Chiralpak IC, hexane/*i*-PrOH = 70/30; flow rate 0.8 ml/min; 25 °C; 210 nm), first peak: t_R = 27.9 min, second peak: t_R = 30.8 min. HRMS (ESI) calcd. For $C_{15}H_{17}NaOP$ $[M+Na]^+$: 267.0909, found: 267.0917.

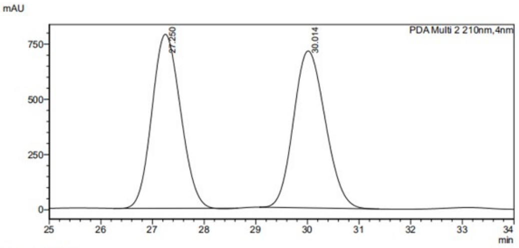

<Peak Table>
PDA Ch2 210nm

Peak#	Ret. Time	Area	Area%	Height	Height%
1	27.250	30427086	49.930	788462	52.583
2	30.014	30512222	50.070	710999	47.417
Total		60939308	100.000	1499461	100.000

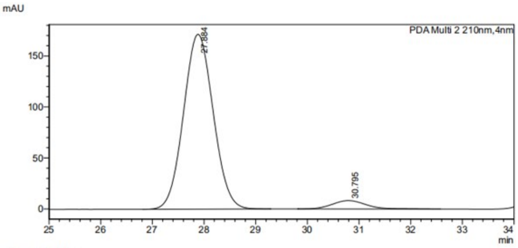

<Peak Table>
PDA Ch2 210nm

Peak#	Ret. Time	Area	Area%	Height	Height%
1	27.884	6847877	94.755	171342	95.418
2	30.795	379015	5.245	8227	4.582
Total		7226892	100.000	179569	100.000

4.2 benzyl (*S*)-2-((mesityl(phenyl)phosphoryl)methyl)acrylate (3ab).

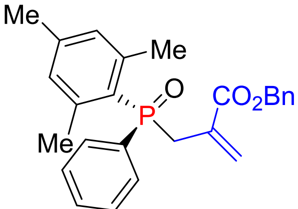

3ab

The general procedure A was followed using **1a** (0.205 mmol) and **2b** (0.1 mmol). After purification by column chromatography (PE/EtOAc = 1:1), **3ab** (35.0 mg, 41%) was obtained. ^1H NMR (400 MHz, $CDCl_3$) δ 7.62 – 7.56 (m, 2H), 7.48 – 7.43 (m, 1H), 7.41 – 7.35 (m, 2H), 7.35 – 7.29 (m, 3H), 7.25 – 7.21 (m, 2H), 6.83 (d, *J* = 3.3 Hz, 2H), 6.41 (d, *J* = 4.7 Hz, 1H), 5.99 (d, *J* = 4.4 Hz, 1H), 4.95 (q, *J* = 12.4 Hz, 2H), 3.61 (s, 1H), 3.57 (d, *J* = 4.2 Hz, 1H), 2.36 (s, 6H), 2.27 (s, 3H). ^{31}P NMR (162 MHz, $CDCl_3$) δ 33.69. ^{13}C NMR (101 MHz, $CDCl_3$) δ 166.13 (d, *J* = 4.0 Hz), 143.33 (d, *J* = 10.4 Hz), 141.56 (d, *J* = 2.6 Hz), 136.33 (d, *J* = 98.2 Hz), 135.73, 131.24 (d, *J* = 2.8 Hz), 131.13, 131.02 (d, *J* = 11.4 Hz), 129.93 (d, *J* = 7.5 Hz), 129.90 (d, *J* = 9.8 Hz), 128.57 (d, *J* = 11.8 Hz), 128.41, 128.07, 127.92, 124.06 (d, *J* = 97.4 Hz), 66.67, 33.72,

33.06, 23.51 (d, J = 2.9 Hz), 20.96. $[\alpha]^{22}_{D}$ = 0.23 (c 0.5, $CHCl_3$); Enantiomeric excess: 90%, determined by HPLC (Chiralpak IC, hexane/i-PrOH = 70/30; flow rate 0.8 ml/min; 25 °C; 210 nm), first peak: t_R = 43.7 min, second peak: t_R = 50.7 min. HRMS (ESI) calcd. For $C_{26}H_{27}NaO_3P$ $[M+Na]^+$: 441.1590, found: 441.1599.

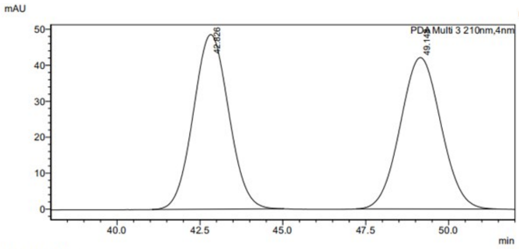

<Peak Table>
PDA Ch3 210nm

Peak#	Ret. Time	Area	Area%	Height	Height%
1	42.826	3613998	50.073	48524	53.566
2	49.148	3603523	49.927	42063	46.434
Total		7217521	100.000	90587	100.000

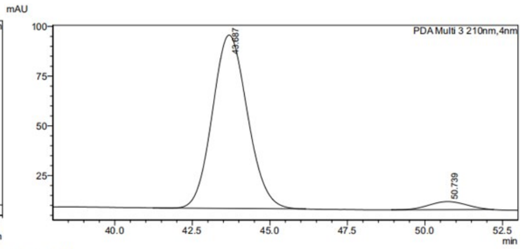

<Peak Table>
PDA Ch3 210nm

Peak#	Ret. Time	Area	Area%	Height	Height%
1	43.687	6875114	95.240	87192	95.553
2	50.739	343645	4.760	4058	4.447
Total		7218759	100.000	91250	100.000

4.3 (*R*)-(3-isopropylphenyl)(mesityl)phosphine oxide ((*R*)-1b).

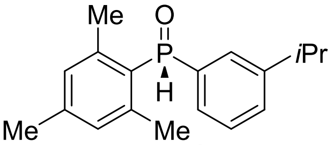

(*R*)-**1b**

(*R*)-**1b** (22.9 mg, 39%) was prepared as a white solid following general procedure A. $[\alpha]^{20}_{D}$ = 0.12 (c 0.5, $CHCl_3$); Enantiomeric excess: 93%, determined by HPLC (Chiralpak IF, hexane/i-PrOH = 70/30; flow rate 0.8 ml/min; 25 °C; 254 nm), first peak: t_R = 9.8 min, second peak: t_R = 12.8 min. HRMS (ESI) calcd. For $C_{18}H_{23}NaOP$ $[M+Na]^+$: 309.1379, found: 309.1371.

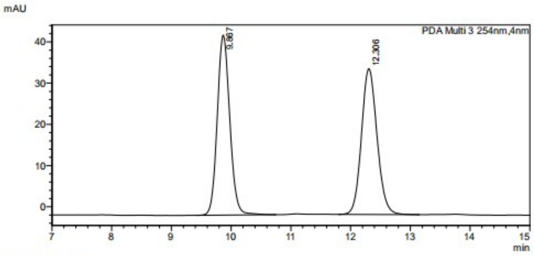

<Peak Table>
PDA Ch3 254nm

Peak#	Ret. Time	Area	Area%	Height	Height%
1	9.867	636748	49.811	43696	55.265
2	12.306	641573	50.189	35371	44.735
Total		1278321	100.000	79067	100.000

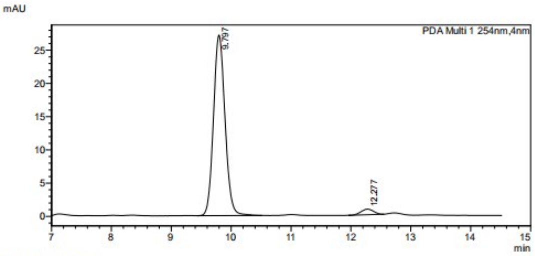

<Peak Table>
PDA Ch1 254nm

Peak#	Ret. Time	Area	Area%	Height	Height%
1	9.797	363574	96.712	27167	97.003
2	12.277	12359	3.288	839	2.997
Total		375934	100.000	28007	100.000

4.4 benzyl (*S*)-2-(((3-isopropylphenyl)(mesityl)phosphoryl)methyl)acrylate (3bb).

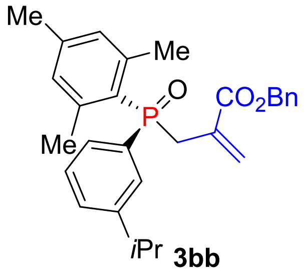

3bb

The general procedure A was followed using **1b** (0.205 mmol) and **2b** (0.1 mmol). After purification by column chromatography (PE/EtOAc = 1:1), **3bb** (37.8 mg, 40%) was obtained. ^{1}H NMR (400 MHz, $CDCl_3$) δ 7.48 (d, J = 12.4 Hz, 1H), 7.38 – 7.29 (m, 6H), 7.25 – 7.21 (m, 2H), 6.83 (d, J = 3.5 Hz, 2H), 6.40 (d, J = 4.7 Hz, 1H), 6.00 (d, J = 3.8 Hz, 1H), 4.94 (q, J = 12.5 Hz, 2H), 3.60 (d, J = 4.0 Hz, 1H), 3.56 (d, J = 7.0 Hz, 1H), 2.97 – 2.81 (m, 1H), 2.35 (s, 6H), 2.28 (s, 3H), 1.21 (d, J = 6.9 Hz, 6H). ^{31}P NMR (162 MHz, $CDCl_3$) δ 34.07. ^{13}C NMR (101 MHz, $CDCl_3$) δ 166.21 (d, J = 4.1 Hz), 149.28 (d, J = 11.1 Hz), 143.41 (d, J = 10.2 Hz), 141.47 (d, J = 2.8 Hz), 135.76, 131.00 (d, J = 11.3 Hz), 129.32 (d, J = 2.7 Hz), 128.65 (d, J = 12.5 Hz), 128.44, 128.22 (d, J = 9.5 Hz), 128.09, 127.93, 127.34 (d, J = 10.4 Hz), 66.68, 34.11, 31.49, 30.11, 23.82 (d, J = 8.8 Hz), 23.55 (d, J = 3.6 Hz), 21.02 (d, J = 1.1 Hz). $[\alpha]^{22}_{D}$ = 0.10 (c 0.5, $CHCl_3$); Enantiomeric excess: 91%, determined by HPLC (Chiralpak IF, hexane/i-PrOH = 70/30; flow rate 0.8 ml/min; 25 °C; 210 nm), first peak: t_R = 10.2 min, second peak: t_R = 15.2 min. HRMS (ESI) calcd. For $C_{29}H_{33}NaO_3P$ [M+Na]$^+$: 483.5511, found: 483.5510.

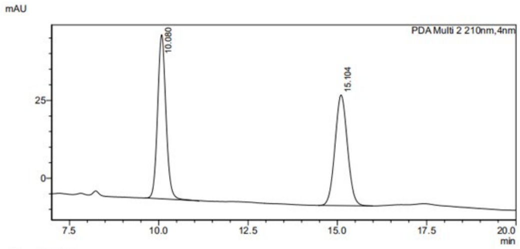

<Peak Table>

PDA Ch2 210nm

Peak#	Ret. Time	Area	Area%	Height	Height%
1	10.080	859595	49.984	52690	59.731
2	15.104	860132	50.016	35522	40.269
Total		1719727	100.000	88213	100.000

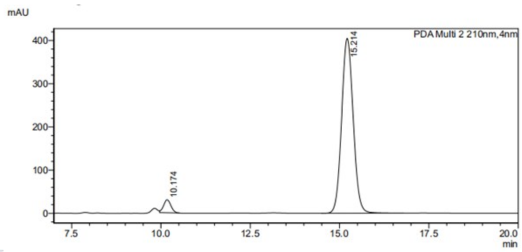

Peak#	Ret. Time	Area	Area%	Height	Height%
1	10.174	429564	4.498	29957	6.893
2	15.214	9120961	95.502	404677	93.107
Total		9550525	100.000	434634	100.000

4.5 (*R*)-mesityl(*m*-tolyl)phosphine oxide ((*R*)-1c).

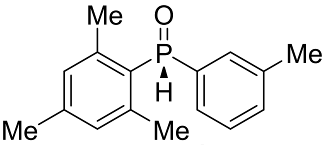

(*R*)-**1c**

(*R*)-**1c** (20.1 mg, 38%) was prepared as a white solid following general procedure A. $[\alpha]^{20}_{D}$ = 0.86 (*c* 0.5, $CHCl_3$); Enantiomeric excess: 98%, determined by HPLC (Chiralpak IC, hexane/*i*-PrOH = 70/30; flow rate 0.8 ml/min; 25 °C; 210 nm), first peak: t_R = 25.7 min, second peak: t_R = 27.6 min. HRMS (ESI) calcd. For $C_{16}H_{19}NaOP$ $[M+Na]^+$: 281.1066, found: 281.1071.

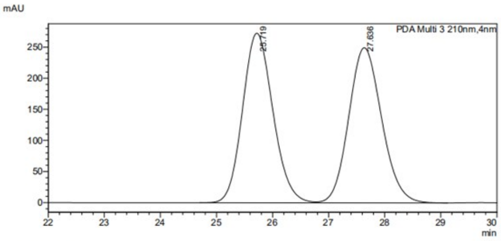

<Peak Table>

PDA Ch3 210nm

Peak#	Ret. Time	Area	Area%	Height	Height%
1	25.719	10264676	50.170	272658	52.222
2	27.636	10195086	49.830	249458	47.778
Total		20459762	100.000	522115	100.000

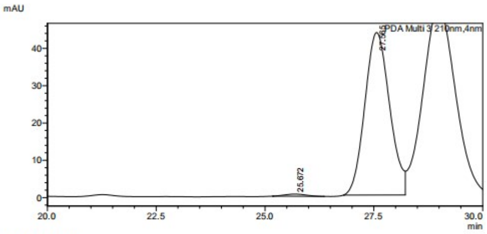

<Peak Table>

PDA Ch3 210nm

Peak#	Ret. Time	Area	Area%	Height	Height%
1	25.672	19844	1.136	573	1.299
2	27.565	1726804	98.864	43552	98.701
Total		1746648	100.000	44126	100.000

4.6 benzyl (*S*)-2-((mesityl(m-tolyl)phosphoryl)methyl)acrylate (3cb).

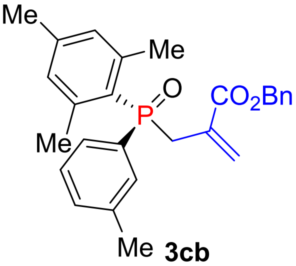

3cb

The general procedure A was followed using **1c** (0.21 mmol) and **2b** (0.1 mmol). After purification by column chromatography (PE/EtOAc = 1:1), **3cb** (35.4 mg, 39%) was obtained. ^1H NMR (400 MHz, $CDCl_3$) δ 7.42 (d, *J* = 12.3 Hz, 1H), 7.39 – 7.29 (m, 5H), 7.28 – 7.25 (m, 1H), 7.24 – 7.21 (m, 2H), 6.83 (d, *J* = 3.5 Hz, 2H), 6.40 (d, *J* = 4.4 Hz, 1H), 5.99 (d, *J* = 4.0 Hz, 1H), 5.02 – 4.77 (m, 2H), 3.70 – 3.45 (m, 2H), 2.36 (s, 6H), 2.32 (s, 3H), 2.27 (s, 3H). ^{31}P NMR (162 MHz, $CDCl_3$) δ 33.83. ^{13}C NMR (101 MHz, $CDCl_3$) δ 166.14 (d, *J* = 4.1 Hz), 143.35 (d, *J* = 10.3 Hz), 141.45 (d, *J* = 2.7 Hz), 138.41 (d, *J* = 11.7 Hz), 136.64, 135.68 (d, *J* = 2.8 Hz), 132.59 (d, *J* = 2.8 Hz), 132.42 (d, *J* = 9.5 Hz), 132.05 (d, *J* = 2.7 Hz), 131.15 (d, *J* = 8.2 Hz), 130.97 (d,

J = 11.4 Hz), 130.33 (d, J = 9.5 Hz), 129.88 (d, J = 8.0 Hz), 129.10 (d, J = 10.3 Hz), 128.53, 128.39, 128.17 (d, J = 13.0 Hz), 128.05, 127.88, 126.80 (d, J = 10.0 Hz), 123.99 (d, J = 97.1 Hz), 66.63, 33.16 (d, J = 65.6 Hz), 23.52 (d, J = 3.7 Hz), 21.39, 20.98 (d, J = 1.1 Hz). $[\alpha]^{22}_{D}$ = 0.34 (*c* 0.5, $CHCl_3$); Enantiomeric excess: 87%, determined by HPLC (Chiralpak IF, hexane/*i*-PrOH = 70/30; flow rate 0.8 ml/min; 25 °C; 210 nm), first peak: t_R = 30.9 min, second peak: t_R = 38.9 min. HRMS (ESI) calcd. For $C_{27}H_{29}NaO_3P$ $[M+Na]^+$: 455.3201, found: 455.3200.

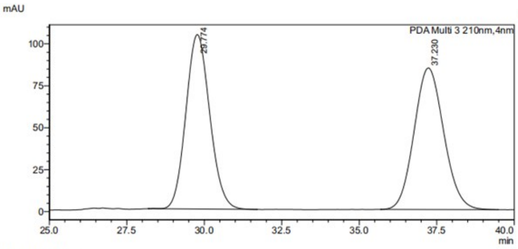

<Peak Table>

PDA Ch3 210nm

Peak#	Ret. Time	Area	Area%	Height	Height%
1	29.774	5582542	49.846	103960	55.215
2	37.230	5616942	50.154	84323	44.785
Total		11199483	100.000	188283	100.000

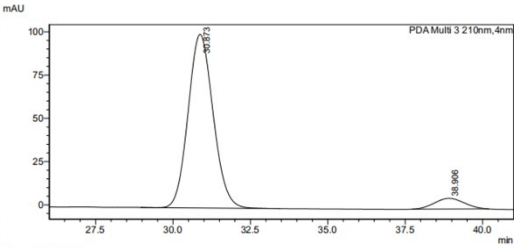

<Peak Table>

PDA Ch3 210nm

Peak#	Ret. Time	Area	Area%	Height	Height%
1	30.873	5635234	93.172	100222	94.202
2	38.906	412998	6.828	6169	5.798
Total		6048231	100.000	106391	100.000

4.7 (*R*)-mesityl(4-vinylphenyl)phosphine oxide ((*R*)-1d).

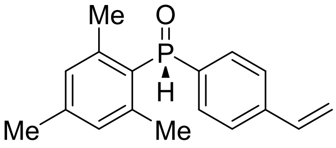

(*R*)-**1d**

(*R*)-**1d** (19.0 mg, 32%) was prepared as a white solid following general procedure A. $[\alpha]^{20}_{D}$ = 0.36 (*c* 0.5, $CHCl_3$); Enantiomeric excess: 90%, determined by HPLC (Chiralpak IF, hexane/*i*-PrOH = 70/30; flow rate 0.8 ml/min; 25 °C; 210 nm), first peak: t_R = 14.0 min, second peak: t_R = 15.0 min. HRMS (ESI) calcd. For $C_{17}H_{19}NaOP$ $[M+Na]^+$: 293.1066, found: 293.1069.

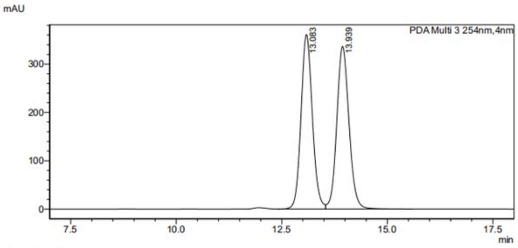

<Peak Table>

PDA Ch3 254nm

Peak#	Ret. Time	Area	Area%	Height	Height%
1	13.083	6740506	49.931	360902	51.750
2	13.939	6759043	50.069	336491	48.250
Total		13499549	100.000	697394	100.000

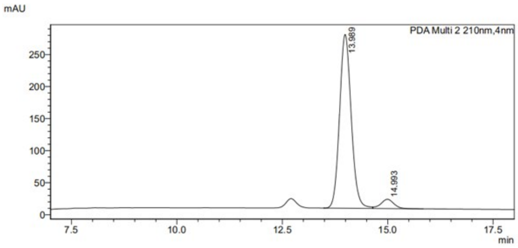

Peak#	Ret. Time	Area	Area%	Height	Height%
1	13.989	5219237	94.362	271227	94.907
2	14.993	311849	5.638	14555	5.093
Total		5531087	100.000	285782	100.000

4.8 benzyl (*S*)-2-((mesityl(4-vinylphenyl)phosphoryl)methyl)acrylate (3db).

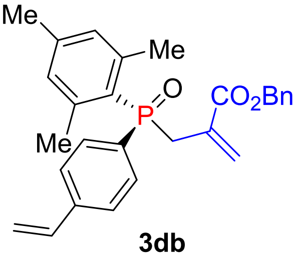

3db

The general procedure A was followed using **1d** (0.22 mmol) and **2b** (0.1 mmol). After purification by column chromatography (PE/EtOAc = 1:1), **3db** (30.1 mg, 33%) was obtained. ^{1}H NMR (500 MHz, $CDCl_3$) δ 8.07 (d, *J* = 7.5 Hz, 1H), 7.58 – 7.53 (m, 2H), 7.44 – 7.38 (m, 2H), 7.34 – 7.27 (m, 2H), 7.22 (dd, *J* = 7.3, 1.9 Hz, 2H), 6.84 (d, *J* = 3.5 Hz, 2H), 6.70 (dd, *J* = 17.6, 10.9 Hz, 1H), 6.43 (d, *J* = 4.8 Hz, 1H), 6.05 (d, *J* = 4.5 Hz, 1H), 5.81 (d, *J* = 17.6 Hz, 1H), 5.34 (d, *J* = 10.9 Hz, 1H), 4.93 (q, *J* = 12.5 Hz, 2H), 3.64 (d, *J* = 13.2 Hz, 2H), 2.37 (s, 6H), 2.27 (s, 3H). ^{31}P NMR (202 MHz, $CDCl_3$) δ 34.45 (d, *J* = 10.9 Hz). ^{13}C NMR (126 MHz, $CDCl_3$) δ 166.12 (d, *J* = 4.0 Hz), 143.40 (d, *J* = 10.5 Hz), 141.74 (d, *J* = 2.5 Hz), 140.42 (d, *J* = 2.7 Hz), 135.93, 135.69, 131.08 (d, *J* = 11.5 Hz), 130.31 (d, *J* = 10.1 Hz), 129.96, 128.42, 128.23 (d, *J* = 0.8 Hz), 128.09, 127.93, 126.34 (d, *J* = 12.2 Hz), 116.17, 66.70, 33.27 (d, *J* = 65.9 Hz), 30.30, 23.60 (d, *J* = 3.7 Hz), 21.01 (d, *J* = 0.9 Hz). $[\alpha]^{22}_D$ = 0.06 (*c* 0.5, $CHCl_3$); Enantiomeric excess: 89%, determined by HPLC (Chiralpak IF, hexane/*i*-PrOH = 70/30; flow rate 0.8 ml/min; 25 °C; 254 nm), first peak: t_R = 22.7 min, second peak: t_R = 26.7 min. HRMS (ESI) calcd. For $C_{28}H_{29}NaO_3P$ $[M+Na]^+$: 467.1254, found: 467.1254.

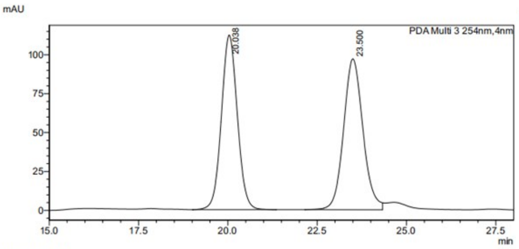

<Peak Table>
PDA Ch3 254nm

Peak#	Ret. Time	Area	Area%	Height	Height%
1	20.038	3480997	48.775	112165	53.644
2	23.500	3655862	51.225	96925	46.356
Total		7136860	100.000	209090	100.000

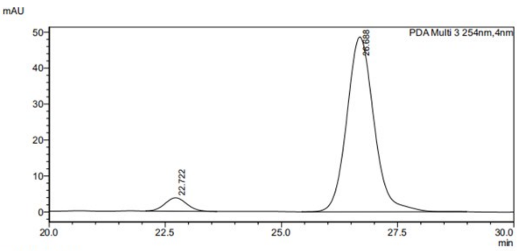

<Peak Table>
PDA Ch3 254nm

Peak#	Ret. Time	Area	Area%	Height	Height%
1	22.722	120989	5.773	3711	7.093
2	26.688	1974759	94.227	48600	92.907
Total		2095748	100.000	52310	100.000

4.9 (*R*)-mesityl(*p*-tolyl)phosphine oxide ((*R*)-1e).

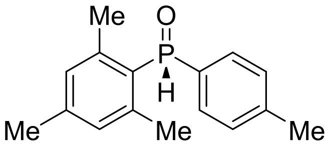

(R)-**1e**

(R)-**1e** (18.0 mg, 34%) was prepared as a white solid following general procedure A. $[\alpha]^{20}_{D}$ = 0.60 (c 0.5, $CHCl_3$); Enantiomeric excess: 84%, determined by HPLC (Chiralpak IF, hexane/i-PrOH = 70/30; flow rate 0.8 ml/min; 25 °C; 210 nm), first peak: t_R = 13.3 min, second peak: t_R = 14.5 min. HRMS (ESI) calcd. For $C_{16}H_{19}NaOP$ $[M+Na]^+$: 281.2102, found: 281.2106.

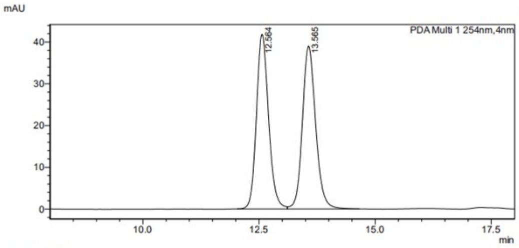

<Peak Table>
PDA Ch1 254nm

Peak#	Ret. Time	Area	Area%	Height	Height%
1	12.564	759577	49.533	41826	51.734
2	13.565	773910	50.467	39022	48.266
Total		1533487	100.000	80848	100.000

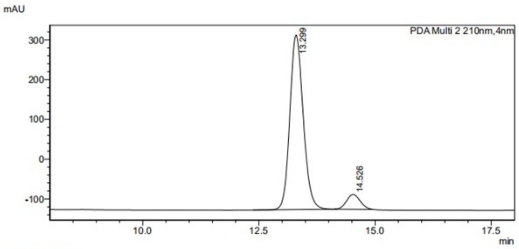

<Peak Table>
PDA Ch2 210nm

Peak#	Ret. Time	Area	Area%	Height	Height%
1	13.299	8804752	92.061	438955	92.169
2	14.526	759244	7.939	37296	7.831
Total		9563996	100.000	476251	100.000

4.10 benzyl (S)-2-((mesityl(p-tolyl)phosphoryl)methyl)acrylate (3eb).

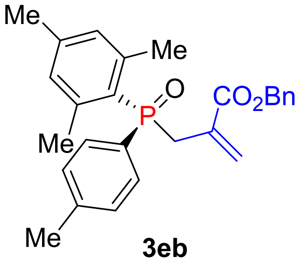

3eb

The general procedure A was followed using **1e** (0.205 mmol) and **2b** (0.1 mmol). After purification by column chromatography (PE/EtOAc = 1:1), **3eb** (35.1 mg, 39%) was obtained. ^{1}H NMR (400 MHz, $CDCl_3$) δ 7.47 (dd, J = 11.8, 8.1 Hz, 2H), 7.36 – 7.30 (m, 3H), 7.25 – 7.21 (m, 2H), 7.19 (dd, J = 8.0, 2.3 Hz, 2H), 6.83 (d, J = 3.4 Hz, 2H), 6.40 (d, J = 4.7 Hz, 1H), 6.00 – 5.97 (m, 1H), 5.02 – 4.86 (m, 2H), 3.65 – 3.49 (m, 2H), 2.36 (s, 3H), 2.35 (s, 6H), 2.27 (s, 3H). ^{31}P NMR (162 MHz, $CDCl_3$) δ 33.67. ^{13}C NMR (101 MHz, $CDCl_3$) δ 166.20 (d, J = 4.0 Hz), 143.35 (d, J = 10.3 Hz), 141.64 (d, J = 2.7 Hz), 141.45 (d, J = 2.7 Hz), 135.74, 133.03 (d, J = 100.6 Hz), 131.22 (d, J = 8.3 Hz), 130.99 (d, J = 11.4 Hz), 129.95, 129.94 (d, J = 10.2 Hz),

129.30 (d, J = 12.3 Hz), 128.42, 128.07, 127.91, 66.65, 33.35 (d, J = 65.8 Hz), 23.55 (d, J = 3.6 Hz), 21.53 (d, J = 1.0 Hz), 20.99 (d, J = 1.3 Hz). $[\alpha]^{22}_{D}$ = 0.12 (*c* 0.5, $CHCl_3$); Enantiomeric excess: 92%, determined by HPLC (Chiralpak IF, hexane/*i*-PrOH = 70/30; flow rate 0.8 ml/min; 25 °C; 210 nm), first peak: t_R = 21.6 min, second peak: t_R = 23.9 min. HRMS (ESI) calcd. For $C_{27}H_{29}NaO_3P$ $[M+Na]^+$: 455.3122, found: 455.3120.

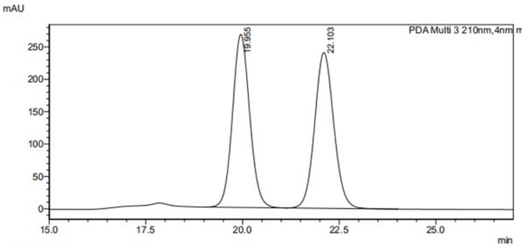

<Peak Table>
PDA Ch3 210nm

Peak#	Ret. Time	Area	Area%	Height	Height%
1	19.955	8286994	49.934	267337	52.612
2	22.103	8308759	50.066	240797	47.388
Total		16595753	100.000	508134	100.000

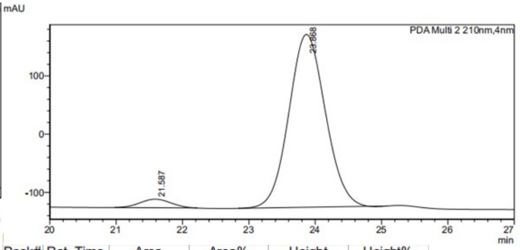

Peak#	Ret. Time	Area	Area%	Height	Height%
1	21.587	477386	4.028	14662	4.707
2	23.868	11374165	95.972	296841	95.293
Total		11851551	100.000	311502	100.000

4.11 (*R*)-[1,1'-biphenyl]-4-yl(mesityl)phosphine oxide ((*R*)-3f).

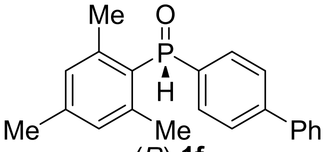

(*R*)-**1f**

(*R*)-**1f** (23.6 mg, 36%) was prepared as a white solid following general procedure A. $[\alpha]^{20}_{D}$ = 0.84 (*c* 0.5, $CHCl_3$); Enantiomeric excess: 84%, determined by HPLC (Chiralpak IC, hexane/*i*-PrOH = 70/30; flow rate 0.8 ml/min; 25 °C; 190 nm), first peak: t_R = 46.9 min, second peak: t_R = 52.8 min. HRMS (ESI) calcd. For $C_{21}H_{22}OP$ $[M+H]^+$: 321.1403, found: 321.1400.

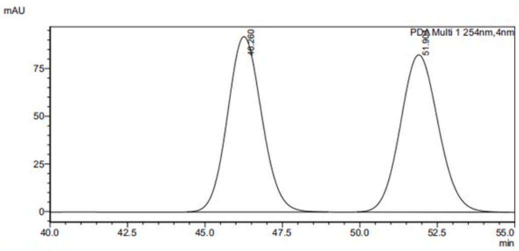

<Peak Table>
PDA Ch1 254nm

Peak#	Ret. Time	Area	Area%	Height	Height%
1	46.260	7094813	50.081	91927	52.725
2	51.901	7071879	49.919	82426	47.275
Total		14166691	100.000	174354	100.000

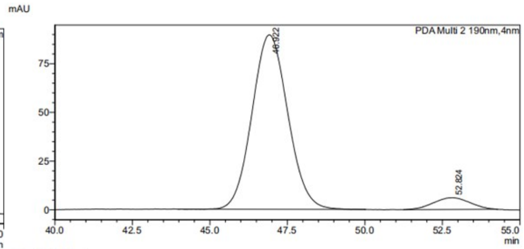

<Peak Table>
PDA Ch2 190nm

Peak#	Ret. Time	Area	Area%	Height	Height%
1	46.922	7223081	93.618	89584	93.694
2	52.824	492397	6.382	6030	6.306
Total		7715479	100.000	95614	100.000

4.12 benzyl (*S*)-2-(([1,1'-biphenyl]-4-yl(mesityl)phosphoryl)methyl)acrylate (3fb).

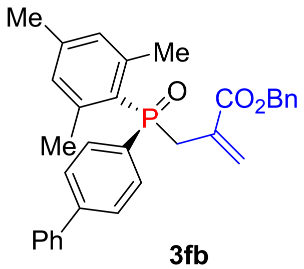

3fb

The general procedure A was followed using **1f** (0.205 mmol) and **2b** (0.1 mmol). After purification by column chromatography (PE/EtOAc = 1:1), **3fb** (40.4 mg, 40%) was obtained. ^{1}H NMR (500 MHz, $CDCl_3$) δ 7.66 (dd, *J* = 11.3, 8.3 Hz, 2H), 7.61 (dd, *J* = 8.3, 2.7 Hz, 2H), 7.59 – 7.56 (m, 2H), 7.45 (t, *J* = 7.6 Hz, 2H), 7.39 (d, *J* = 7.2 Hz, 1H), 7.34 – 7.29 (m, 3H), 7.25 – 7.22 (m, 2H), 6.86 (d, *J* = 3.4 Hz, 2H), 6.43 (d, *J* = 4.4 Hz, 1H), 6.02 (d, *J* = 4.1 Hz, 1H), 4.96 (q, *J* = 12.5 Hz, 2H), 3.62 (d, *J* = 13.1 Hz, 2H), 2.40 (s, 6H), 2.29 (s, 3H). ^{31}P NMR (162 MHz, $CDCl_3$) δ 33.50. ^{13}C NMR (126 MHz, $CDCl_3$) δ 166.19 (d, *J* = 4.0 Hz), 143.99 (d, *J* = 2.7 Hz), 143.38 (d, *J* = 10.4 Hz), 141.63 (d, *J* = 2.6 Hz), 139.94, 135.72, 134.93 (d, *J* = 99.1 Hz), 131.18 (d, *J* = 8.4 Hz), 131.08 (d, *J* = 11.4 Hz), 130.47 (d, *J* = 10.0 Hz), 130.05 (d, *J* = 8.0 Hz), 128.90, 128.44, 128.10, 127.95, 127.65 (d, *J* = 92.7 Hz), 127.18, 124.08 (d, *J* = 97.5 Hz), 66.72, 33.53 (d, *J* = 65.9 Hz), 23.62 (d, *J* = 3.6 Hz), 21.02 (d, *J* = 0.8 Hz). $[\alpha]^{22}_{D}$ = 0.16 (*c* 0.5, $CHCl_3$); Enantiomeric excess: 94%, determined by HPLC (Chiralpak IF, hexane/*i*-PrOH = 70/30; flow rate 0.8 ml/min; 25 °C; 210 nm), first peak: t_R = 26.1 min, second peak: t_R = 28.0 min. HRMS (ESI) calcd. For $C_{32}H_{31}NaO_3P$ [M+Na]$^+$: 517.5501, found: 517.5506.

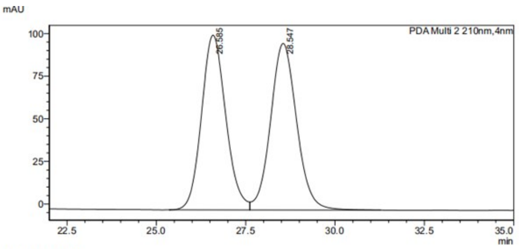

<Peak Table>
PDA Ch2 210nm

Peak#	Ret. Time	Area	Area%	Height	Height%
1	26.585	4854122	49.395	102469	51.198
2	28.547	4973105	50.605	97673	48.802
Total		9827227	100.000	200143	100.000

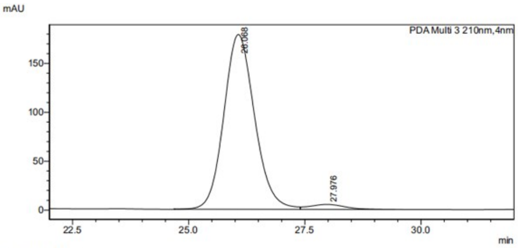

<Peak Table>
PDA Ch3 210nm

Peak#	Ret. Time	Area	Area%	Height	Height%
1	26.068	8246680	96.886	178804	97.212
2	27.976	265053	3.114	5127	2.788
Total		8511733	100.000	183932	100.000

4.13 (*R*)-4-(*tert*-butyl)phenyl)(mesityl)phosphine oxide ((*R*)-1g).

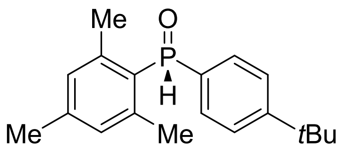

(R)-**1g**

(R)-**14** (16.0 mg, 28%) was prepared as a white solid following general procedure B. $[\alpha]^{20}_{D}$ = 0.52 (c 0.5, $CHCl_3$); Enantiomeric excess: 97%, determined by HPLC (Chiralpak IF, hexane/i-PrOH = 70/30; flow rate 0.8 ml/min; 25 °C; 190 nm), first peak: t_R = 11.3 min, second peak: t_R = 13.2 min. HRMS (ESI) calcd. For $C_{19}H_{25}NaOP$ $[M+Na]^+$: 323.1535, found: 323.1539.

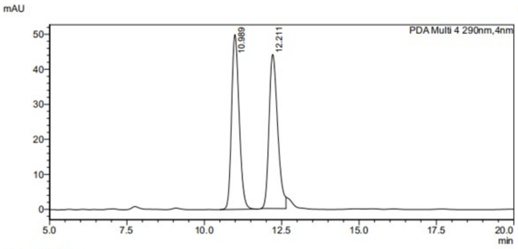

<Peak Table>

PDA Ch4 290nm

Peak#	Ret. Time	Area	Area%	Height	Height%
1	10.989	851416	49.923	49927	53.126
2	12.211	854032	50.077	44051	46.874
Total		1705448	100.000	93978	100.000

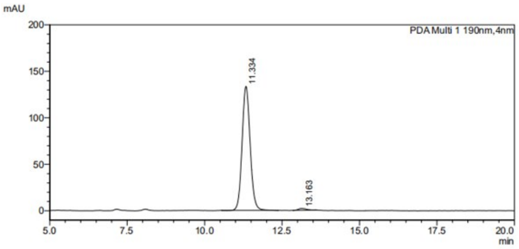

PDA Ch1 190nm

Peak#	Ret. Time	Area	Area%	Height	Height%
1	11.334	2422514	98.542	133366	98.638
2	13.163	35847	1.458	1842	1.362
Total		2458361	100.000	135207	100.000

4.14 benzyl (*S*)-2-(((4-(tert-butyl)phenyl)(mesityl)phosphoryl)methyl)acrylate (3gb).

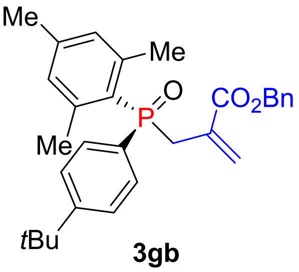

3gb

The general procedure A was followed using **1g** (0.21 mmol) and **2b** (0.1 mmol). After purification by column chromatography (PE/EtOAc = 1:1), **3gb** (29.9 mg, 30%) was obtained. ^1H NMR (400 MHz, $CDCl_3$) δ 7.54 – 7.47 (m, 2H), 7.42 – 7.37 (m, 2H), 7.35 – 7.29 (m, 3H), 7.25 – 7.22 (m, 2H), 6.83 (d, J = 2.8 Hz, 2H), 6.41 (d, J = 4.4 Hz, 1H), 6.00 (d, J = 3.9 Hz, 1H), 4.95 (q, J = 12.5 Hz, 2H), 3.57 (d, J = 12.8 Hz, 2H), 2.37 (s, 6H), 2.27 (s, 3H), 1.29 (s, 9H). ^{31}P NMR (162 MHz, $CDCl_3$) δ 33.51. ^{13}C NMR (101 MHz, $CDCl_3$) δ 166.20 (d, J = 3.9 Hz), 154.69 (d, J = 2.5 Hz), 143.36 (d, J = 10.3 Hz), 141.41 (d, J = 2.6 Hz), 135.72, 132.99 (d, J = 100.3 Hz), 131.27 (d, J =

8.3 Hz), 130.97 (d, J = 11.4 Hz), 129.91 (d, J = 8.1 Hz), 129.75 (d, J = 10.1 Hz), 128.41, 128.07, 127.91, 125.56 (d, J = 12.1 Hz), 66.64, 34.87, 33.36 (d, J = 65.9 Hz), 31.06, 23.56, 20.99. $[\alpha]^{22}_D$ = 0.11 (c 0.5, $CHCl_3$); Enantiomeric excess: 94%, determined by HPLC (Chiralpak IF, hexane/i-PrOH = 70/30; flow rate 0.8 ml/min; 25 °C; 254 nm), first peak: t_R = 14.2 min, second peak: t_R = 15.2 min. HRMS (ESI) calcd. For $C_{30}H_{35}NaO_3P$ $[M+Na]^+$: 497.2216, found: 497.2218.

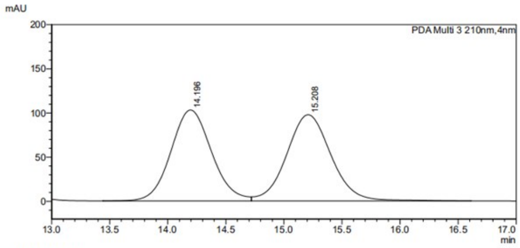

<Peak Table>
PDA Ch3 210nm

Peak#	Ret. Time	Area	Area%	Height	Height%
1	14.196	2508087	49.197	102885	51.338
2	15.208	2589984	50.803	97524	48.662
Total		5098071	100.000	200409	100.000

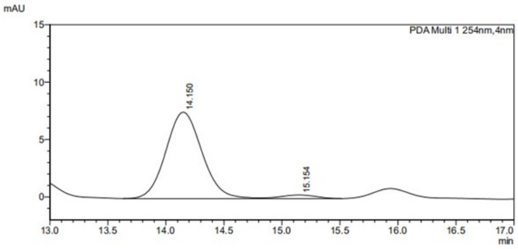

Peak#	Ret. Time	Area	Area%	Height	Height%
1	14.150	162855	96.157	7518	96.039
2	15.154	6509	3.843	310	3.961
Total		169364	100.000	7828	100.000

4.15 (*R*)-(3,5-di-tert-butylphenyl)(mesityl)phosphine oxide ((*R*)-1h).

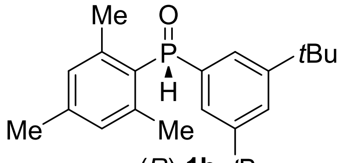

(*R*)-**1h**

(*R*)-**1h** (30.0 mg, 41%) was prepared as a white solid following general procedure A. $[\alpha]^{20}_D$ = 0.31 (c 0.5, $CHCl_3$); Enantiomeric excess: 97%, determined by HPLC (Chiralpak IF, hexane/i-PrOH = 70/30; flow rate 0.8 ml/min; 25 °C; 254 nm), first peak: t_R = 7.2 min, second peak: t_R = 10.1 min. HRMS (ESI) calcd. For $C_{23}H_{33}NaOP$ $[M+Na]^+$: 379.2161, found: 379.2155.

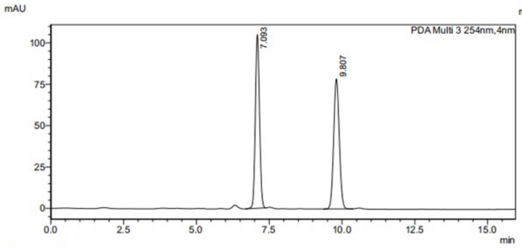

<Peak Table>
PDA Ch3 254nm

Peak#	Ret. Time	Area	Area%	Height	Height%
1	7.093	1050044	49.813	105030	57.218
2	9.807	1057908	50.187	78532	42.782
Total		2107953	100.000	183562	100.000

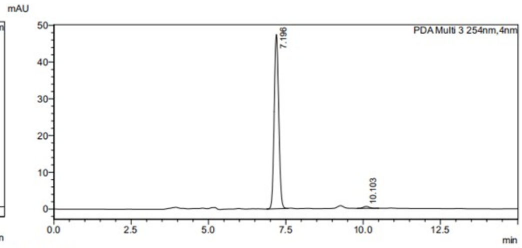

<Peak Table>
PDA Ch3 254nm

Peak#	Ret. Time	Area	Area%	Height	Height%
1	7.196	483646	98.580	47413	98.920
2	10.103	6967	1.420	518	1.080
Total		490613	100.000	47931	100.000

4.16 benzyl (*S*)-2-(((3,5-di-tert-butylphenyl)(mesityl)phosphoryl)methyl)acrylate (3hb).

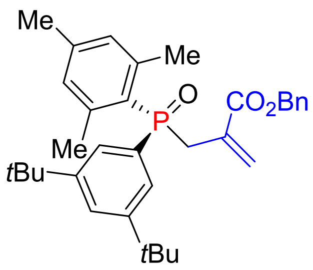

3hb

The general procedure A was followed using **1h** (0.205 mmol) and **2b** (0.1 mmol). After purification by column chromatography (PE/EtOAc = 2:1), **3hb** (44.4 mg, 41%) was obtained. ^{1}H NMR (400 MHz, $CDCl_3$) δ 7.51 (s, 1H), 7.42 (d, *J* = 12.7 Hz, 2H), 7.35 – 7.29 (m, 3H), 7.25 – 7.20 (m, 2H), 6.83 (s, 2H), 6.40 (d, *J* = 3.8 Hz, 1H), 6.02 (d, *J* = 3.0 Hz, 1H), 4.96 (q, *J* = 12.5 Hz, 2H), 3.57 (d, *J* = 13.1 Hz, 2H), 2.35 (s, 6H), 2.27 (s, 3H), 1.27 (s, 18H). ^{31}P NMR (162 MHz, $CDCl_3$) δ 35.09. ^{13}C NMR (101 MHz, $CDCl_3$) δ 166.27 (d, *J* = 3.9 Hz), 151.06 (d, *J* = 11.6 Hz), 143.41 (d, *J* = 10.3 Hz), 141.25, 135.80, 130.92 (d, *J* = 11.2 Hz), 129.61 (d, *J* = 7.1 Hz), 128.41, 128.04, 127.87, 125.35, 124.07 (d, *J* = 10.3 Hz), 66.64, 34.94, 31.26, 23.50, 20.99. $[\alpha]^{22}_{D}$ = 0.08 (*c* 0.5, $CHCl_3$); Enantiomeric excess: 95%, determined by HPLC (Chiralpak IF, hexane/*i*-PrOH = 70/30; flow rate 0.8 ml/min; 25 °C; 190 nm), first peak: t_R = 6.6 min, second peak: t_R = 9.3 min. HRMS (ESI) calcd. For $C_{34}H_{43}NaO_3P$ $[M+Na]^+$: 553.2911, found: 553.2910.

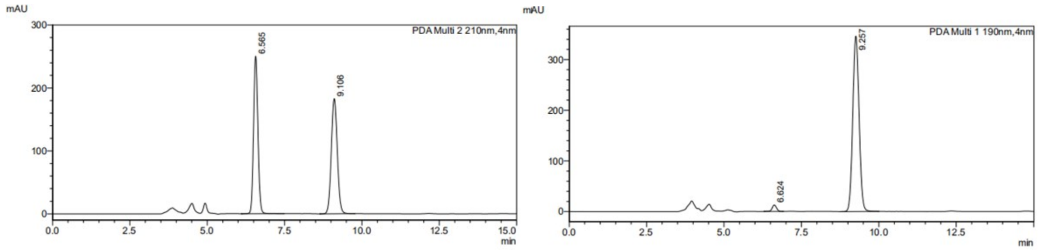

Peak#	Ret. Time	Area%	Area	Height%	Height
1	6.565	49.966	2457420	57.772	250437
2	9.106	50.034	2460766	42.228	183058
Total		100.000	4918187	100.000	433494

PDA Ch1 190nm

Peak#	Ret. Time	Area	Area%	Height	Height%
1	6.624	127761	2.521	12764	3.555
2	9.257	4940947	97.479	346266	96.445
Total		5068708	100.000	359031	100.000

4.17 (*R*)-(3,5-dimethylphenyl)(mesityl)phosphine oxide ((*R*)-1i).

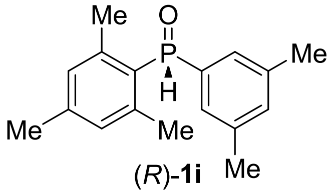

(*R*)-**1i**

(R)-**1i** (18.4 mg, 33%) was prepared as a white solid following general procedure A. $[\alpha]^{20}_{D}$ = 1.34 (c 0.5, $CHCl_3$); Enantiomeric excess: 92%, determined by HPLC (Chiralpak IF, hexane/i-PrOH = 70/30; flow rate 0.8 ml/min; 25 °C; 254 nm), first peak: t_R = 12.7 min, second peak: t_R = 21.7 min. HRMS (ESI) calcd. For $C_{17}H_{21}NaOP$ $[M+Na]^+$: 295.1222, found: 295.1229.

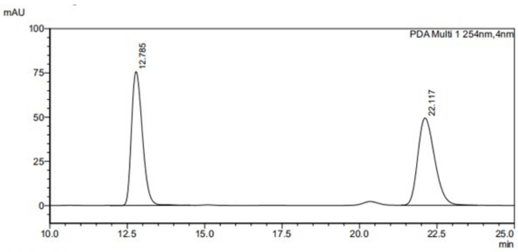

<Peak Table>
PDA Ch1 254nm

Peak#	Ret. Time	Area	Area%	Height	Height%
1	12.785	1849018	49.854	75607	60.528
2	22.117	1859876	50.146	49306	39.472
Total		3708894	100.000	124913	100.000

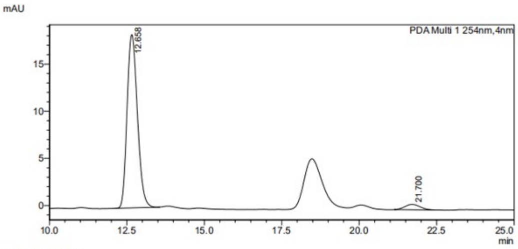

<Peak Table>
PDA Ch1 254nm

Peak#	Ret. Time	Area	Area%	Height	Height%
1	12.658	427103	95.730	18382	97.033
2	21.700	19049	4.270	562	2.967
Total		446152	100.000	18944	100.000

4.18 benzyl (*S*)-2-(((3,5-dimethylphenyl)(mesityl)phosphoryl)methyl)acrylate (3ib).

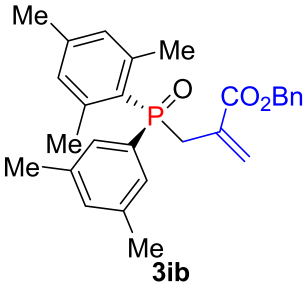

3ib

The general procedure was followed using **1i** (0.22 mmol) and **2b** (0.1 mmol). After purification by column chromatography (PE/EtOAc = 1:1), **3ib** (37.0 mg, 38%) was obtained. ^{1}H NMR (500 MHz, $CDCl_3$) δ 7.36 – 7.30 (m, 3H), 7.23 – 7.20 (m, 3H), 7.18 (s, 1H), 7.09 (s, 1H), 6.83 (d, J = 3.3 Hz, 2H), 6.40 (d, J = 4.6 Hz, 1H), 6.02 (s, 1H), 4.93 (q, J = 12.5 Hz, 2H), 3.66 – 3.52 (m, 2H), 2.35 (s, 6H), 2.28 (s, 6H), 2.27 (s, 3H). ^{31}P NMR (202 MHz, $CDCl_3$) δ 34.81 (d, J = 11.5 Hz). ^{13}C NMR (126 MHz, $CDCl_3$) δ 166.17 (d, J = 3.8 Hz), 143.48 (d, J = 10.4 Hz), 141.51, 138.34 (d, J = 12.5 Hz), 135.73, 133.12, 131.00 (d, J = 11.6 Hz), 130.01 (d, J = 6.1 Hz), 128.41, 128.07, 127.88, 127.38 (d, J = 9.7 Hz), 66.64, 23.55 (d, J = 3.5 Hz), 21.30, 21.01. $[\alpha]^{22}_{D}$ = 0.25 (c 0.5, $CHCl_3$); Enantiomeric excess: 93%, determined by HPLC (Chiralpak ODH, hexane/i-PrOH = 90/10; flow rate 0.6 ml/min; 25 °C; 210 nm), first peak: t_R =

13.9 min, second peak: t_R = 18.5 min. HRMS (ESI) calcd. For $C_{28}H_{31}NaO_3P$ $[M+Na]^+$: 469.1903, found: 469.1896.

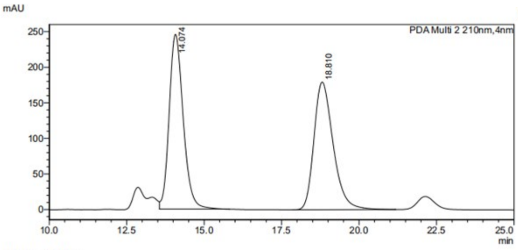

<Peak Table>
PDA Ch2 210nm

Peak#	Ret. Time	Area	Area%	Height	Height%
1	14.074	7757045	50.039	245618	57.787
2	18.810	7744995	49.961	179424	42.213
Total		15502040	100.000	425043	100.000

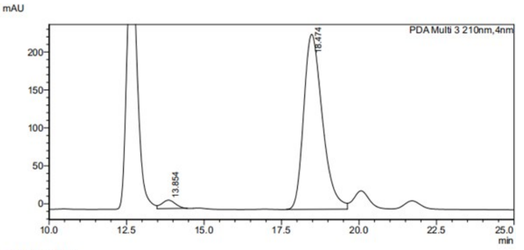

<Peak Table>
PDA Ch3 210nm

Peak#	Ret. Time	Area	Area%	Height	Height%
1	13.854	336334	3.346	10966	4.537
2	18.474	9714792	96.654	230711	95.463
Total		10051126	100.000	241677	100.000

4.19 (*R*)-mesityl(3,4,5-trimethoxyphenyl)phosphine oxide ((*R*)-1j).

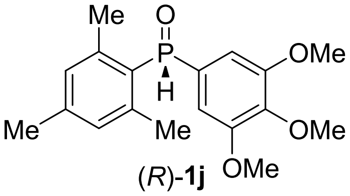

(*R*)-**1j**

(*R*)-**1j** (27.4 mg, 40%) was prepared as a white solid following general procedure A. $[\alpha]^{20}_D$ = 0.06 (*c* 0.5, $CHCl_3$); Enantiomeric excess: 96%, determined by HPLC (Chiralpak IF, hexane/*i*-PrOH = 70/30; flow rate 0.8 ml/min; 25 °C; 210 nm), first peak: t_R = 18.3 min, second peak: t_R = 24.1 min. HRMS (ESI) calcd. For $C_{18}H_{23}NaO_4P$ $[M+Na]^+$: 357.1226, found: 357.1227.

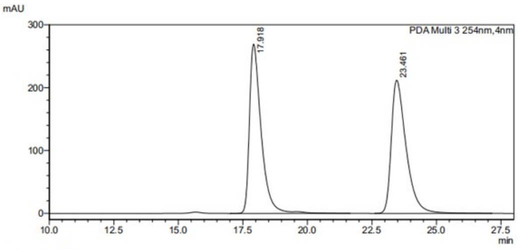

<Peak Table>
PDA Ch3 254nm

Peak#	Ret. Time	Area	Area%	Height	Height%
1	17.918	8543262	50.164	269543	55.969
2	23.461	8487311	49.836	212048	44.031
Total		17030573	100.000	481591	100.000

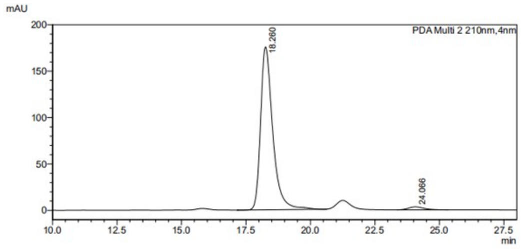

Peak#	Ret. Time	Area	Area%	Height	Height%
1	18.260	5762057	97.813	175650	98.261
2	24.066	128810	2.187	3109	1.739
Total		5890867	100.000	178759	100.000

4.20 benzyl (*S*)-2-((mesityl(3,4,5-trimethoxyphenyl)phosphoryl)methyl)acrylate (3jb).

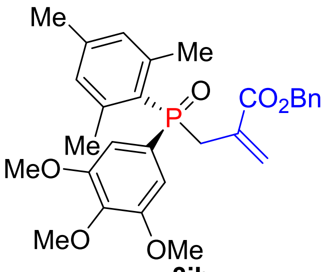

3jb

The general procedure was followed using **1j** (0.21 mmol) and **2b** (0.1 mmol). After purification by column chromatography (PE/EtOAc = 1:1), **3jb** (41.9 mg, 41%) was obtained. ^{1}H NMR (400 MHz, $CDCl_3$) δ 7.36 – 7.29 (m, 3H), 7.25 – 7.19 (m, 2H), 6.85 (d, J = 3.5 Hz, 2H), 6.80 (s, 1H), 6.77 (s, 1H), 6.41 (d, J = 4.8 Hz, 1H), 6.00 (d, J = 4.5 Hz, 1H), 4.99 – 4.91 (m, 2H), 3.85 (s, 3H), 3.76 (s, 6H), 3.64 – 3.45 (m, 2H), 2.39 (s, 6H), 2.27 (s, 3H). ^{31}P NMR (162 MHz, $CDCl_3$) δ 34.31. ^{13}C NMR (101 MHz, $CDCl_3$) δ 166.16 (d, J = 4.0 Hz), 153.35 (d, J = 17.2 Hz), 143.26 (d, J = 10.3 Hz), 141.61 (d, J = 2.7 Hz), 140.61 (d, J = 2.7 Hz), 135.59, 131.29 (d, J = 27.0 Hz), 131.03 (d, J = 11.3 Hz), 130.43, 130.05 (d, J = 8.0 Hz), 128.42, 128.12, 127.89, 124.04 (d, J = 97.9 Hz), 107.14 (d, J = 11.5 Hz), 69.43, 66.75, 60.85 (d, J = 2.4 Hz), 56.22 (d, J = 2.1 Hz), 53.73, 33.74 (d, J = 66.0 Hz), 29.20, 23.58 (d, J = 1.5 Hz), 20.98. $[\alpha]^{22}_D$ = -0.32 (c 0.5, $CHCl_3$); Enantiomeric excess: 92%, determined by HPLC (Chiralpak IF, hexane/i-PrOH = 70/30; flow rate 0.8 ml/min; 25 °C; 190 nm), first peak: t_R = 21.2 min, second peak: t_R = 38.1 min. HRMS (ESI) calcd. For $C_{29}H_{33}NaO_6P$ [M+Na]$^+$: 531.1907, found: 531.1907.

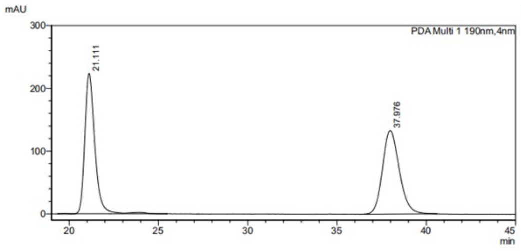

PDA Ch1 190nm

Peak#	Ret. Time	Area	Area%	Height	Height%
1	21.111	8827269	50.297	223805	62.706
2	37.976	8722962	49.703	133106	37.294
Total		17550231	100.000	356911	100.000

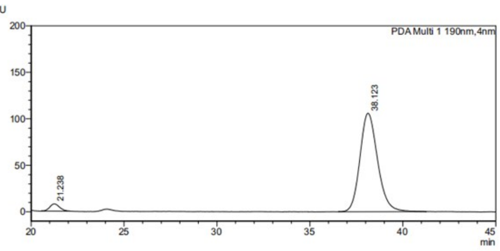

PDA Ch1 190nm

Peak#	Ret. Time	Area	Area%	Height	Height%
1	21.238	279928	3.834	7745	6.814
2	38.123	7021859	96.166	105923	93.186
Total		7301786	100.000	113668	100.000

4.21 (*R*)-(2,6-dimethylphenyl)(phenyl)phosphine oxide ((*R*)-1k).

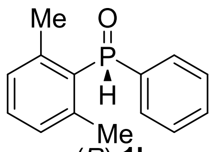

(*R*)-**1k**

(*R*)-**1k** (13.8 mg, 30%) was prepared as a white solid following general procedure B. $[\alpha]^{20}_{D}$ = 0.33 (*c* 0.5, $CHCl_3$); Enantiomeric excess: 93%, determined by HPLC (Chiralpak IF, hexane/*i*-PrOH = 70/30; flow rate 0.8 ml/min; 25 °C; 190 nm), first peak: t_R = 11.6 min, second peak: t_R = 12.2 min. HRMS (ESI) calcd. For $C_{14}H_{15}NaOP$ $[M+Na]^+$: 253.0753, found: 253.0760.

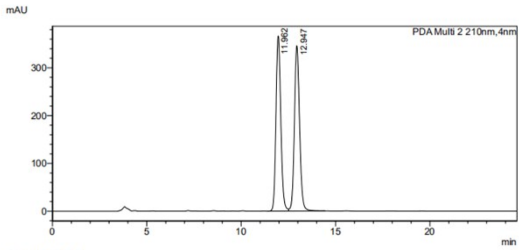

<Peak Table>

PDA Ch2 210nm

Peak#	Ret. Time	Area	Area%	Height	Height%
1	11.962	6492469	49.582	366158	51.441
2	12.947	6602022	50.418	345651	48.559
Total		13094491	100.000	711809	100.000

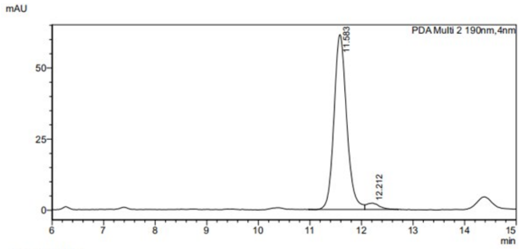

<Peak Table>

PDA Ch2 190nm

Peak#	Ret. Time	Area	Area%	Height	Height%
1	11.583	1051123	96.423	61468	96.510
2	12.212	38988	3.577	2223	3.490
Total		1090111	100.000	63691	100.000

4.22 benzyl (*S*)-2-(((2,6-dimethylphenyl)(phenyl)phosphoryl)methyl)acrylate (3kb).

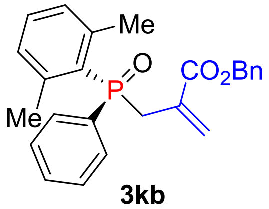

3kb

The general procedure A was followed using **1k** (0.205 mmol) and **2b** (0.1 mmol). After purification by column chromatography (PE/EtOAc = 2:1), **3kb** (31.9 mg, 39%) was obtained. ^{1}H NMR (500 MHz, $CDCl_3$) δ 7.62 – 7.57 (m, 2H), 7.47 (td, *J* = 7.3, 1.3 Hz, 1H), 7.41 – 7.38 (m, 2H), 7.35 – 7.29 (m, 3H), 7.29 – 7.24 (m, 1H), 7.24 – 7.19 (m, 2H), 7.01 (dd, *J* = 7.6, 3.8 Hz, 2H), 6.42 (d, *J* = 4.8 Hz, 1H), 6.00 (d, *J* = 4.2 Hz, 1H), 4.94 (q, *J* = 12.4 Hz, 2H), 3.68 – 3.53 (m, 2H), 2.40 (s, 6H). ^{31}P NMR (162 MHz, $CDCl_3$) δ 33.55. ^{13}C NMR (126 MHz, $CDCl_3$) δ 166.09 (d, *J* = 4.0 Hz), 143.39 (d, *J* = 10.0 Hz), 136.49, 135.69 (d, *J* = 2.6 Hz), 131.45 (d, *J* = 2.6 Hz), 131.35 (d, *J* = 2.7 Hz), 131.04 (d, *J* = 8.4 Hz), 130.16 (d, *J* = 10.9 Hz), 130.06, 129.88 (d, *J* = 9.8

Hz), 128.64 (d, J = 11.8 Hz), 128.44, 128.11, 127.99, 127.37 (d, J = 95.0 Hz), 66.71, 33.30 (d, J = 65.6 Hz), 23.66 (d, J = 3.7 Hz). $[\alpha]^{22}_{D}$ = 0.21 (c 0.5, $CHCl_3$); Enantiomeric excess: 90%, determined by HPLC (Chiralpak IF, hexane/i-PrOH = 80/20; flow rate 0.8 ml/min; 25 °C; 220 nm), first peak: t_R = 26.2 min, second peak: t_R = 37.9 min. HRMS (ESI) calcd. For $C_{25}H_{25}NaO_3P$ $[M+Na]^+$: 427.1433, found: 427.1431.

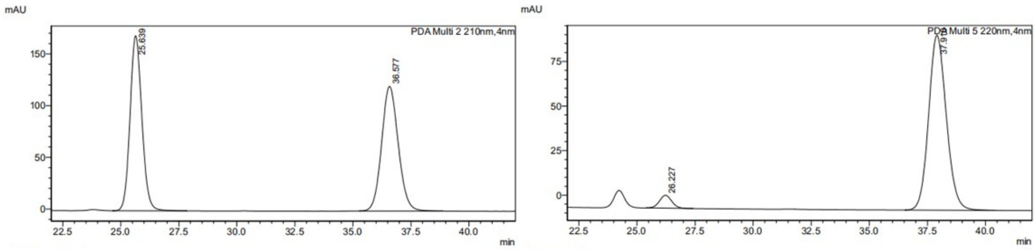

<Peak Table>
PDA Ch2 210nm

Peak#	Ret. Time	Area	Area%	Height	Height%
1	25.639	6013414	50.050	169169	58.415
2	36.577	6001300	49.950	120428	41.585
Total		12014714	100.000	289597	100.000

<Peak Table>
PDA Ch5 220nm

Peak#	Ret. Time	Area	Area%	Height	Height%
1	26.227	267259	4.880	7234	6.866
2	37.910	5209622	95.120	98125	93.134
Total		5476882	100.000	105359	100.000

4.23 (*R*)-(3,5-di-tert-butylphenyl)(2,6-dimethylphenyl)phosphine oxide ((*R*)-1l).

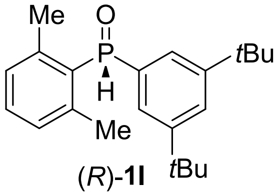

(*R*)-**1l**

(*R*)-**1l** (26.0 mg, 37%) was prepared as a white solid following general procedure A. $[\alpha]^{20}_{D}$ = 0.55 (c 0.5, $CHCl_3$); Enantiomeric excess: 98%, determined by HPLC (Chiralpak IF, hexane/i-PrOH = 80/20; flow rate 0.8 ml/min; 25 °C; 210 nm), first peak: t_R = 8.9 min, second peak: t_R = 11.0 min. HRMS (ESI) calcd. For $C_{22}H_{31}NaOP$ $[M+Na]^+$: 365.2002, found: 365.2005.

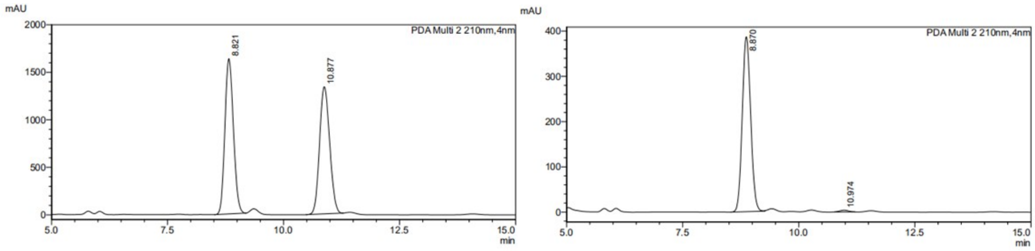

Peak#	Ret. Time	Area	Area%	Height	Height%
1	8.821	20873392	50.698	1635458	54.980
2	10.877	20298635	49.302	1339190	45.020
Total		41172027	100.000	2974648	100.000

Peak#	Ret. Time	Area	Area%	Height	Height%
1	8.870	4862648	98.987	386240	99.038
2	10.974	49742	1.013	3750	0.962
Total		4912390	100.000	389990	100.000

4.24 benzyl (*S*)-2-(((3,5-di-tert-butylphenyl)(2,6-dimethylphenyl)phosphoryl) methyl)acrylate (3lb).

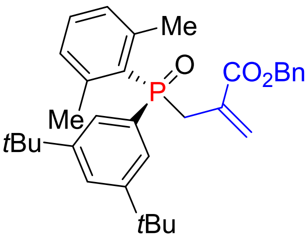

3lb

The general procedure A was followed using **1l** (0.205 mmol) and **2b** (0.1 mmol). After purification by column chromatography (PE/EtOAc = 2:1), **3lb** (40.4 mg, 38%) was obtained. ^{1}H NMR (400 MHz, $CDCl_3$) δ 7.51 (d, *J* = 1.3 Hz, 1H), 7.43 (d, *J* = 1.8 Hz, 1H), 7.39 (d, *J* = 1.8 Hz, 1H), 7.35 – 7.29 (m, 3H), 7.23 (td, *J* = 6.6, 1.5 Hz, 3H), 7.01 (dd, *J* = 7.6, 3.7 Hz, 2H), 6.41 (d, *J* = 4.1 Hz, 1H), 6.03 (d, *J* = 3.8 Hz, 1H), 4.95 (q, *J* = 12.5 Hz, 2H), 3.63 – 3.56 (m, 2H), 2.39 (s, 6H), 1.26 (s, 18H). ^{31}P NMR (162 MHz, $CDCl_3$) δ 34.93. ^{13}C NMR (101 MHz, $CDCl_3$) δ 166.22 (d, *J* = 4.2 Hz), 151.11 (d, *J* = 11.7 Hz), 143.48 (d, *J* = 9.9 Hz), 135.74, 134.87 (d, *J* = 98.8 Hz), 131.42 (d, *J* = 8.2 Hz), 131.26 (d, *J* = 2.4 Hz), 130.04 (d, *J* = 10.9 Hz), 129.78 (d, *J* = 7.8 Hz), 128.43, 128.08, 127.93, 127.42 (d, *J* = 94.3 Hz), 125.43 (d, *J* = 2.6 Hz), 124.03 (d, *J* = 10.6 Hz), 66.67, 34.95, 33.26 (d, *J* = 65.4 Hz), 31.24, 23.64 (t, *J* = 2.9 Hz). $[\alpha]^{22}_{D}$ = -0.11 (*c* 0.5, $CHCl_3$); Enantiomeric excess: 91%, determined by HPLC (Chiralpak IF, hexane/*i*-PrOH = 80/20; flow rate 0.8 ml/min; 25 °C; 290 nm), first peak: t_R = 8.2 min, second peak: t_R = 10.3 min. HRMS (ESI) calcd. For $C_{33}H_{41}NaO_3P$ [M+Na]$^+$: 539.2686, found: 539.2692.

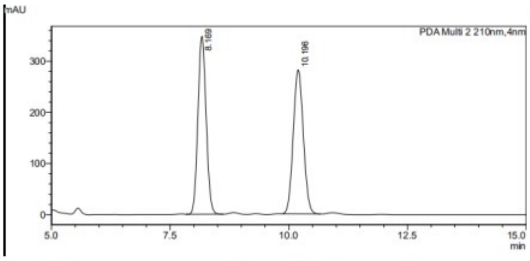

Peak#	Ret. Time	Area	Area%	Height	Height%
1	8.169	4196097	50.125	348167	55.266
2	10.196	4175227	49.875	281821	44.734
Total		8371323	100.000	629988	100.000

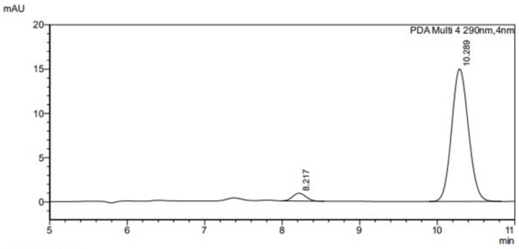

<Peak Table>

PDA Ch4 290nm

Peak#	Ret. Time	Area	Area%	Height	Height%
1	8.217	10408	4.398	883	5.579
2	10.289	226253	95.602	14951	94.421
Total		236661	100.000	15834	100.000

4.25 (*R*)-(4-isopropylphenyl)(mesityl)phosphine oxide ((*R*)-1m).

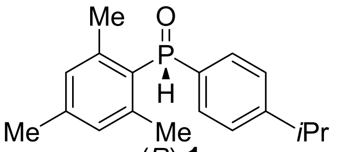

(*R*)-**1m**

(*R*)-**1m** (18.5 mg, 33%) was prepared as a white solid following general procedure B. $[\alpha]^{20}_{D}$ = 0.11 (*c* 0.5, $CHCl_3$); Enantiomeric excess: 98%, determined by HPLC (Chiralpak IF, hexane/*i*-PrOH = 70/30; flow rate 0.8 ml/min; 25 °C; 210 nm), first peak: t_R = 12.4 min, second peak: t_R = 13.5 min. For $C_{18}H_{23}NaOP$ $[M+Na]^+$: 309.2001, found: 309.2003.

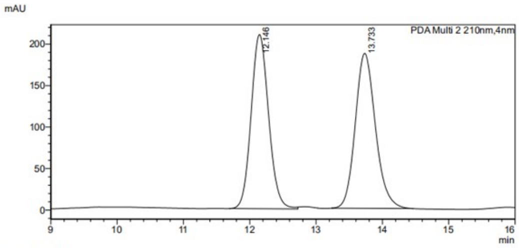

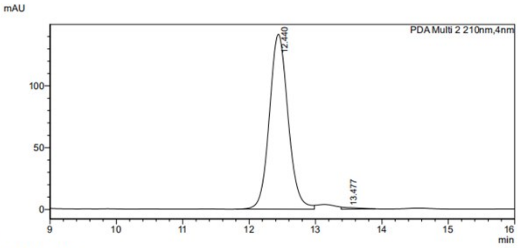

<Peak Table>
PDA Ch2 210nm

Peak#	Ret. Time	Area	Area%	Height	Height%
1	12.146	3845298	49.261	209868	52.932
2	13.733	3960677	50.739	186615	47.068
Total		7805975	100.000	396482	100.000

<Peak Table>
PDA Ch2 210nm

Peak#	Ret. Time	Area	Area%	Height	Height%
1	12.440	2772197	99.197	141652	99.143
2	13.477	22431	0.803	1224	0.857
Total		2794627	100.000	142876	100.000

4.26 (*R*)-(4-fluorophenyl)(mesityl)phosphine oxide ((*R*)-1n).

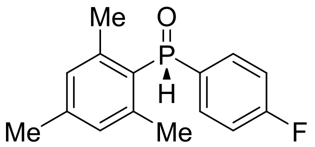

(*R*)-**1n**

(*R*)-**1n** (14.6 mg, 29%) was prepared as a white solid following general procedure B. $[\alpha]^{20}_{D}$ = 0.23 (*c* 0.5, $CHCl_3$); Enantiomeric excess: 93%, determined by HPLC (Chiralpak IF, hexane/*i*-PrOH = 70/30; flow rate 0.8 ml/min; 25 °C; 210 nm), first peak: t_R = 11.7 min, second peak: t_R = 12.8 min. For $C_{15}H_{16}FNaOP$ $[M+Na]^+$: 285.0834, found: 285.0831.

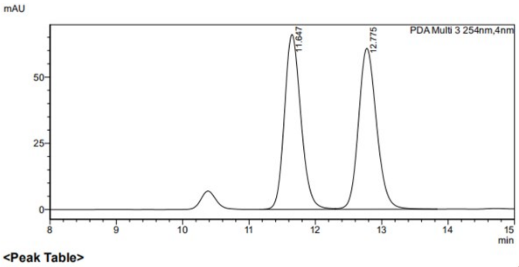

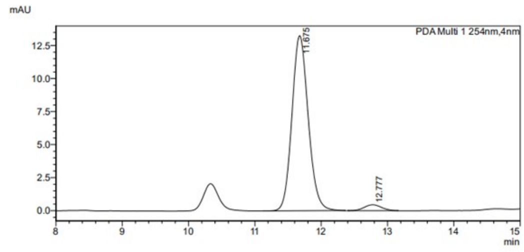

<Peak Table>
PDA Ch3 254nm

Peak#	Ret. Time	Area	Area%	Height	Height%
1	11.647	1138800	49.755	65976	52.083
2	12.775	1150002	50.245	60699	47.917
Total		2288802	100.000	126675	100.000

PDA Ch1 254nm

Peak#	Ret. Time	Area	Area%	Height	Height%
1	11.675	226137	96.578	13241	96.748
2	12.777	8014	3.422	445	3.252
Total		234151	100.000	13686	100.000

4.27 (*R*)-mesityl(4-methoxyphenyl)phosphine oxide ((*R*)-1o)

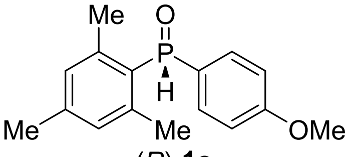

(*R*)-**1o**

(*R*)-**1o** (16.1 mg, 31%) was prepared as a white solid following general procedure B. $[\alpha]^{20}_{D}$ = 1.34 (*c* 0.5, $CHCl_3$); Enantiomeric excess: 99%, determined by HPLC (Chiralpak IF, hexane/*i*-PrOH = 70/30; flow rate 0.8 ml/min; 25 °C; 210 nm), first peak: t_R = 17.2 min, second peak: t_R = 18.4 min. For $C_{16}H_{19}NaO_2P$ $[M+Na]^+$: 297.1015, found: 297.1015.

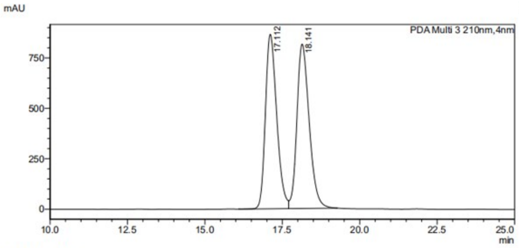

<Peak Table>

PDA Ch3 210nm

Peak#	Ret. Time	Area	Area%	Height	Height%
1	17.112	21969205	49.420	865947	51.504
2	18.141	22484696	50.580	815359	48.496
Total		44453901	100.000	1681306	100.000

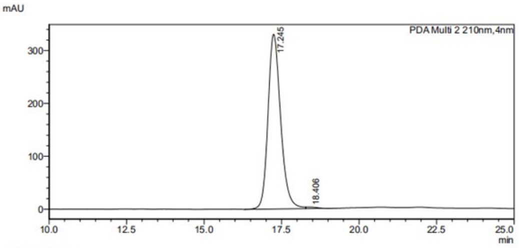

<Peak Table>

PDA Ch2 210nm

Peak#	Ret. Time	Area	Area%	Height	Height%
1	17.245	9382848	99.284	330373	99.116
2	18.406	67626	0.716	2948	0.884
Total		9450474	100.000	333321	100.000

4.28 (*R*)-mesityl(naphthalen-2-yl)phosphine oxide ((*R*)-1p).

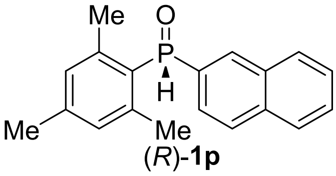

(*R*)-**1p**

(*R*)-**1p** (16.7 mg, 30%) was prepared as a white solid following general procedure B. $[\alpha]^{20}_{D}$ = 1.38 (*c* 0.5, $CHCl_3$); Enantiomeric excess: 90%, determined by HPLC (Chiralpak IF, hexane/*i*-PrOH = 70/30; flow rate 0.8 ml/min; 25 °C; 254 nm), first peak: t_R = 16.1 min, second peak: t_R = 17.7 min. For $C_{19}H_{19}NaOP$ $[M+Na]^+$: 317.1066, found: 317.1073.

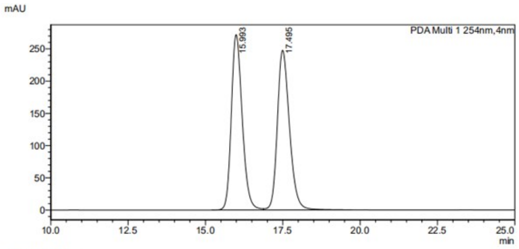

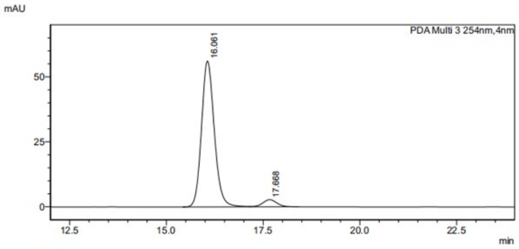

<Peak Table>
PDA Ch1 254nm

Peak#	Ret. Time	Area	Area%	Height	Height%
1	15.993	6677970	49.696	271979	52.340
2	17.495	6759762	50.304	247658	47.660
Total		13437732	100.000	519637	100.000

<Peak Table>
PDA Ch3 254nm

Peak#	Ret. Time	Area	Area%	Height	Height%
1	16.061	1295267	94.773	56139	95.307
2	17.668	71431	5.227	2764	4.693
Total		1366698	100.000	58904	100.000

4.29 (*R*)-(3-fluorophenyl)(mesityl)phosphine oxide ((*R*)-1q).

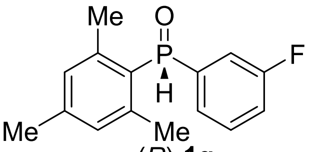

(*R*)-**1q**

(*R*)-**1q** (13.0 mg, 26%) was prepared as a white solid following general procedure B. $[\alpha]^{20}_{D}$ = 0.24 (*c* 0.5, $CHCl_3$); Enantiomeric excess: 91%, determined by HPLC (Chiralpak ID, hexane/*i*-PrOH = 70/30; flow rate 0.8 ml/min; 25 °C; 210 nm), first peak: t_R = 14.0 min, second peak: t_R = 15.0 min. For $C_{15}H_{16}FNaOP$ $[M+Na]^+$: 285.0815, found: 285.0819.

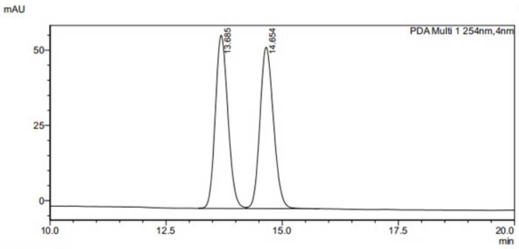

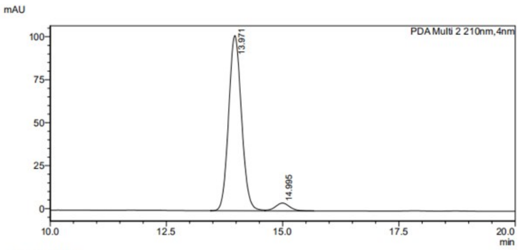

<Peak Table>
PDA Ch1 254nm

Peak#	Ret. Time	Area	Area%	Height	Height%
1	13.685	1083107	49.871	57535	51.770
2	14.654	1088691	50.129	53600	48.230
Total		2171798	100.000	111136	100.000

<Peak Table>
PDA Ch2 210nm

Peak#	Ret. Time	Area	Area%	Height	Height%
1	13.971	1966019	95.382	101884	95.790
2	14.995	95191	4.618	4478	4.210
Total		2061210	100.000	106362	100.000

4.30 (*S*)-mesityl(thiophen-3-yl)phosphine oxide ((*S*)-1r).

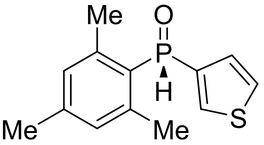

(*S*)-**1r**

(*S*)-**1r** was prepared as a brown solid following general procedure B. $[\alpha]^{20}_{D}$ = 0.53 (*c* 0.5, $CHCl_3$); Enantiomeric excess: 91%, determined by HPLC (Chiralpak IF, hexane/*i*-PrOH = 70/30; flow rate 0.8 ml/min; 25 °C; 210 nm), first peak: t_R = 12.8

min, second peak: t_R = 14.2 min. For $C_{13}H_{15}NaOPS$ $[M+Na]^+$: 273.0381, found: 285.0386.

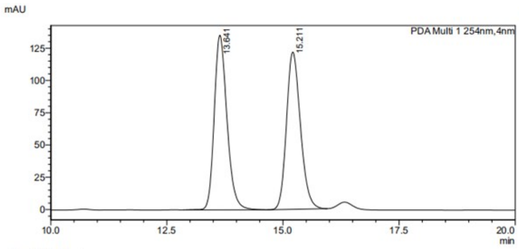

<Peak Table>

PDA Ch1 254nm

Peak#	Ret. Time	Area	Area%	Height	Height%
1	13.641	2586729	50.333	135199	52.600
2	15.211	2552551	49.667	121831	47.400
Total		5139280	100.000	257030	100.000

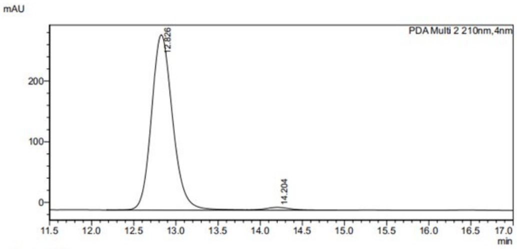

<Peak Table>

PDA Ch2 210nm

Peak#	Ret. Time	Area	Area%	Height	Height%
1	12.826	5031836	98.204	288635	98.438
2	14.204	92011	1.796	4579	1.562
Total		5123848	100.000	293214	100.000

4.31 benzyl (*S*)-2-(((4-isopropylphenyl)(mesityl)phosphoryl)methyl)acrylate (3mb).

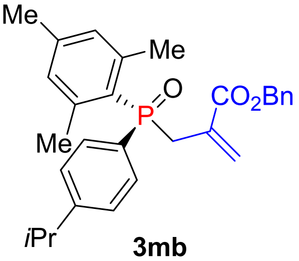

3mb

The general procedure C was followed using **2b** (0.1 mmol) and the corresponding SPO (0.25 mmol). After purification by column chromatography (PE/EtOAc = 1:1), **3mb** (40.0 mg, 88%) was obtained. ^{1}H NMR (500 MHz, $CDCl_3$) δ 7.50 (dd, J = 11.7, 8.2 Hz, 2H), 7.36 – 7.27 (m, 3H), 7.25 – 7.20 (m, 4H), 6.83 (d, J = 3.3 Hz, 2H), 6.40 (d, J = 4.6 Hz, 1H), 5.99 (d, J = 4.4 Hz, 1H), 4.95 (q, J = 12.5 Hz, 2H), 3.65 – 3.48 (m, 2H), 2.97 – 2.86 (m, 1H), 2.36 (s, 6H), 2.27 (s, 3H), 1.23 (d, J = 6.9 Hz, 6H). ^{31}P NMR (202 MHz, $CDCl_3$) δ 33.52. ^{13}C NMR (126 MHz, $CDCl_3$) δ 166.22 (d, J = 4.1 Hz), 152.47 (d, J = 2.6 Hz), 143.38 (d, J = 10.3 Hz), 141.42 (d, J = 2.6 Hz), 135.76, 133.44 (d, J = 100.4 Hz), 131.91 (d, J = 11.8 Hz), 131.30 (d, J = 8.3 Hz), 130.99 (d, J = 11.4 Hz), 130.02 (d, J = 10.1 Hz), 129.89 (d, J = 7.9 Hz), 128.43, 128.08, 127.92, 126.75 (d, J = 12.2 Hz), 124.23 (d, J = 97.2 Hz), 66.66, 34.10, 33.40 (d, J = 65.9 Hz), 23.69, 23.55 (d, J = 3.6 Hz), 20.99 (d, J = 1.0 Hz). $[\alpha]^{22}_D$ = 0.10 (c 0.5, $CHCl_3$); Enantiomeric excess: 94%, determined by HPLC (Chiralpak IF, hexane/i-PrOH = 70/30; flow rate 0.8 ml/min; 25 °C; 210 nm), first peak: t_R = 14.3 min, second peak: t_R

= 15.2 min. HRMS (ESI) calcd. For $C_{29}H_{33}NaO_3P$ $[M+Na]^+$: 483.2217, found: 483.2211.

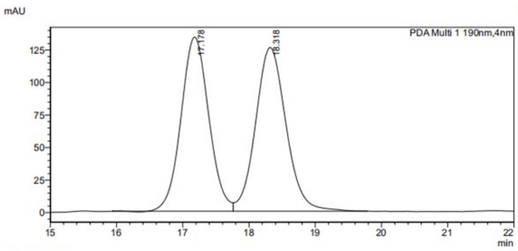

<Peak Table>

PDA Ch1 190nm

Peak#	Ret. Time	Area	Area%	Height	Height%
1	17.178	3898358	49.210	133855	51.544
2	18.318	4023532	50.790	125837	48.456
Total		7921889	100.000	259692	100.000

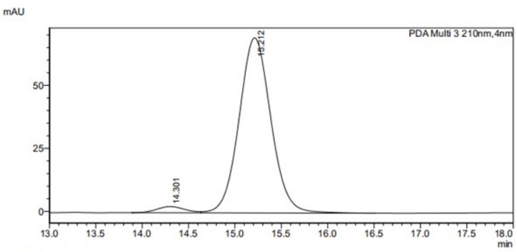

<Peak Table>

PDA Ch3 210nm

Peak#	Ret. Time	Area	Area%	Height	Height%
1	14.301	51510	3.011	2459	3.418
2	15.212	1659434	96.989	69486	96.582
Total		1710943	100.000	71945	100.000

4.32 benzyl (*S*)-2-(((4-fluorophenyl)(mesityl)phosphoryl)methyl)acrylate (3nb).

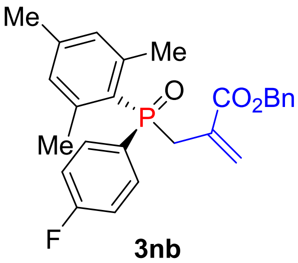

3nb

The general procedure C was followed using **2b** (0.1 mmol) and the corresponding SPO (0.25 mmol). After purification by column chromatography (PE/EtOAc = 1:1), **3nb** (32.6 mg, 75%) was obtained. ^{1}H NMR (500 MHz, $CDCl_3$) δ 7.62 – 7.54 (m, 2H), 7.38 – 7.30 (m, 3H), 7.25 – 7.21 (m, 2H), 7.10 – 7.03 (m, 2H), 6.85 (d, *J* = 3.5 Hz, 2H), 6.42 (d, *J* = 4.8 Hz, 1H), 5.99 (d, *J* = 4.5 Hz, 1H), 5.01 – 4.93 (m, 2H), 3.57 (d, *J* = 13.2 Hz, 2H), 2.36 (s, 6H), 2.28 (s, 3H). ^{19}F NMR (376 MHz, $CDCl_3$) δ -105.95 (d, *J* = 1.8 Hz). ^{31}P NMR (202 MHz, $CDCl_3$) δ 32.78. ^{13}C NMR (126 MHz, $CDCl_3$) δ 166.10 (d, *J* = 4.0 Hz), 164.48 (dd, *J* = 252.6, 3.1 Hz), 143.22 (d, *J* = 10.4 Hz), 141.81 (d, *J* = 2.7 Hz), 135.65, 132.66 (d, *J* = 3.6 Hz), 132.47 (dd, *J* = 11.2, 8.6 Hz), 131.86 (d, *J* = 3.5 Hz), 131.14 (d, *J* = 11.5 Hz), 130.99 (d, *J* = 8.5 Hz), 130.18 (d, *J* = 8.1 Hz), 128.46, 128.16, 127.98, 123.97 (d, *J* = 98.2 Hz), 115.87 (dd, *J* = 21.3, 12.9 Hz), 66.77, 33.80 (d, *J* = 66.2 Hz), 23.56 (d, *J* = 3.6 Hz), 20.99 (d, *J* = 0.9 Hz). $[\alpha]^{22}_D$ = 0.08 (*c* 0.5, $CHCl_3$); Enantiomeric excess: 93%, determined by HPLC (Chiralpak IF, hexane/*i*-PrOH = 70/30; flow rate 0.8 ml/min; 25 °C; 210 nm), first peak: t_R = 15.7 min, second peak: t_R = 19.9 min. HRMS (ESI) calcd. For $C_{26}H_{26}NaFO_3P$ $[M+Na]^+$: 459.1496, found: 459.1497.

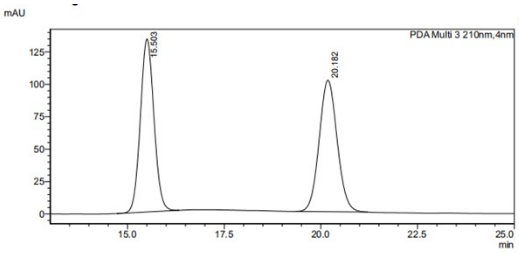

<Peak Table>
PDA Ch3 210nm

Peak#	Ret. Time	Area	Area%	Height	Height%
1	15.503	3268552	49.834	133398	56.806
2	20.182	3290268	50.166	101433	43.194
Total		6558820	100.000	234831	100.000

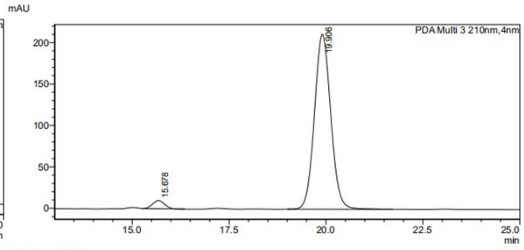

<Peak Table>
PDA Ch3 210nm

Peak#	Ret. Time	Area	Area%	Height	Height%
1	15.678	211399	3.305	9813	4.442
2	19.906	6185075	96.695	211109	95.558
Total		6396474	100.000	220922	100.000

4.33 benzyl (*S*)-2-((mesityl(4-methoxyphenyl)phosphoryl)methyl)acrylate (3ob).

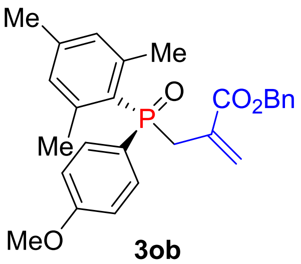

3ob

The general procedure C was followed using **2b** (0.1 mmol) and the corresponding SPO (0.25 mmol). After purification by column chromatography (PE/EtOAc = 1:1), **3ob** (36.5 mg, 81%) was obtained. ^{1}H NMR (400 MHz, $CDCl_3$) δ 7.51 (dd, *J* = 10.8, 8.7 Hz, 2H), 7.36 – 7.28 (m, 3H), 7.25 – 7.21 (m, 2H), 6.88 (d, *J* = 7.7 Hz, 2H), 6.83 (s, 2H), 6.40 (d, *J* = 4.0 Hz, 1H), 5.98 (d, *J* = 3.0 Hz, 1H), 5.02 – 4.88 (m, 2H), 3.80 (s, 3H), 3.55 (d, *J* = 13.3 Hz, 2H), 2.36 (s, 6H), 2.26 (s, 3H). ^{31}P NMR (162 MHz, $CDCl_3$) δ 33.04. ^{13}C NMR (101 MHz, $CDCl_3$) δ 166.18 (d, *J* = 3.5 Hz), 161.84, 143.26 (d, *J* = 10.3 Hz), 141.36, 135.73, 131.80 (d, *J* = 11.0 Hz), 131.32 (d, *J* = 8.2 Hz), 130.98 (d, *J* = 11.2 Hz), 129.76 (d, *J* = 7.6 Hz), 128.37, 128.02, 127.87, 114.04 (d, *J* = 12.7 Hz), 66.62, 55.19 (d, *J* = 3.1 Hz), 33.57 (d, *J* = 66.6 Hz), 23.50, 20.92 (d, *J* = 1.3 Hz). $[\alpha]^{22}_D$ = 0.45 (*c* 0.5, $CHCl_3$); Enantiomeric excess: 90%, determined by HPLC (Chiralpak IF, hexane/*i*-PrOH = 70/30; flow rate 0.8 ml/min; 25 °C; 210 nm), first peak: t_R = 32.9 min, second peak: t_R = 41.6 min. HRMS (ESI) calcd. For $C_{27}H_{29}NaO_4P$ [M+Na]$^+$: 471.1696, found: 471.1696.

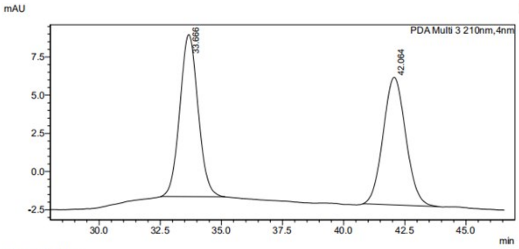

<Peak Table>
PDA Ch3 210nm

Peak#	Ret. Time	Area	Area%	Height	Height%
1	33.666	551139	50.552	10604	55.952
2	42.064	539105	49.448	8348	44.048
Total		1090244	100.000	18951	100.000

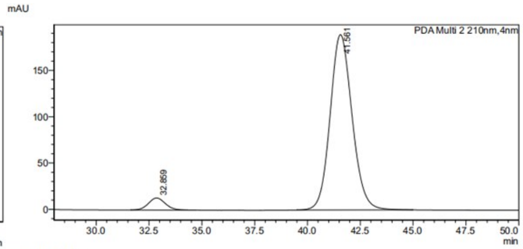

<Peak Table>
PDA Ch2 210nm

Peak#	Ret. Time	Area	Area%	Height	Height%
1	32.859	730721	5.060	13020	6.435
2	41.561	13711354	94.940	189319	93.565
Total		14442075	100.000	202339	100.000

4.34 benzyl (*S*)-2-((mesityl(*o*-tolyl)phosphoryl)methyl)acrylate (3pb).

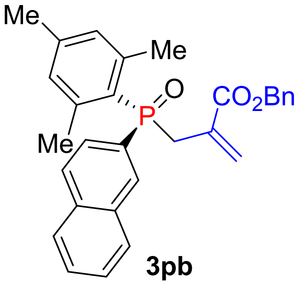

3pb

The general procedure C was followed using **2b** (0.1 mmol) and the corresponding SPO (0.25 mmol). After purification by column chromatography (PE/EtOAc = 1:1), **3pb** (33.0 mg, 71%) was obtained. ^{1}H NMR (400 MHz, $CDCl_3$) δ 8.17 (d, *J* = 13.4 Hz, 1H), 7.84 (d, *J* = 8.4 Hz, 3H), 7.61 – 7.48 (m, 3H), 7.33 – 7.27 (m, 3H), 7.20 – 7.14 (m, 2H), 6.86 (d, *J* = 3.1 Hz, 2H), 6.43 (d, *J* = 4.7 Hz, 1H), 6.02 (d, *J* = 4.3 Hz, 1H), 4.85 (q, *J* = 31.6 Hz, 2H), 3.69 (d, *J* = 13.2 Hz, 2H), 2.39 (s, 6H), 2.29 (s, 3H). ^{31}P NMR (162 MHz, $CDCl_3$) δ 33.73. ^{13}C NMR (101 MHz, $CDCl_3$) δ 166.12 (d, *J* = 3.9 Hz), 143.32 (d, *J* = 10.4 Hz), 141.65 (d, *J* = 2.5 Hz), 135.62, 134.34 (d, *J* = 2.0 Hz), 133.36 (d, *J* = 98.3 Hz), 132.48 (d, *J* = 12.9 Hz), 131.28 (d, *J* = 9.3 Hz), 131.08 (d, *J* = 10.9 Hz), 131.08 (d, *J* = 10.9 Hz), 130.08 (d, *J* = 8.2 Hz), 128.82, 128.50, 128.38, 127.94 (d, *J* = 22.9 Hz), 127.91, 127.76, 126.75, 125.44 (d, *J* = 10.5 Hz), 66.65, 33.65 (d, *J* = 65.6 Hz), 23.65 (d, *J* = 1.5 Hz), 21.01. $[\alpha]^{22}_D$ = 0.10 (*c* 0.5, $CHCl_3$); Enantiomeric excess: 91%, determined by HPLC (Chiralpak IF, hexane/*i*-PrOH = 70/30; flow rate 0.8 ml/min; 25 °C; 190 nm), first peak: t_R = 21.0 min, second peak: t_R = 35.8 min. HRMS (ESI) calcd. For $C_{30}H_{29}NaO_3P$ [M+Na]$^+$: 491.1747, found: 491.1741.

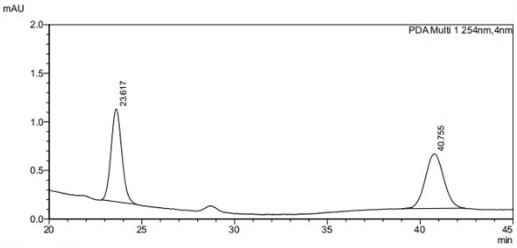

<Peak Table>
PDA Ch1 254nm

Peak#	Ret. Time	Area	Area%	Height	Height%
1	23.617	38217	49.700	954	62.973
2	40.755	38679	50.300	561	37.027
Total		76896	100.000	1514	100.000

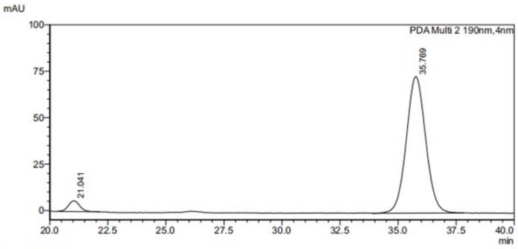

<Peak Table>
PDA Ch2 190nm

Peak#	Ret. Time	Area	Area%	Height	Height%
1	21.041	196246	4.391	5804	7.317
2	35.769	4273459	95.609	73516	92.683
Total		4469705	100.000	79320	100.000

4.35 benzyl (*S*)-2-(((3-fluorophenyl)(mesityl)phosphoryl)methyl)acrylate (3qb).

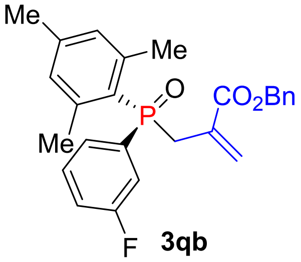

3qb

The general procedure C was followed using **2b** (0.1 mmol) and the corresponding SPO (0.25 mmol). After purification by column chromatography (PE/EtOAc = 1:1), **3qb** (33.9 mg, 78%) was obtained. ^{1}H NMR (500 MHz, $CDCl_3$) δ 7.44 – 7.36 (m, 2H), 7.35 – 7.28 (m, 4H), 7.23 (dd, *J* = 7.3, 1.8 Hz, 2H), 7.17 – 7.12 (m, 1H), 6.85 (d, *J* = 3.6 Hz, 2H), 6.43 (d, *J* = 4.9 Hz, 1H), 6.03 (d, *J* = 4.5 Hz, 1H), 4.96 (q, *J* = 12.4 Hz, 2H), 3.73 – 3.52 (m, 2H), 2.37 (s, 6H), 2.28 (s, 3H). ^{31}P NMR (202 MHz, $CDCl_3$) δ 33.33 (d, *J* = 5.1 Hz). ^{19}F NMR (376 MHz, $CDCl_3$) δ -111.04 (d, *J* = 5.3 Hz). ^{13}C NMR (126 MHz, $CDCl_3$) δ 166.03 (d, *J* = 4.0 Hz), 162.52 (dd, *J* = 250.1, 16.4 Hz),, 143.34 (d, *J* = 10.5 Hz), 142.01 (d, *J* = 2.7 Hz), 138.77 (dd, *J* = 96.5, 5.2 Hz), 135.63, 132.77, 131.17 (d, *J* = 11.6 Hz), 130.80 – 130.35 (m), 129.91, 128.45, 128.20, 128.15, 127.97, 125.68 (dd, *J* = 9.2, 3.2 Hz), 123.26 (d, *J* = 98.8 Hz), 118.47 (dd, *J* = 21.2, 2.3 Hz), 116.94 (dd, *J* = 22.2, 10.7 Hz), 66.77, 33.46 (d, *J* = 66.2 Hz), 23.52 (d, *J* = 3.8 Hz), 21.02 (d, *J* = 1.1 Hz). $[\alpha]^{22}_D$ = 0.12 (*c* 0.5, $CHCl_3$); Enantiomeric excess: 91%, determined by HPLC (Chiralpak ID, hexane/*i*-PrOH = 70/30; flow rate 0.8 ml/min; 25 °C; 210 nm), first peak: t_R = 21.9 min, second peak: t_R = 37.9 min. HRMS (ESI) calcd. For $C_{26}H_{26}NaFO_3P$ $[M+Na]^+$: 459.1496, found: 459.1498.

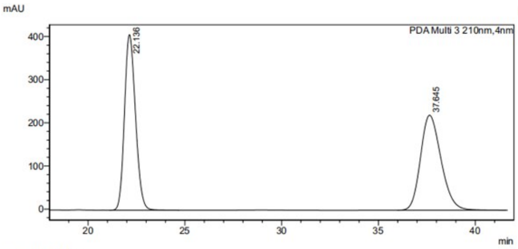

<Peak Table>
PDA Ch3 210nm

Peak#	Ret. Time	Area	Area%	Height	Height%
1	22.136	16114627	49.788	407061	64.885
2	37.645	16251790	50.212	220295	35.115
Total		32366417	100.000	627356	100.000

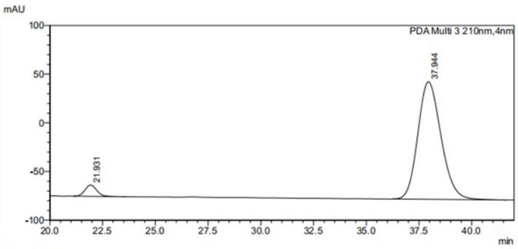

<Peak Table>
PDA Ch3 210nm

Peak#	Ret. Time	Area	Area%	Height	Height%
1	21.931	447841	4.625	11589	8.758
2	37.944	9234199	95.375	120729	91.242
Total		9682040	100.000	132317	100.000

4.36 benzyl (*R*)-2-((mesityl(thiophen-3-yl)phosphoryl)methyl)acrylate (3rb).

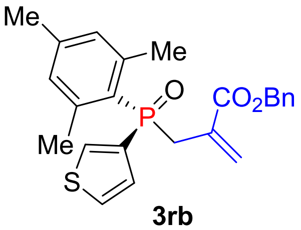

3rb

The general procedure C was followed using **2b** (0.1 mmol) and the corresponding SPO (0.25 mmol). After purification by column chromatography (PE/EtOAc = 1:1), **3rb** (33.6 mg, 79%) was obtained. ^{1}H NMR (400 MHz, $CDCl_3$) δ 7.71 (ddd, *J* = 7.3, 2.7, 1.1 Hz, 1H), 7.38 – 7.30 (m, 4H), 7.26 – 7.22 (m, 2H), 7.22 – 7.17 (m, 1H), 6.84 (d, *J* = 3.6 Hz, 2H), 6.44 (d, *J* = 4.9 Hz, 1H), 6.02 (d, *J* = 4.6 Hz, 1H), 4.99 (q, *J* = 12.4 Hz, 2H), 3.65 – 3.52 (m, 2H), 2.37 (s, 6H), 2.27 (s, 3H). ^{31}P NMR (162 MHz, $CDCl_3$) δ 26.57. ^{13}C NMR (101 MHz, $CDCl_3$) δ 166.18 (d, *J* = 4.0 Hz), 143.21 (d, *J* = 10.7 Hz), 141.66 (d, *J* = 2.8 Hz), 137.78, 136.78, 135.66, 132.23 (d, *J* = 14.0 Hz), 131.10 (d, *J* = 11.6 Hz), 131.05 (d, *J* = 8.7 Hz), 130.18 (d, *J* = 8.3 Hz), 128.58, 128.45, 128.15, 128.01, 127.14 (d, *J* = 14.6 Hz), 124.32 (d, *J* = 100.0 Hz), 66.78, 34.35 (d, *J* = 67.8 Hz), 23.32 (d, *J* = 3.7 Hz), 20.98 (d, *J* = 1.2 Hz). $[\alpha]^{22}_{D}$ = 0.11 (*c* 0.5, $CHCl_3$); Enantiomeric excess: 90%, determined by HPLC (Chiralpak IF, hexane/*i*-PrOH = 70/30; flow rate 0.8 ml/min; 25 °C; 210 nm), first peak: t_R = 16.5 min, second peak: t_R = 20.5 min. HRMS (ESI) calcd. For $C_{24}H_{25}NaO_3PS$ $[M+Na]^+$: 447.1154, found: 447.1162.

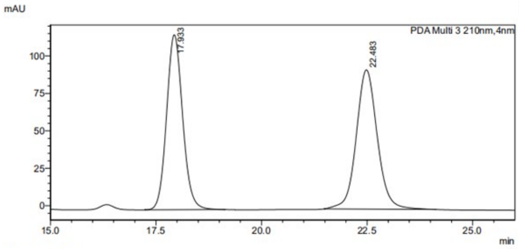

<Peak Table>

PDA Ch3 210nm

Peak#	Ret. Time	Area	Area%	Height	Height%
1	17.933	3096544	49.013	116857	55.674
2	22.483	3221275	50.987	93037	44.326
Total		6317819	100.000	209894	100.000

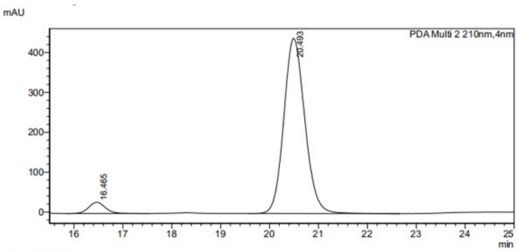

<Peak Table>

PDA Ch2 210nm

Peak#	Ret. Time	Area	Area%	Height	Height%
1	16.465	667808	4.830	28027	6.000
2	20.493	13159132	95.170	439057	94.000
Total		13826940	100.000	467084	100.000

4.37 benzyl (*S*)-2-((mesityl(*o*-tolyl)phosphoryl)methyl)acrylate (3sb).

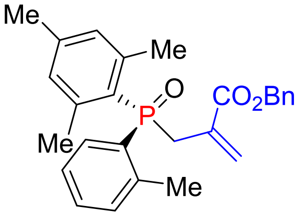

3sb

The general procedure C was followed using **2b** (0.1 mmol) and the corresponding SPO (0.25 mmol). After purification by column chromatography (PE/EtOAc = 1:1), **3sb** (29.9 mg, 69%) was obtained. ^{1}H NMR (500 MHz, $CDCl_3$) δ 7.66 (dd, *J* = 12.6, 7.6 Hz, 1H), 7.39 – 7.29 (m, 4H), 7.25 – 7.18 (m, 3H), 7.16 (dd, *J* = 7.4, 4.1 Hz, 1H), 6.77 (d, *J* = 3.4 Hz, 2H), 6.39 (d, *J* = 5.0 Hz, 1H), 6.00 (d, *J* = 4.6 Hz, 1H), 4.87 (q, *J* = 32 Hz, 2H), 3.72 (dd, *J* = 13.9, 10.1 Hz, 1H), 3.53 (dd, *J* = 16.7, 14.2 Hz, 1H), 2.29 (s, 6H), 2.24 (s, 3H), 2.23 (s, 3H). ^{31}P NMR (202 MHz, $CDCl_3$) δ 32.16. ^{13}C NMR (126 MHz, $CDCl_3$) δ 166.14 (d, *J* = 3.6 Hz), 142.68 (d, *J* = 10.5 Hz), 141.44 (d, *J* = 7.8 Hz), 141.21 (d, *J* = 2.7 Hz), 135.75, 134.61 (d, *J* = 97.7 Hz), 131.69 (d, *J* = 10.2 Hz), 131.32 (d, *J* = 2.5 Hz), 131.04 (d, *J* = 11.4 Hz), 130.87 (d, *J* = 7.9 Hz), 130.27 (d, *J* = 8.0 Hz), 129.93 (d, *J* = 10.7 Hz), 128.42, 128.07, 127.87, 125.55 (d, *J* = 11.8 Hz), 66.60, 32.41 (d, *J* = 65.5 Hz), 23.33 (d, *J* = 3.6 Hz), 20.96 (d, *J* = 1.2 Hz), 20.60 (d, *J* = 5.1 Hz). $[\alpha]^{22}_{D}$ = 1.1 (*c* 0.5, $CHCl_3$); Enantiomeric excess: 86%, determined by HPLC (Chiralpak IF, hexane/*i*-PrOH = 70/30; flow rate 0.8 ml/min; 25 °C; 254 nm), first peak: t_R = 15.3 min, second peak: t_R = 33.2 min. HRMS (ESI) calcd. For $C_{27}H_{29}NaO_3P$ $[M+Na]^+$: 455.1918, found: 455.1912.

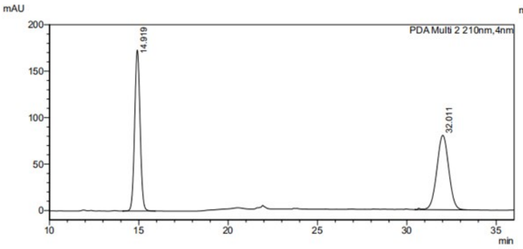

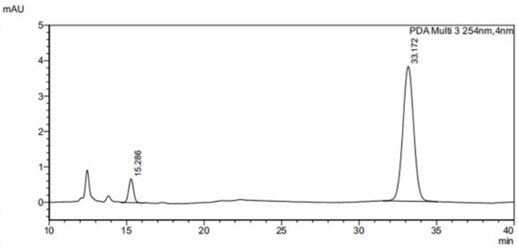

Peak#	Ret. Time	Area	Area%	Height	Height%
1	14.919	3681012	49.796	173295	68.374
2	32.011	3711187	50.204	80158	31.626
Total		7392200	100.000	253453	100.000

PDA Ch3 254nm

Peak#	Ret. Time	Area	Area%	Height	Height%
1	15.286	14114	7.044	669	14.953
2	33.172	186251	92.956	3805	85.047
Total		200365	100.000	4474	100.000

4.38 benzyl (*S*)-2-(([1,1'-biphenyl]-2-yl(mesityl)phosphoryl)methyl)acrylate (3tb).

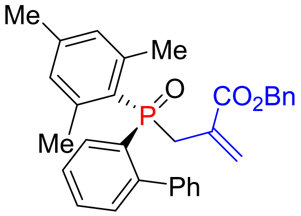

3tb

The general procedure C was followed using **2b** (0.1 mmol) and the corresponding SPO (0.25 mmol). After purification by column chromatography (PE/EtOAc = 1:1), **3tb** (29.9 mg, 70%) was obtained. ^{1}H NMR (500 MHz, $CDCl_3$) δ 7.83 – 7.76 (m, 1H), 7.53 (t, *J* = 7.5 Hz, 1H), 7.48 – 7.40 (m, 1H), 7.36 – 7.26 (m, 6H), 7.20 – 7.13 (m, 5H), 6.56 (d, *J* = 3.5 Hz, 2H), 6.28 (d, *J* = 5.1 Hz, 1H), 5.87 (d, *J* = 4.8 Hz, 1H), 4.81 (q, *J* = 30.0 Hz, 2H), 3.47 (dd, *J* = 14.0, 8.5 Hz, 1H), 3.19 (dd, *J* = 18.3, 14.1 Hz, 1H), 2.16 (s, 3H), 2.09 (s, 6H). ^{31}P NMR (202 MHz, $CDCl_3$) δ 33.66. ^{13}C NMR (126 MHz, $CDCl_3$) δ 166.03 (d, *J* = 3.5 Hz), 145.50 (d, *J* = 8.4 Hz), 142.49 (d, *J* = 10.4 Hz), 140.64 (d, *J* = 2.7 Hz), 140.35 (d, *J* = 4.1 Hz), 135.72, 134.76 (d, *J* = 97.0 Hz), 131.52 (d, *J* = 9.7 Hz), 131.12 (d, *J* = 8.0 Hz), 130.90 (d, *J* = 2.4 Hz), 130.50 (d, *J* = 11.7 Hz), 130.32 (d, *J* = 10.6 Hz), 129.85 (d, *J* = 8.3 Hz), 129.35, 128.33, 127.97, 127.76, 127.25, 127.23, 124.25 (d, *J* = 98.1 Hz), 66.40, 32.86 (d, *J* = 65.9 Hz), 22.94 (d, *J* = 3.7 Hz), 20.80 (d, *J* = 1.1 Hz). $[\alpha]^{22}_{D}$ = 0.71 (*c* 0.5, $CHCl_3$); Enantiomeric excess: 91%, determined by HPLC (Chiralpak IF, hexane/*i*-PrOH = 70/30; flow rate 0.8 ml/min; 25 °C; 210 nm), first peak: t_R = 15.9 min, second peak: t_R = 31.4 min. HRMS (ESI) calcd. For $C_{32}H_{31}NaO_3P$ $[M+Na]^+$: 517.2009, found: 517.2002.

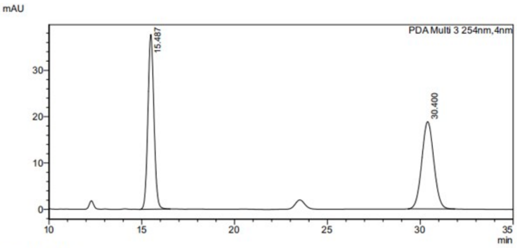

<Peak Table>
PDA Ch3 254nm

Peak#	Ret. Time	Area	Area%	Height	Height%
1	15.487	853040	50.084	37693	66.689
2	30.400	850183	49.916	18827	33.311
Total		1703222	100.000	56520	100.000

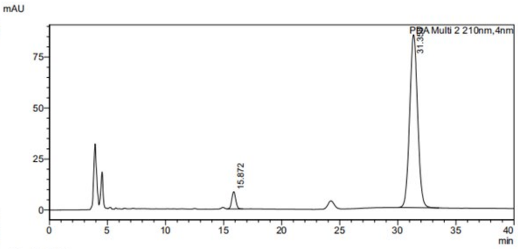

<Peak Table>
PDA Ch2 210nm

Peak#	Ret. Time	Area	Area%	Height	Height%
1	15.872	198550	4.670	8475	9.086
2	31.358	4053304	95.330	84799	90.914
Total		4251854	100.000	93274	100.000

4.39 benzyl (*S*)-2-((mesityl(naphthalen-1-yl)phosphoryl)methyl)acrylate (3ub).

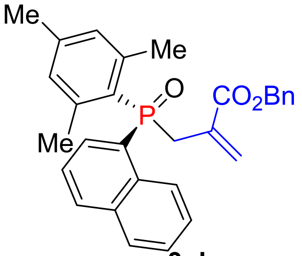

3ub

The general procedure C was followed using **2b** (0.1 mmol) and the corresponding SPO (0.25 mmol). After purification by column chromatography (PE/EtOAc = 1:1), **3ub** (28.6 mg, 60%) was obtained. ^{1}H NMR (400 MHz, $CDCl_3$) δ 8.28 (d, *J* = 8.5 Hz, 1H), 7.97 (d, *J* = 8.2 Hz, 1H), 7.93 – 7.79 (m, 2H), 7.51 – 7.43 (m, 2H), 7.41 – 7.28 (m, 4H), 7.24 – 7.18 (m, 2H), 6.78 (d, *J* = 3.5 Hz, 2H), 6.42 (d, *J* = 4.9 Hz, 1H), 6.02 (d, *J* = 4.6 Hz, 1H), 4.89 – 4.77 (m, 2H), 3.87 – 3.62 (m, 2H), 2.33 (s, 6H), 2.23 (s, 3H). ^{31}P NMR (162 MHz, $CDCl_3$) δ 32.69. ^{13}C NMR (126 MHz, $CDCl_3$) δ 166.07 (d, *J* = 3.4 Hz), 142.60 (d, *J* = 10.5 Hz), 141.27 (d, *J* = 2.4 Hz), 135.71, 133.69 (d, *J* = 8.8 Hz), 133.03, 132.74 (d, *J* = 7.8 Hz), 132.48, 132.26, 131.20 (d, *J* = 11.5 Hz), 131.04 (d, *J* = 7.9 Hz), 130.32 (d, *J* = 8.0 Hz), 129.77 (d, *J* = 10.1 Hz), 128.73, 128.39, 128.05, 127.86, 127.05, 126.32, 126.05 (d, *J* = 6.0 Hz), 124.48 (d, *J* = 13.4 Hz), 66.57, 65.81, 33.40 (d, *J* = 66.2 Hz), 23.62 (d, *J* = 3.2 Hz), 20.95, 15.24. $[\alpha]^{22}_D$ = 0.41 (*c* 0.5, $CHCl_3$); Enantiomeric excess: 90%, determined by HPLC (Chiralpak IF, hexane/*i*-PrOH = 70/30; flow rate 0.8 ml/min; 25 °C; 254 nm), first peak: t_R = 29.4 min, second peak: t_R = 31.7 min. HRMS (ESI) calcd. For $C_{30}H_{29}NaO_3P$ $[M+Na]^+$: 491.1747, found: 491.1750.

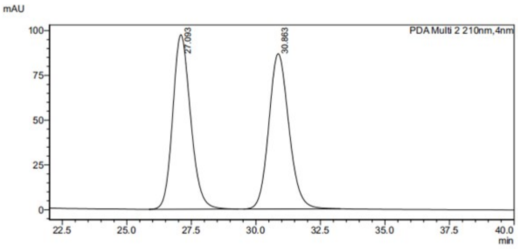

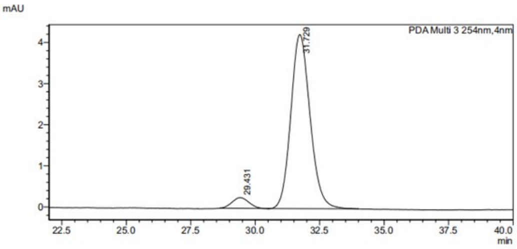

Peak#	Ret. Time	Area	Area%	Height	Height%
1	27.093	4702313	50.032	97409	52.926
2	30.863	4696222	49.968	86638	47.074
Total		9398534	100.000	184047	100.000

PDA Ch3 254nm

Peak#	Ret. Time	Area	Area%	Height	Height%
1	29.431	11805	5.087	260	5.796
2	31.729	220269	94.913	4233	94.204
Total		232074	100.000	4494	100.000

4.40 benzyl (*S*)-2-(((3,5-bis(trifluoromethyl)phenyl)(mesityl)phosphoryl)methyl) acrylate (3vb).

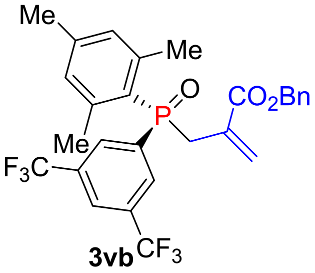

3vb

The general procedure C was followed using **2b** (0.1 mmol) and the corresponding SPO (0.25 mmol). After purification by column chromatography (PE/EtOAc = 5:1), **3vb** (36.5 mg, 66%) was obtained. ^{1}H NMR (400 MHz, $CDCl_3$) δ 8.18 – 8.14 (m, 1H), 8.04 (s, 1H), 7.96 (s, 1H), 7.35 – 7.30 (m, 3H), 7.23 – 7.20 (m, 2H), 6.90 (d, *J* = 3.8 Hz, 2H), 6.48 (d, *J* = 5.2 Hz, 1H), 6.08 (d, *J* = 4.8 Hz, 1H), 4.94 (q, *J* = 30.8 Hz, 2H), 3.76 – 3.60 (m, 2H), 2.37 (s, 6H), 2.31 (s, 3H). ^{31}P NMR (162 MHz, $CDCl_3$) δ 33.07. ^{19}F NMR (376 MHz, $CDCl_3$) δ -62.87. ^{13}C NMR (101 MHz, $CDCl_3$) δ 165.83 (d, *J* = 4.0 Hz), 143.22 (d, *J* = 10.8 Hz), 142.87 (d, *J* = 2.7 Hz), 135.43, 134.52, 133.36 (d, *J* = 2.4 Hz), 133.02, 131.52 (d, *J* = 11.8 Hz), 130.56, 130.06, 129.69, 128.72 (d, *J* = 28.6 Hz), 128.42 (d, *J* = 12.6 Hz), 128.22, 128.20 (d, *J* = 10.4 Hz), 128.00, 66.95, 66.68, 33.82 (d, *J* = 66.5 Hz), 23.65 (d, *J* = 3.8 Hz), 21.08 (d, *J* = 1.1 Hz). $[\alpha]^{22}_{D}$ = 0.832 (*c* 0.5, $CHCl_3$); Enantiomeric excess: 91%, determined by HPLC (Chiralpak IF, hexane/*i*-PrOH = 70/30; flow rate 0.8 ml/min; 25 °C; 210 nm), first peak: t_R = 6.6 min, second peak: t_R = 7.2 min. For $C_{28}H_{25}NaF_6O_3P$ $[M+Na]^+$: 577.1431, found: 577.1437.

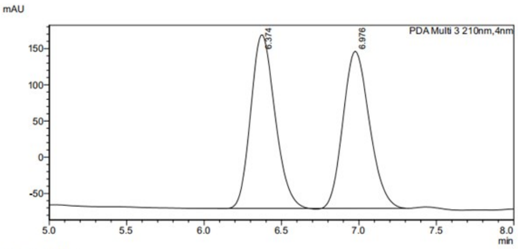

<Peak Table>

PDA Ch3 210nm

Peak#	Ret. Time	Area	Area%	Height	Height%
1	6.374	2623827	50.403	239217	52.456
2	6.976	2581917	49.597	216819	47.544
Total		5205745	100.000	456037	100.000

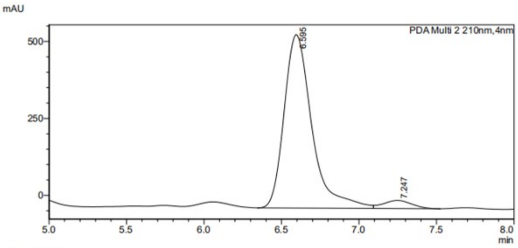

<Peak Table>

PDA Ch2 210nm

Peak#	Ret. Time	Area	Area%	Height	Height%
1	6.595	7019752	95.491	563654	95.566
2	7.247	331449	4.509	26153	4.434
Total		7351200	100.000	589807	100.000

4.41 benzyl (*S*)-2-(((4-methoxyphenyl)(2,4,6-triisopropylphenyl)phosphoryl) methyl)acrylate (3wb).

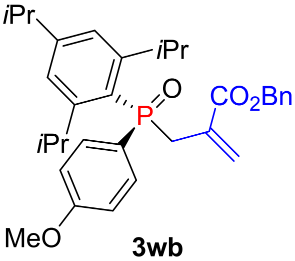

3wb

The general procedure C was followed using **2b** (0.1 mmol) and the corresponding SPO (0.25 mmol). After purification by column chromatography (PE/EtOAc = 1:1), **3wb** (33.3 mg, 63%) was obtained. ^{1}H NMR (400 MHz, $CDCl_3$) δ 7.54 (dd, *J* = 11.5, 8.7 Hz, 2H), 7.36 – 7.27 (m, 5H), 7.09 (d, *J* = 3.7 Hz, 2H), 6.87 (dd, *J* = 8.8, 2.1 Hz, 2H), 6.46 (d, *J* = 4.4 Hz, 1H), 5.89 (d, *J* = 4.1 Hz, 1H), 5.15 – 5.04 (m, 2H), 3.90 – 3.80 (m, 3H), 3.79 (s, 3H), 3.34 (t, *J* = 13.8 Hz, 1H), 2.95 – 2.84 (m, 1H), 1.26 (d, *J* = 6.9 Hz, 6H), 1.15 (d, *J* = 6.7 Hz, 6H), 0.96 (d, *J* = 6.6 Hz, 6H). ^{31}P NMR (162 MHz, $CDCl_3$) δ 33.51. ^{13}C NMR (101 MHz, $CDCl_3$) δ 166.35 (d, *J* = 4.1 Hz), 161.54 (d, *J* = 2.7 Hz), 154.41 (d, *J* = 11.2 Hz), 152.18 (d, *J* = 2.6 Hz), 135.81, 131.85 (d, *J* = 11.3 Hz), 131.82 (d, *J* = 8.4 Hz), 129.81, 129.73, 128.69, 128.39, 128.06, 128.02, 123.03 (d, *J* = 97.9 Hz), 122.87 (d, *J* = 11.2 Hz), 113.78 (d, *J* = 12.9 Hz), 66.72, 55.19, 34.46 (d, *J* = 66.9 Hz), 34.04, 29.99 (d, *J* = 4.3 Hz), 25.06, 24.25, 23.57 (d, *J* = 2.4 Hz). $[\alpha]^{22}_{D}$ = 0.38 (*c* 0.5, $CHCl_3$); Enantiomeric excess: 86%, determined by HPLC (Chiralpak IF, hexane/*i*-PrOH = 60/40; flow rate 0.8 ml/min; 25 °C; 234 nm), first peak: t_R = 23.3 min, second peak: t_R = 44.6 min. HRMS (ESI) calcd. For $C_{33}H_{41}NaO_4P$ $[M+Na]^+$: 555.2713, found: 555.2710.

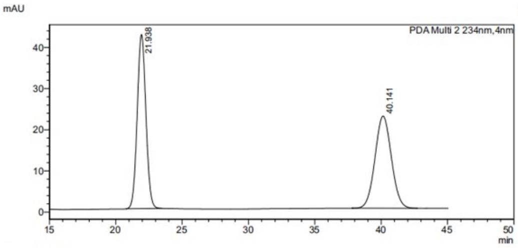

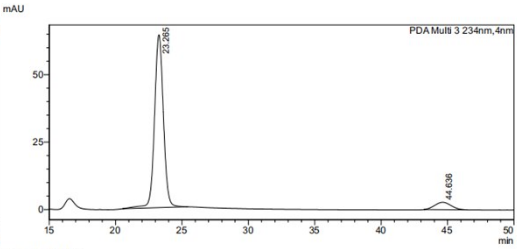

<Peak Table>
PDA Ch2 234nm

Peak#	Ret. Time	Area	Area%	Height	Height%
1	21.938	1927757	50.070	42281	65.390
2	40.141	1922402	49.930	22379	34.610
Total		3850159	100.000	64661	100.000

<Peak Table>
PDA Ch3 234nm

Peak#	Ret. Time	Area	Area%	Height	Height%
1	23.265	2994351	92.804	64129	95.888
2	44.636	232192	7.196	2750	4.112
Total		3226543	100.000	66879	100.000

4.42 *tert*-butyl (*R*)-2-((mesityl(propyl)phosphoryl)methyl)acrylate (3xc).

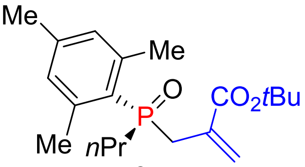

3xc

The general procedure D was followed using **2c** (0.1 mmol) and the corresponding SPO (0.30 mmol). After purification by column chromatography (PE/EtOAc = 1:2), **3xc** (21.5 mg, 62%) was obtained. ^{1}H NMR (500 MHz, $CDCl_3$) δ 6.86 (s, 2H), 6.25 (s, 1H), 5.85 (s, 1H), 3.24 (t, *J* = 12.0 Hz, 1H), 3.04 (t, *J* = 13.2 Hz, 1H), 2.59 (s, 6H), 2.25 (s, 3H), 2.06 (dd, *J* = 47.0, 12.6 Hz, 2H), 1.68 (d, *J* = 12.6 Hz, 1H), 1.44 (d, *J* = 7.6 Hz, 1H), 1.39 (s, 9H), 0.97 (t, *J* = 6.7 Hz, 3H). ^{31}P NMR (202 MHz, $CDCl_3$) δ 44.10. ^{13}C NMR (126 MHz, $CDCl_3$) δ 165.72, 142.60, 141.16, 132.36 (d, *J* = 8.2 Hz), 131.14 (d, *J* = 10.7 Hz), 128.86 (d, *J* = 6.0 Hz), 124.16 (d, *J* = 60.5 Hz), 119.00 (d, *J* = 1.7 Hz), 81.00, 34.63 (d, *J* = 43.0 Hz), 29.64, 27.79, 23.52, 20.84, 15.62 (d, *J* = 21.6 Hz). $[\alpha]^{22}_D$ = -0.06 (*c* 0.5, $CHCl_3$); Enantiomeric excess: 82%, determined by HPLC (Chiralpak IC, hexane/*i*-PrOH = 70/30; flow rate 0.8 ml/min; 25 °C; 254 nm), first peak: t_R = 9.4 min, second peak: t_R = 12.0 min. HRMS (ESI) calcd. For $C_{20}H_{31}NaO_3P$ $[M+Na]^+$: 373.1903, found: 373.1913.

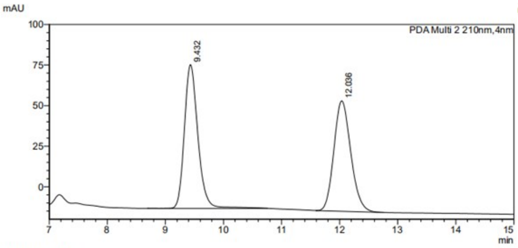

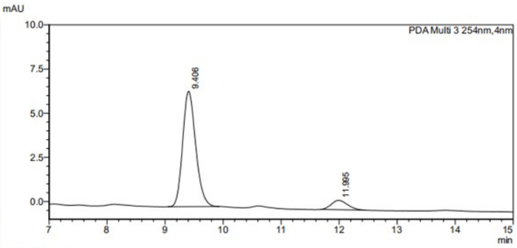

<Peak Table>
PDA Ch2 210nm

Peak#	Ret. Time	Area	Area%	Height	Height%
1	9.432	1435542	51.047	88574	56.565
2	12.036	1376666	48.953	68014	43.435
Total		2812208	100.000	156588	100.000

<Peak Table>
PDA Ch3 254nm

Peak#	Ret. Time	Area	Area%	Height	Height%
1	9.406	102146	90.843	6533	92.535
2	11.995	10297	9.157	527	7.465
Total		112442	100.000	7060	100.000

4.43 *tert*-butyl (*R*)-2-((isobutyl(mesityl)phosphoryl)methyl)acrylate (3yc).

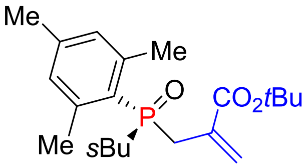

3yc

The general procedure D was followed using **2c** (0.1 mmol) and the corresponding SPO (0.30 mmol). After purification by column chromatography (PE/EtOAc = 1:2), **3yc** (21.5 mg, 62%) was obtained. ^{1}H NMR (500 MHz, $CDCl_3$) δ 6.85 (s, 2H), 6.24 (s, 1H), 5.86 (s, 1H), 3.12 (dt, *J* = 27.8, 13.6 Hz, 2H), 2.59 (s, 6H), 2.25 (s, 3H), 2.13 – 1.98 (m, 2H), 1.95 – 1.85 (m, 1H), 1.38 (s, 9H), 1.03 (d, *J* = 6.1 Hz, 3H), 0.88 (d, *J* = 6.1 Hz, 3H). ^{31}P NMR (202 MHz, $CDCl_3$) δ 42.86. ^{13}C NMR (126 MHz, $CDCl_3$) δ 165.77, 142.51 (d, *J* = 10.1 Hz), 141.06, 132.40 (d, *J* = 9.0 Hz), 131.18 (d, *J* = 11.1 Hz), 128.79 (d, *J* = 7.5 Hz), 125.10 (d, *J* = 88.6 Hz), 124.16 (d, *J* = 61.5 Hz), 80.92, 40.45 (d, *J* = 66.0 Hz), 35.33 (d, *J* = 60.4 Hz), 27.81, 24.59 (d, *J* = 11.2 Hz), 24.14 (d, *J* = 6.7 Hz), 23.91 (d, *J* = 3.3 Hz), 23.47, 20.84. $[\alpha]^{22}_D$ = 0.11 (*c* 0.5, $CHCl_3$); Enantiomeric excess: 87%, determined by HPLC (Chiralpak IC, hexane/*i*-PrOH = 70/30; flow rate 0.8 ml/min; 25 °C; 210 nm), first peak: t_R = 7.5 min, second peak: t_R = 9.8 min. HRMS (ESI) calcd. For $C_{21}H_{33}NaO_3P$ [M+Na]$^+$: 387.2216, found: 387.2212.

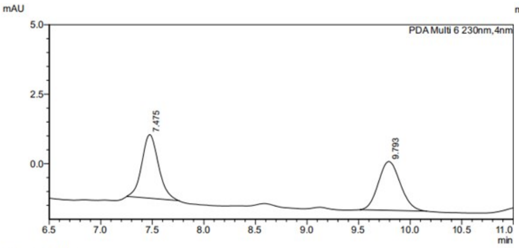

<Peak Table>
PDA Ch6 230nm

Peak#	Ret. Time	Area	Area%	Height	Height%
1	7.475	25831	49.786	2293	56.611
2	9.793	26053	50.214	1758	43.389
Total		51884	100.000	4051	100.000

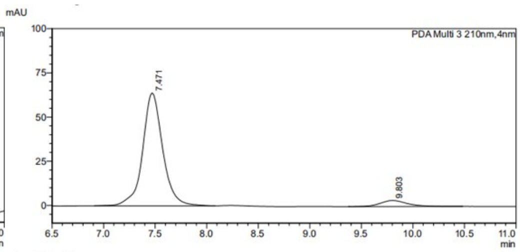

<Peak Table>
PDA Ch3 210nm

Peak#	Ret. Time	Area	Area%	Height	Height%
1	7.471	866627	93.237	63759	94.918
2	9.803	62857	6.763	3414	5.082
Total		929484	100.000	67173	100.000

4.44 benzhydryl (*S*)-2-((mesityl(phenyl)phosphoryl)methyl)acrylate (3ae).

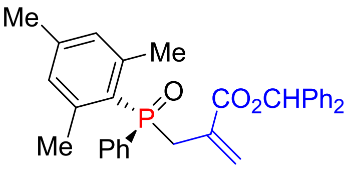

3ae

The general procedure A was followed using **1a** (0.21 mmol) and **2e** (0.1 mmol). After purification by column chromatography (PE/EtOAc = 1:1), **3ae** (40.4 mg, 39%) was obtained. ^{1}H NMR (500 MHz, $CDCl_3$) δ 7.60 – 7.55 (m, 2H), 7.45 – 7.40 (m, 1H), 7.36 – 7.26 (m, 10H), 7.21 – 7.17 (m, 2H), 6.79 (d, J = 3.4 Hz, 2H), 6.68 (s, 1H), 6.56 (d, J = 4.6 Hz, 1H), 6.17 (d, J = 4.0 Hz, 1H), 3.70 – 3.54 (m, 2H), 2.36 (s, 6H), 2.24 (s, 3H). ^{31}P NMR (202 MHz, $CDCl_3$) δ 33.82. ^{13}C NMR (126 MHz, $CDCl_3$) δ 165.26 (d, J = 4.4 Hz), 143.29 (d, J = 10.3 Hz), 141.54 (d, J = 2.7 Hz), 139.92 (d, J = 2.2 Hz), 136.64, 135.86, 131.23 (d, J = 2.6 Hz), 131.14 (d, J = 8.1 Hz), 131.04 (d, J = 11.4 Hz), 130.26 (d, J = 7.8 Hz), 129.81 (d, J = 9.8 Hz), 128.58 (d, J = 11.8 Hz), 128.38 (d, J = 13.0 Hz), 127.80 (d, J = 20.8 Hz), 126.96 (d, J = 33.2 Hz), 123.81 (d, J = 97.6 Hz), 77.49, 32.94 (d, J = 65.8 Hz), 23.49 (d, J = 3.7 Hz), 20.97 (d, J = 1.0 Hz). $[\alpha]^{22}_{D}$ = 0.55 (c 0.5, $CHCl_3$); Enantiomeric excess: 92%, determined by HPLC (Chiralpak IB, hexane/i-PrOH = 70/30; flow rate 0.8 ml/min; 25 °C; 210 nm), first peak: t_R = 7.0 min, second peak: t_R = 8.3 min. HRMS (ESI) calcd. For $C_{32}H_{31}NaO_3P$ $[M+Na]^+$: 517.2711, found: 517.2718.

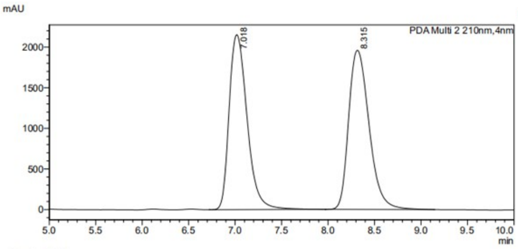

<Peak Table>

PDA Ch2 210nm

Peak#	Ret. Time	Area	Area%	Height	Height%
1	7.018	28936899	49.148	2153042	52.380
2	8.315	29939566	50.852	1957421	47.620
Total		58876465	100.000	4110463	100.000

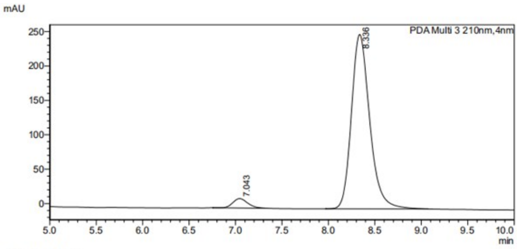

<Peak Table>

PDA Ch3 210nm

Peak#	Ret. Time	Area	Area%	Height	Height%
1	7.043	152607	4.205	13666	5.121
2	8.336	3476151	95.795	253203	94.879
Total		3628758	100.000	266869	100.000

4.45 2-(trifluoromethyl)benzyl (*S*)-2-((mesityl(phenyl)phosphoryl)methyl) acrylate (3af).

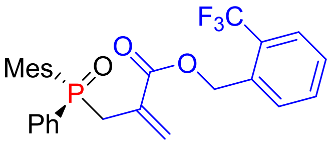

3af

The general procedure A was followed using **1a** (0.21 mmol) and **2f** (0.1 mmol). After purification by column chromatography (PE/acetone = 5:1), **3af** (31.7 mg, 32%) was obtained. ^{1}H NMR (300 MHz, $CDCl_3$) δ 7.68 – 7.50 (m, 4H), 7.46 – 7.35 (m, 5H),

6.84 (d, J = 3.5 Hz, 2H), 6.43 (d, J = 5.0 Hz, 1H), 6.03 (d, J = 4.5 Hz, 1H), 5.14 (s, 2H), 3.68 – 3.55 (m, 2H), 2.36 (s, 6H), 2.26 (s, 3H). ^{31}P NMR (122 MHz, $CDCl_3$) δ 33.96. ^{13}C NMR (126 MHz, $CDCl_3$) δ 165.85 (d, J = 4.0 Hz), 143.36 (d, J = 10.4 Hz), 141.68 (d, J = 2.7 Hz), 136.70, 135.92, 134.09, 131.99, 131.31 (d, J = 2.7 Hz), 131.10, 131.01, 130.26 (d, J = 8.0 Hz), 129.91 (d, J = 9.8 Hz), 128.63 (d, J = 11.8 Hz), 128.07, 126.00 (d, J = 5.6 Hz), 63.02 (d, J = 2.8 Hz), 33.40 (d, J = 65.6 Hz), 23.53 (d, J = 3.7 Hz), 20.96 (d, J = 1.2 Hz). $[\alpha]^{22}_D$ = 0.72 (c 0.5, $CHCl_3$); Enantiomeric excess: 89%, determined by HPLC (Chiralpak IE+IE, hexane/i-PrOH = 70/30; flow rate 0.8 ml/min; 25 °C; 210 nm), first peak: t_R = 42.3 min, second peak: t_R = 49.9 min. HRMS (ESI) calcd. For $C_{27}H_{26}NaF_3O_3P$ $[M+Na]^+$: 509.1634, found: 509.1630.

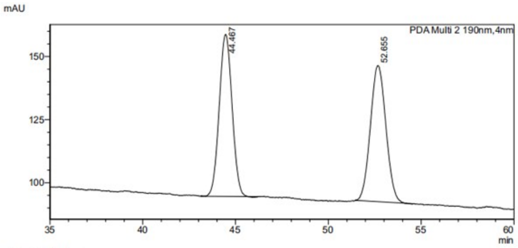

<Peak Table>

PDA Ch2 190nm

Peak#	Ret. Time	Area	Area%	Height	Height%
1	44.467	3086460	49.576	64203	54.325
2	52.655	3139258	50.424	53980	45.675
Total		6225718	100.000	118183	100.000

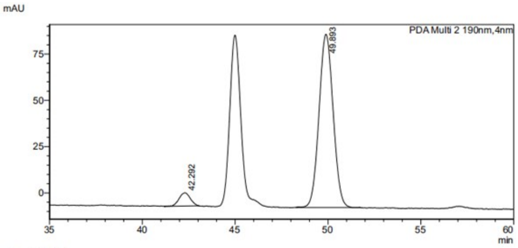

<Peak Table>

PDA Ch2 190nm

Peak#	Ret. Time	Area	Area%	Height	Height%
1	42.292	303079	5.782	7179	7.131
2	49.893	4938767	94.218	93504	92.869
Total		5241846	100.000	100683	100.000

5. Transformation of chiral SPOs and TPOs.

5.1 Allylic alkylation reaction of chiral SPO 1l.

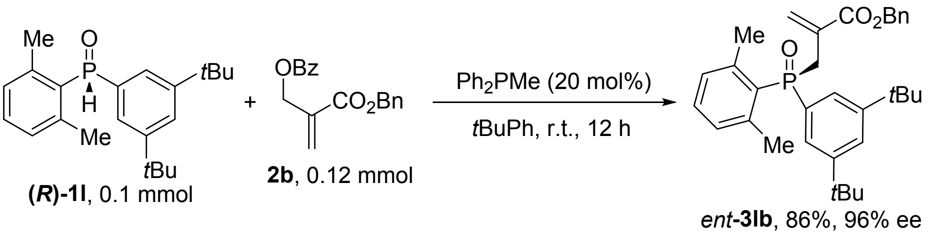

To a flame-dried glass tube with a magnetic stirring bar were added a neat mixture of SPO (*R*)-**1l** (34.2 mg, 0.1 mmol, 97% ee), and Ph_2PMe (4.0 mg, 0.02 mmol), followed by the addition of *t*BuPh (1.0 mL). Then MBH carbonates **2b** (35.5 mg, 0.12 mmol) was slowly added via syringe at room temperature under inert atmosphere.The mixture was stirred at room temperature for 12 h, and TLC show that the reaction was completed. The mixture was directly purified by column chromatography on silica gel (petroleum ether/ethyl acetate = 2:1) to afford the corresponding TPO *ent*-**3lb** (86%, 96% ee). $[\alpha]^{22}_D = 0.09$ (*c* 0.5, $CHCl_3$).

5.2 Arylation of chiral SPO 1l.[2]

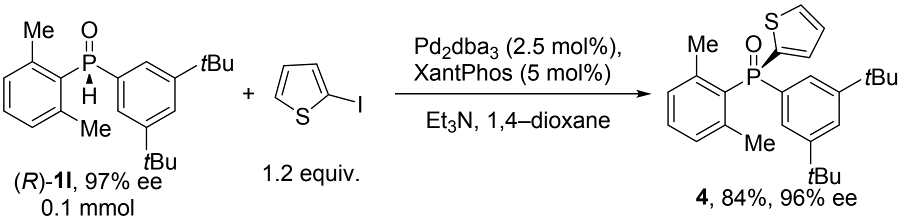

To a flame-dried glass tube with a magnetic stirring bar were added a neat mixture of SPO (*R*)-**1l** (34.2 mg, 0.1 mmol, 97% ee), 2-iodothiophene (25.2 mg, 0.12 mmol, 1.20 equiv.) and triethylamine (12.2 mg, 0.12 mmol, 1.2 equiv.), followed by the addition of dioxane (1.0 mL). In a separate flame-dried glass tube, Pd_2dba_3 (2.3 mg, 2.5 μmol, 2.5 mol%) and Xantphos (2.9 mg, 5.0 μmol, 5.0 mol%) was dissolved in dioxane (0.5 mL). During this time, the catalyst solution changed color from dark purple to brown. The catalyst solution was transferred to the vial containing the SPO and iodothiophene reagents.The reaction mixture was stirred for 12 h and TLC show that the reaction was completed. Then dioxane was removed under reduced pressure. The residue was purified by column chromatography on silica gel (petroleum ether/ethyl

acetate = 1:1) to afford **4** (84%, 96% *ee*). $[\alpha]^{22}_{D}$ = 1.65 (*c* 0.5, $CHCl_3$); ^{1}H NMR (400 MHz, $CDCl_3$) δ 7.70 (t, *J* = 4.3 Hz, 1H), 7.56 – 7.53 (m, 2H), 7.51 (d, *J* = 1.7 Hz, 1H), 7.44 – 7.40 (m, 1H), 7.31 – 7.26 (m, 1H), 7.18 – 7.14 (m, 1H), 7.05 (dd, *J* = 7.6, 4.0 Hz, 2H), 2.22 (s, 6H), 1.27 (s, 18H). ^{31}P NMR (162 MHz, $CDCl_3$) δ 24.09. ^{13}C NMR (101 MHz, $CDCl_3$) δ 151.21 (d, *J* = 12.5 Hz), 143.50 (d, *J* = 10.1 Hz), 137.17 (d, *J* = 107.6 Hz), 135.82 (d, *J* = 10.0 Hz), 134.62 (d, *J* = 108.6 Hz), 133.15 (d, *J* = 4.7 Hz), 131.53 (d, *J* = 2.6 Hz), 130.15 (d, *J* = 11.2 Hz), 129.40 (d, *J* = 104.5 Hz), 128.07 (d, *J* = 13.6 Hz), 125.68 (d, *J* = 2.8 Hz), 125.36 (d, *J* = 11.6 Hz), 34.99, 31.23, 23.62 (d, *J* = 4.5 Hz). Enantiomeric excess: 96%, determined by HPLC (Chiralpak IF, hexane/*i*-PrOH = 70/30; flow rate 0.8 ml/min; 25 °C; 254 nm), first peak: t_R = 6.6 min, second peak: t_R = 7.1 min. HRMS (ESI) calcd. For $C_{26}H_{33}NaOPS$ $[M+Na]^+$: 447.2057, found: 447.2051.

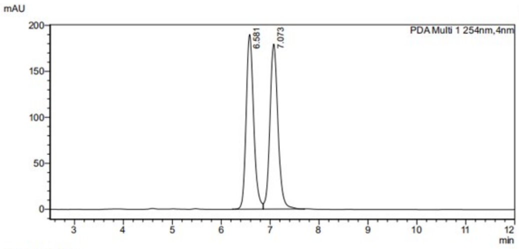

<Peak Table>
PDA Ch1 254nm

Peak#	Ret. Time	Area	Area%	Height	Height%
1	6.581	2024249	49.628	190008	51.414
2	7.073	2054572	50.372	179556	48.586
Total		4078821	100.000	369564	100.000

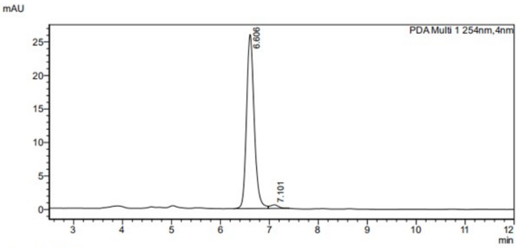

<Peak Table>
PDA Ch1 254nm

Peak#	Ret. Time	Area	Area%	Height	Height%
1	6.606	282791	97.846	25965	98.017
2	7.101	6225	2.154	525	1.983
Total		289016	100.000	26491	100.000

5.3 Base-promoted alkylation of chiral SPO 1l.[3]

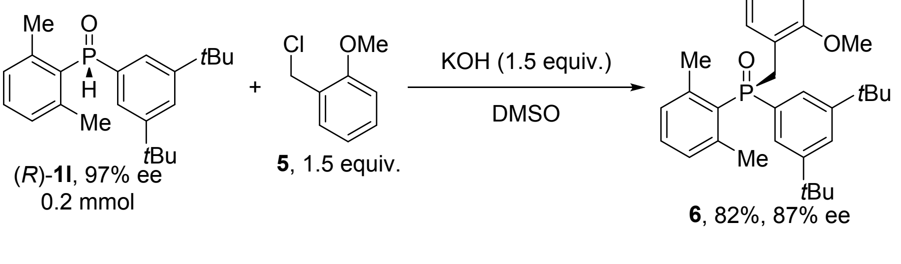

To the mixture of (*R*)-**1l** (68.4 mg, 0.2 mmol, 97% ee) and potassium hydroxide (17.0 mg, 0.3 mmol), DMSO (1.0 mL) was added, followed by the addition of benzyl chloride **5** (47.0 mg, 0.3 mmol). The mixture was stirred at room temperature for 24 h, and monitored with TLC. After the reaction was completed, brine (5 mL) was added. The mixture was extracted with DCM (3 × 5 mL), washed with water, dried over anhydrous Na_2SO_4. After removing the solvent in vacuo, the residue was purified with

column chromatography on silica gel (petroleum ether/ethyl acetate = 2/1) to afford desired product **6** (82%, 87% ee). $[\alpha]^{22}_{D}$ = 0.08 (*c* 0.5, $CHCl_3$); ^1H NMR (500 MHz, $CDCl_3$) δ 7.54 – 7.49 (m, 1H), 7.47 (d, *J* = 1.1 Hz, 1H), 7.33 (dd, *J* = 12.6, 1.8 Hz, 2H), 7.22 – 7.14 (m, 2H), 6.96 (dd, *J* = 7.5, 3.6 Hz, 2H), 6.91 (t, *J* = 7.4 Hz, 1H), 6.64 (d, *J* = 8.2 Hz, 1H), 3.92 – 3.77 (m, 2H), 3.32 (s, 3H), 2.27 (s, 6H), 1.24 (s, 18H). ^{31}P NMR (202 MHz, $CDCl_3$) δ 35.31. ^{13}C NMR (126 MHz, $CDCl_3$) δ 157.04 (d, *J* = 5.4 Hz), 150.71 (d, *J* = 11.5 Hz), 143.54 (d, *J* = 9.7 Hz), 135.56 (d, *J* = 97.4 Hz), 132.36 (d, *J* = 4.5 Hz), 130.75 (d, *J* = 2.6 Hz), 129.60 (d, *J* = 10.8 Hz), 128.74 (d, *J* = 93.0 Hz), 127.91 (d, *J* = 3.0 Hz), 125.00 (d, *J* = 2.6 Hz), 124.13 (d, *J* = 10.3 Hz), 120.61 (d, *J* = 8.0 Hz), 120.44 (d, *J* = 2.8 Hz), 109.98 (d, *J* = 2.4 Hz), 54.74, 34.91, 32.03 (d, *J* = 64.9 Hz), 31.27, 23.51 (d, *J* = 3.6 Hz). Enantiomeric excess: 87%, determined by HPLC (Chiralpak IC, hexane/*i*-PrOH = 70/30; flow rate 0.8 ml/min; 25 °C; 210 nm), first peak: t_R = 7.4 min, second peak: t_R = 7.9 min. HRMS (ESI) calcd. For $C_{30}H_{39}NaO_2P$ $[M+Na]^+$: 485.2057, found: 485.2051.

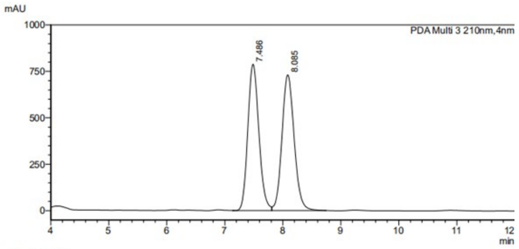

<Peak Table>
PDA Ch3 210nm

Peak#	Ret. Time	Area	Area%	Height	Height%
1	7.486	10453915	49.474	787700	51.875
2	8.085	10676298	50.526	730746	48.125
Total		21130213	100.000	1518446	100.000

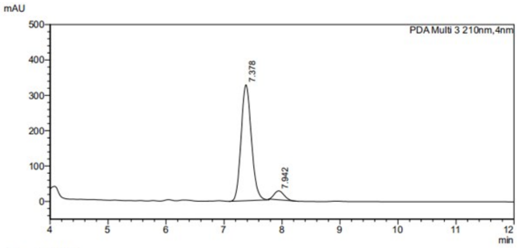

<Peak Table>
PDA Ch3 210nm

Peak#	Ret. Time	Area	Area%	Height	Height%
1	7.378	4046663	93.151	327930	92.852
2	7.942	297539	6.849	25246	7.148
Total		4344202	100.000	353177	100.000

5.4 Demethylation of TPO 6.[4]

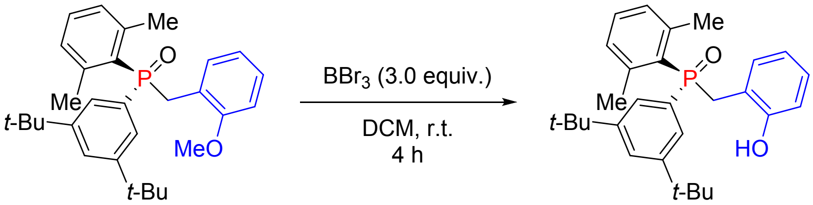

6 **7**, 63%, 88% ee

To a solution of **6** (46.2 mg, 0.1 mmol) in CH_2Cl_2 (1.0 mL) was added BBr_3 (75 mg, 0.3 mmol) at 0 °C. The reaction mixture was warmed to ambient temperature and stirred for 16 hours, then monitored with TLC, after which reaction was quenched by

addition of H_2O (0.5mL). The pH of the aqueous layer was adjusted to pH 8 using sat. $NaHCO_3$ (0.5 mL). The mixture was extracted with CH_2Cl_2 (3 × 5 mL), washed with sat. $NaHCO_3$, then brine, dried over anhydrous Na_2SO_4. After removing the solvent in vacuo, the residue was purified with column chromatography on silica gel (petroleum ether/ethyl acetate = 1/2) to afford desired product **7** as a white solid (63%, 88% ee). $[\alpha]^{22}_D$ = 0.03 (*c* 0.5, $CHCl_3$); ^{1}H NMR (400 MHz, $CDCl_3$) δ 10.36 (s, 1H), 7.54 (dd, *J* = 2.9, 1.7 Hz, 1H), 7.33 (d, *J* = 1.8 Hz, 1H), 7.31 – 7.27 (m, 2H), 7.15 – 7.08 (m, 1H), 7.06 (dd, *J* = 7.6, 3.9 Hz, 2H), 7.02 (d, *J* = 7.9 Hz, 1H), 6.70 – 6.64 (m, 2H), 3.85 (ddd, *J* = 41.8, 14.5, 12.4 Hz, 2H), 2.43 (s, 6H), 1.22 (s, 18H). ^{31}P NMR (162 MHz, $CDCl_3$) δ 45.90. ^{13}C NMR (101 MHz, $CDCl_3$) δ 156.68 (d, *J* = 3.8 Hz), 151.34 (d, *J* = 11.7 Hz), 143.17 (d, *J* = 9.8 Hz), 132.83 (d, *J* = 97.6 Hz), 131.77 (d, *J* = 2.6 Hz), 131.41 (d, *J* = 6.0 Hz), 130.22 (d, *J* = 10.9 Hz), 128.76 (d, *J* = 2.8 Hz), 126.07 (d, *J* = 2.7 Hz), 125.79 (d, *J* = 94.5 Hz), 123.98 (d, *J* = 10.6 Hz), 120.34 (dd, *J* = 5.2, 3.2 Hz), 119.40 (d, *J* = 2.6 Hz), 36.76 (d, *J* = 65.2 Hz), 34.91, 31.15, 23.63 (d, *J* = 3.9 Hz). Enantiomeric excess: 88%, determined by HPLC (Chiralpak OD-H, hexane/*i*-PrOH = 85/15; flow rate 0.8 ml/min; 25 °C; 254 nm), first peak: t_R = 5.6 min, second peak: t_R = 6.2 min. HRMS (ESI) calcd. For $C_{29}H_{37}NaO_2P$ [M+Na]$^+$: 471.2516, found: 471.2511.

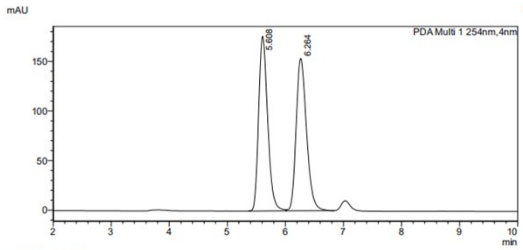

<Peak Table>
PDA Ch1 254nm

Peak#	Ret. Time	Area	Area%	Height	Height%
1	5.608	1905608	49.906	176168	53.406
2	6.264	1912780	50.094	153695	46.594
Total		3818387	100.000	329862	100.000

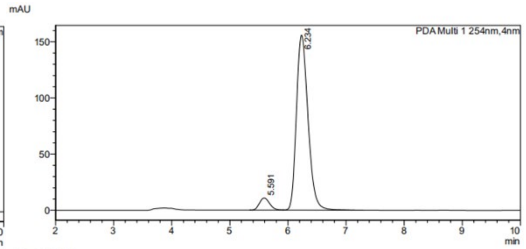

<Peak Table>
PDA Ch1 254nm

Peak#	Ret. Time	Area	Area%	Height	Height%
1	5.591	132837	5.776	10818	6.490
2	6.234	2166876	94.224	155854	93.510
Total		2299713	100.000	166671	100.000

5.5 Synthesis of chiral pincer-type ligand *via* chiral SPO 1a.

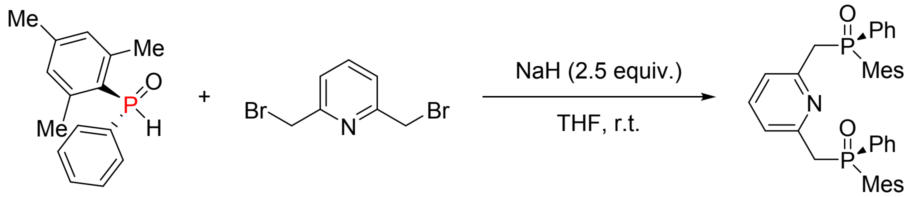

(*R*)-**1a**
2.1 equiv.

8, 47%, 81% *ee*

To a solution of (*R*)-**1a** (153.8 mg, 0.63 mmol) in THF (2 mL) was added NaH (30 mg, 0.75 mmol of a 60% w/w dispersion in oil) in portions at 0 °C. The reaction mixture was warmed to ambient temperature and after 60 minutes was cooled to 0 °C before 2,6-bis(bromomethyl)pyridine (79.2 mg, 0.3 mmol) was added and the mixture was then warmed to room temperature and stirred for a further 2 hour. The reaction mixture was then quenched with water and extracted with EtOAc (3 × 10 mL). The combined organic extracts were washed with brine and dried with sodium sulfate. After removing the solvent in vacuo, the residue was purified with column chromatography on silica gel (petroleum ether/ethyl acetate = 1/2) to afford desired product **8** (47%, 81% ee). $[\alpha]^{22}_D = 0.13$ (*c* 0.5, $CHCl_3$); ^1H NMR (500 MHz, $CDCl_3$) δ 7.66 – 7.57 (m, 2H), 7.46 – 7.42 (m, 4H), 7.39 – 7.35 (m, 4H), 7.28 (s, 1H), 4.01 (dt, *J* = 26.7, 14.1 Hz, 4H), 2.36 (s, 12H), 2.27 (s, 6H). ^{31}P NMR (202 MHz, $CDCl_3$) δ 34.99. ^{13}C NMR (126 MHz, $CDCl_3$) δ 157.68 (d, *J* = 1.6 Hz), 152.41 (d, *J* = 7.0 Hz), 143.17 (d, *J* = 10.2 Hz), 141.46 (d, *J* = 2.6 Hz), 136.42 (d, *J* = 98.4 Hz), 136.34 (d, *J* = 2.1 Hz), 131.18 (d, *J* = 2.7 Hz), 130.91 (d, *J* = 11.5 Hz), 130.12 (d, *J* = 9.9 Hz), 128.49 (d, *J* = 11.9 Hz), 122.00 (d, *J* = 3.7 Hz), 121.09 (d, *J* = 2.5 Hz), 42.20 (d, *J* = 63.0 Hz), 24.16, 23.59 (d, *J* = 3.7 Hz), 20.97 (d, *J* = 1.2 Hz). Enantiomeric excess: 81%, determined by HPLC (Chiralpak IF, hexane/*i*-PrOH = 70/30; flow rate 0.8 ml/min; 25 °C; 210 nm), first peak: t_R = 16.2 min, second peak: t_R = 24.9 min. HRMS (ESI) calcd. For $C_{37}H_{39}NNaO_2P_2$ $[M+Na]^+$: 614.2533, found: 614.2532.

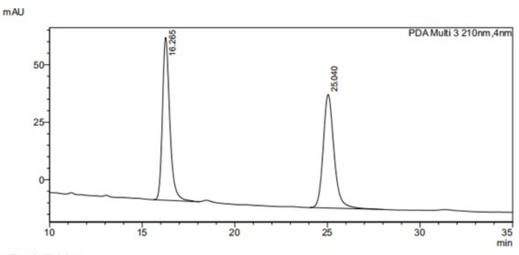

<Peak Table>
PDA Ch3 210nm

Peak#	Ret. Time	Area	Area%	Height	Height%
1	16.265	1958946	49.312	70762	58.907
2	25.040	2013590	50.688	49362	41.093
Total		3972536	100.000	120124	100.000

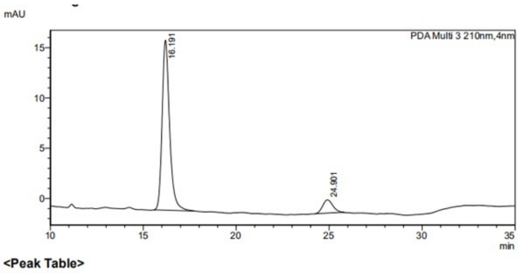

<Peak Table>
PDA Ch3 210nm

Peak#	Ret. Time	Area	Area%	Height	Height%
1	16.191	473826	90.448	16915	92.768
2	24.901	50041	9.552	1319	7.232
Total		523868	100.000	18234	100.000

5.6 Cyclopropanantion of enantiopure TPO 3ab.[5]

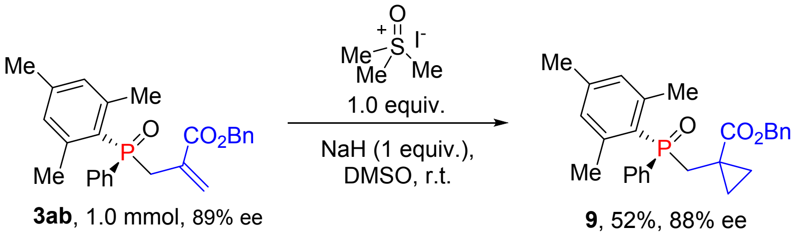

To a flame-dried glass tube with a magnetic stirring bar were added a neat mixture of sodium hydride (60%, 44 mg) and trimethylsulfoxonium iodide salt (220 mg, 1.0 mmol) was added, followed by dry DMSO (1 mL). The suspension was stirred for 1 h until evolution of hydrogen ceased. A solution of the chiral TPO **3ab** in dry DMSO (1 mL) was slowly added at 0 °C. The reaction mixture was warmed to ambient temperature and stirred for 4 hours.The mixture was poured into ice water and extracted with Et_2O (3 × 10 mL). The combined organic extracts were washed with water (2 × 10 mL) and dried with sodium sulfate. After removing the solvent in vacuo, the residue was purified with column chromatography on silica gel (petroleum ether/ethyl acetate = 2/1) to afford desired product **9** (52%, 88% ee). $[\alpha]^{22}_D$ = -0.16 (*c* 0.5, $CHCl_3$); ^1H NMR (500 MHz, $CDCl_3$) δ 7.59 (dd, *J* = 11.7, 7.3 Hz, 2H), 7.44 (t, *J* = 7.0 Hz, 1H), 7.39 – 7.33 (m, 2H), 7.32 – 7.28 (m, 3H), 7.22 – 7.18 (m, 2H), 6.85 (d, *J* = 3.0 Hz, 2H), 4.92 (d, *J* = 12.4 Hz, 1H), 4.79 (d, *J* = 12.4 Hz, 1H), 3.03 (dd, *J* = 15.2, 11.0 Hz, 1H), 2.68 (dd, *J* = 15.3, 8.0 Hz, 1H), 2.36 (s, 6H), 2.28 (s, 3H), 1.45 – 1.37 (m, 2H), 1.20 – 1.08 (m, 2H). ^{31}P NMR (202 MHz, $CDCl_3$) δ 35.31. ^{13}C NMR (126 MHz, $CDCl_3$) δ 173.86 (d, *J* = 1.3 Hz), 143.09 (d, *J* = 10.2 Hz), 141.33 (d, *J* = 2.6 Hz), 137.04 (d, *J* = 97.0 Hz), 135.90, 131.06 (d, *J* = 2.6 Hz), 131.05 (d, *J* = 11.1 Hz), 129.88 (d, *J* = 9.8 Hz), 128.53 (d, *J* = 11.6 Hz), 128.36, 128.03, 127.98, 125.28 (d, *J* = 95.3 Hz), 66.56, 34.62 (d, *J* = 70.5 Hz), 23.45 (d, *J* = 3.6 Hz), 20.98, 19.26 (d, *J* = 4.4 Hz), 16.39 (d, *J* = 6.3 Hz), 15.81 (d, *J* = 5.8 Hz). Enantiomeric excess: 88%, determined by HPLC (Chiralpak IC, hexane/*i*-PrOH = 70/30; flow rate 0.8 ml/min; 25 °C; 220 nm), first peak: t_R = 32.2 min, second peak: t_R = 44.1 min. HRMS (ESI) calcd. For $C_{27}H_{29}NaO_3P$ $[M+Na]^+$: 455.1747, found: 455.1752.

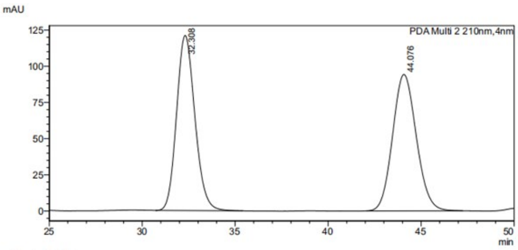

<Peak Table>

PDA Ch2 210nm

Peak#	Ret. Time	Area	Area%	Height	Height%
1	32.308	8305011	49.845	120880	56.213
2	44.076	8356626	50.155	94160	43.787
Total		16661637	100.000	215040	100.000

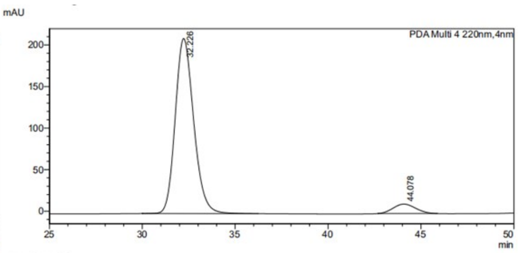

<Peak Table>

PDA Ch4 220nm

Peak#	Ret. Time	Area	Area%	Height	Height%
1	32.226	14590615	93.858	210655	94.893
2	44.078	954851	6.142	11338	5.107
Total		15545466	100.000	221993	100.000

5.7 Reduction of TPO 9.[3,6,7]

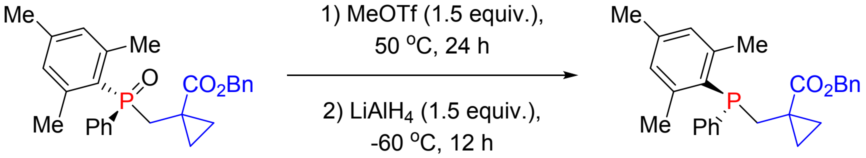

9, 52%, 88% ee **10**, 62%, 85% ee

To a stirred solution of alkylating agent MeOTf (1.2 mmol) in DME (1.0 mL), TPO **9** (0.1 mmol) disolved in DME (1.0 mL) was added dropwise at room temperature under a nitrogen atmosphere. The reaction mixture was warmed to 50 °C and stirred for 24 h. The flask was then immersed in a -60 ºC bath. A solution of lithium aluminum hydride in tetrahydrofuran (1.0 M, 0.25 mmol, 2.5 equiv) cooled to -60 °C was transferred to the reaction flask.the mixture was stirred for 12 h at -60 ºC. The reaction wasthen allowed to warm to 0 ºC over 2 h. The reaction mixture was washed with deionised water (5 mL),and the isolated organic layer was dried over anhydrous Na_2SO_4. The drying agent was removed by filtration, and the solvent was removed in vacuo to give colourless oil. The residue was purified by column chromatography on silica gel (petroleum ether/ethyl acetate = 10:1) to afford **10** (62%, 85% *ee*). $[\alpha]^{22}_D$ = 0.26 (*c* 0.5, $CHCl_3$); ^{1}H NMR (400 MHz, $CDCl_3$) δ 7.35 – 7.27 (m, 5H), 7.24 – 7.17 (m, 4H), 7.16 – 7.12 (m, 1H), 6.87 (d, *J* = 1.9 Hz, 2H), 5.13 (d, *J* = 12.5 Hz, 1H), 5.02 (d, *J* = 12.5 Hz, 1H), 2.83 (d, *J* = 15.2 Hz, 1H), 2.34 – 2.29 (m, 1H), 2.28 (s, 9H), 1.35 (d, *J* = 2.5 Hz, 1H), 0.96 – 0.91 (m, 1H), 0.91 – 0.85 (m, 1H), 0.77 – 0.73 (m, 1H). ^{31}P NMR (162 MHz, $CDCl_3$) δ -28.55. ^{13}C NMR (101 MHz, $CDCl_3$) δ 174.85 (d, *J* = 1.3 Hz), 144.82 (d, *J* = 15.9 Hz), 141.60 (d, *J* = 15.3 Hz), 139.51 (d, *J* = 1.4 Hz), 136.05, 129.65 (d, *J* = 4.4 Hz), 128.90 (d, *J* = 15.4 Hz), 128.41, 128.21 (d, *J* = 33.0 Hz), 128.17 (d, *J* = 3.6 Hz), 127.97, 127.95, 126.11 (d, *J* = 1.6 Hz),

66.48, 32.28 (d, J = 15.7 Hz), 23.43 (d, J = 17.9 Hz), 23.06, 21.05, 16.97 (d, J = 11.0 Hz), 15.97 (d, J = 10.4 Hz). Enantiomeric excess: 85%, determined by HPLC (Chiralpak ID, hexane/*i*-PrOH = 95/5; flow rate 0.8 ml/min; 25 °C; 210 nm), first peak: t_R = 6.6 min, second peak: t_R = 8.0 min. HRMS (ESI) calcd. For $C_{27}H_{29}NaO_2P$ $[M+Na]^+$: 439.1973, found: 439.1973.

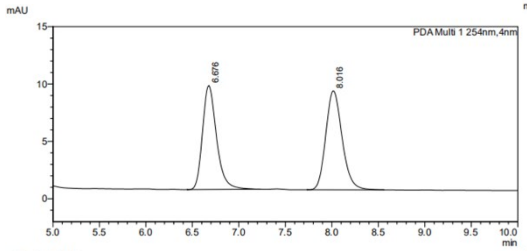

<Peak Table>
PDA Ch1 254nm

Peak#	Ret. Time	Area	Area%	Height	Height%
1	6.676	95196	47.468	9063	51.258
2	8.016	105349	52.532	8618	48.742
Total		200545	100.000	17680	100.000

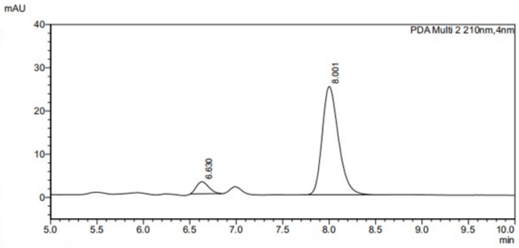

<Peak Table>
PDA Ch2 210nm

Peak#	Ret. Time	Area	Area%	Height	Height%
1	6.630	25387	7.669	2767	9.929
2	8.001	305622	92.331	25102	90.071
Total		331009	100.000	27869	100.000

5.8 Base-promoted alkylation of chiral SPO 1a.[3]

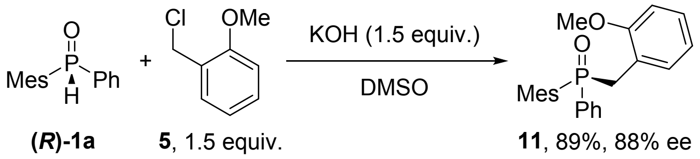

(*R*)-1a 0.2 mmol **5**, 1.5 equiv. **11**, 89%, 88% ee

The experimental procedure was the same as **5.2**. The pure compound **11** was obtained as a white solid (89%, 88% ee) from flash chromatography (silica gel, petroleum ether/ethyl acetate = 1/1 as eluent). $[\alpha]^{22}_D$ = -0.09 (c 0.5, $CHCl_3$); ^1H NMR (400 MHz, $CDCl_3$) δ 7.57 – 7.51 (m, 2H), 7.49 – 7.39 (m, 2H), 7.38 – 7.32 (m, 2H), 7.20 – 7.11 (m, 1H), 6.89 (t, J = 7.4 Hz, 1H), 6.78 (d, J = 3.4 Hz, 2H), 6.62 (d, J = 8.2 Hz, 1H), 3.97 – 3.72 (m, 2H), 3.32 (s, 3H), 2.24 (s, 3H), 2.23 (s, 6H). ^{31}P NMR (162 MHz, $CDCl_3$) δ 34.32. ^{13}C NMR (101 MHz, $CDCl_3$) δ 157.02 (d, J = 5.4 Hz), 143.42 (d, J = 10.2 Hz), 140.91 (d, J = 2.7 Hz), 137.17 (d, J = 96.7 Hz), 132.23 (d, J = 4.7 Hz), 130.79 (d, J = 2.6 Hz), 130.50 (d, J = 11.3 Hz), 129.86 (d, J = 9.5 Hz), 128.24 (d, J = 11.6 Hz), 127.91 (d, J = 3.1 Hz), 124.95 (d, J = 96.2 Hz), 120.29 (d, J = 2.9 Hz), 120.20 (d, J = 8.2 Hz), 109.88 (d, J = 2.5 Hz), 54.61, 31.71 (d, J = 65.4 Hz), 23.28 (d, J = 3.6 Hz), 20.85 (d, J = 1.1 Hz). Enantiomeric excess: 88%, determined by HPLC (Chiralpak IC, hexane/*i*-PrOH = 70/30; flow rate 0.8 ml/min; 25 °C; 210 nm), first

peak: t_R = 44.3 min, second peak: t_R = 49.7 min. HRMS (ESI) calcd. For $C_{23}H_{25}NaO_2P$ [M+Na]$^+$: 387.1506, found: 387.1509.

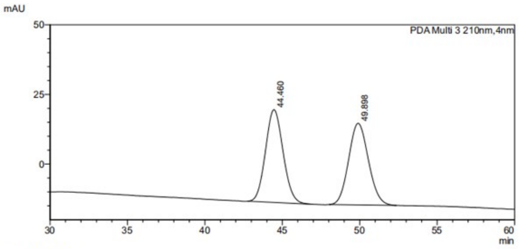

<Peak Table>

PDA Ch3 210nm

Peak#	Ret. Time	Area	Area%	Height	Height%
1	44.460	2636045	50.280	33318	53.211
2	49.898	2606683	49.720	29297	46.789
Total		5242728	100.000	62615	100.000

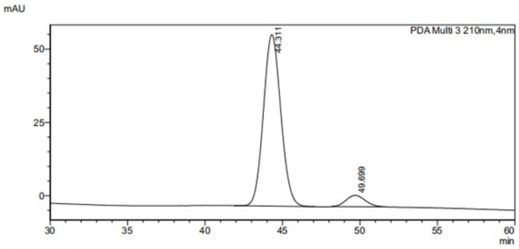

<Peak Table>

PDA Ch3 210nm

Peak#	Ret. Time	Area	Area%	Height	Height%
1	44.311	4583094	93.262	58468	93.813
2	49.699	331130	6.738	3856	6.187
Total		4914224	100.000	62324	100.000

6. The X-ray structure of compound (*R*)-1l (CCDC 1985770).

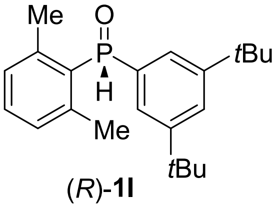

(*R*)-**1l**

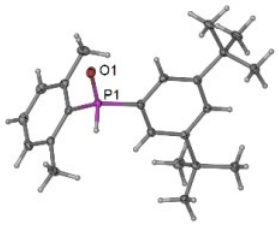

Miller array info:

D:\FRAMES\exp_923\struct\olex2_exp_923\exp_923.hkl:Iobs,SigIobs

Observation type: xray.intensity

Type of data: double, size=48625

Type of sigmas: double, size=48625

Number of Miller indices: 48625

Anomalous flag: True

Unit cell: (11.2099, 17.2004, 21.3573, 90, 98.838, 90)

Space group: C 1 2 1 (No. 5)

Systematic absences: 0

Centric reflections: 2061

Resolution range: 10.5519 0.799934

use_set_completion: True

solvent_radius: 1.20

shrink_truncation_radius: 1.20

van der Waals radii:

C	H	O	P
1.70	1.09	1.52	1.80

Total solvent accessible volume / cell = 83.2 Ang^3 [2.0%]

Total electron count / cell = 2.4

gridding: (60,90,108)

Void	#Grid points	Vol/A^3	Vol/%	Centre of mass (frac)	Eigenvectors (frac)	
1	2301	16.1	0.4	(0.024, 0.420, 0.311)	1	(0.975, 0.217, 0.045)
					2	(-0.174, 0.627, 0.759)
					3	(-0.136, 0.748, -0.650)
2	2301	16.1	0.4	(-0.024, 0.420, 0.689)	1	(0.975,-0.217, 0.045)
					2	(-0.174,-0.627, 0.759)
					3	(0.136, 0.748, 0.650)
3	1361	9.5	0.2	(-0.000, 0.411, 0.500)	1	(1.000, 0.000, -0.016)
					2	(0.000, 1.000, 0.000)
					3	(0.016, 0.000, 1.000)
4	2301	16.1	0.4	(0.476, 0.920, 0.689)	1	(0.975,-0.217, 0.045)
					2	(-0.174,-0.627, 0.759)
					3	(0.136, 0.748, 0.650)
5	1361	9.5	0.2	(0.500, 0.911, 0.500)	1	(1.000, 0.000, -0.016)

2 (0.000, 1.000,

0.000)

3 (0.016, 0.000,

1.000)

6 2301 16.1 0.4 (0.524, 0.920, 0.311) 1 (0.975, 0.217,

0.045)

2 (-0.174, 0.627,

0.759)

3 (-0.136, 0.748, -

0.650)

Void	Vol/Ang^3	#Electrons
1	16.1	0.4
2	16.1	0.4
3	9.5	0.4
4	16.1	0.4
5	9.5	0.4
6	16.1	0.4

7. References:

1. Q. Dai, W. Li, Z. Li, J. Zhang, *J. Am. Chem. Soc.* **2019**, *141*, 20556.

2. A. J. Bloomfield, S. B. Herzon, *Org. Lett.* **2012**, *14*, 4371.

3. S.-Z. Nie, Z.-Y. Zhou, J.-P. Wang, H. Yan, J.-H. Wen, J.-J. Ye, Y.-Y. Cui, C.-Q. Zhao, *J. Org. Chem.* **2017**, *82*, 9425.

4. H. R. Beddoe, G. K. Andrews, V. Magné, D. J. Cuthbertson, J. Saska, L. A. Shannon-Little, E. S. Shanahan, F. H. Sneddon, M. R. Denton, *Science* **2019**, *365*, 910.

5. T. N. Tzvetkov, T. Arndtb, J. Mattay, *Tetrahedron* **2007**, *63*, 10497.

6. K. V. Rajendran, D. G. Gilheany, *Chem. Commun*. **2012**, *48*, 817.

7. X.-T. Liu, Y.-Q. Zhang, X.-Y. Han, S.-P. Sun, Q.-W. Zhang, *J. Am. Chem. Soc.* **2019**, *141*, 16584.

8. NMR spectra:

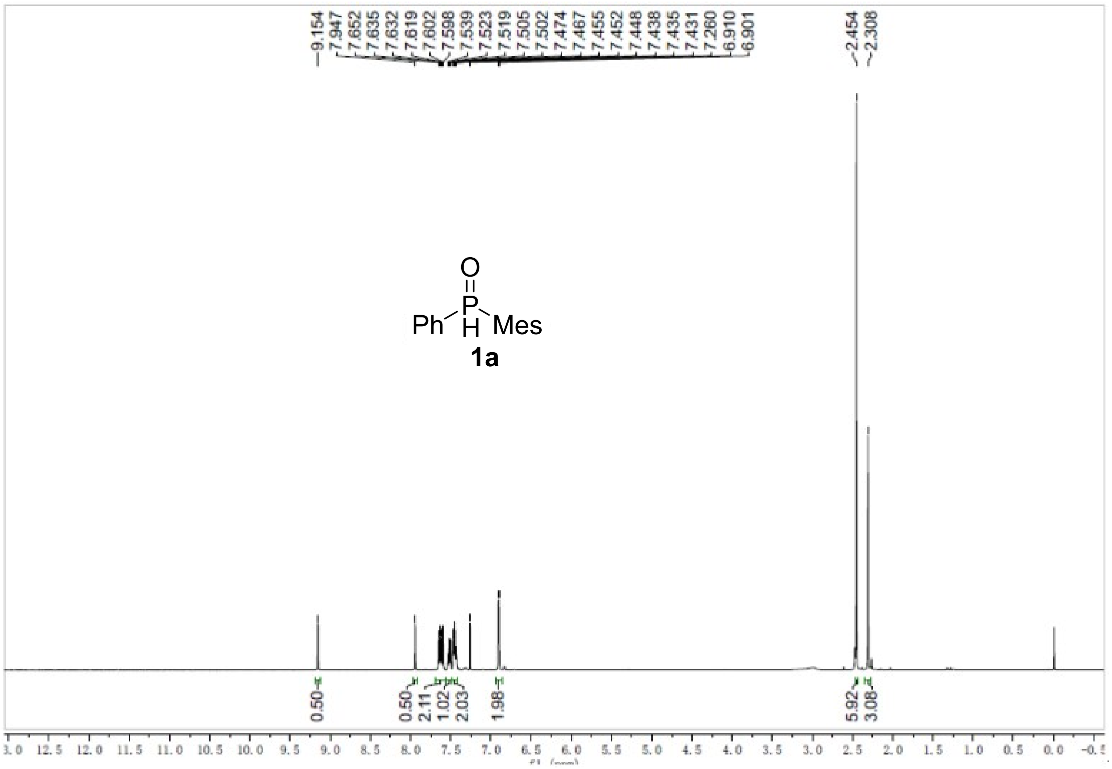

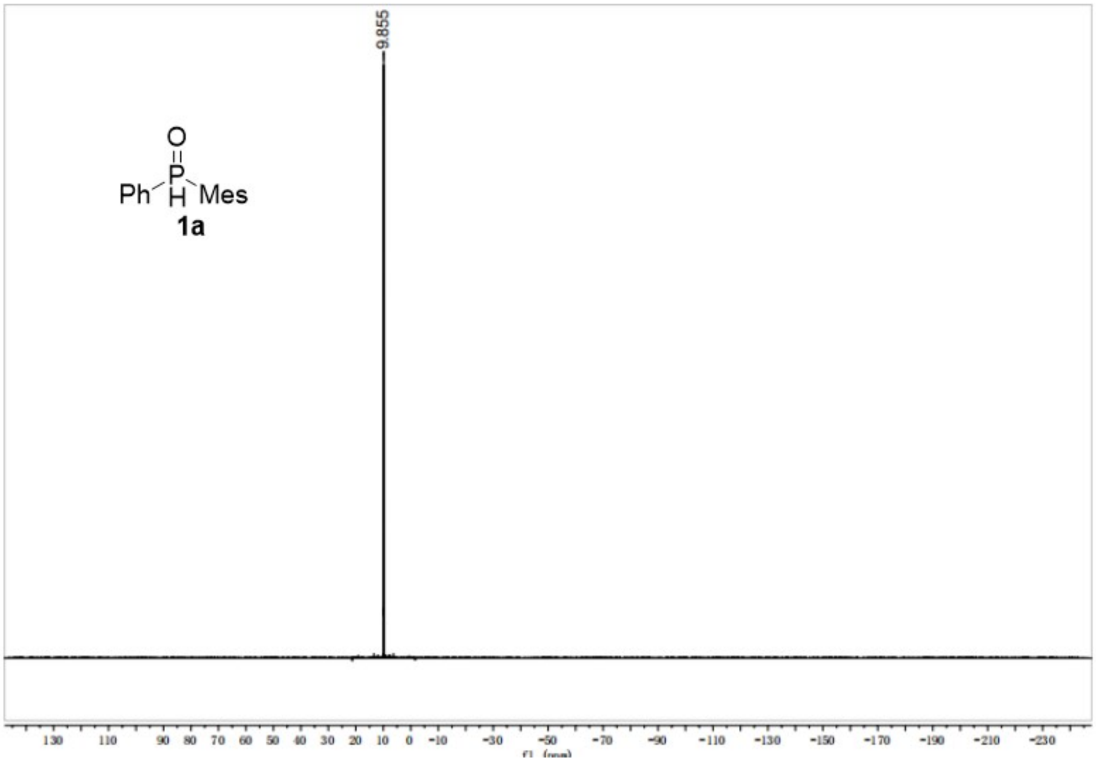

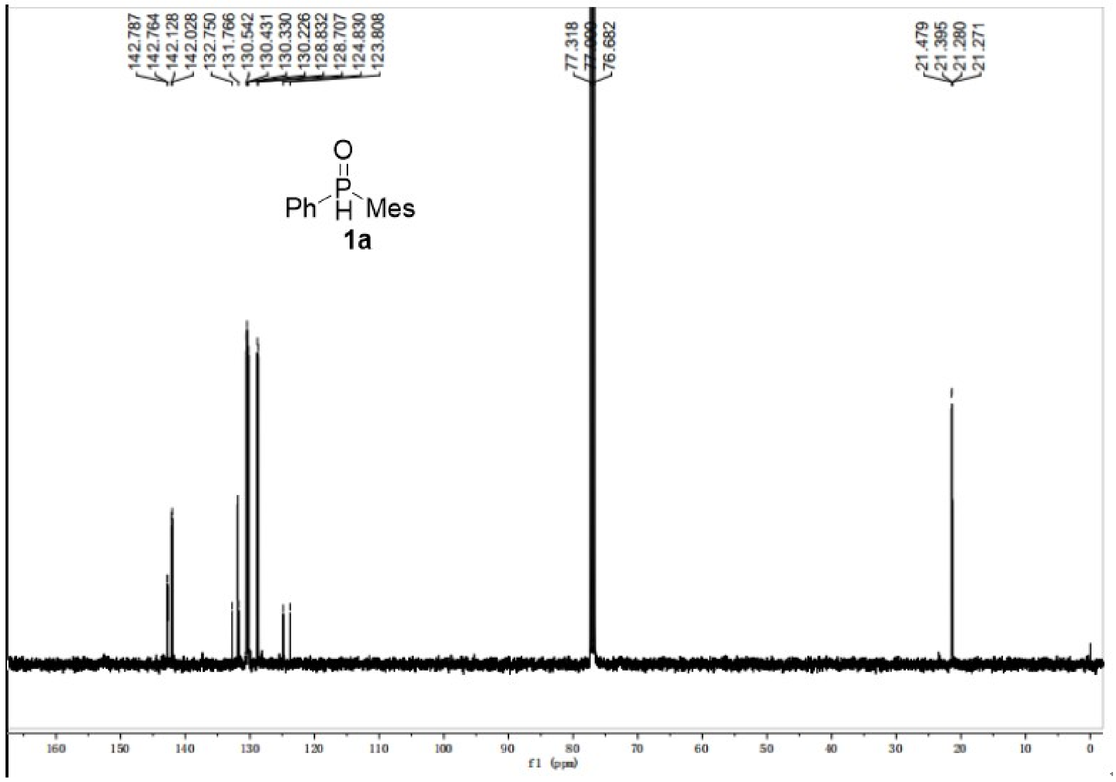
Ph
Mes
1a
f1 (ppm)

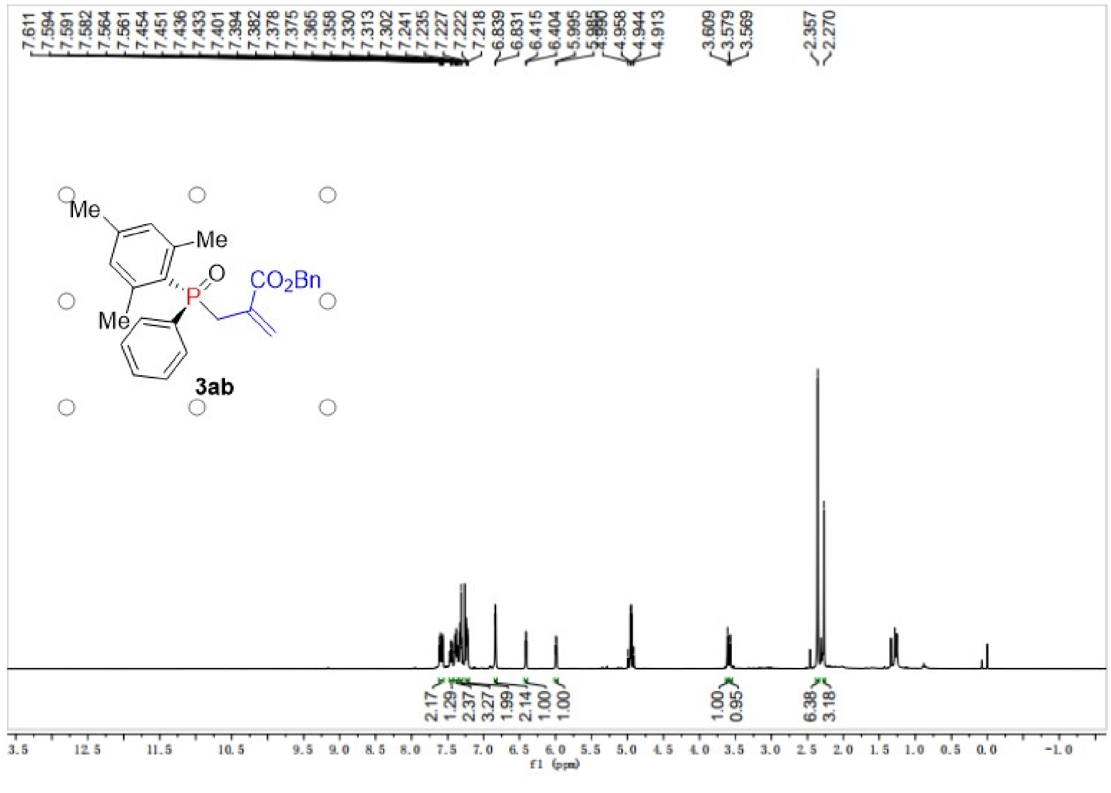
Me
Me
Me
CO2Bn
3ab
f1 (ppm)

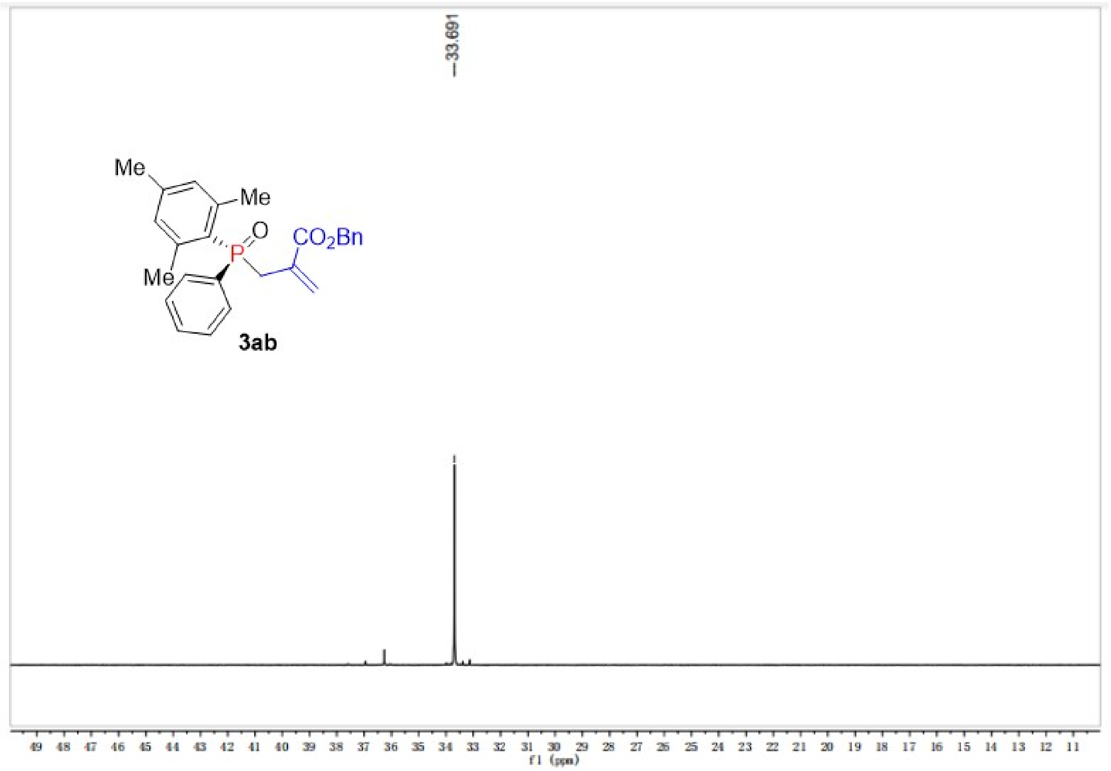

—33.691
Me
Me
Me
O
P
CO2Bn
3ab
f1 (ppm)

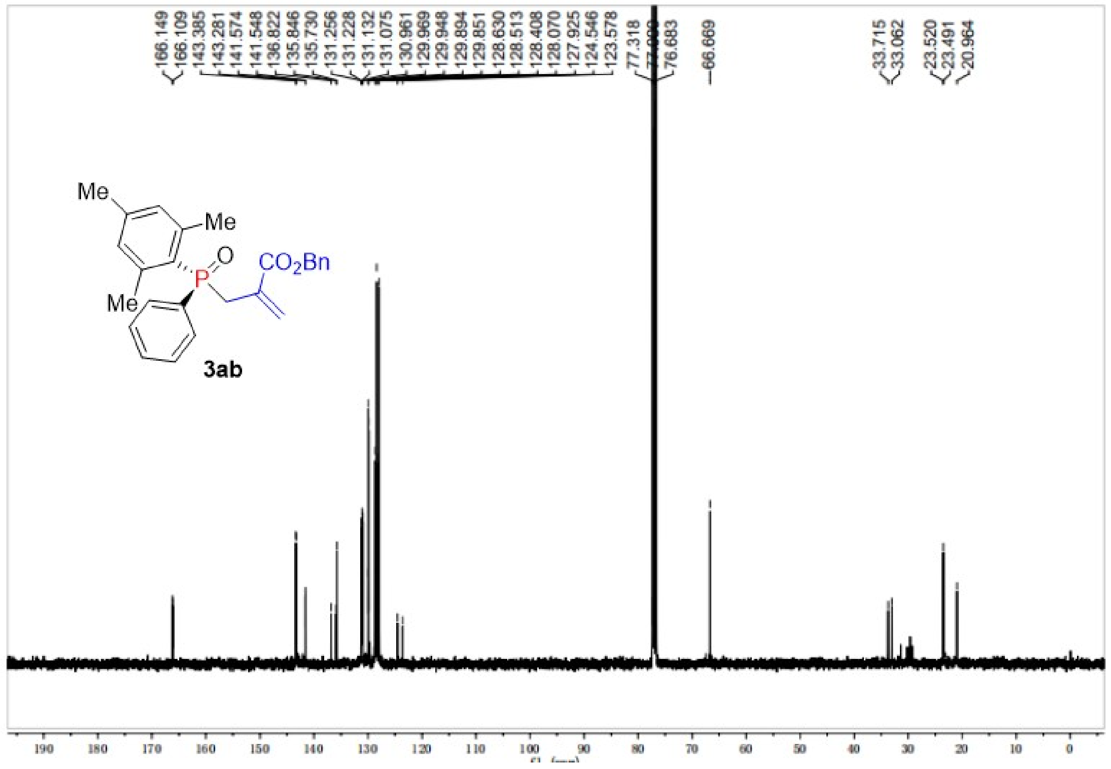

166.149
166.109
143.385
143.281
141.574
141.548
136.822
135.846
135.730
131.256
131.228
131.132
131.075
130.961
129.969
129.948
129.894
129.851
128.630
128.513
128.408
128.070
127.925
124.546
123.578
77.318
76.683
66.669
33.715
33.062
23.520
23.491
20.964
Me
Me
Me
O
P
CO2Bn
3ab

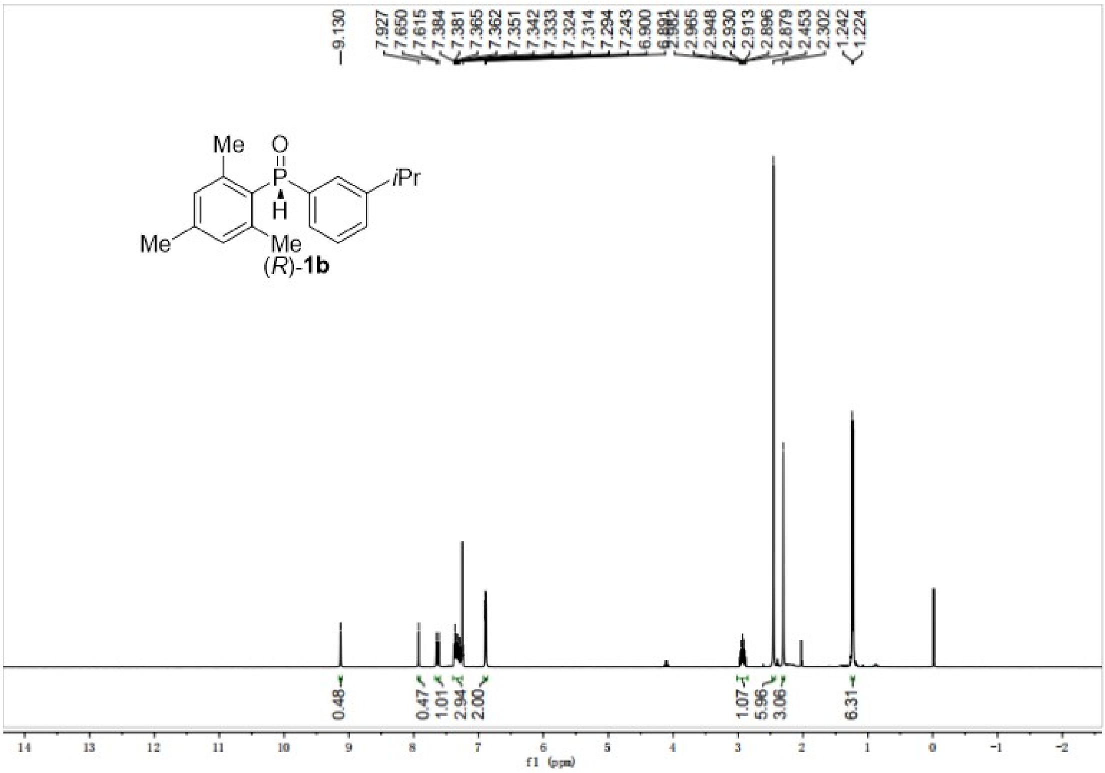

9.130
7.927
7.650
7.615
7.384
7.381
7.365
7.362
7.351
7.342
7.333
7.324
7.314
7.294
7.243
6.900
2.965
2.948
2.930
2.913
2.896
2.879
2.453
2.302
1.242
1.224
Me
O
P
H
iPr
Me
Me
(R)-1b
0.48
0.47
1.01
2.94
2.00
1.07
5.96
3.06
6.31
f1 (ppm)

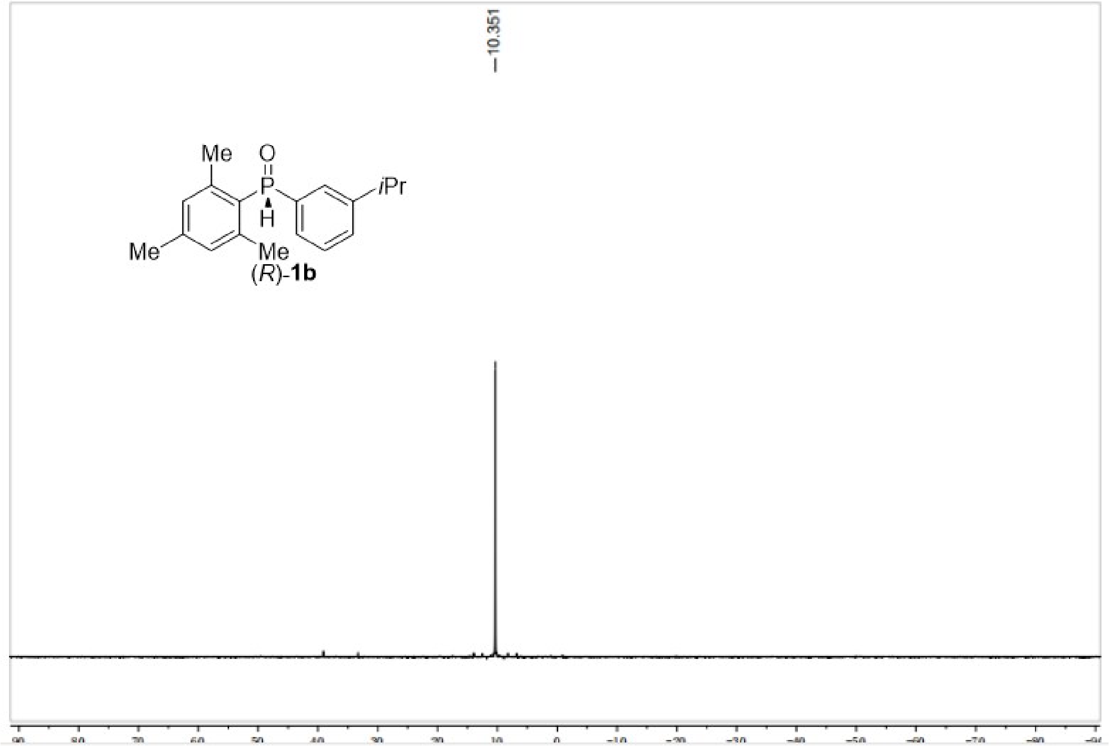

10.351
Me
O
P
H
iPr
Me
Me
(R)-1b

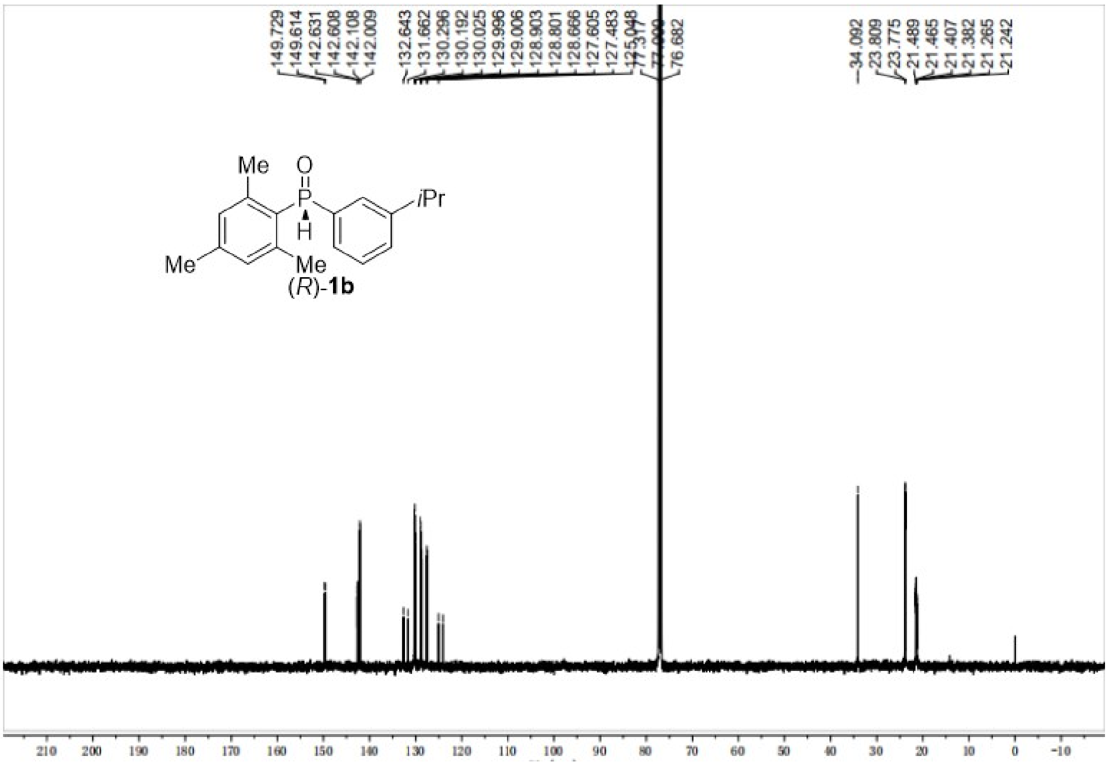
149.729
149.614
142.631
142.608
142.108
142.009
132.643
131.662
130.296
130.192
130.025
129.996
129.006
128.903
128.801
128.666
127.605
127.483
77.317
76.682
34.092
23.809
23.775
21.489
21.465
21.407
21.382
21.265
21.242
Me
O
P
H
iPr
Me
Me
(R)-1b
210
200
190
180
170
160
150
140
130
120
110
100
90
80
70
60
50
40
30
20
10
0
-10

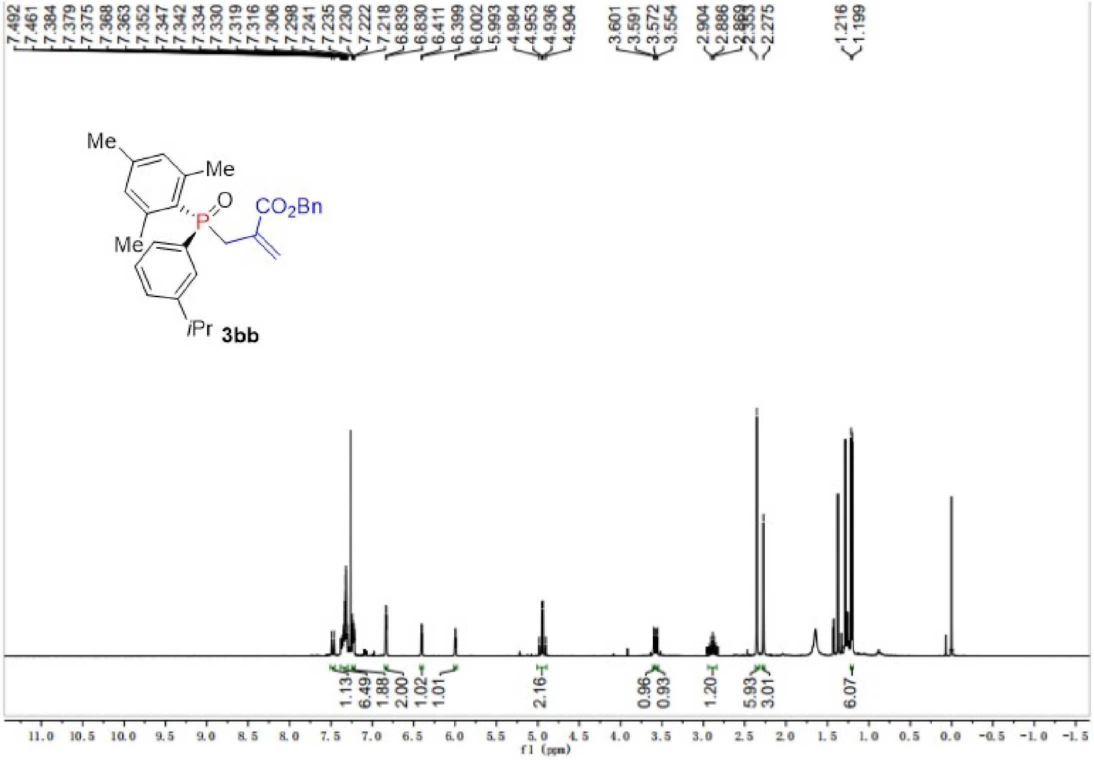
7.492
7.461
7.384
7.379
7.375
7.368
7.363
7.352
7.347
7.342
7.334
7.330
7.319
7.316
7.306
7.298
7.241
7.235
7.230
7.222
7.218
6.839
6.830
6.411
6.399
6.002
5.993
4.984
4.953
4.936
4.904
3.601
3.591
3.572
3.554
2.904
2.886
2.275
1.216
1.199
Me
Me
O
P
CO2Bn
Me
iPr
3bb
1.13
6.49
1.88
2.00
1.02
1.01
2.16
0.96
0.93
1.20
5.93
3.01
6.07
11.0
10.5
10.0
9.5
9.0
8.5
8.0
7.5
7.0
6.5
6.0
5.5
5.0
4.5
4.0
3.5
3.0
2.5
2.0
1.5
1.0
0.5
0.0
-0.5
-1.0
-1.5
f1 (ppm)

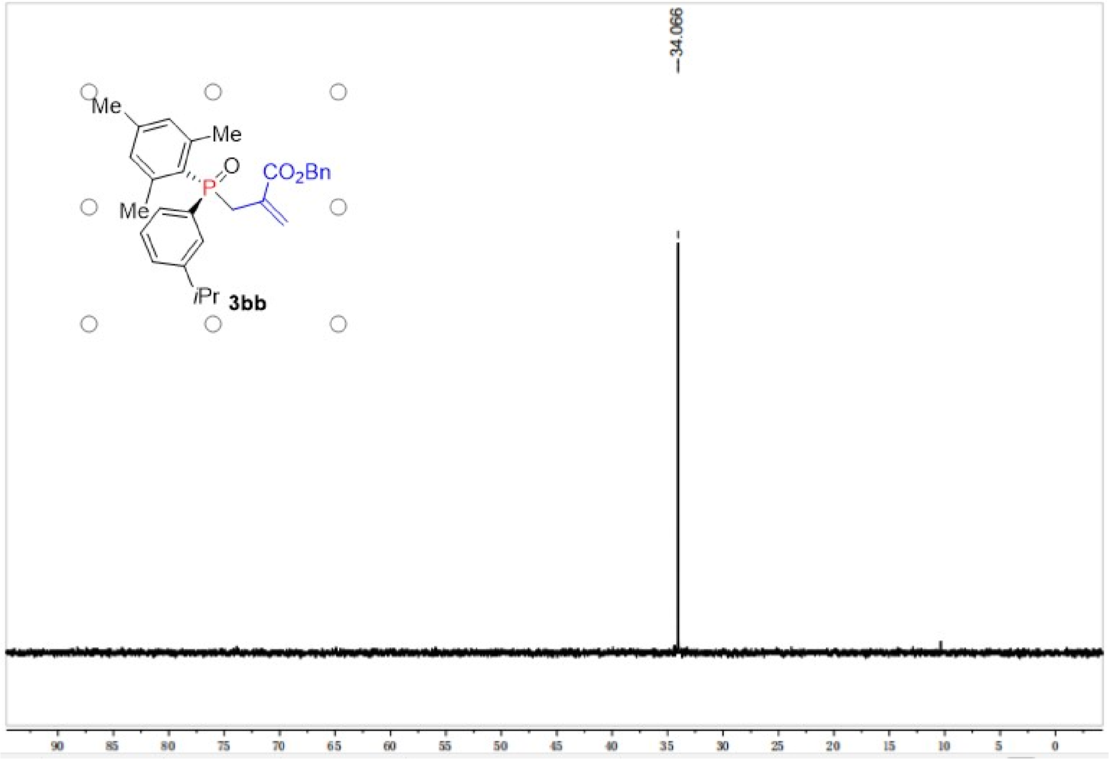

34.066
Me
Me
Me
O
P
CO_2Bn
*i*Pr
3bb
90
85
80
75
70
65
60
55
50
45
40
35
30
25
20
15
10
5
0

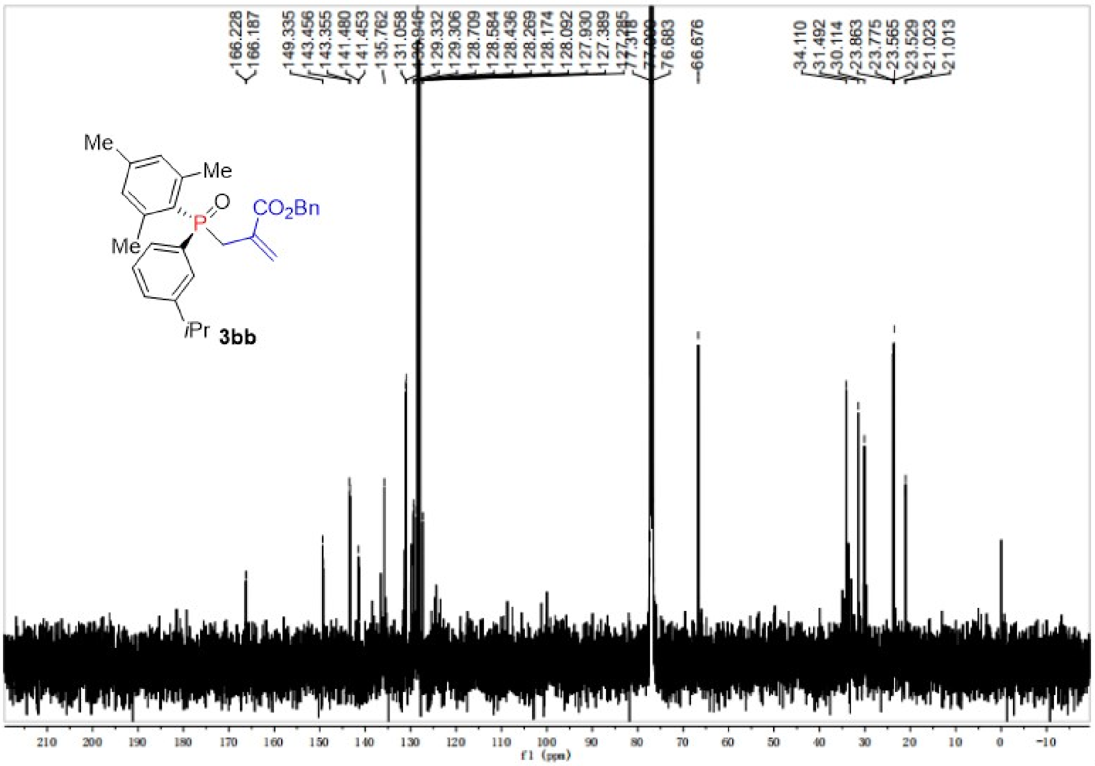

166.228
166.187
149.335
143.456
143.355
141.480
141.453
135.762
131.058
129.332
129.306
128.709
128.584
128.436
128.269
128.174
128.092
127.930
127.389
77.318
76.683
66.676
34.110
31.492
30.114
23.863
23.775
23.565
23.529
21.023
21.013
Me
Me
Me
O
P
CO_2Bn
*i*Pr
3bb
f1 (ppm)

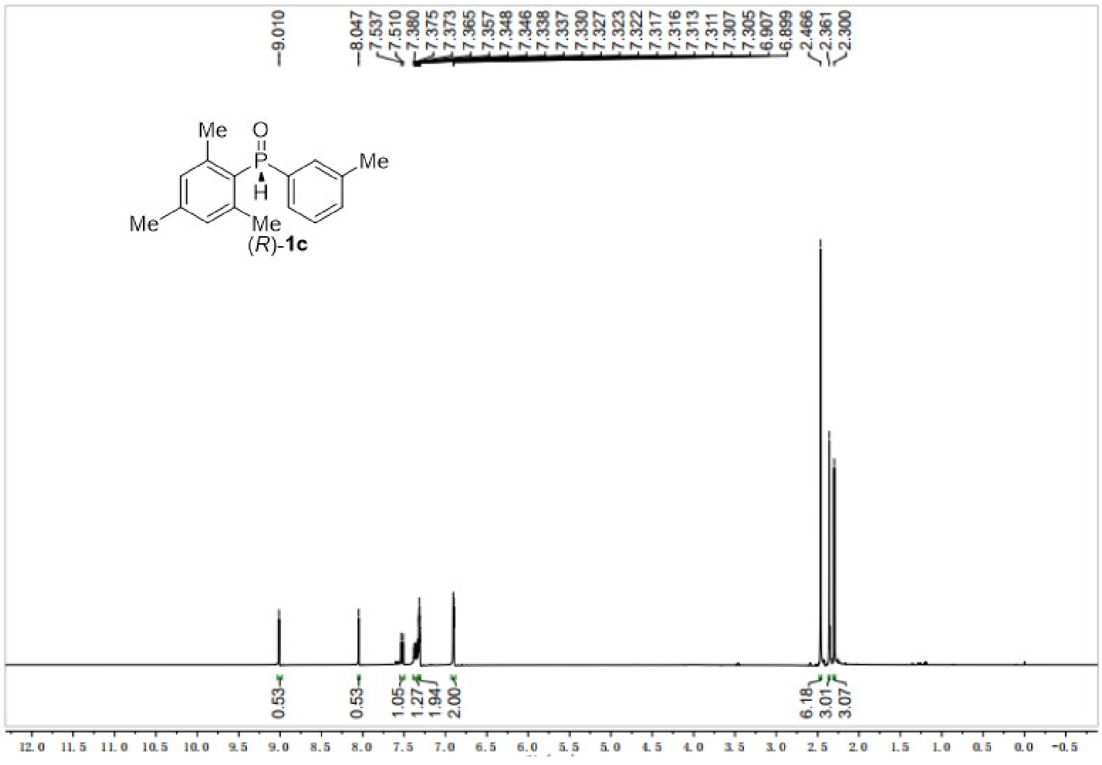
(R)-1c
Me
O
P
H
Me
Me
Me
9.010
8.047
7.537
7.510
7.380
7.375
7.373
7.365
7.357
7.348
7.346
7.338
7.337
7.330
7.327
7.323
7.322
7.317
7.316
7.313
7.311
7.307
7.305
6.907
6.899
2.466
2.361
2.300
0.53
0.53
1.05
1.27
1.94
2.00
6.18
3.01
3.07

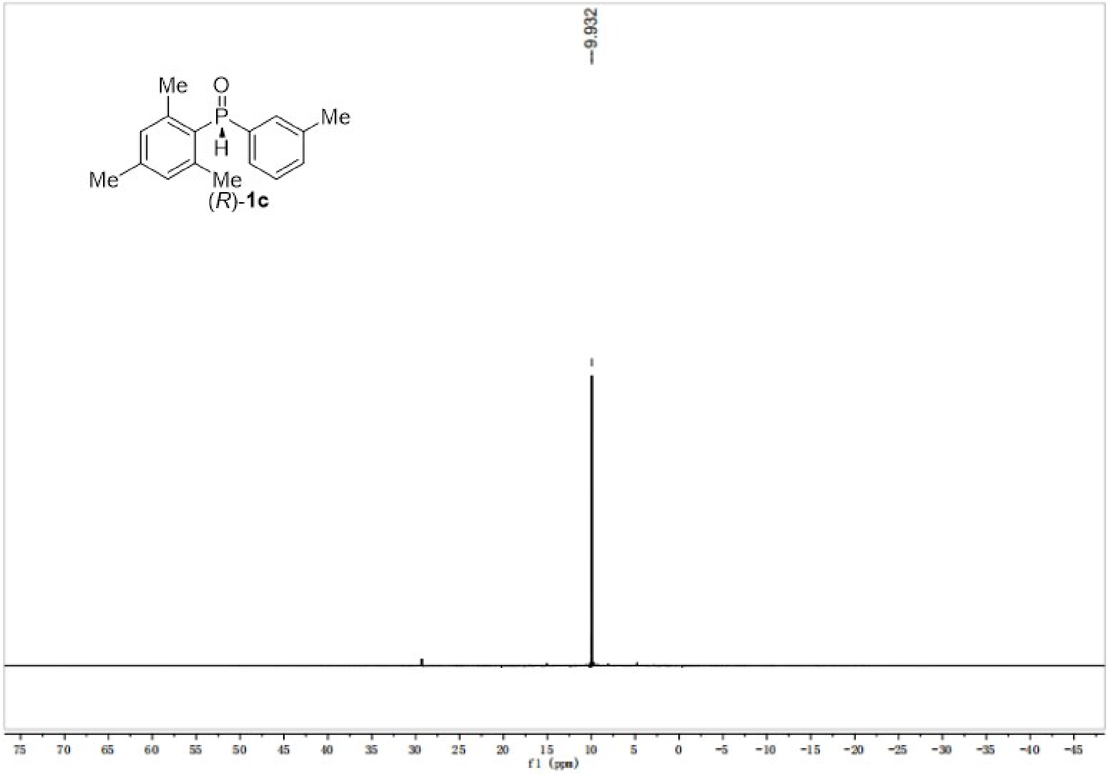
(R)-1c
Me
O
P
H
Me
Me
Me
9.932
f1 (ppm)

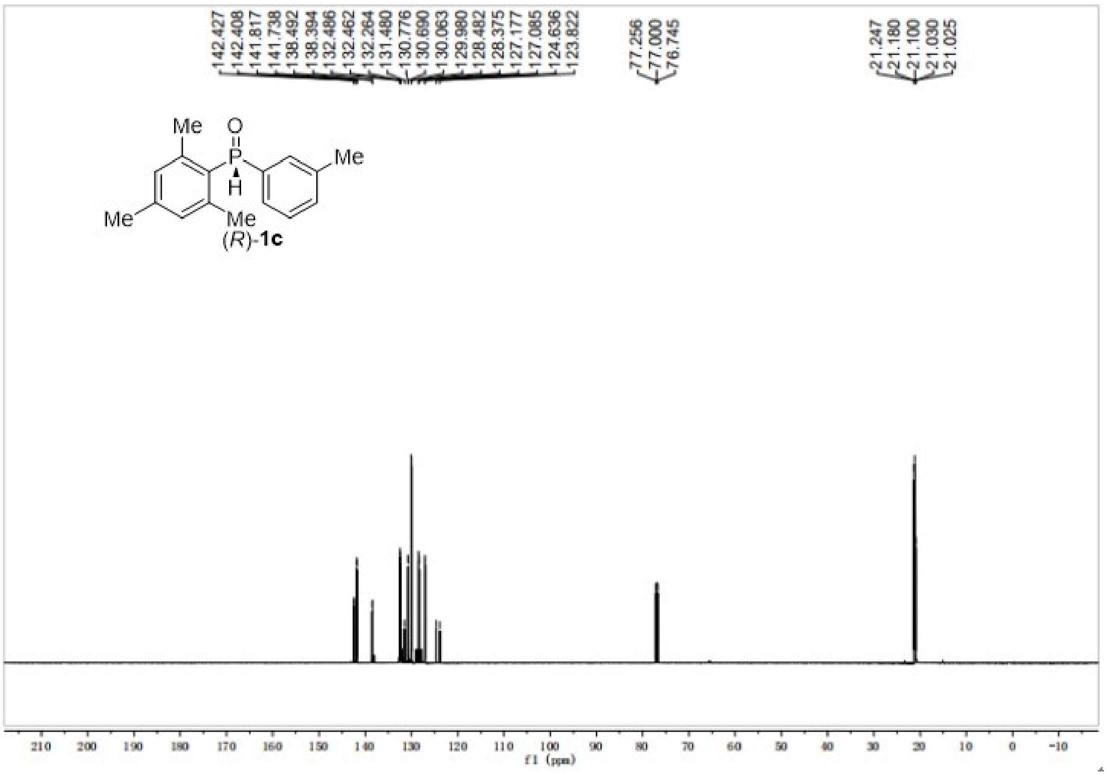
142.427
142.408
141.817
141.738
138.492
138.394
132.486
132.462
132.264
131.480
130.776
130.690
130.063
129.980
128.482
128.375
127.177
127.085
124.636
123.822
77.256
77.000
76.745
21.247
21.180
21.100
21.030
21.025
(R)-1c
f1 (ppm)

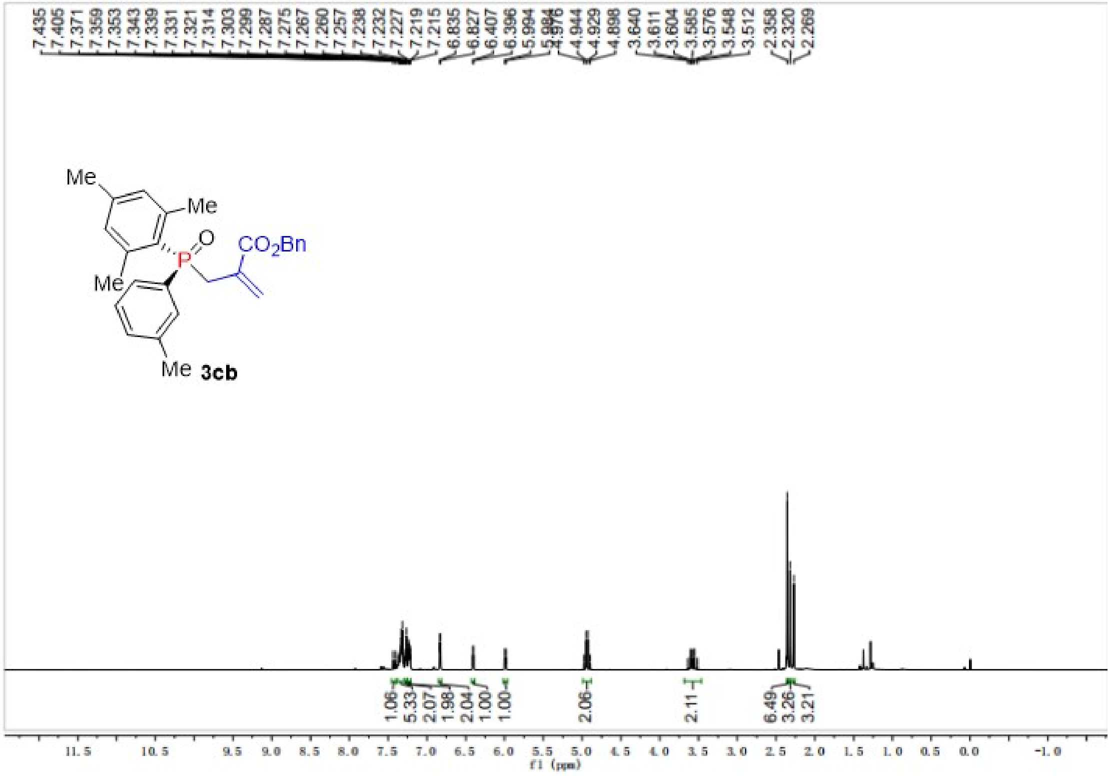
7.435
7.405
7.371
7.359
7.353
7.343
7.339
7.331
7.321
7.314
7.303
7.299
7.287
7.275
7.267
7.260
7.257
7.238
7.232
7.227
7.219
7.215
6.835
6.827
6.407
6.396
5.994
5.984
4.976
4.944
4.929
4.898
3.640
3.611
3.604
3.585
3.576
3.548
3.512
2.358
2.320
2.269
CO2Bn
3cb
1.06
5.33
2.07
1.98
2.04
1.00
1.00
2.06
2.11
6.49
3.26
3.21
f1 (ppm)

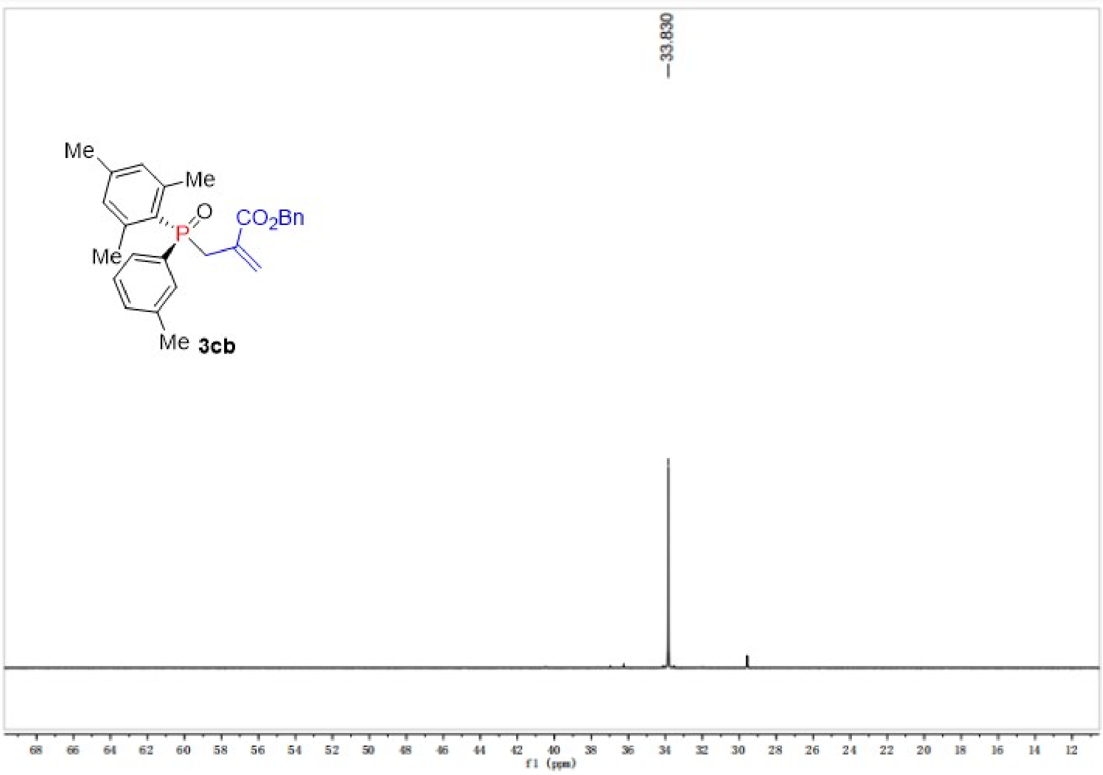

—33.830
Me
Me
O
P
CO2Bn
Me
Me
3cb
68 66 64 62 60 58 56 54 52 50 48 46 44 42 40 38 36 34 32 30 28 26 24 22 20 18 16 14 12
f1 (ppm)

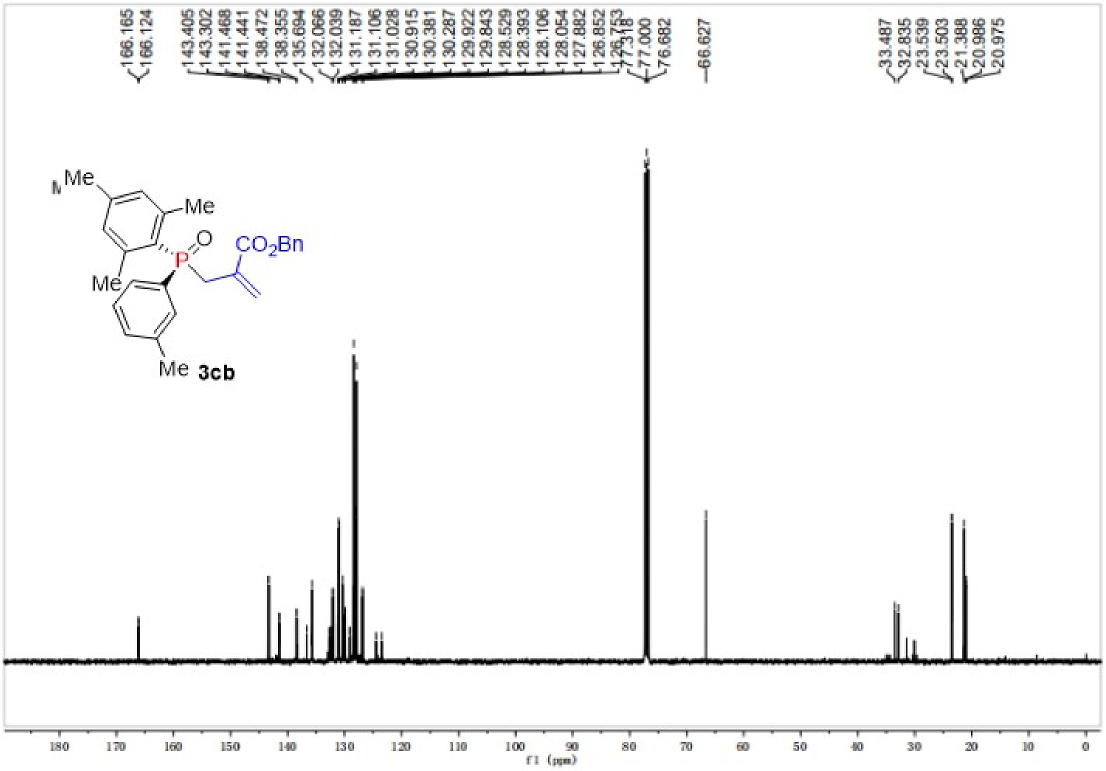

166.165
166.124
143.405
143.302
141.468
141.441
138.472
138.355
135.694
132.066
132.039
131.187
131.106
131.028
130.915
130.381
130.287
129.922
129.843
128.529
128.393
128.106
128.054
127.882
126.852
126.753
77.318
77.000
76.682
66.627
33.487
32.835
23.539
23.503
21.388
20.986
20.975
Me
Me
O
P
CO2Bn
Me
Me
3cb
180 170 160 150 140 130 120 110 100 90 80 70 60 50 40 30 20 10 0
f1 (ppm)

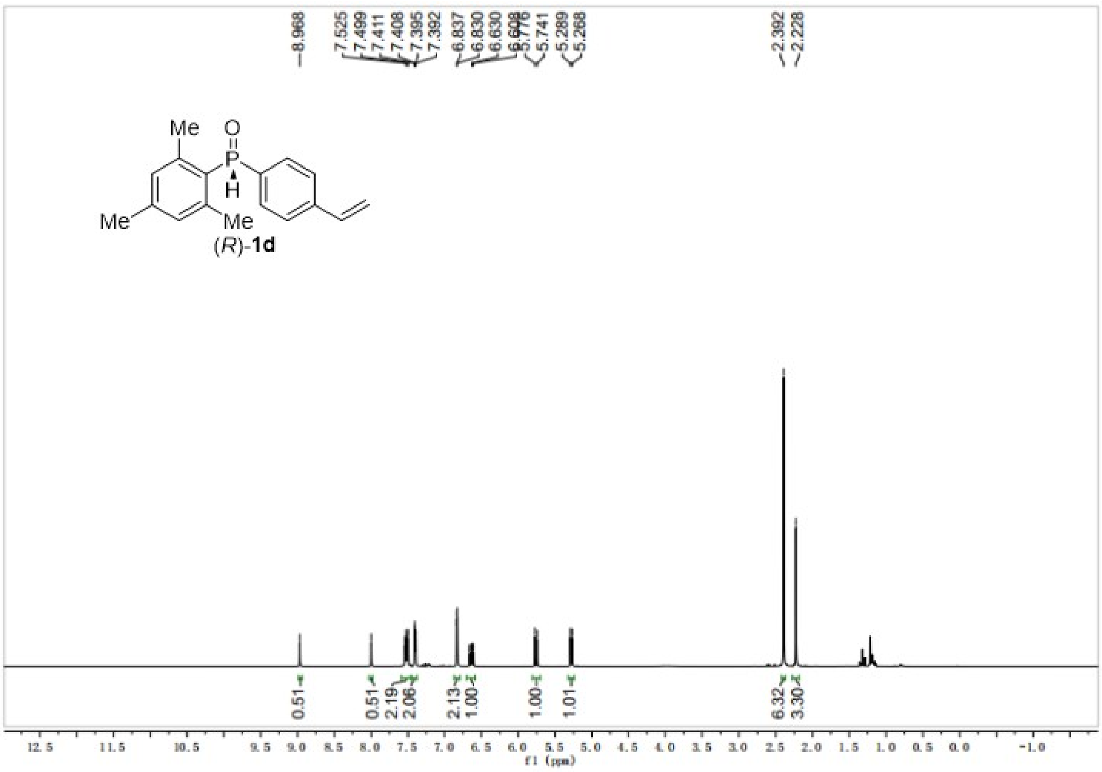

(R)-1d
f1 (ppm)

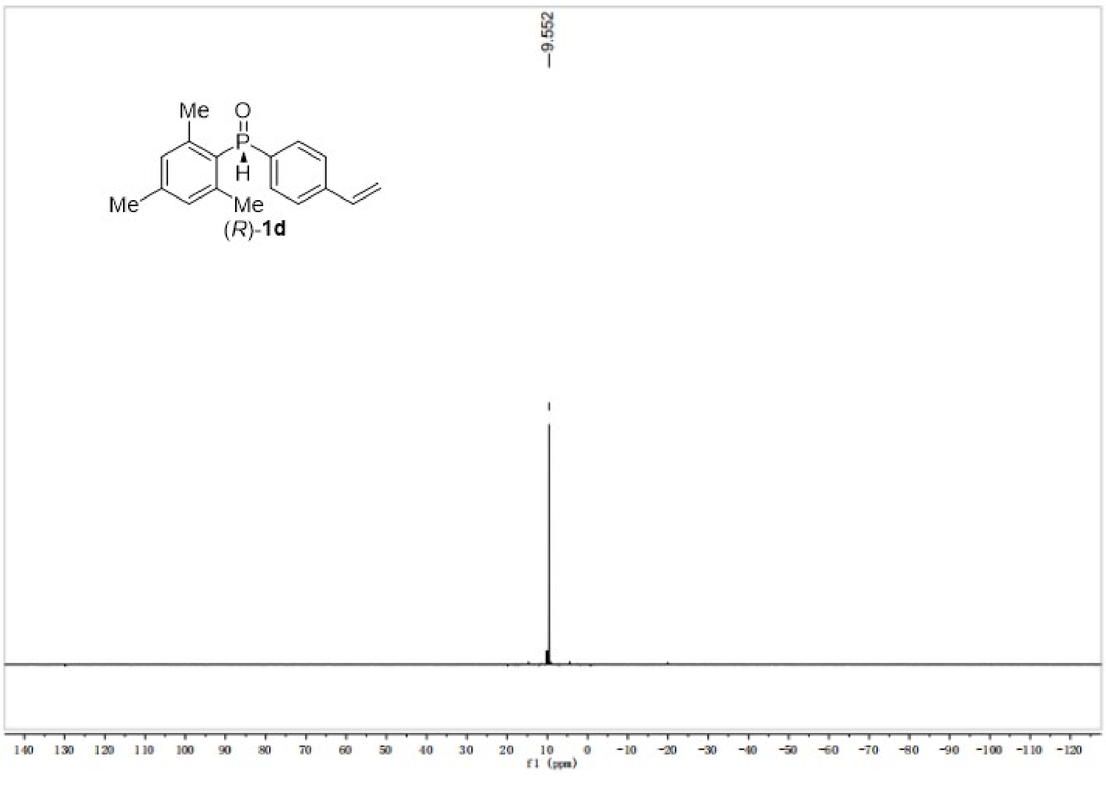

(R)-1d
f1 (ppm)

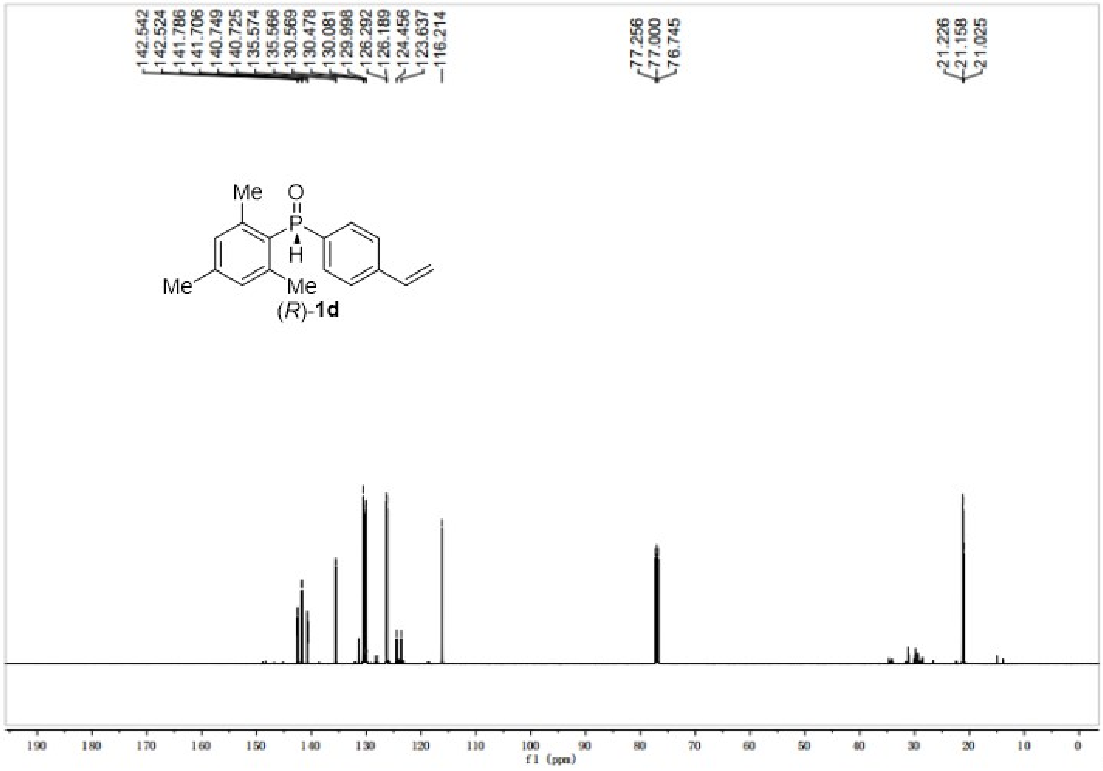
142.542
142.524
141.786
141.706
140.749
140.725
135.574
135.566
130.569
130.478
130.081
129.998
126.292
126.189
124.456
123.637
116.214
77.256
77.000
76.745
21.226
21.158
21.025
Me
O
P
H
Me
Me
(R)-1d
f1 (ppm)

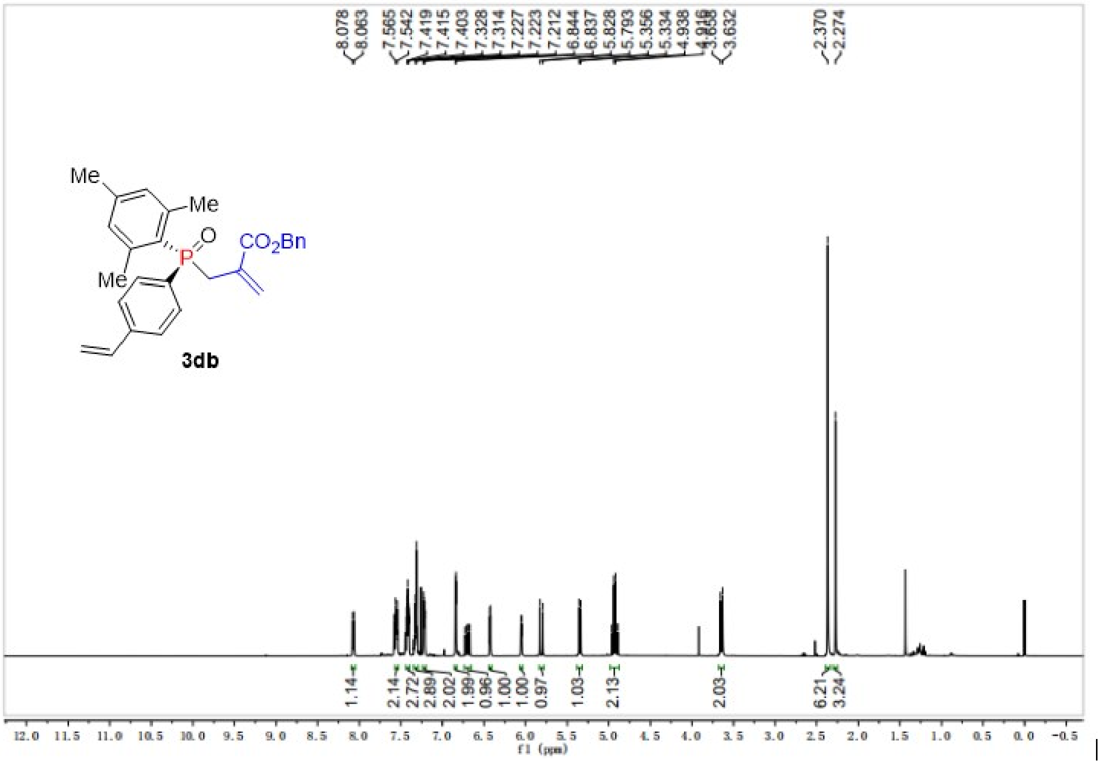
8.078
8.063
7.565
7.542
7.419
7.415
7.403
7.328
7.314
7.227
7.223
7.212
6.844
6.837
5.828
5.793
5.356
5.334
4.938
3.632
2.370
2.274
Me
Me
O
CO2Bn
Me
P
3db
f1 (ppm)

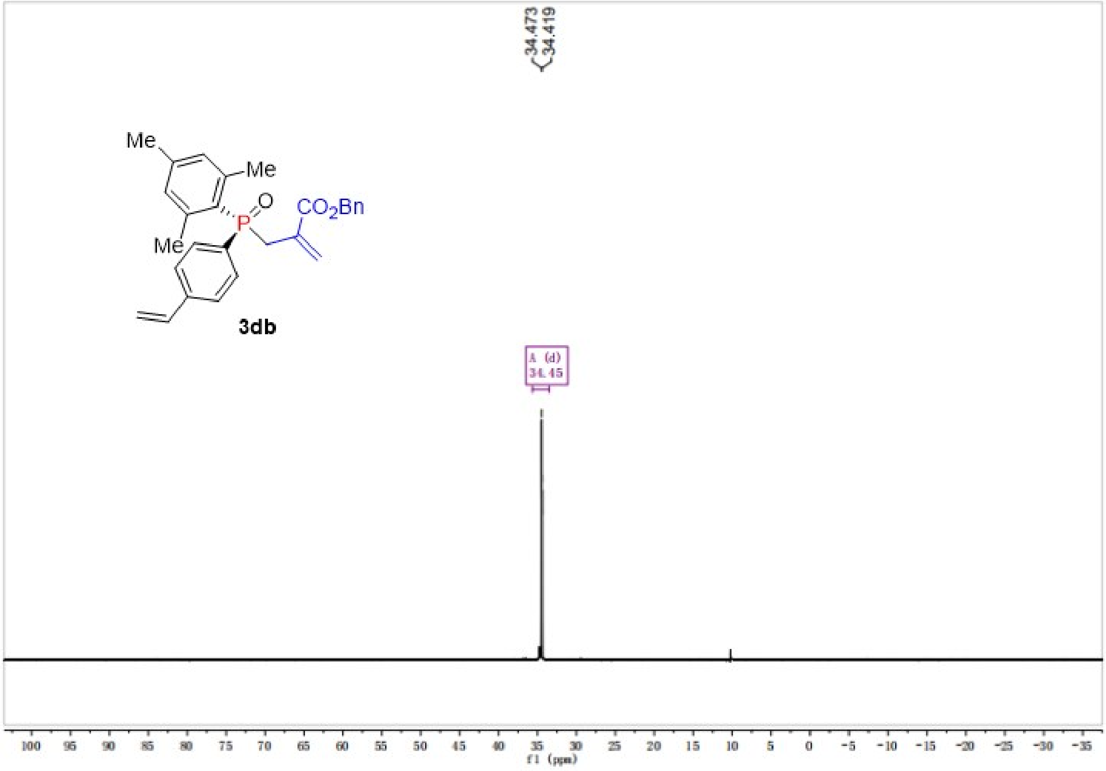

34.473
34.419
Me
Me
O
CO2Bn
P
Me
3db
A (d)
34.45
f1 (ppm)

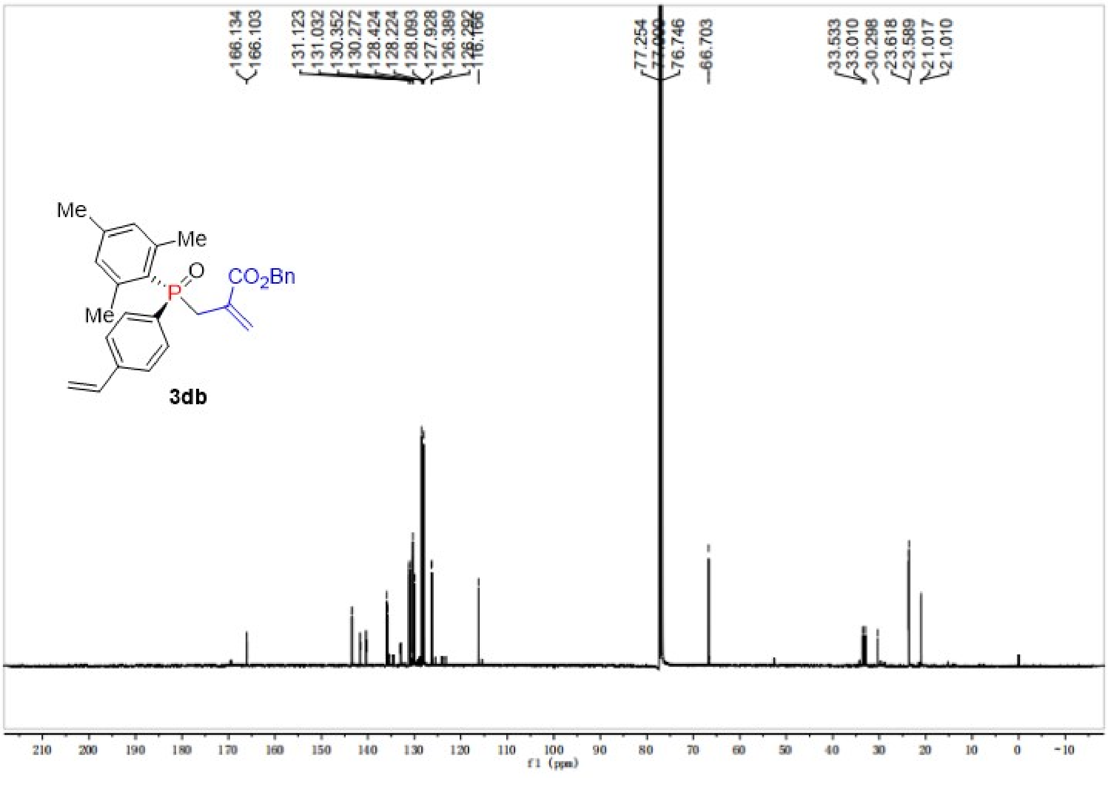

166.134
166.103
131.123
131.032
130.352
130.272
128.424
128.224
128.093
127.928
126.389
126.292
77.254
76.746
66.703
33.533
33.010
30.298
23.618
23.589
21.017
21.010
Me
Me
O
CO2Bn
P
Me
3db
f1 (ppm)

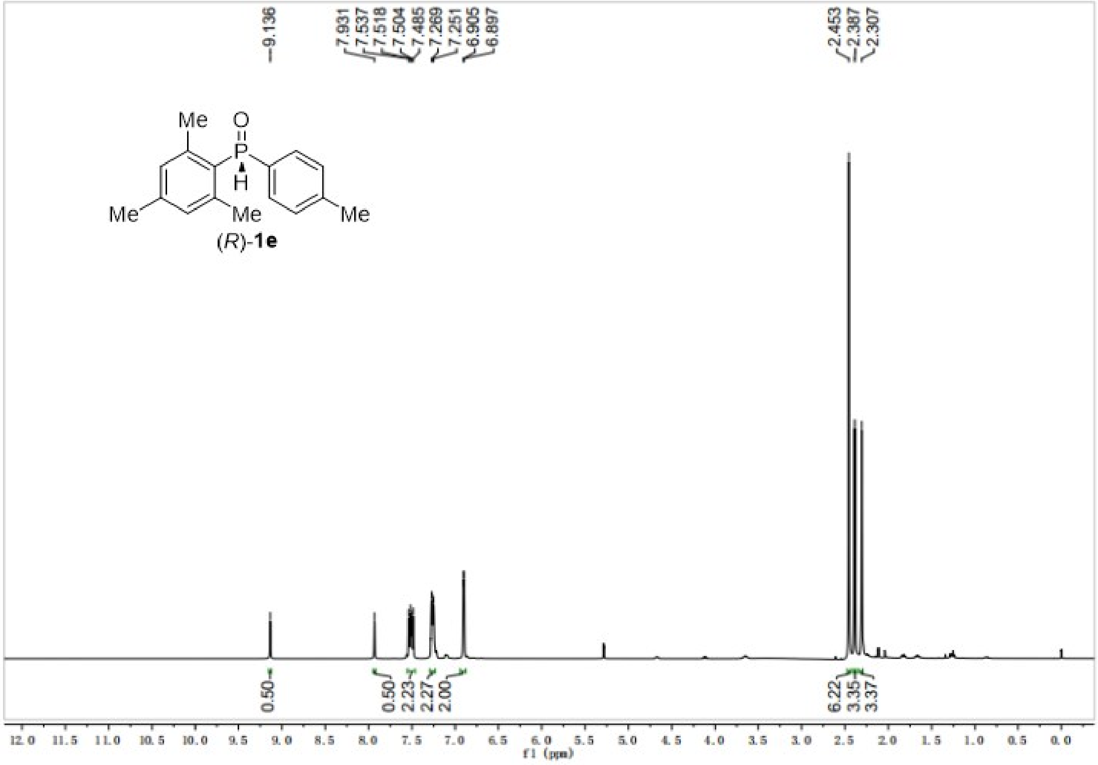

9.136
7.931
7.537
7.518
7.504
7.485
7.269
7.251
6.905
6.897
2.453
2.387
2.307
Me
O
P
H
Me
Me
Me
(R)-1e
0.50
0.50
2.23
2.27
2.00
6.22
3.35
3.37
12.0 11.5 11.0 10.5 10.0 9.5 9.0 8.5 8.0 7.5 7.0 6.5 6.0 5.5 5.0 4.5 4.0 3.5 3.0 2.5 2.0 1.5 1.0 0.5 0.0
f1 (ppm)

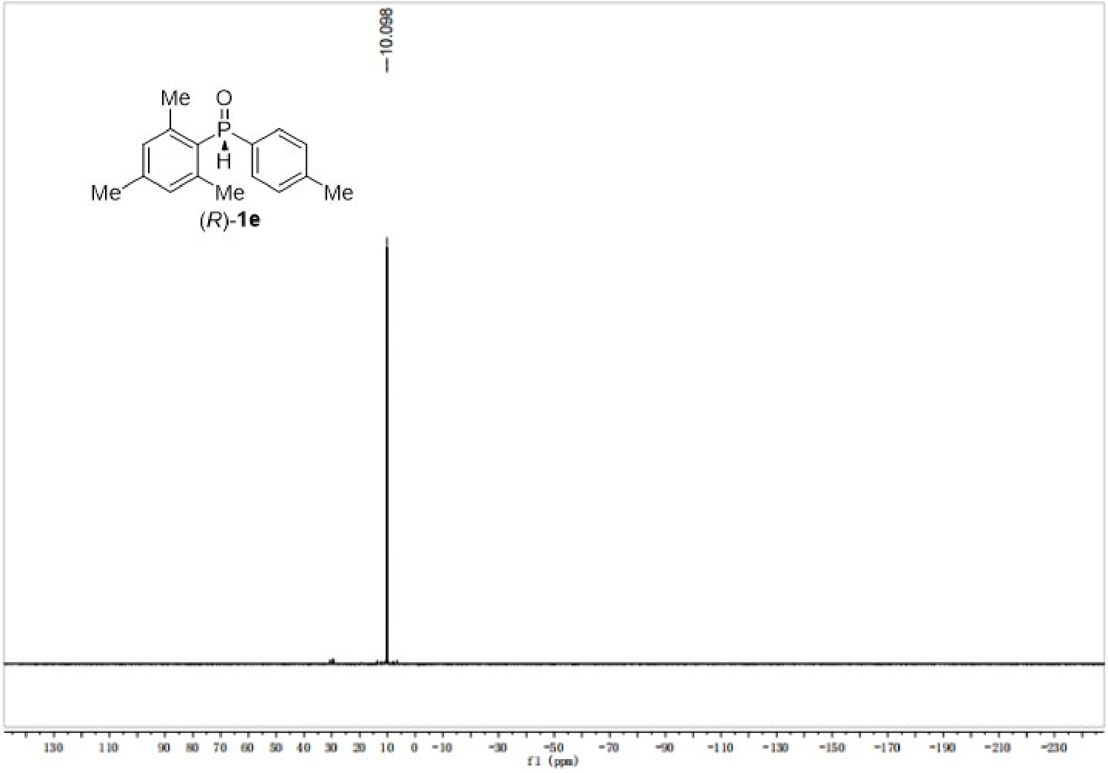

10.098
Me
O
P
H
Me
Me
Me
(R)-1e
130 110 90 80 70 60 50 40 30 20 10 0 -10 -30 -50 -70 -90 -110 -130 -150 -170 -190 -210 -230
f1 (ppm)

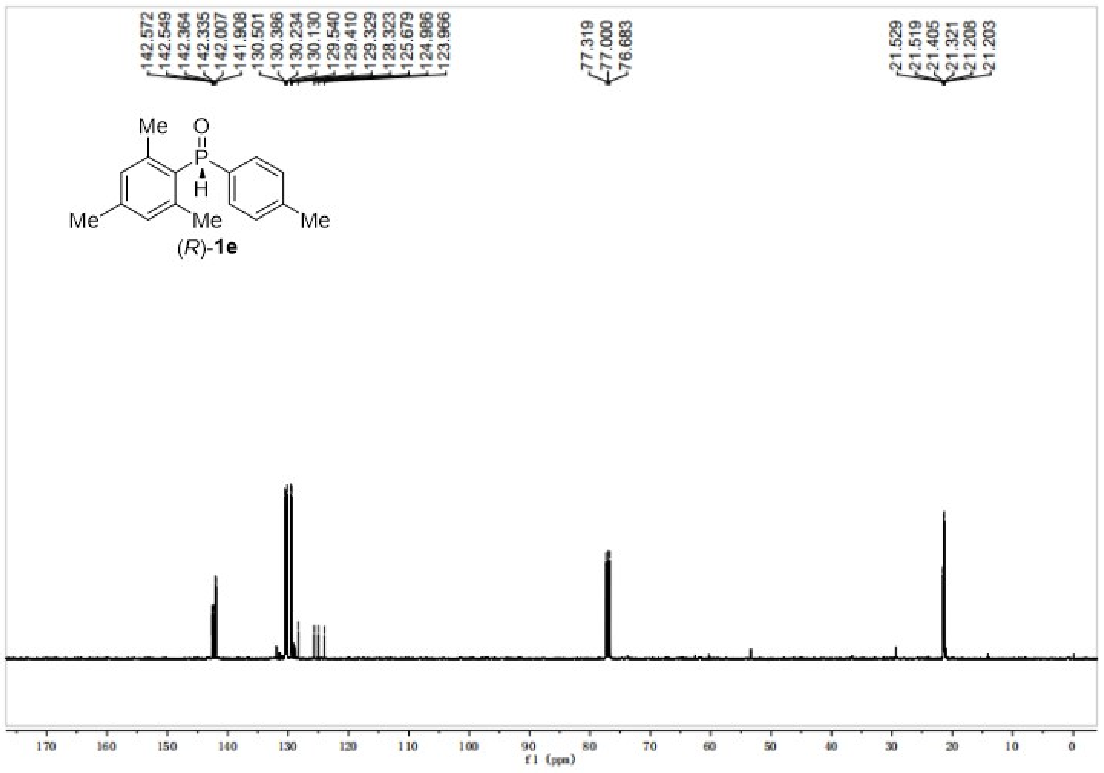
142.572
142.549
142.364
142.335
142.007
141.908
130.501
130.386
130.234
130.130
129.540
129.410
129.329
128.323
125.679
124.986
123.966
77.319
77.000
76.683
21.529
21.519
21.405
21.321
21.208
21.203
Me
O
P
H
Me
Me
Me
(R)-1e
f1 (ppm)

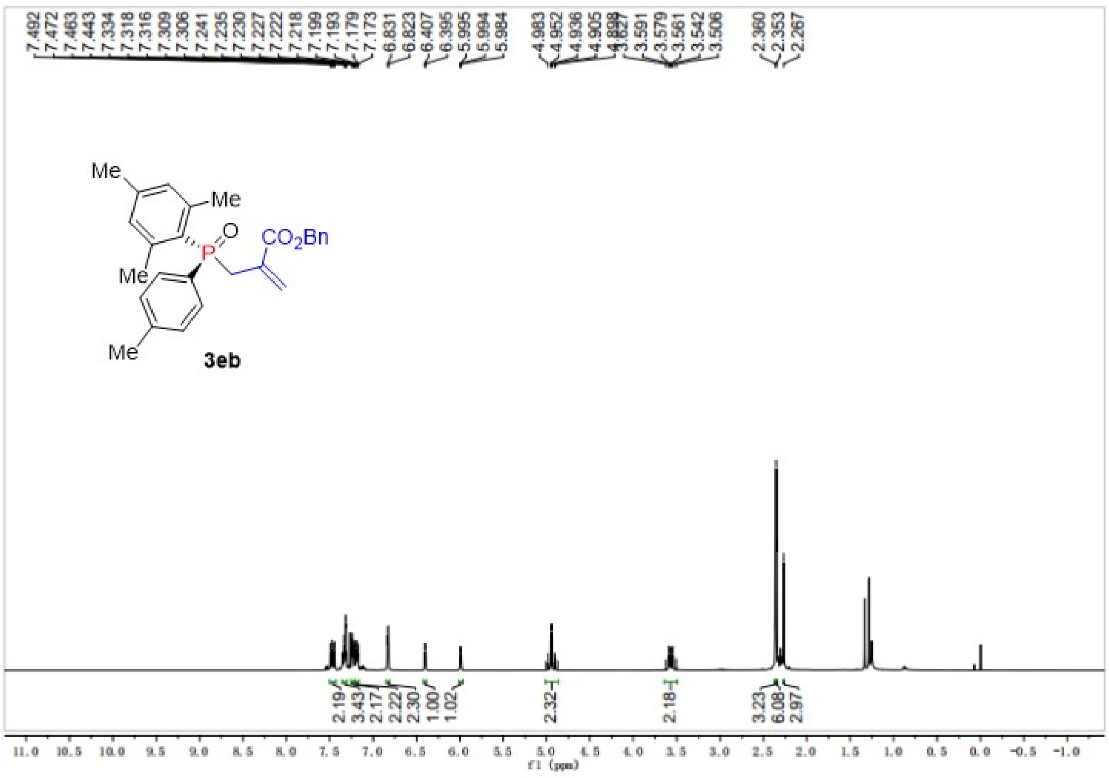
7.492
7.472
7.463
7.443
7.334
7.318
7.316
7.309
7.306
7.241
7.235
7.230
7.227
7.222
7.218
7.199
7.193
7.179
7.173
6.831
6.823
6.407
6.395
5.995
5.994
5.984
4.983
4.952
4.936
4.905
3.591
3.579
3.561
3.542
3.506
2.360
2.353
2.267
Me
Me
O
P
CO2Bn
Me
Me
3eb
2.19
3.43
2.17
2.22
2.30
1.00
1.02
2.32
2.18
3.23
6.08
2.97
f1 (ppm)

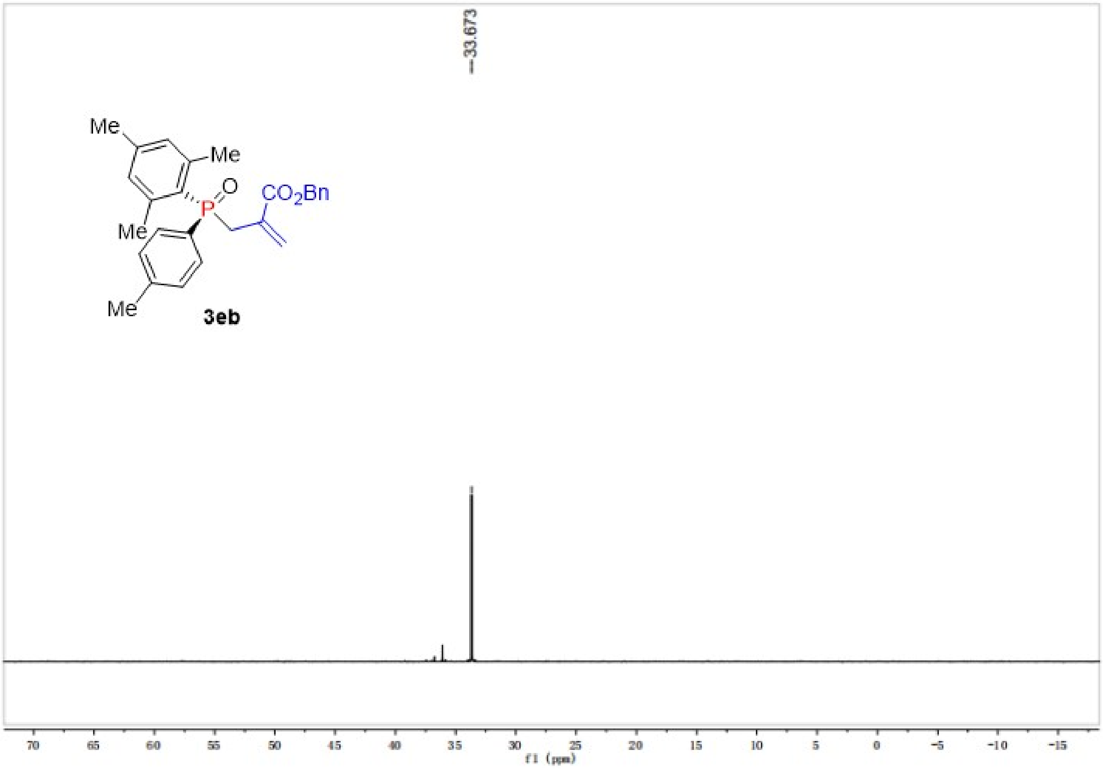

33.673
Me
Me
O
P
CO2Bn
Me
Me
3eb
70 65 60 55 50 45 40 35 30 25 20 15 10 5 0 -5 -10 -15
f1 (ppm)

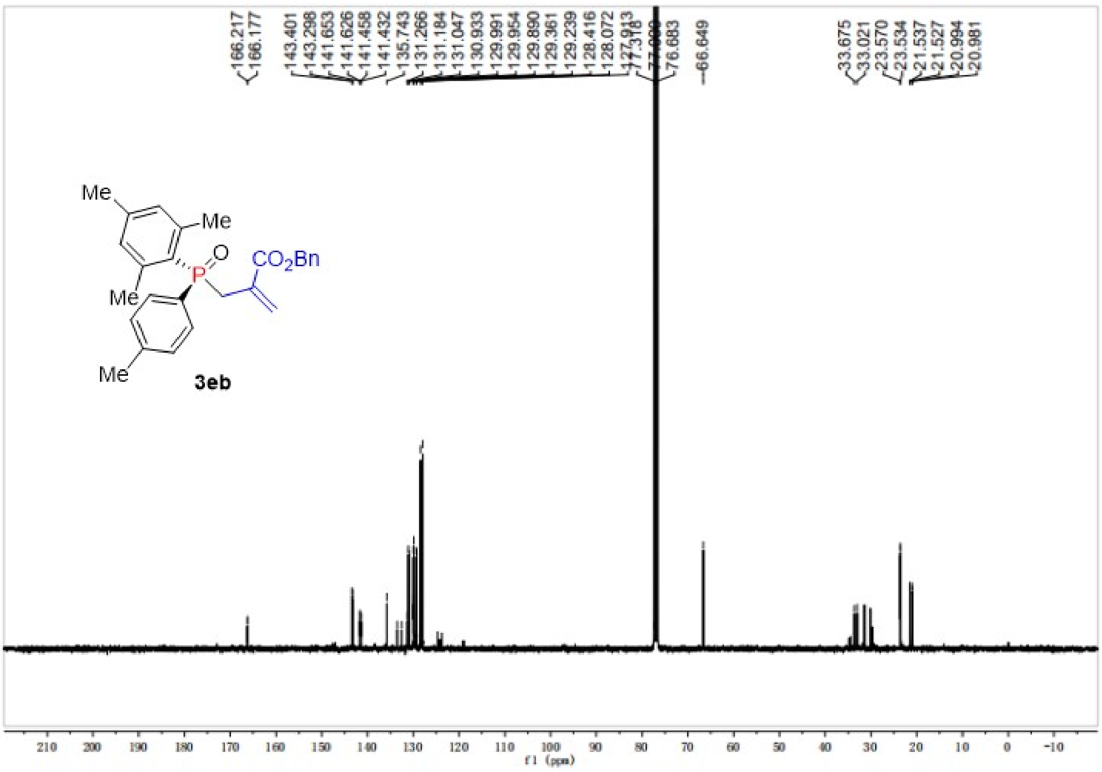

166.217
166.177
143.401
143.298
141.653
141.626
141.458
141.432
135.743
131.266
131.184
131.047
130.933
129.991
129.954
129.890
129.361
129.239
128.416
128.072
127.913
77.318
76.683
66.649
33.675
33.021
23.570
23.534
21.537
21.527
20.994
20.981
Me
Me
O
P
CO2Bn
Me
Me
3eb
210 200 190 180 170 160 150 140 130 120 110 100 90 80 70 60 50 40 30 20 10 0 -10
f1 (ppm)

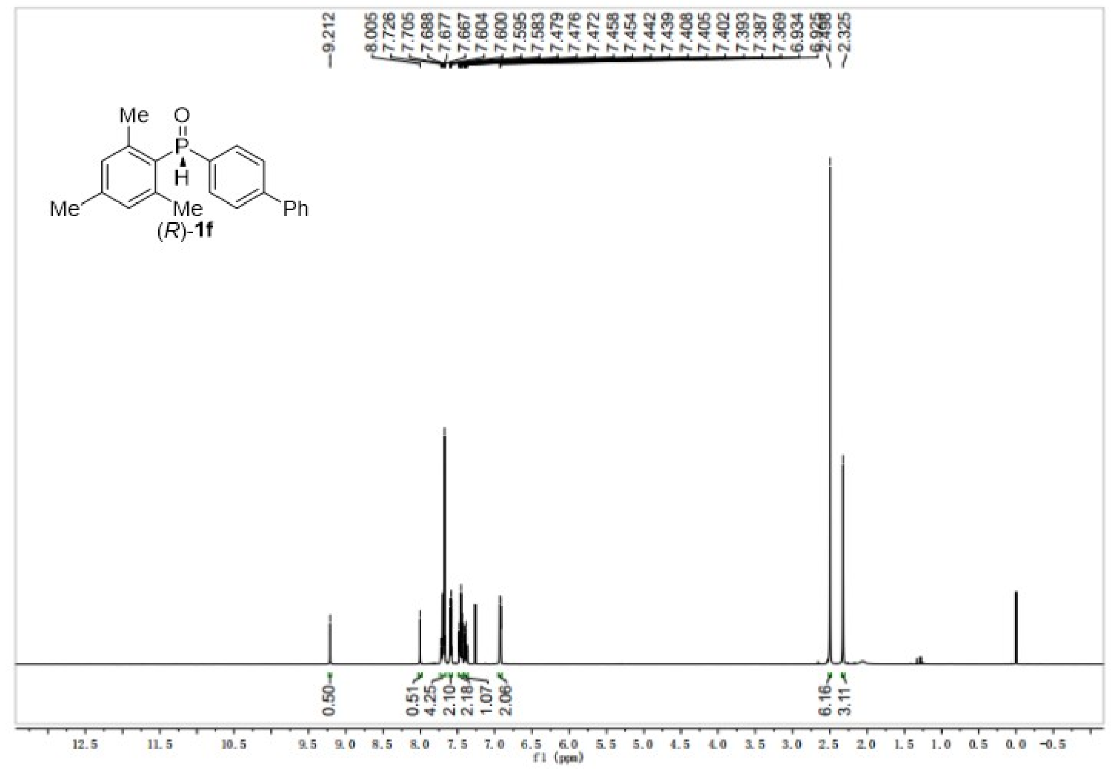

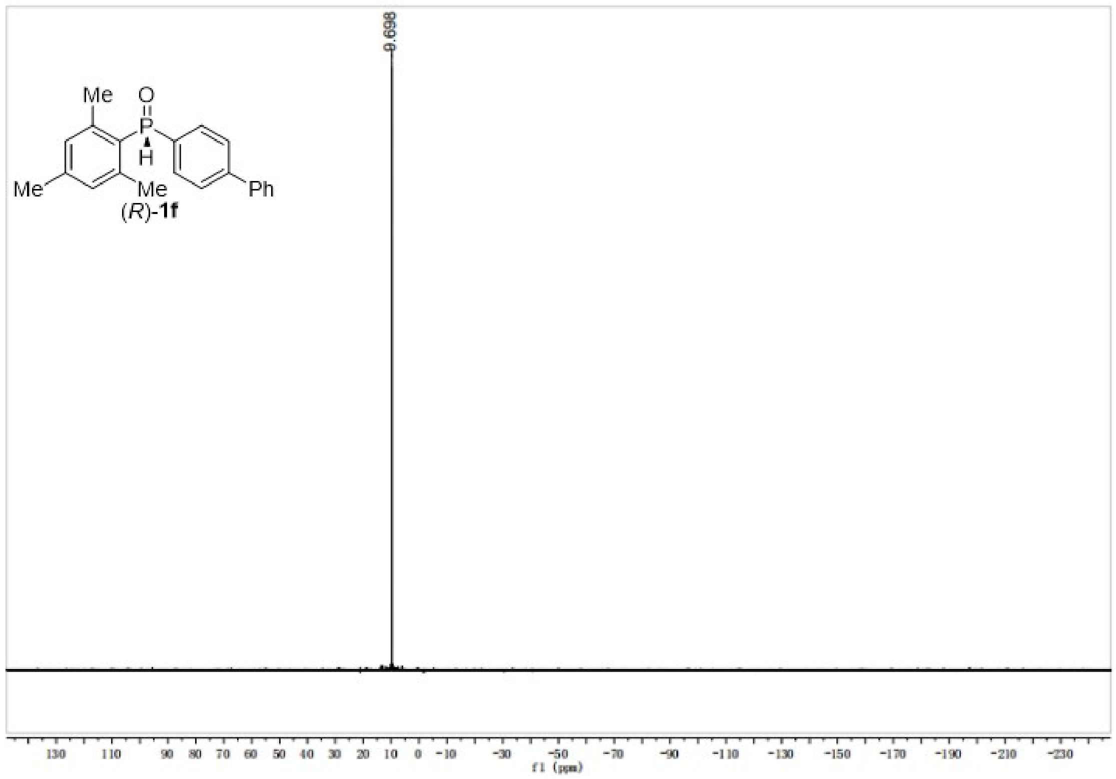

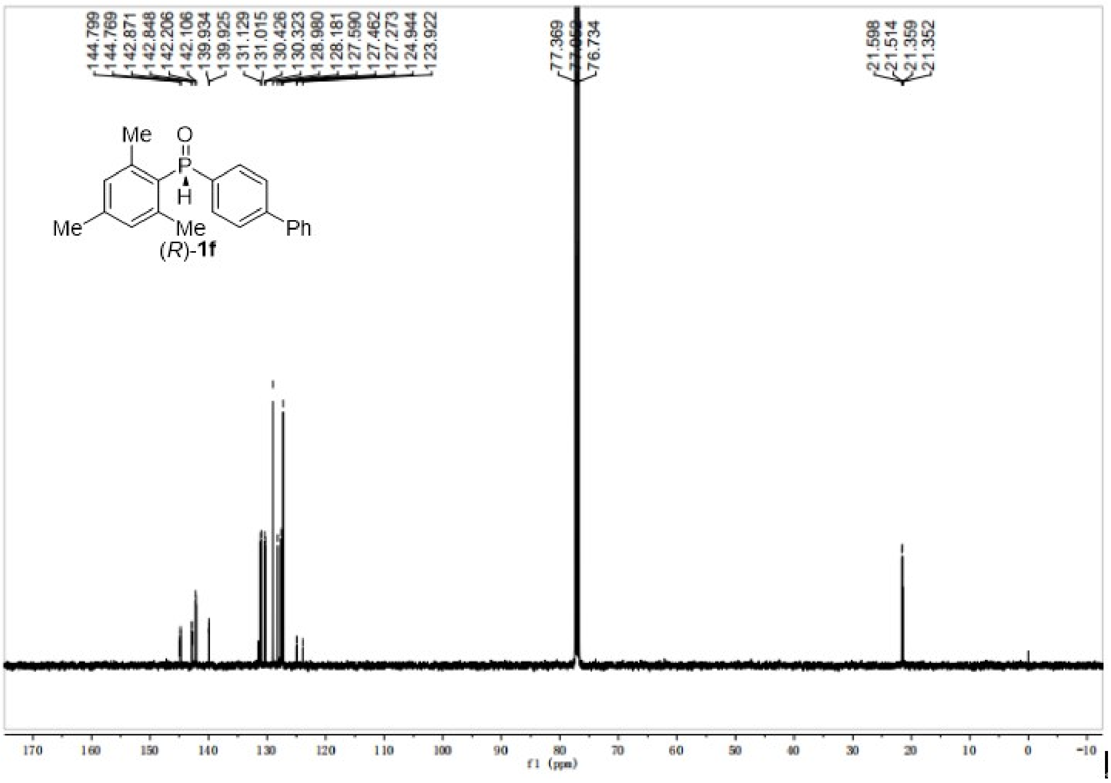
144.799
144.769
142.871
142.848
142.206
142.106
139.934
139.925
131.129
131.015
130.426
130.323
128.980
128.181
127.590
127.462
127.273
124.944
123.922
77.369
76.734
21.598
21.514
21.359
21.352
Me
O
P
H
Me
Me
Ph
(R)-1f
f1 (ppm)

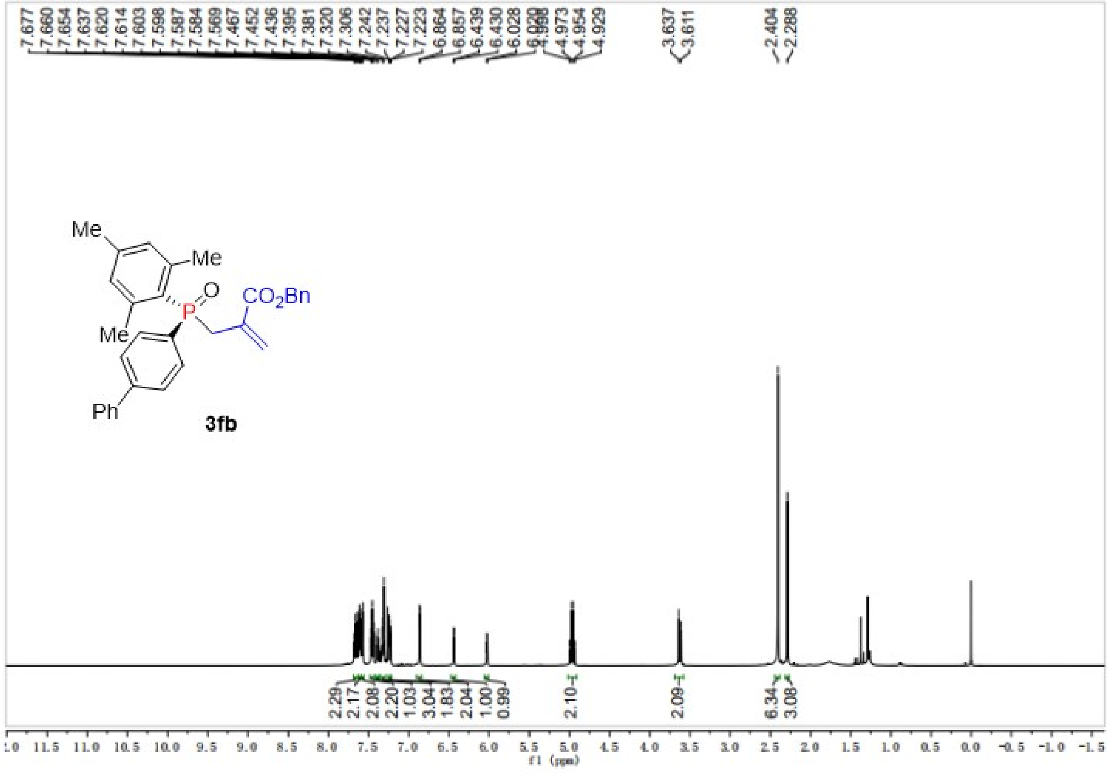
7.677
7.660
7.654
7.637
7.620
7.614
7.603
7.598
7.587
7.584
7.569
7.467
7.452
7.436
7.395
7.381
7.320
7.306
7.242
7.237
7.227
7.223
6.864
6.857
6.439
6.430
6.028
4.973
4.954
4.929
3.637
3.611
2.404
2.288
Me
Me
Me
O
P
CO2Bn
Ph
3fb
2.29
2.17
2.08
2.20
1.03
3.04
1.83
2.04
1.00
0.99
2.10
2.09
6.34
3.08
f1 (ppm)

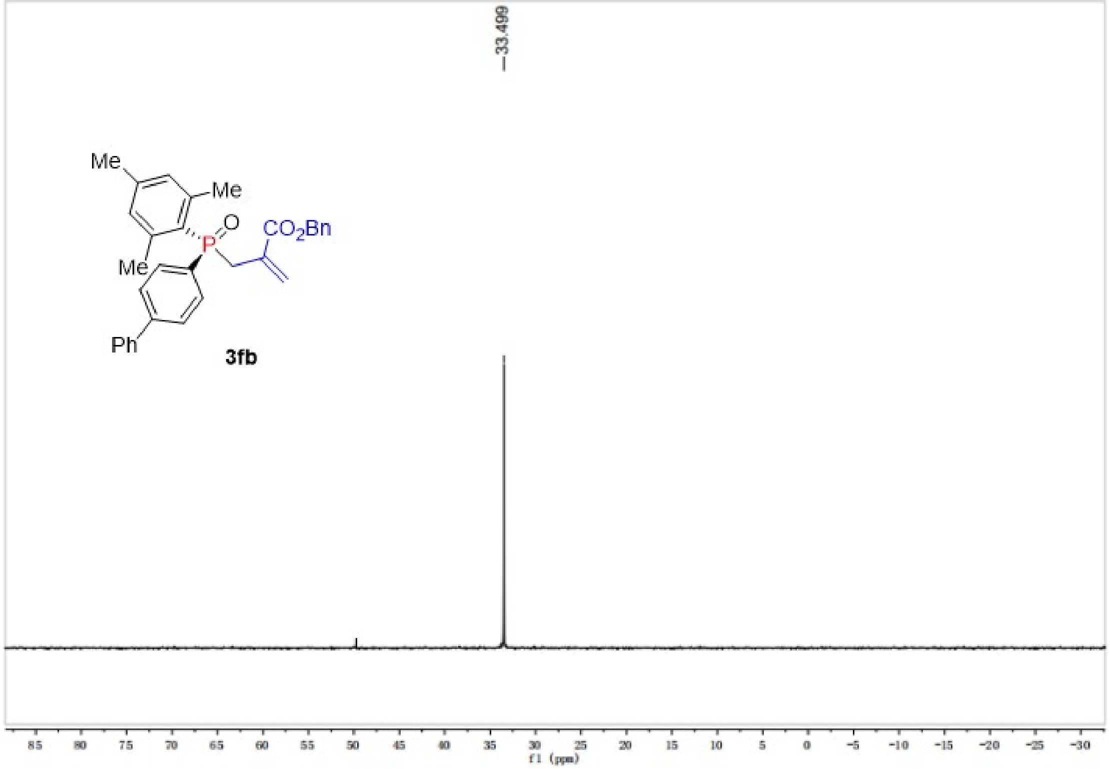

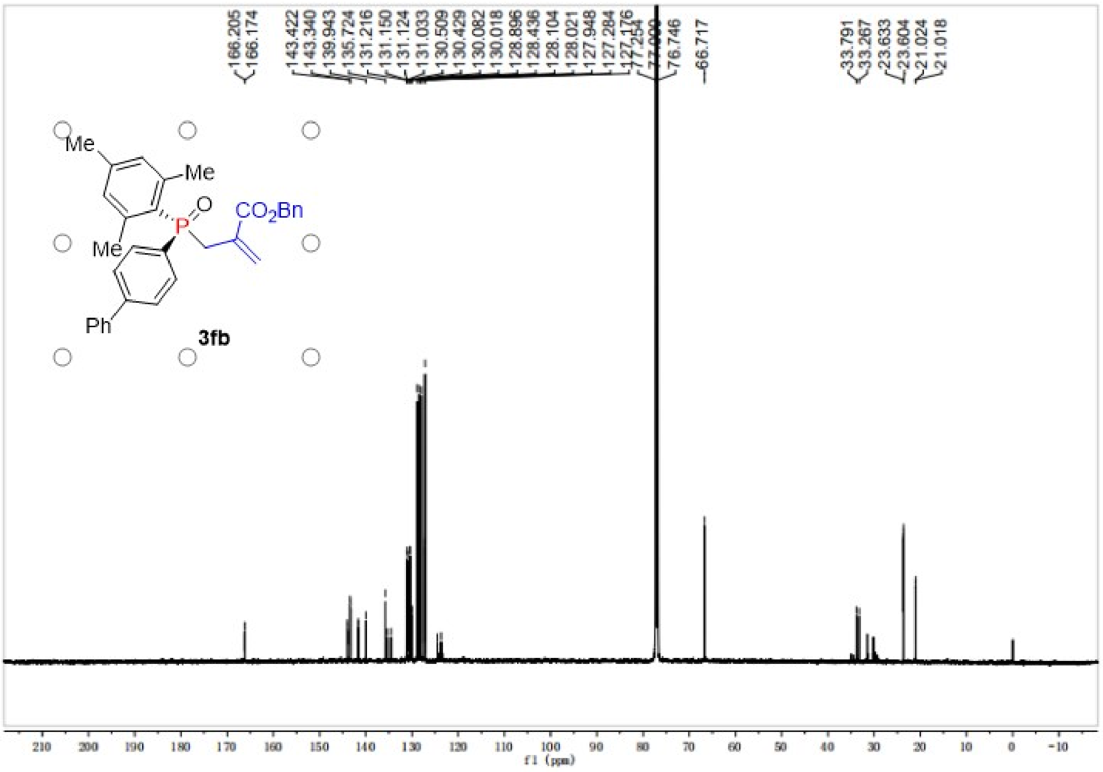

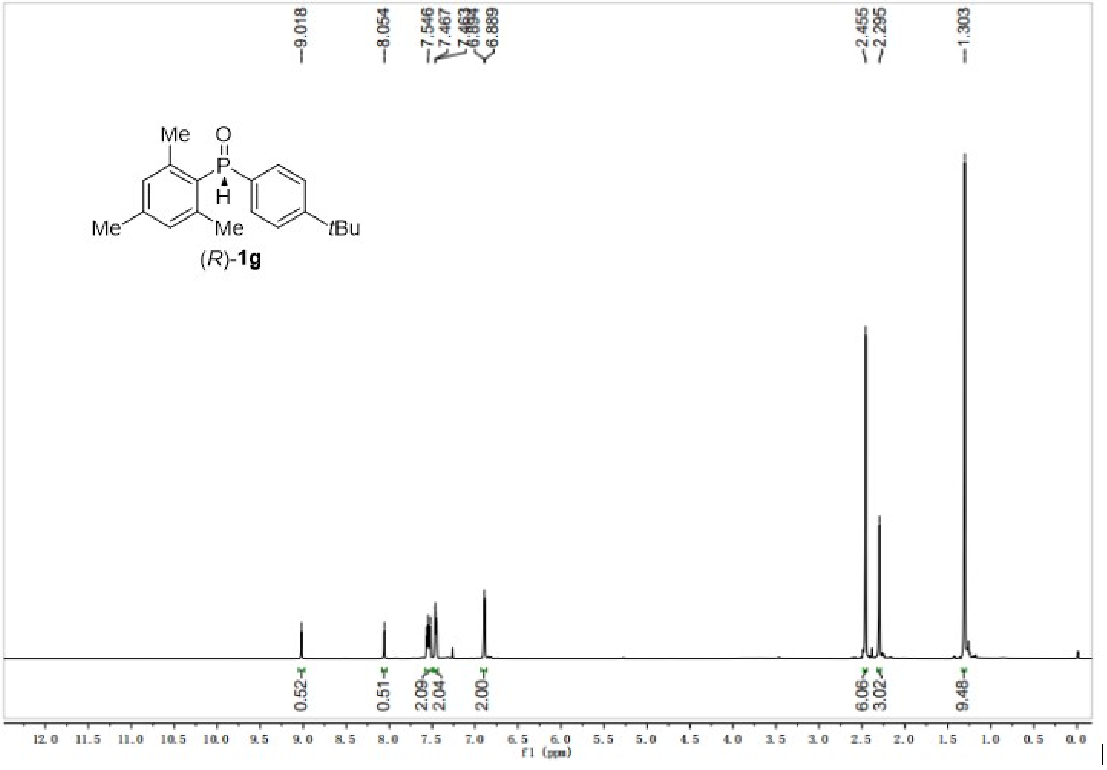

9.018
8.054
7.546
7.467
6.889
2.455
2.295
1.303
Me
O
P
H
Me
Me
tBu
(R)-1g
0.52
0.51
2.09
2.04
2.00
6.06
3.02
9.48
f1 (ppm)

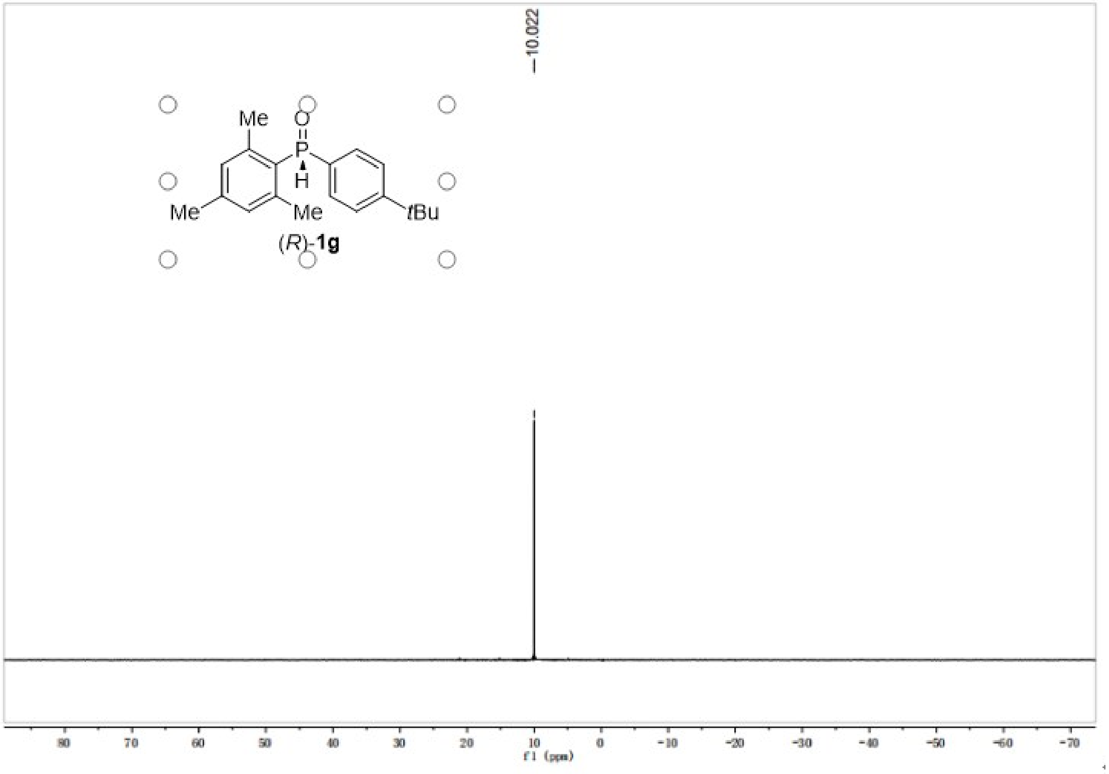

10.022
Me
O
P
H
Me
Me
tBu
(R)-1g
f1 (ppm)

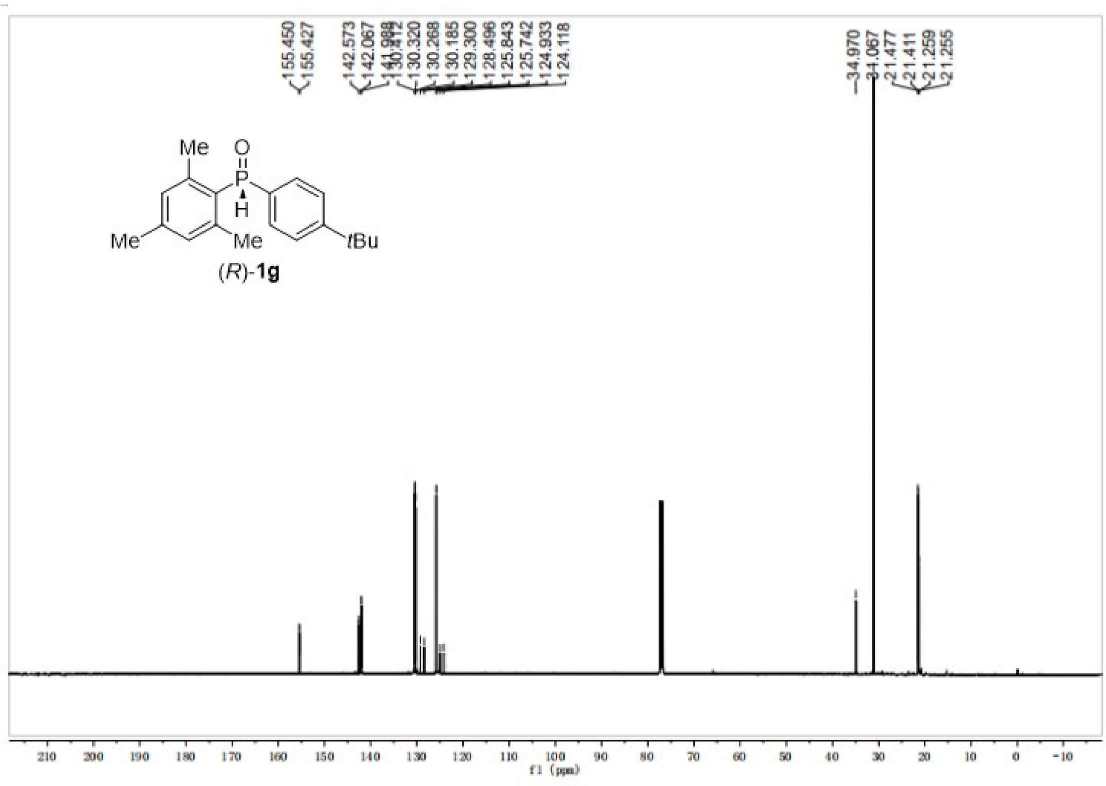

155.450
155.427
142.573
142.067
141.988
130.412
130.320
130.268
130.185
129.300
128.496
125.843
125.742
124.933
124.118
34.970
31.067
21.477
21.411
21.259
21.255
Me
O
P
H
Me
Me
tBu
(R)-1g
f1 (ppm)

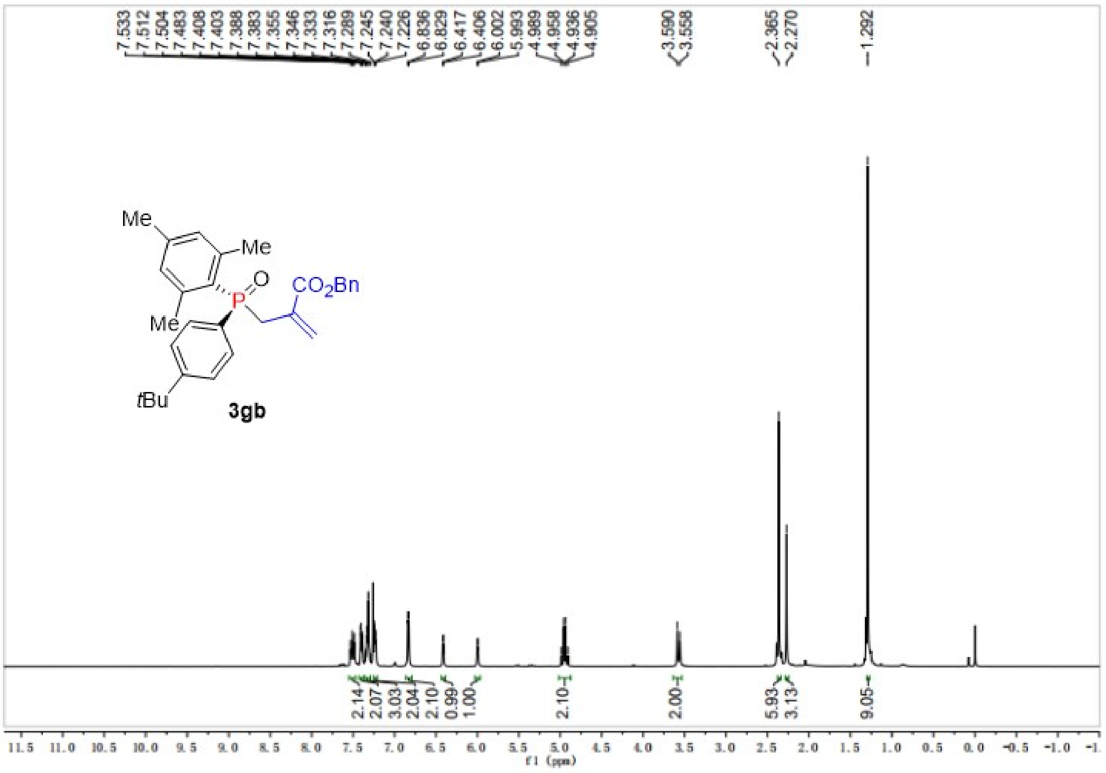

7.533
7.512
7.504
7.483
7.408
7.403
7.388
7.383
7.355
7.346
7.333
7.316
7.289
7.245
7.240
7.226
6.836
6.829
6.417
6.406
6.002
5.993
4.989
4.958
4.936
4.905
3.590
3.558
2.365
2.270
1.292
Me
Me
O
P
CO2Bn
Me
tBu
3gb
2.14
2.07
3.03
2.04
2.10
0.99
1.00
2.10
2.00
5.93
3.13
9.05
f1 (ppm)

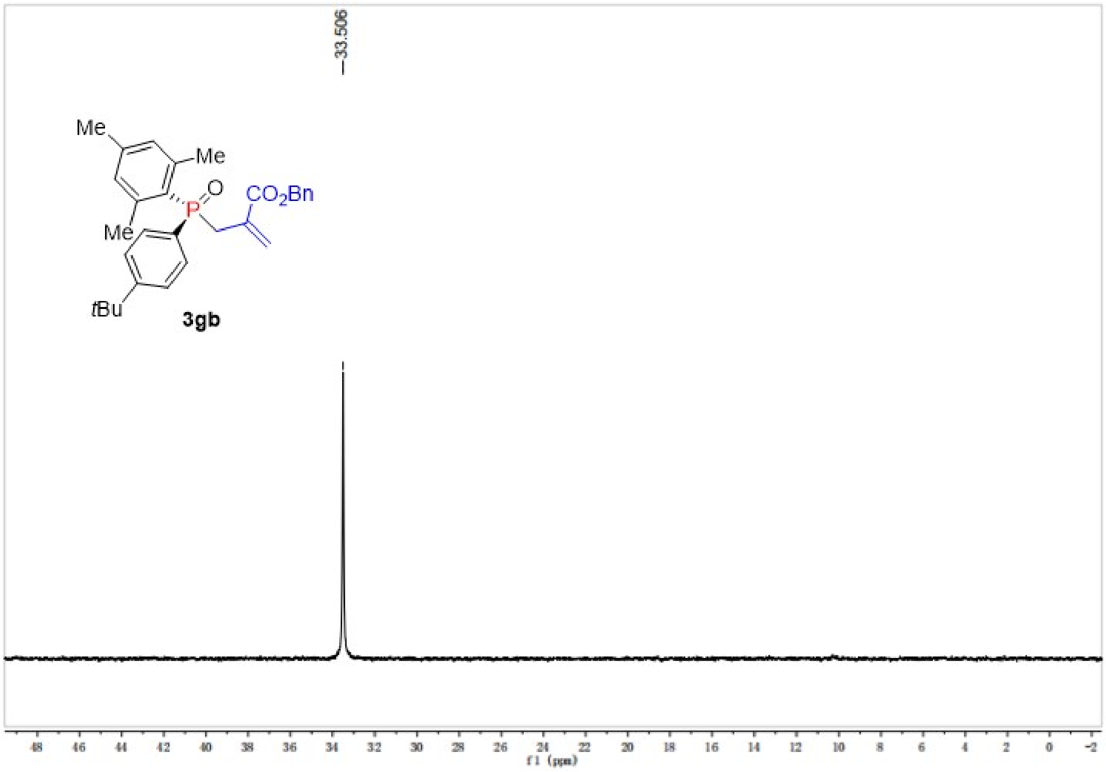
33.506
Me
Me
O
CO2Bn
P
Me
tBu
3gb
f1 (ppm)

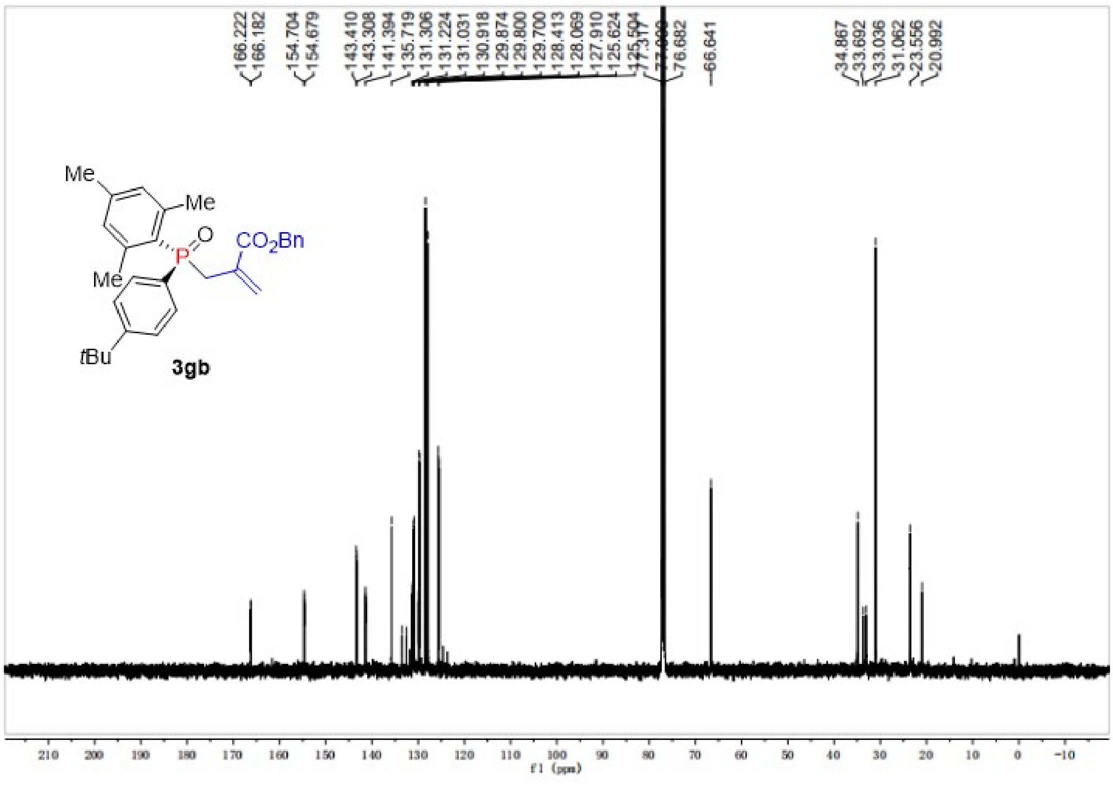
166.222
166.182
154.704
154.679
143.410
143.308
141.394
135.719
131.306
131.224
131.031
130.918
129.874
129.800
129.700
128.413
128.069
127.910
125.624
125.504
77.317
76.682
66.641
34.867
33.692
33.036
31.062
23.556
20.992
Me
Me
O
CO2Bn
P
Me
tBu
3gb
f1 (ppm)

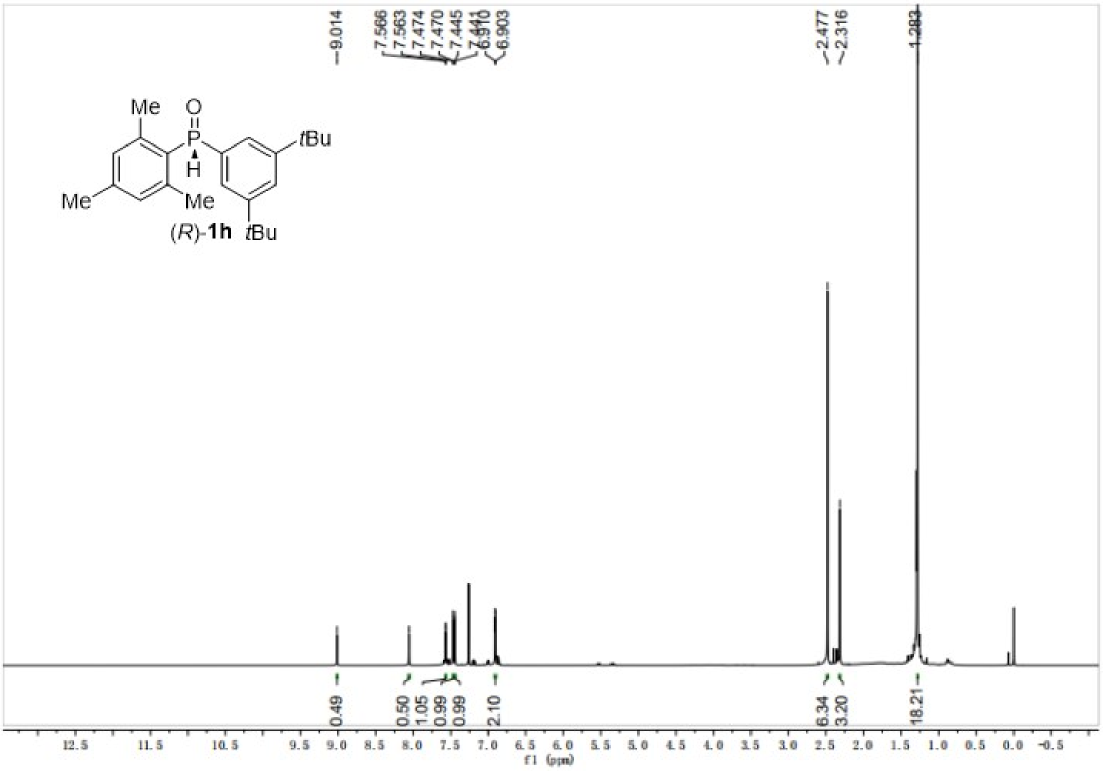
(R)-1h

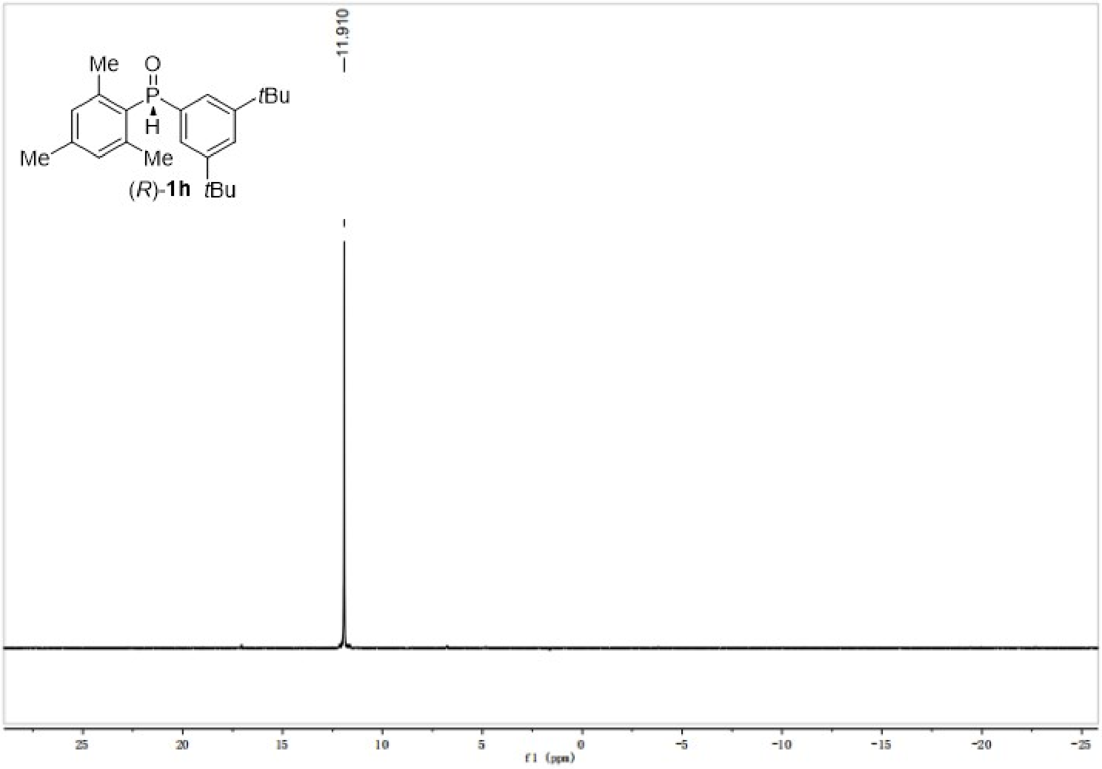
(R)-1h

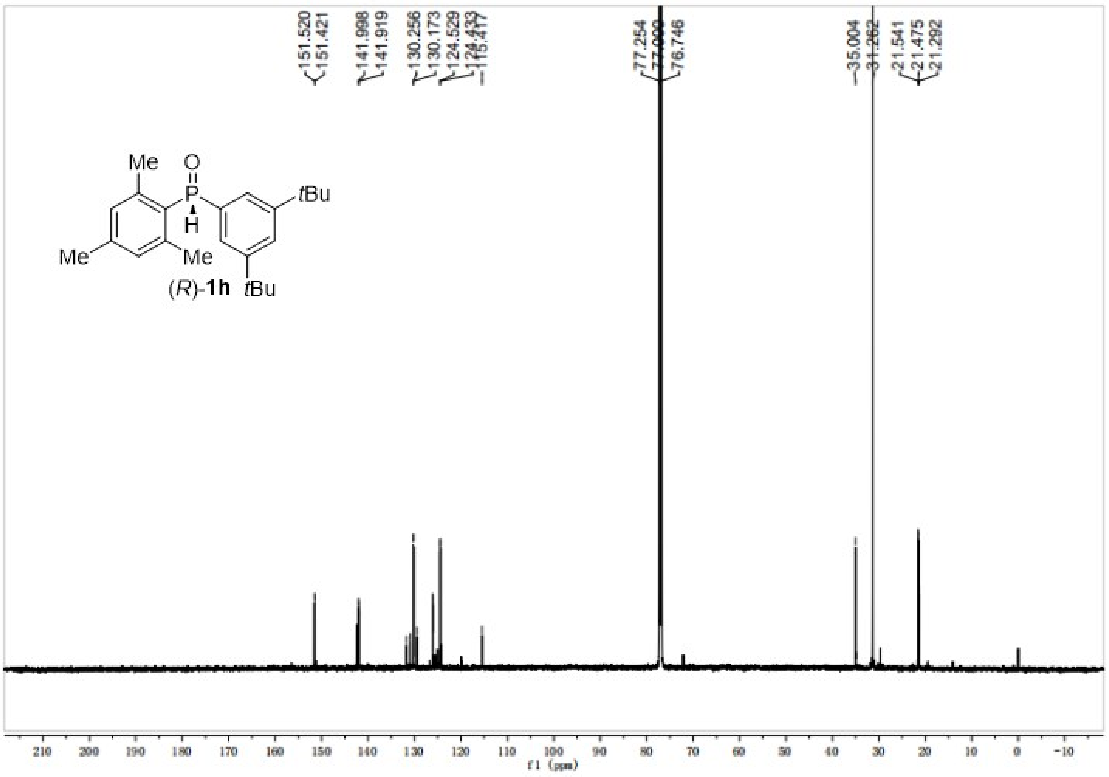
151.520
151.421
141.998
141.919
130.256
130.173
124.529
124.433
115.417
77.254
77.000
76.746
35.004
31.262
21.541
21.475
21.292
(R)-1h
f1 (ppm)

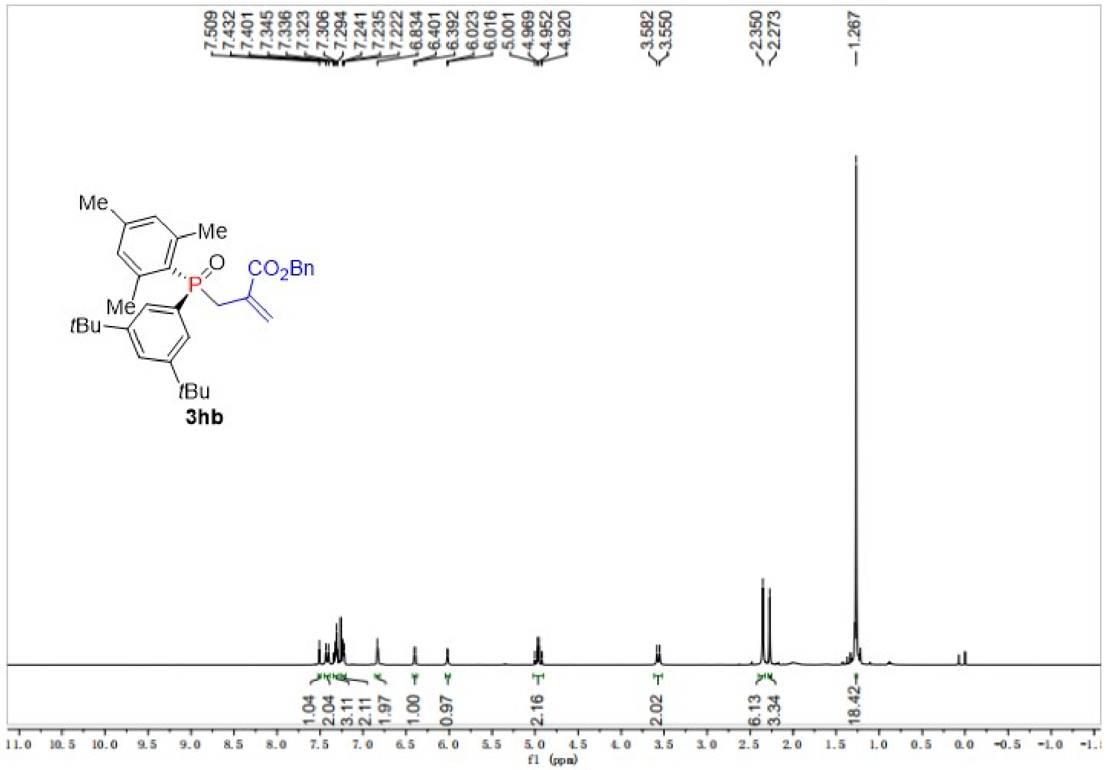
7.509
7.432
7.401
7.345
7.336
7.323
7.306
7.294
7.241
7.235
7.222
6.834
6.401
6.392
6.023
6.016
5.001
4.969
4.952
4.920
3.582
3.550
2.350
2.273
1.267
CO2Bn
3hb
1.04
2.04
3.11
2.11
1.97
1.00
0.97
2.16
2.02
6.13
3.34
18.42
f1 (ppm)

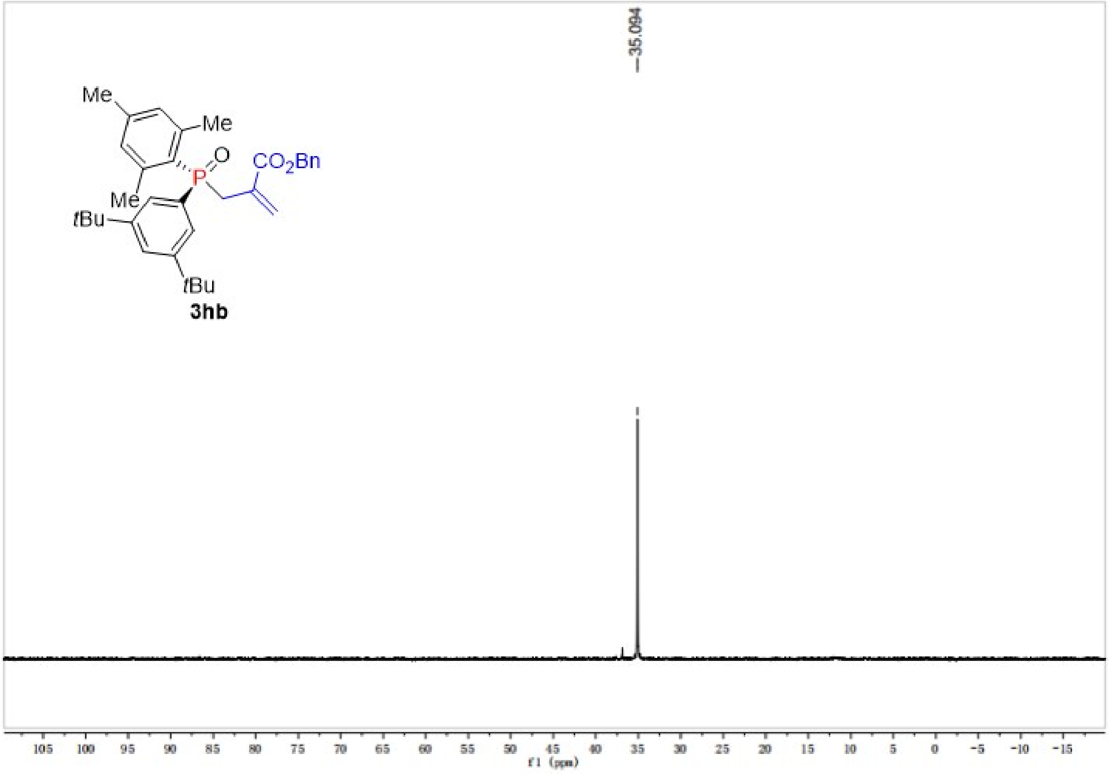
35.094
Me
Me
O
P
CO_2Bn
Me
tBu
tBu
3hb
105 100 95 90 85 80 75 70 65 60 55 50 45 40 35 30 25 20 15 10 5 0 -5 -10 -15
f1 (ppm)

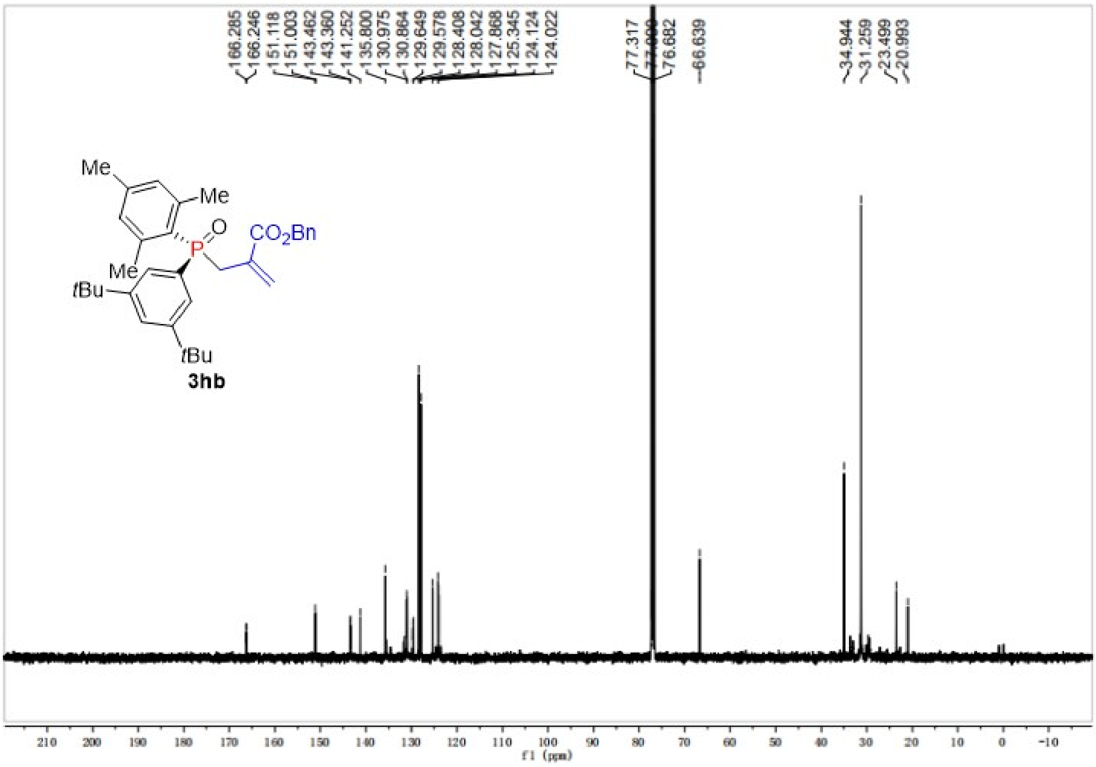
166.285
166.246
151.118
151.003
143.462
143.360
141.252
135.800
130.975
130.864
129.649
129.578
128.408
128.042
127.868
125.345
124.124
124.022
77.317
76.682
66.639
34.944
31.259
23.499
20.993
Me
Me
O
P
CO_2Bn
Me
tBu
tBu
3hb
210 200 190 180 170 160 150 140 130 120 110 100 90 80 70 60 50 40 30 20 10 0 -10
f1 (ppm)

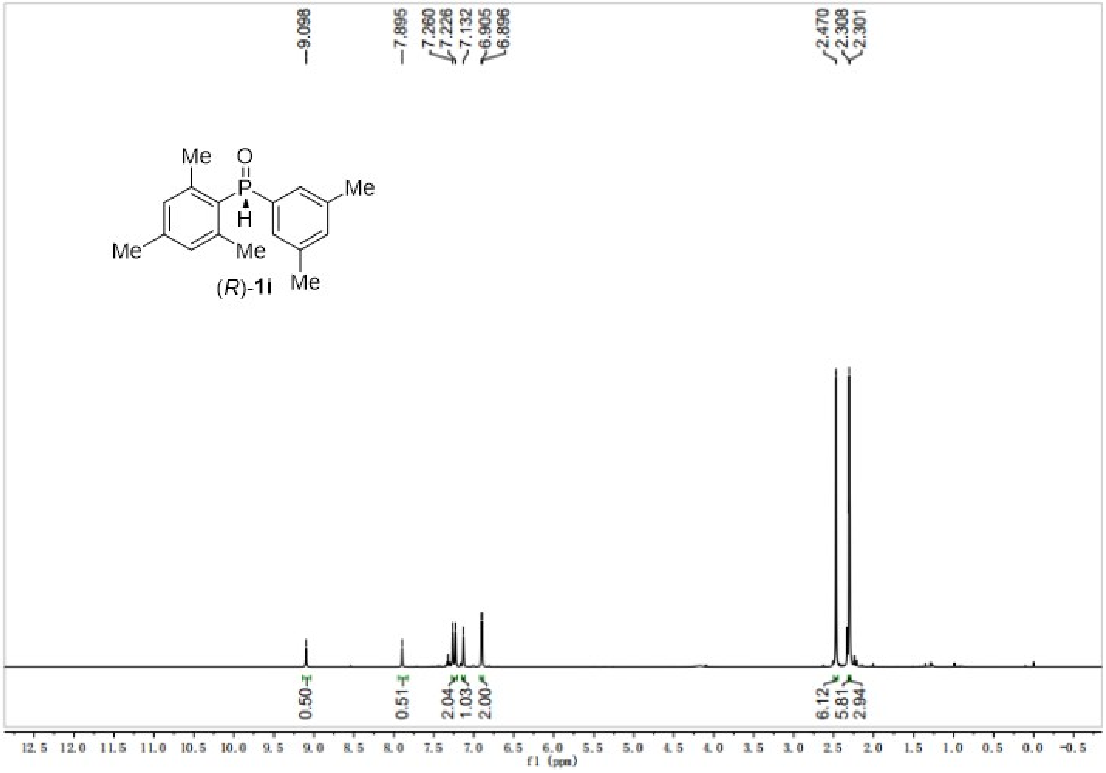
9.098
7.895
7.260
7.226
7.132
6.905
6.896
2.470
2.308
2.301
Me
O
P
H
Me
Me
Me
Me
(R)-1i
0.50
0.51
2.04
1.03
2.00
6.12
5.81
2.94
f1 (ppm)

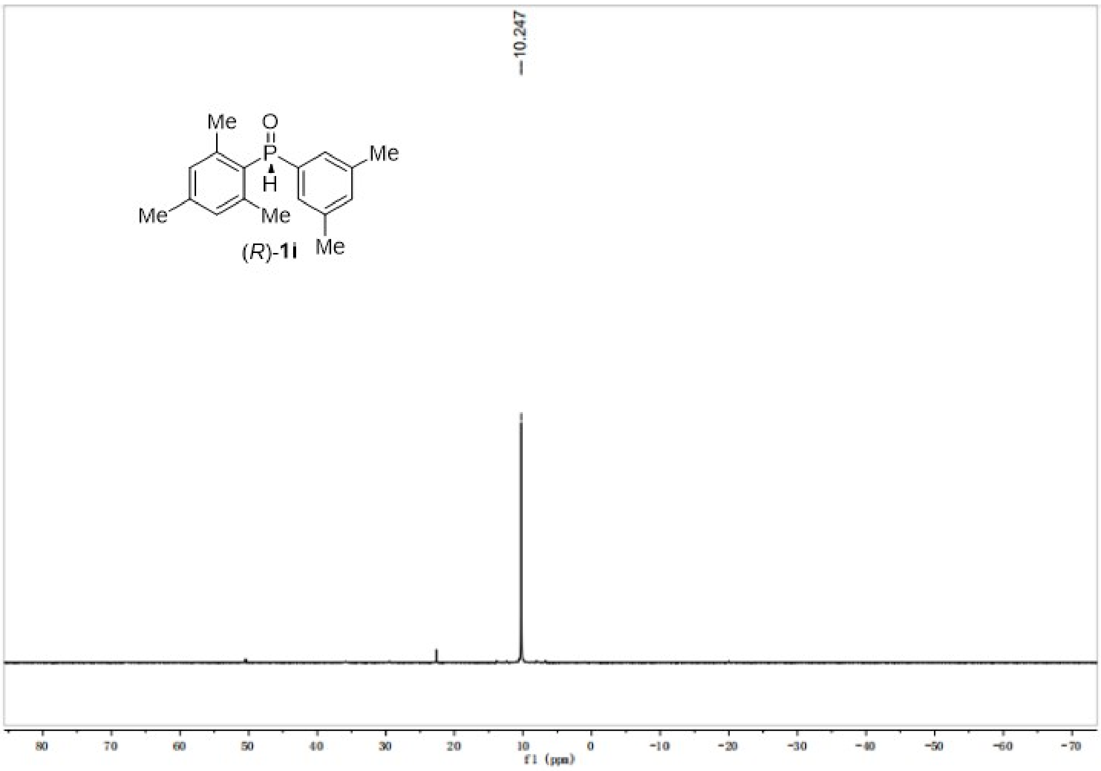
10.247
Me
O
P
H
Me
Me
Me
Me
(R)-1i
f1 (ppm)

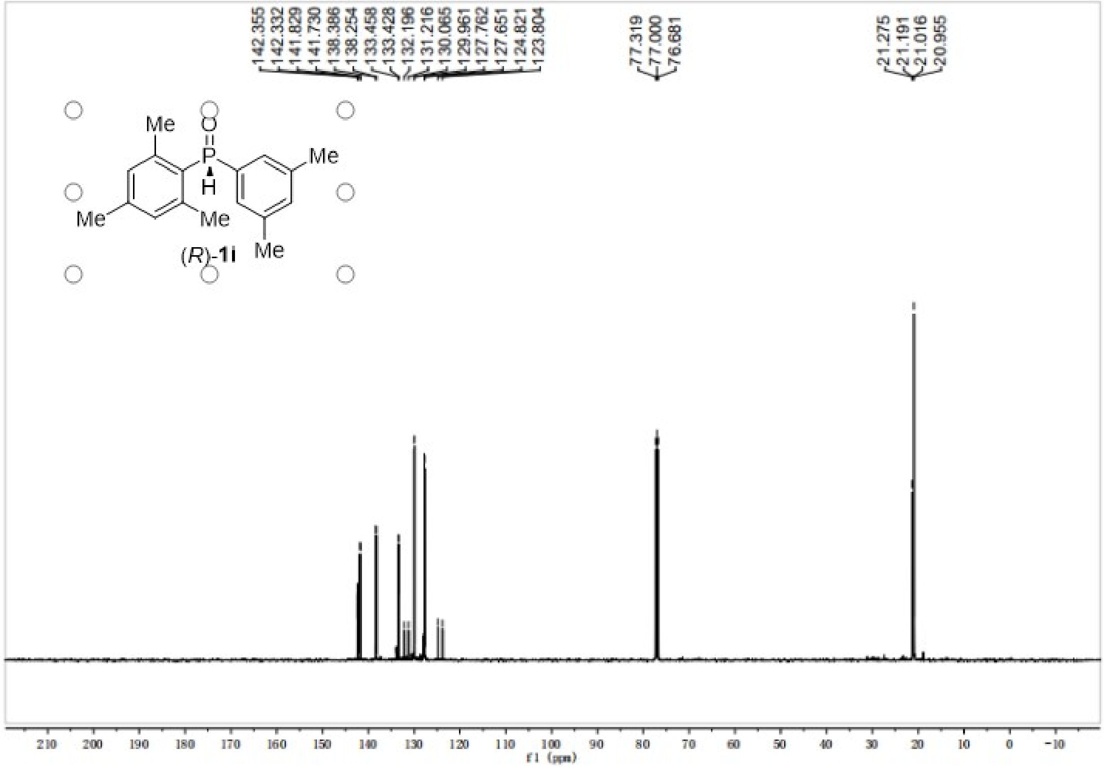
142.355
142.332
141.829
141.730
138.386
138.254
133.458
133.428
132.196
131.216
130.065
129.961
127.762
127.651
124.821
123.804
77.319
77.000
76.681
21.275
21.191
21.016
20.955
Me
O
P
H
Me
Me
Me
Me
(R)-1i
f1 (ppm)

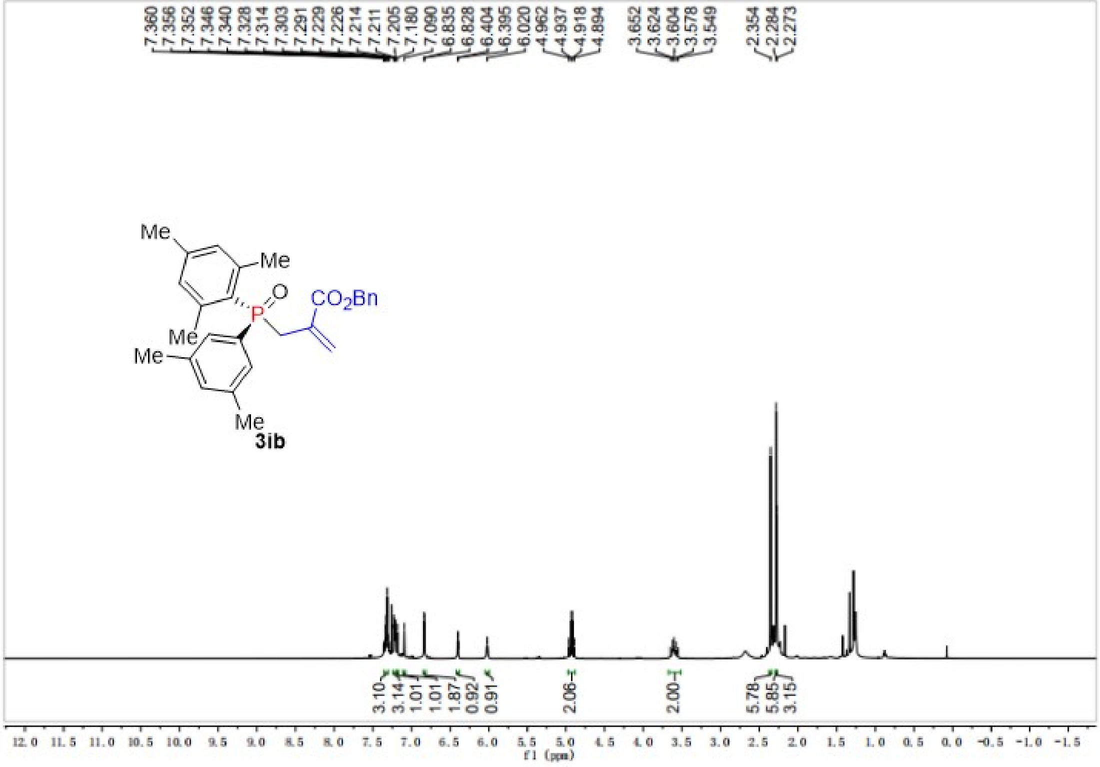
7.360
7.356
7.352
7.346
7.340
7.328
7.314
7.303
7.291
7.229
7.226
7.214
7.211
7.205
7.180
7.090
6.835
6.828
6.404
6.395
6.020
4.962
4.937
4.918
4.894
3.652
3.624
3.604
3.578
3.549
2.354
2.284
2.273
Me
Me
O
CO_2Bn
P
Me
Me
Me
3ib
3.10
3.14
1.01
1.01
1.87
0.92
0.91
2.06
2.00
5.78
5.85
3.15
f1 (ppm)

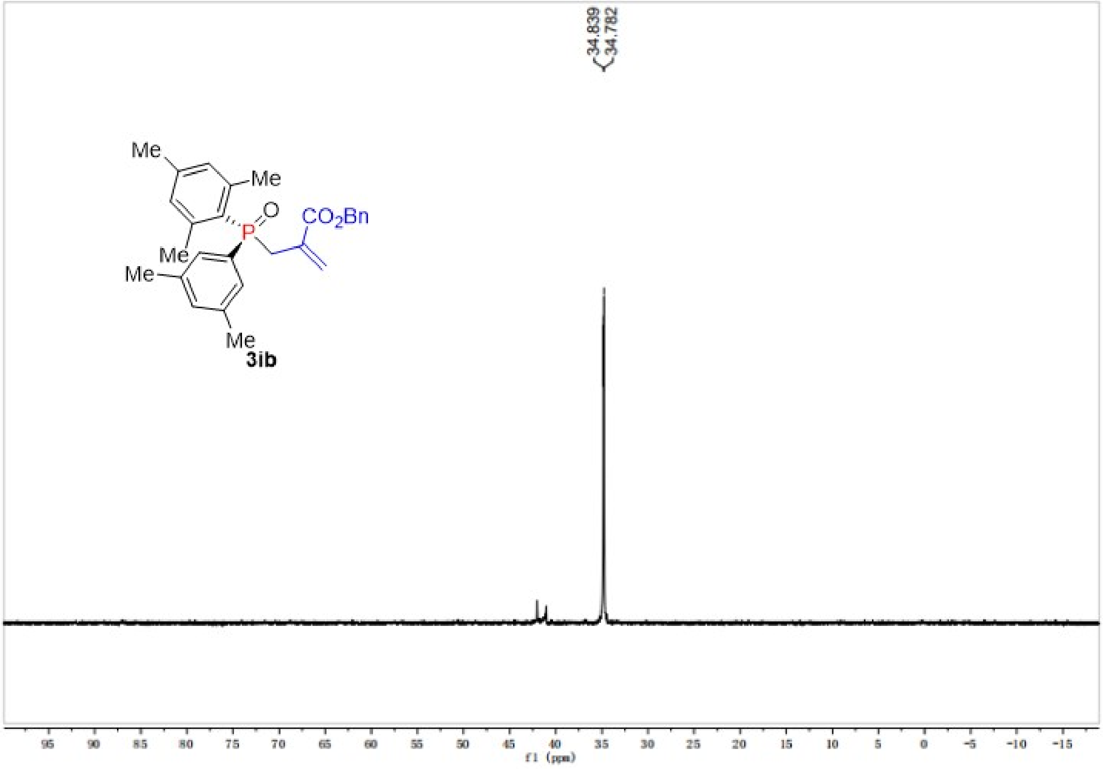
34.839
34.782
Me
Me
O
P
CO2Bn
Me
Me
Me
3ib
95
90
85
80
75
70
65
60
55
50
45
40
35
30
25
20
15
10
5
0
-5
-10
-15
f1 (ppm)

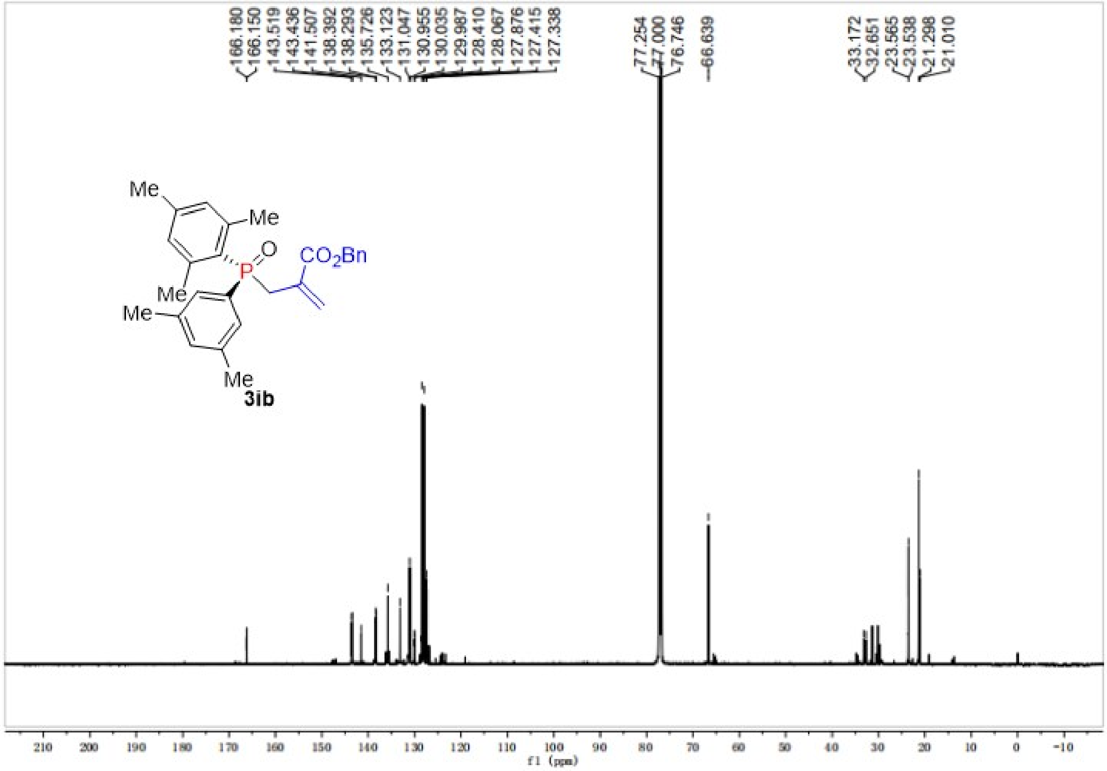
166.180
166.150
143.519
143.436
141.507
138.392
138.293
135.726
133.123
131.047
130.955
130.035
129.987
128.410
128.067
127.876
127.415
127.338
77.254
77.000
76.746
66.639
33.172
32.651
23.565
23.538
21.298
21.010
Me
Me
O
P
CO2Bn
Me
Me
Me
3ib
210
200
190
180
170
160
150
140
130
120
110
100
90
80
70
60
50
40
30
20
10
0
-10
f1 (ppm)

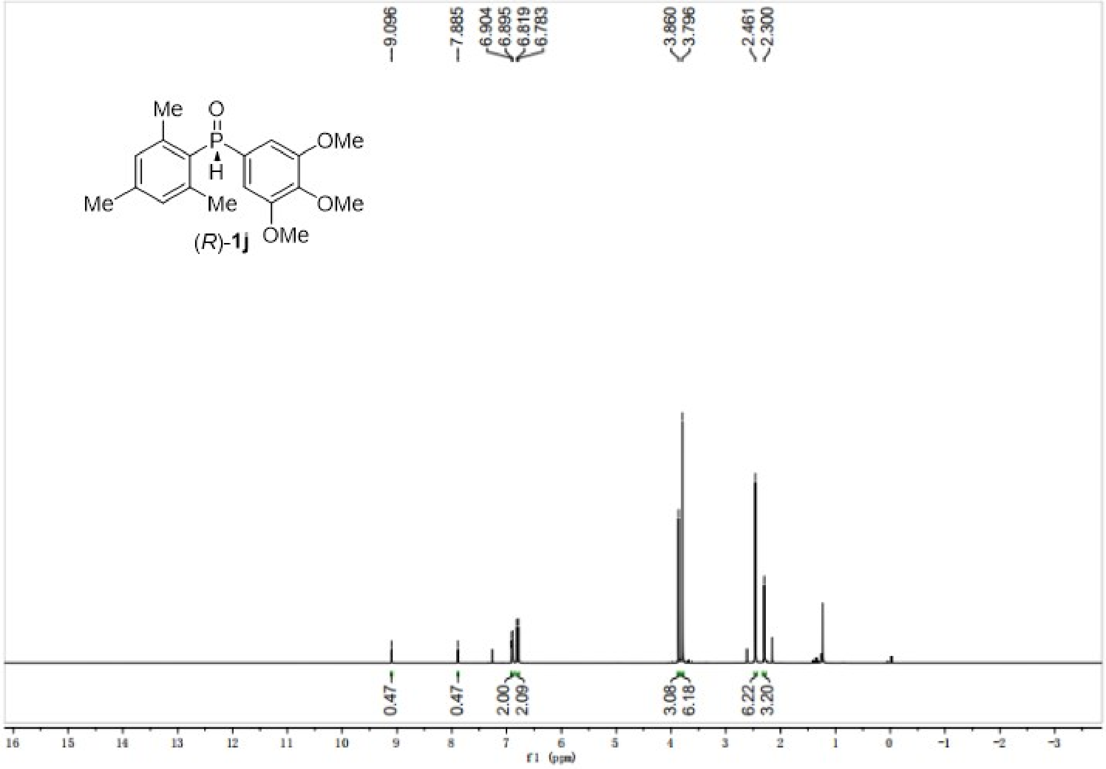
9.096
7.885
6.904
6.895
6.819
6.783
3.860
3.796
2.461
2.300
Me
O
P
H
OMe
Me
Me
OMe
(R)-1j
OMe
0.47
0.47
2.00
2.09
3.08
6.18
6.22
3.20
f1 (ppm)

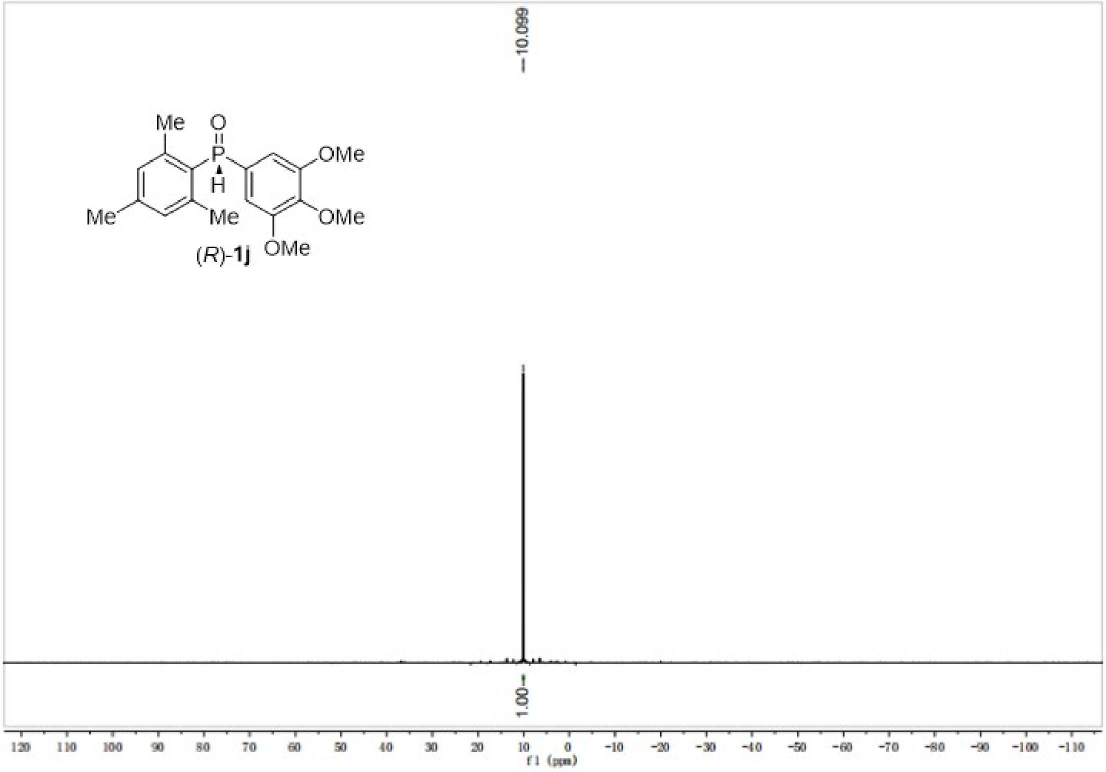
10.099
Me
O
P
H
OMe
Me
Me
OMe
(R)-1j
OMe
1.00
f1 (ppm)

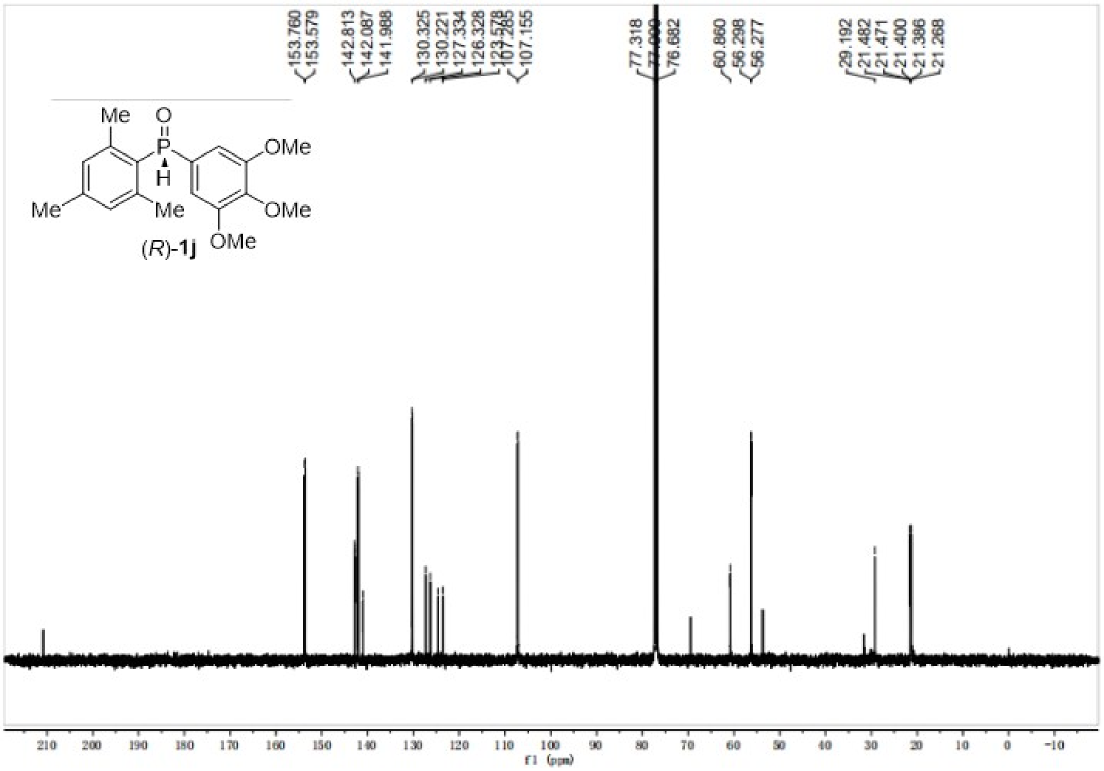
153.760
153.579
142.813
142.087
141.988
130.325
130.221
127.334
126.328
107.155
77.318
76.682
60.860
56.298
56.277
29.192
21.482
21.471
21.400
21.386
21.268
Me
O
P
H
OMe
Me
Me
OMe
(R)-1j
OMe
f1 (ppm)

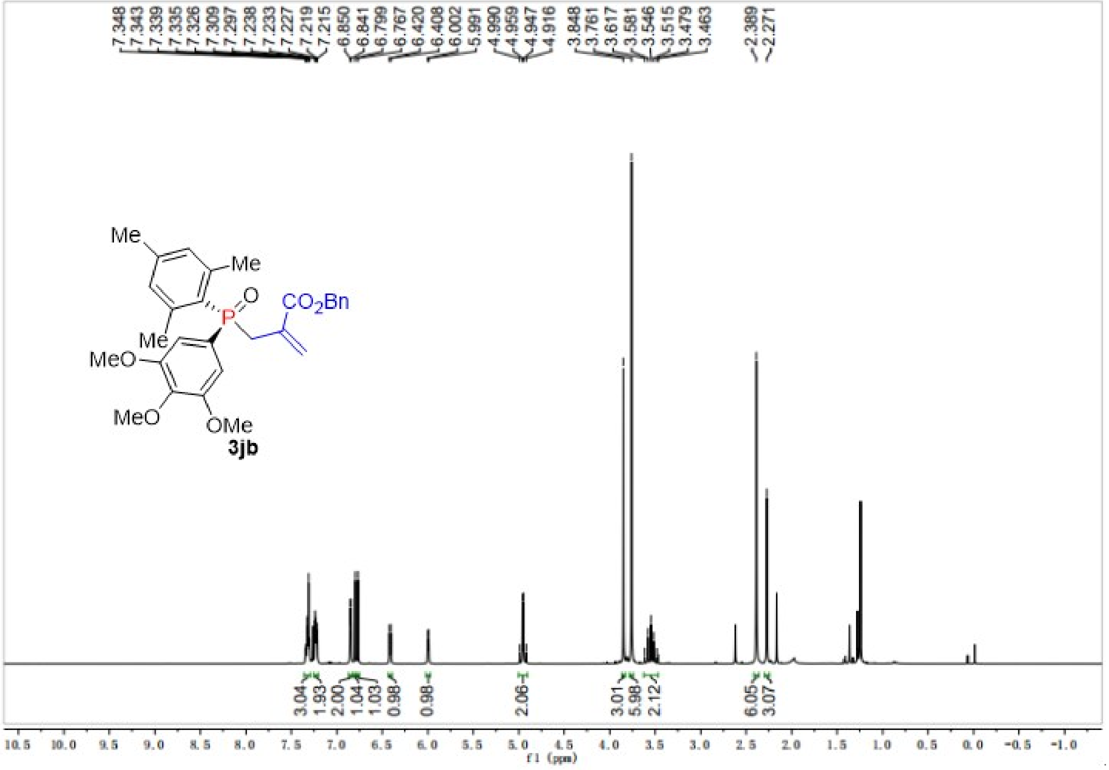
7.348
7.343
7.339
7.335
7.326
7.309
7.297
7.238
7.233
7.227
7.219
7.215
6.850
6.841
6.799
6.767
6.420
6.408
6.002
5.991
4.990
4.959
4.947
4.916
3.848
3.761
3.617
3.581
3.546
3.515
3.479
3.463
2.389
2.271
Me
Me
O
CO2Bn
P
Me
MeO
MeO
OMe
3jb
3.04
1.93
2.00
1.04
1.03
0.98
0.98
2.06
3.01
5.98
2.12
6.05
3.07
f1 (ppm)

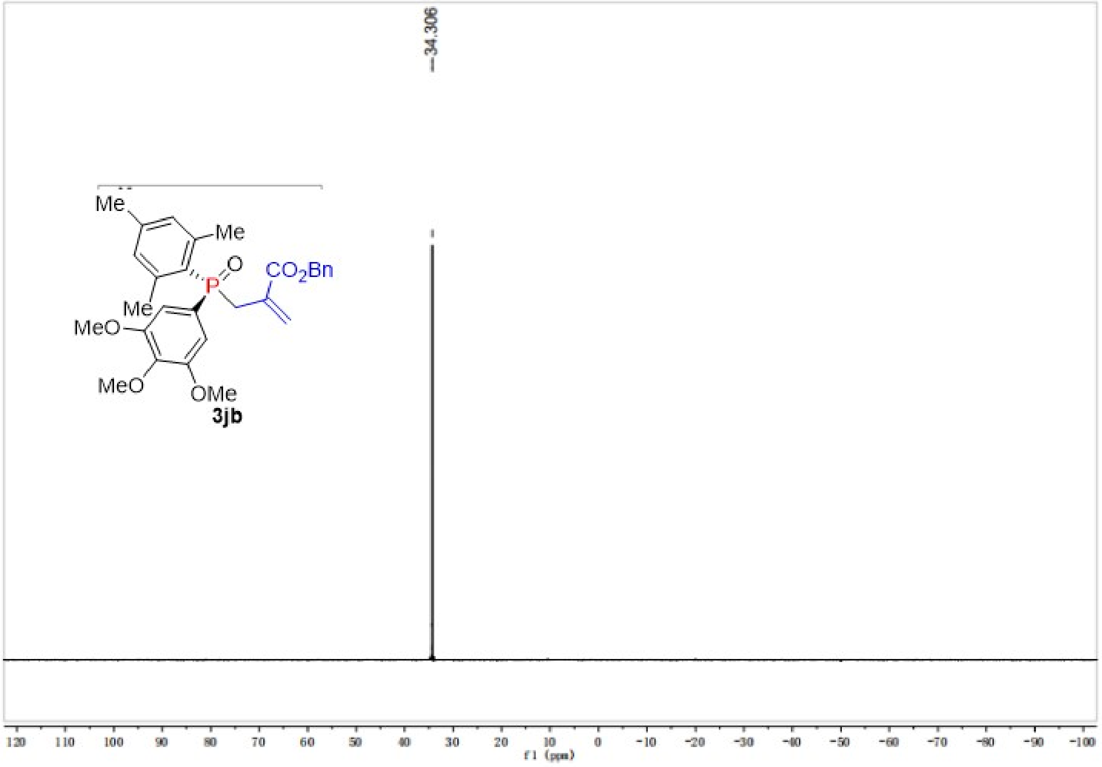

34.306
Me
Me
CO2Bn
O
P
Me
MeO
MeO
OMe
3jb
f1 (ppm)

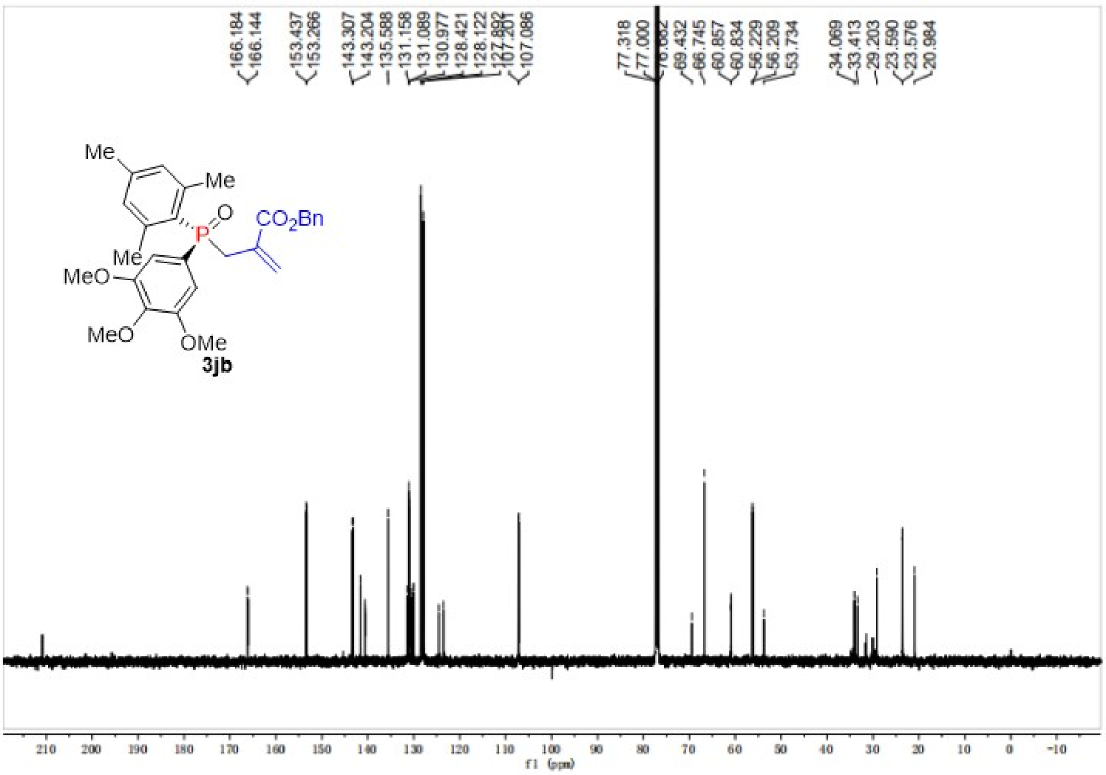

166.184
166.144
153.437
153.266
143.307
143.204
135.588
131.158
131.089
130.977
128.421
128.122
107.086
77.318
77.000
69.432
66.745
60.857
60.834
56.229
56.209
53.734
34.069
33.413
29.203
23.590
23.576
20.984
Me
Me
CO2Bn
O
P
Me
MeO
MeO
OMe
3jb
f1 (ppm)

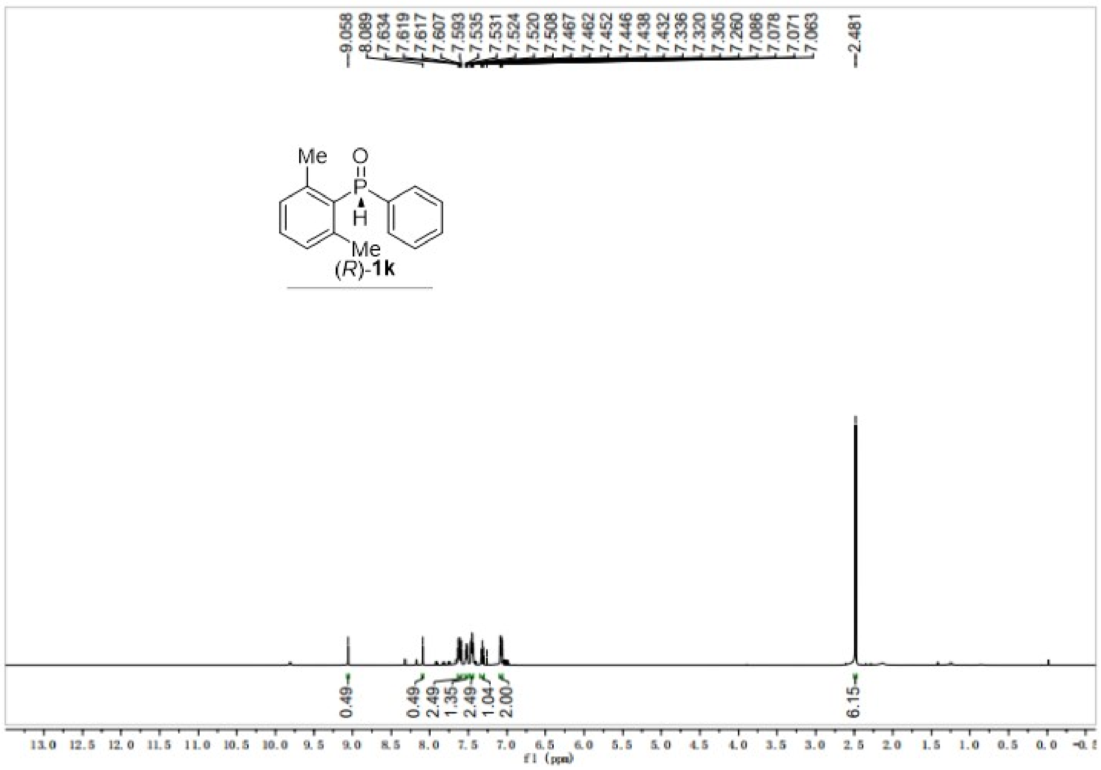

9.058
8.089
7.634
7.619
7.617
7.607
7.593
7.535
7.531
7.524
7.520
7.508
7.467
7.462
7.452
7.446
7.438
7.432
7.336
7.320
7.305
7.260
7.086
7.078
7.071
7.063
2.481
Me
O
P
H
Me
(R)-1k
0.49
0.49
2.49
1.35
2.49
1.04
2.00
6.15
f1 (ppm)

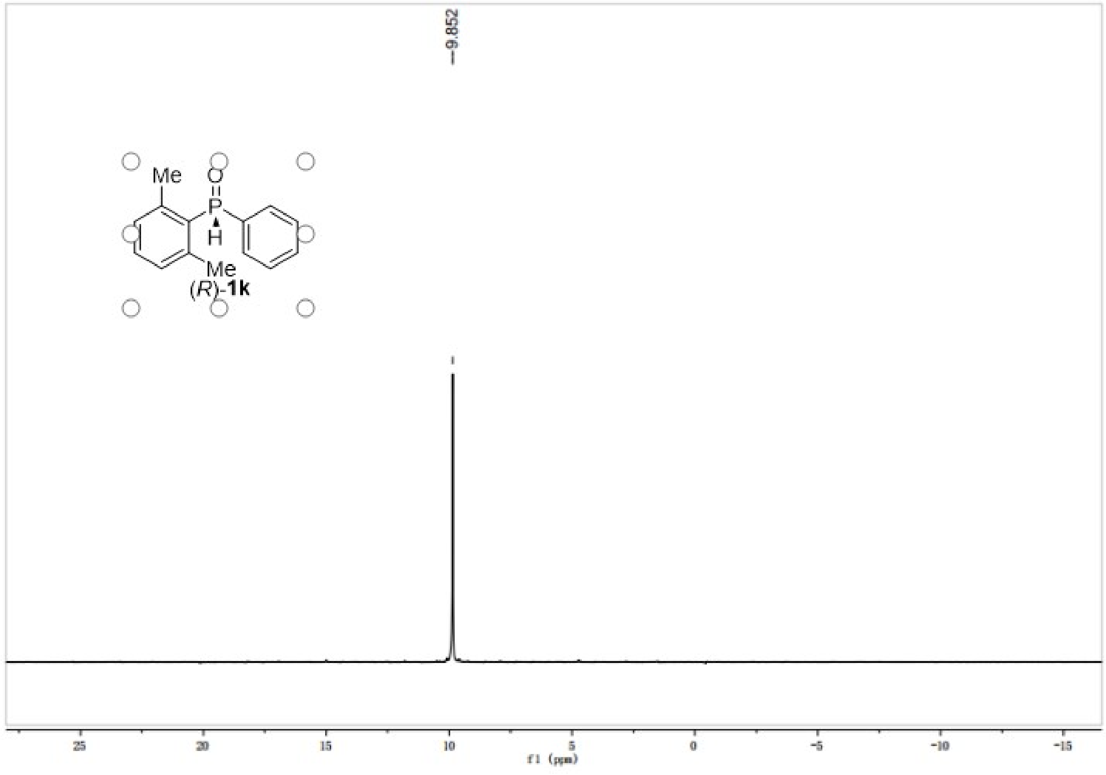

9.852
Me
O
P
H
Me
(R)-1k
25
20
15
10
5
0
-5
-10
-15
f1 (ppm)

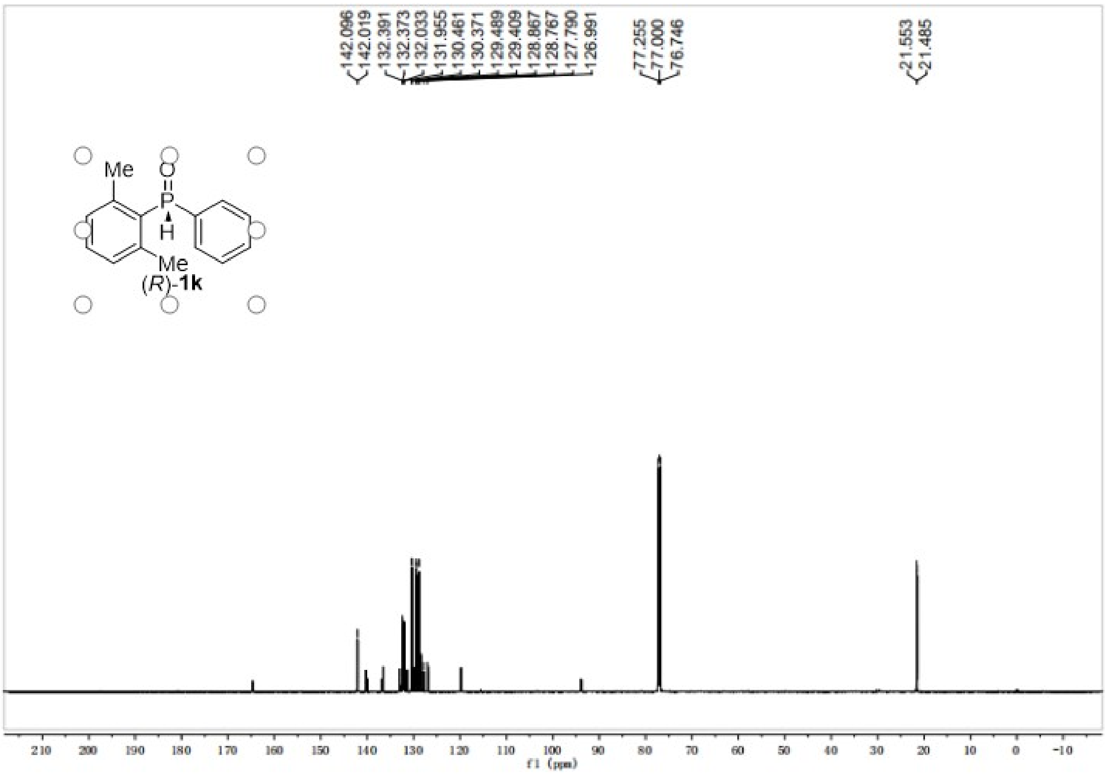
142.096
142.019
132.391
132.373
132.033
131.955
130.461
130.371
129.489
129.409
128.867
128.767
127.790
126.991
77.255
77.000
76.746
21.553
21.485
Me
O
P
H
Me
(R)-1k
f1 (ppm)

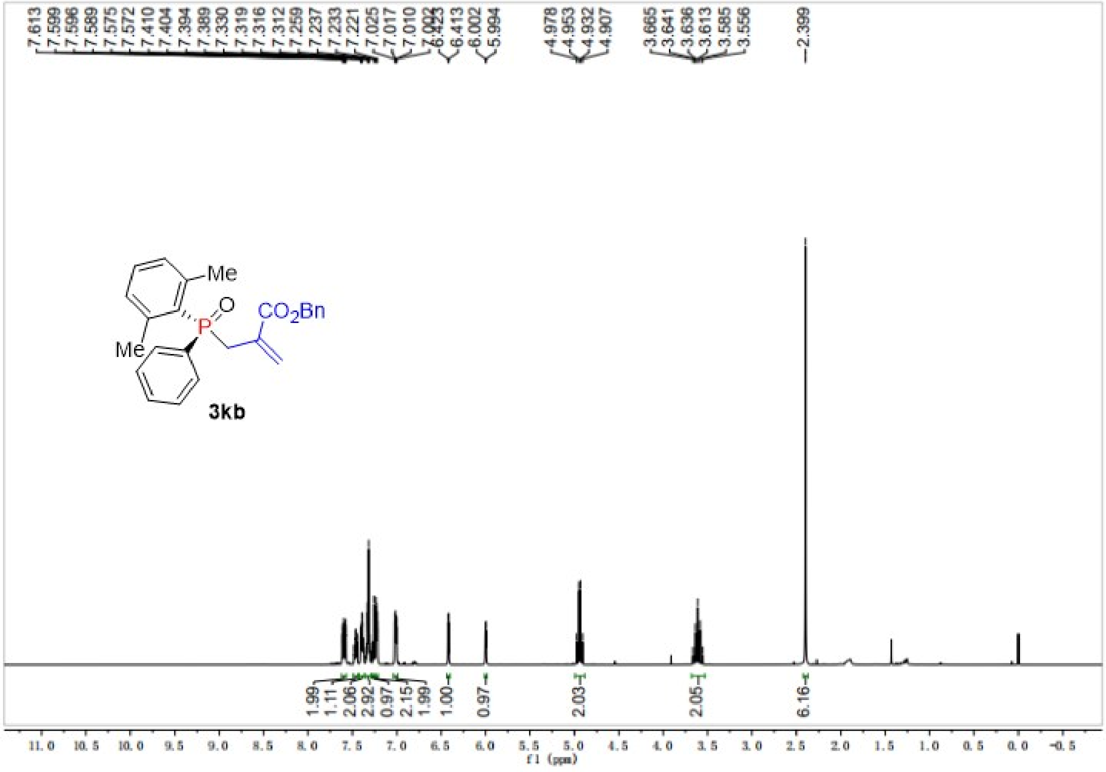
7.613
7.599
7.596
7.589
7.575
7.572
7.410
7.404
7.394
7.389
7.330
7.319
7.316
7.312
7.259
7.237
7.233
7.221
7.025
7.017
7.010
6.413
6.002
5.994
4.978
4.953
4.932
4.907
3.665
3.641
3.636
3.613
3.585
3.556
2.399
Me
O
P
CO2Bn
Me
3kb
1.99
1.11
2.06
2.92
0.97
2.15
1.99
1.00
0.97
2.03
2.05
6.16
f1 (ppm)

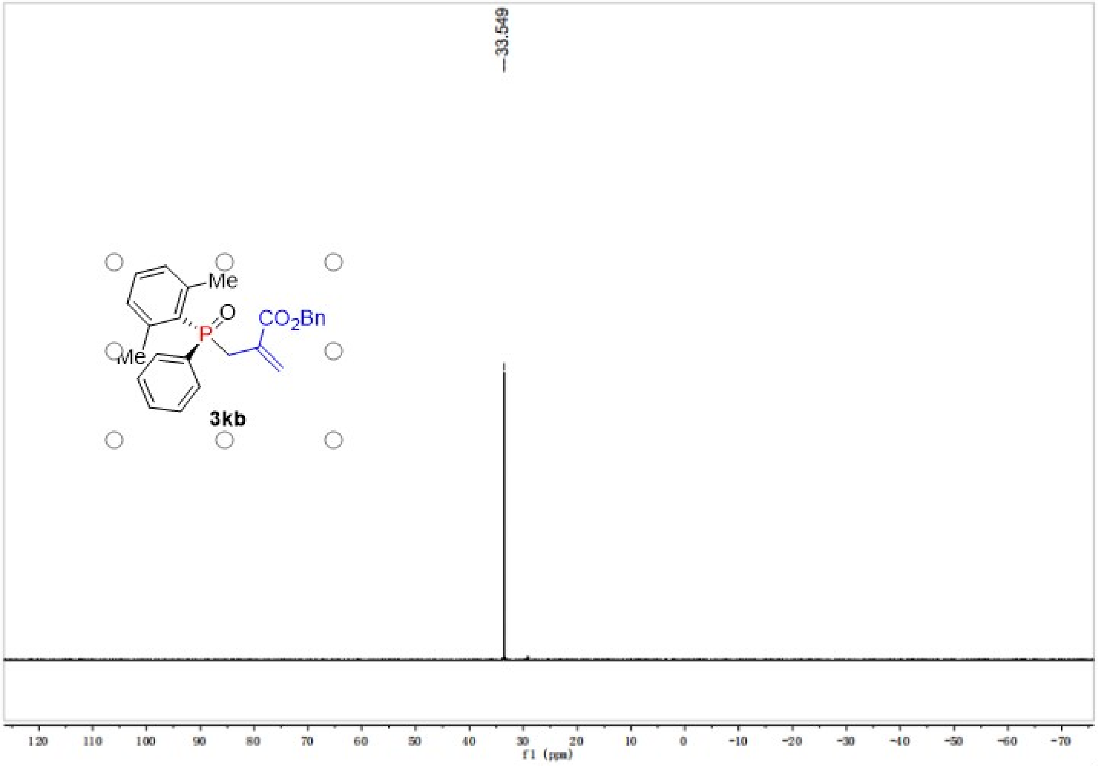

33.549
Me
O
P
CO2Bn
Me
3kb
f1 (ppm)

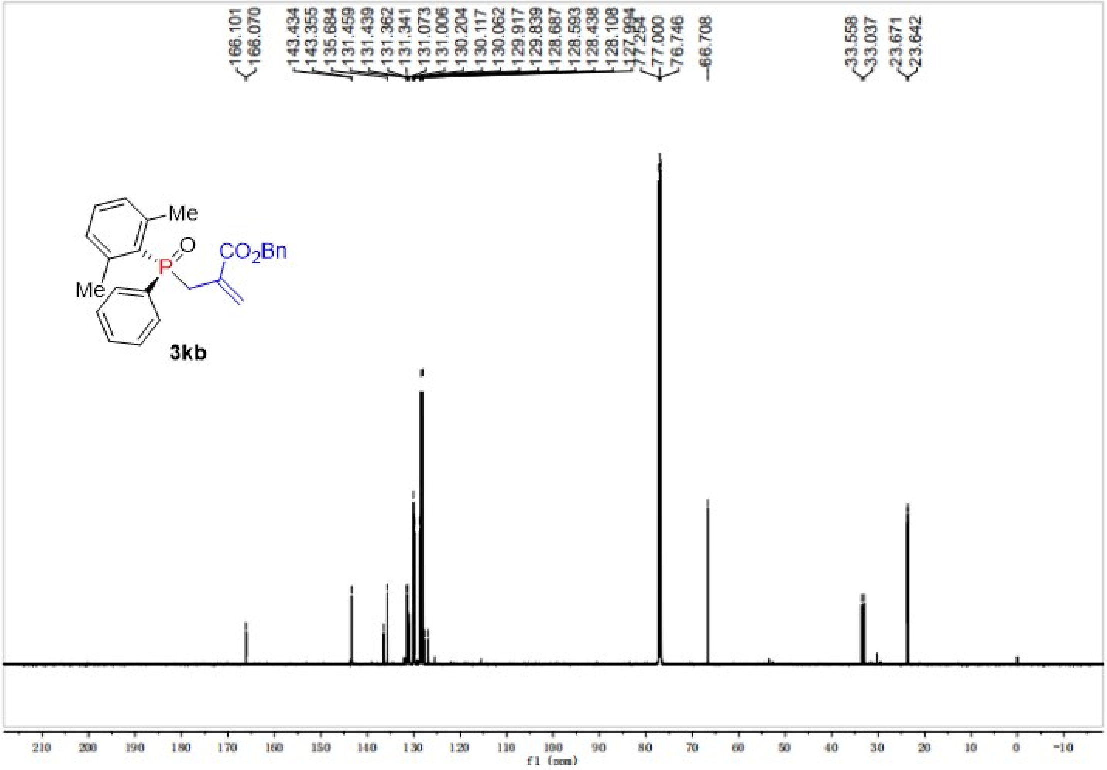

166.101
166.070
143.434
143.355
135.684
131.459
131.439
131.362
131.341
131.073
131.006
130.204
130.117
130.062
129.917
129.839
128.687
128.593
128.438
128.108
77.254
77.000
76.746
66.708
33.558
33.037
23.671
23.642
Me
O
P
CO2Bn
Me
3kb
f1 (ppm)

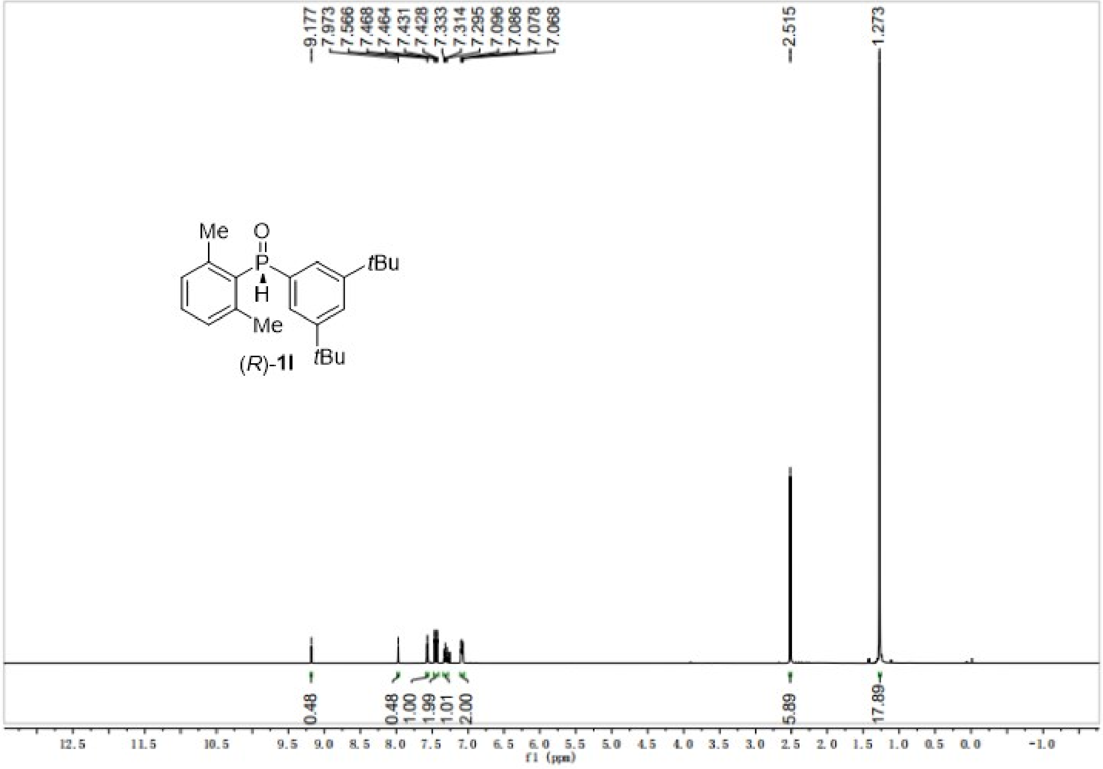
9.177
7.973
7.566
7.468
7.464
7.431
7.428
7.333
7.314
7.295
7.096
7.086
7.078
7.068
2.515
1.273
Me
O
P
H
tBu
Me
(R)-1l
tBu
0.48
0.48
1.00
1.99
1.01
2.00
5.89
17.89
12.5 11.5 10.5 9.5 9.0 8.5 8.0 7.5 7.0 6.5 6.0 5.5 5.0 4.5 4.0 3.5 3.0 2.5 2.0 1.5 1.0 0.5 0.0 -1.0
f1 (ppm)

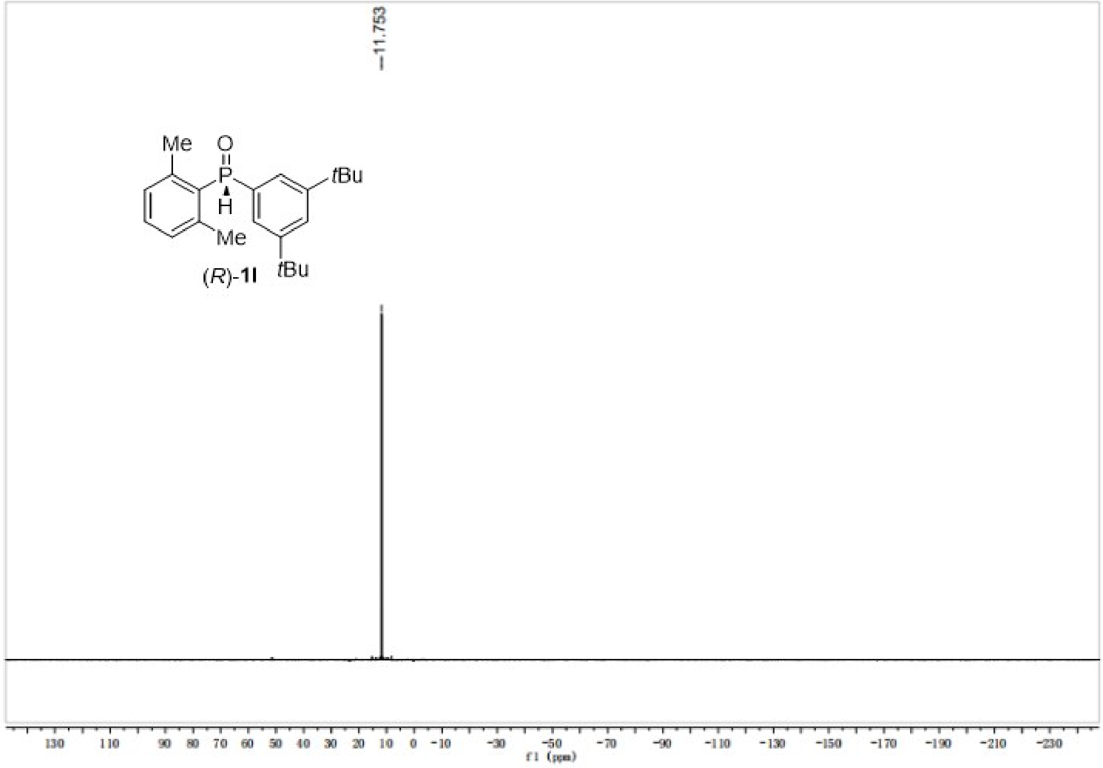
11.753
Me
O
P
H
tBu
Me
(R)-1l
tBu
130 110 90 80 70 60 50 40 30 20 10 0 -10 -30 -50 -70 -90 -110 -130 -150 -170 -190 -210 -230
f1 (ppm)

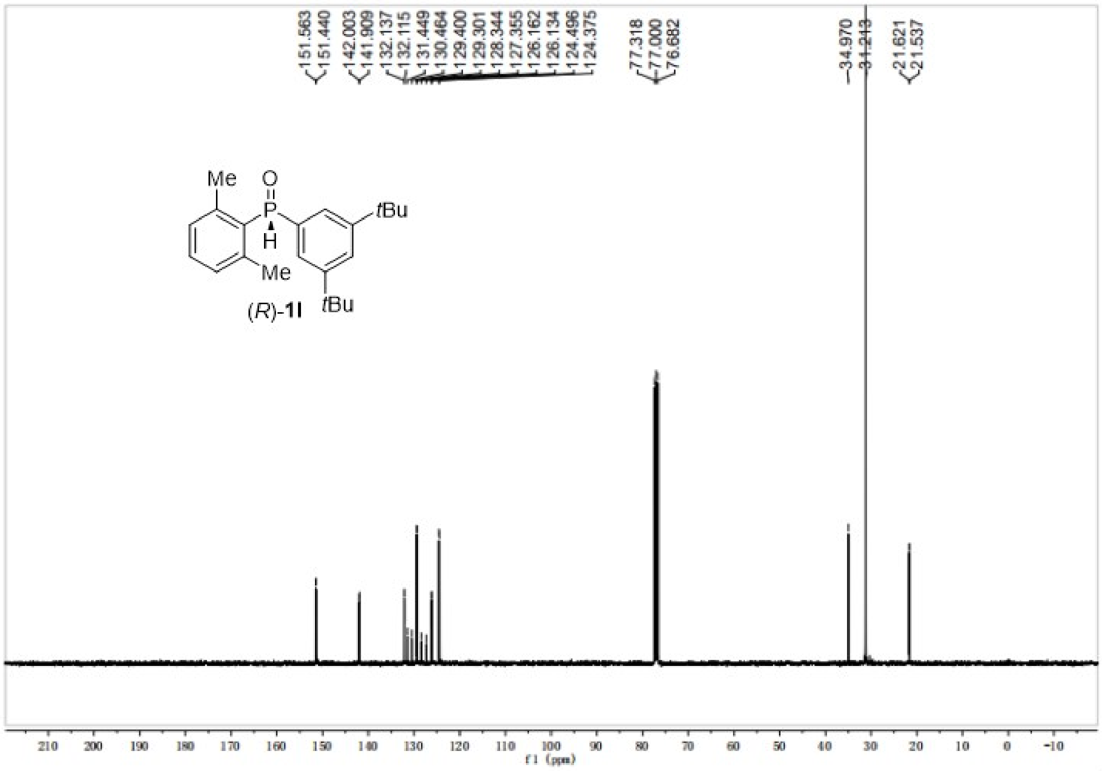
151.563
151.440
142.003
141.909
132.137
132.115
131.449
130.464
129.400
129.301
128.344
127.355
126.162
126.134
124.496
124.375
77.318
77.000
76.682
34.970
31.213
21.621
21.537
Me
O
P
H
tBu
Me
(R)-1l
tBu
f1 (ppm)

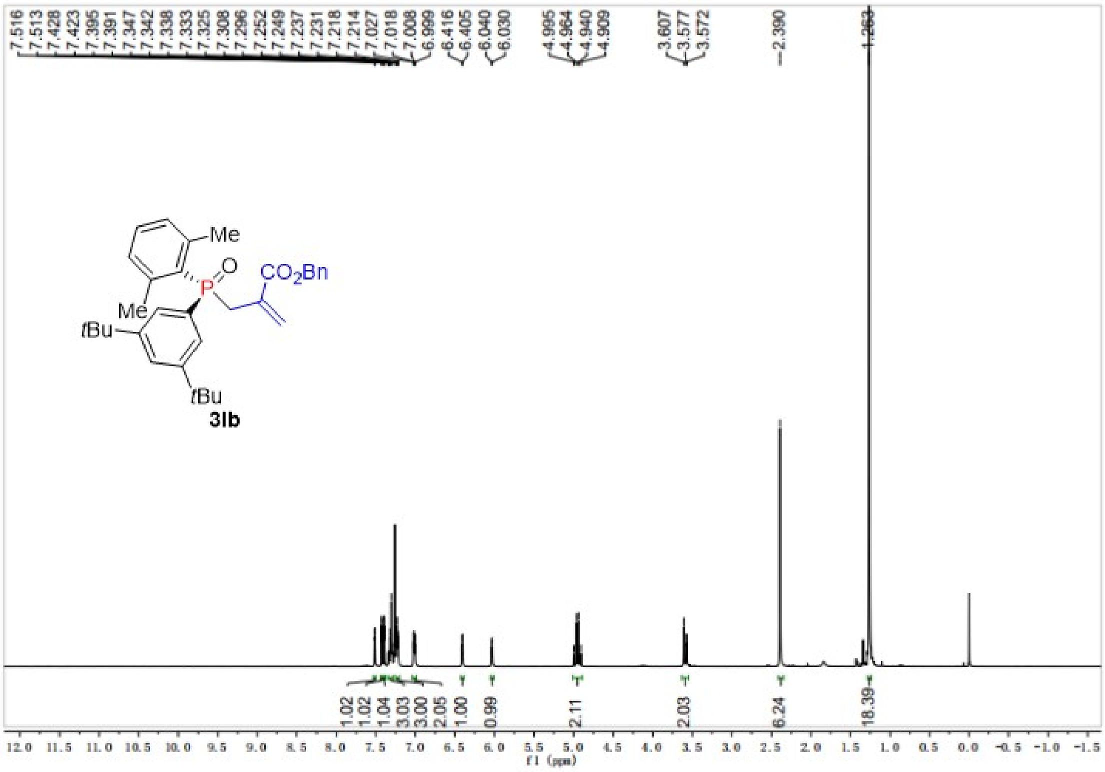
7.516
7.513
7.423
7.395
7.391
7.347
7.342
7.338
7.333
7.325
7.308
7.296
7.252
7.249
7.237
7.231
7.218
7.214
7.027
7.018
7.008
6.999
6.416
6.405
6.040
6.030
4.995
4.964
4.940
4.909
3.607
3.577
3.572
2.390
1.263
Me
O
P
CO2Bn
Me
tBu
tBu
3lb
1.02
1.02
1.04
3.03
3.00
2.05
1.00
0.99
2.11
2.03
6.24
18.39
f1 (ppm)

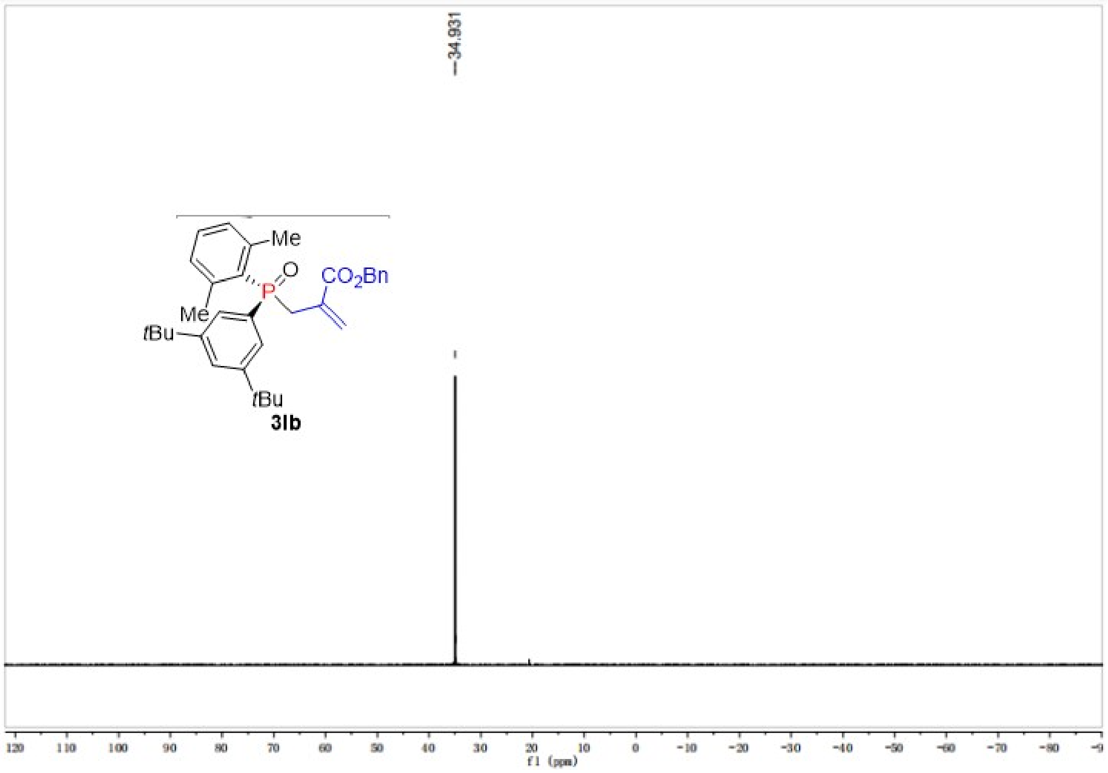

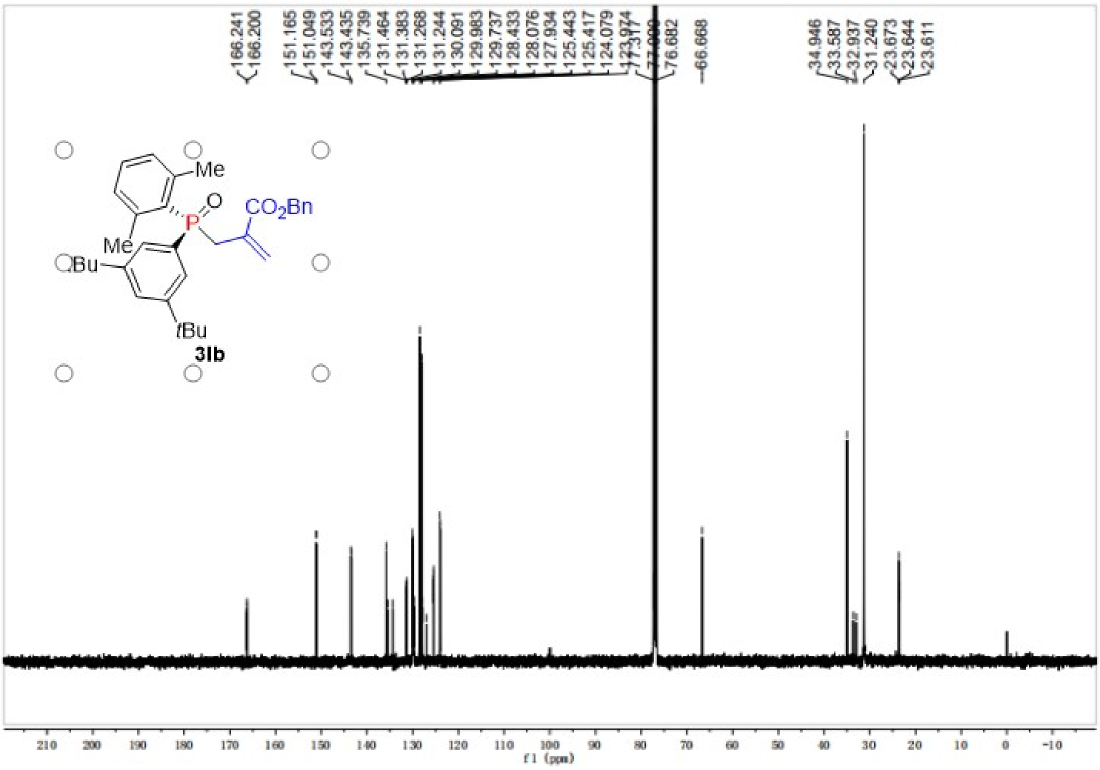

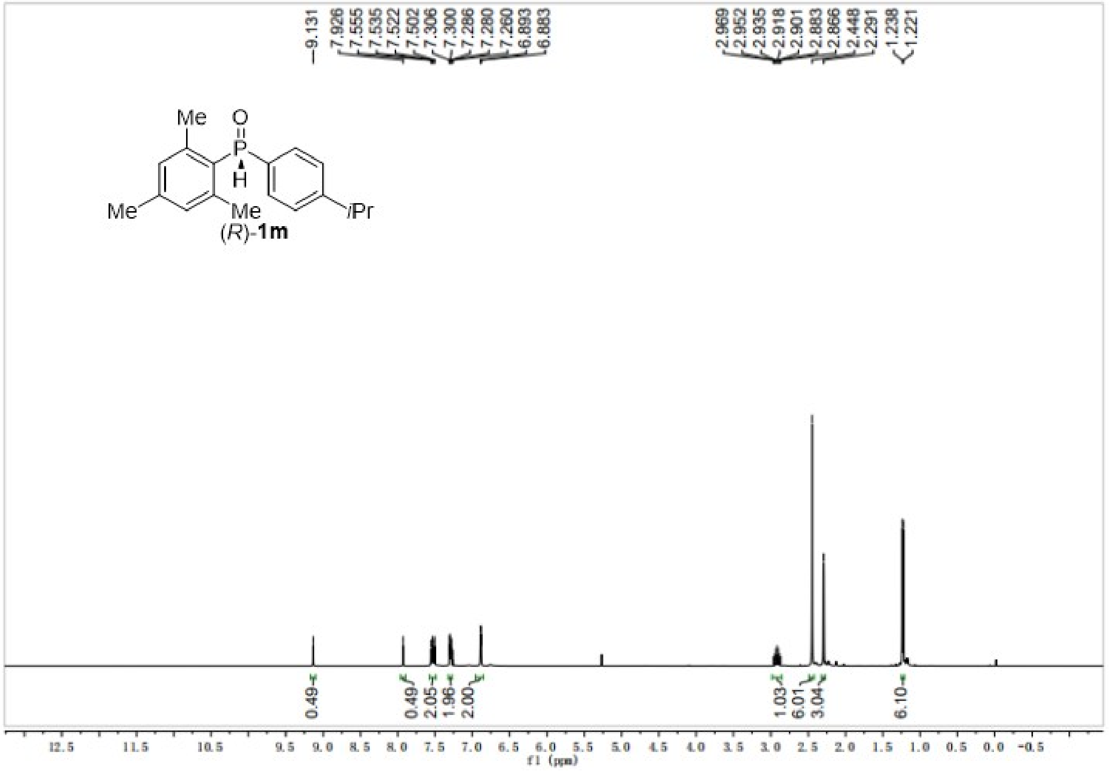

9.131
7.926
7.555
7.535
7.522
7.502
7.306
7.300
7.286
7.280
7.260
6.893
6.883
2.969
2.952
2.935
2.918
2.901
2.883
2.866
2.448
2.291
1.238
1.221
Me
O
P
H
Me
Me
iPr
(R)-1m
0.49
0.49
2.05
1.96
2.00
1.03
6.01
3.04
6.10
f1 (ppm)

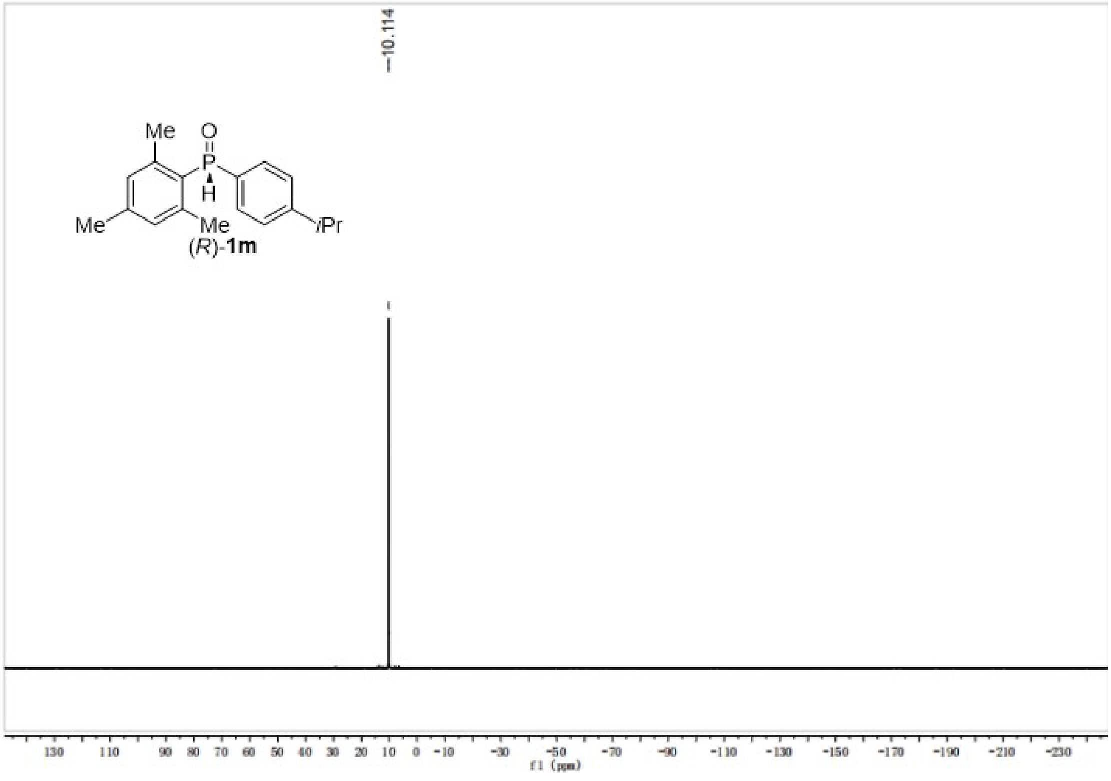

10.114
Me
O
P
H
Me
Me
iPr
(R)-1m
f1 (ppm)

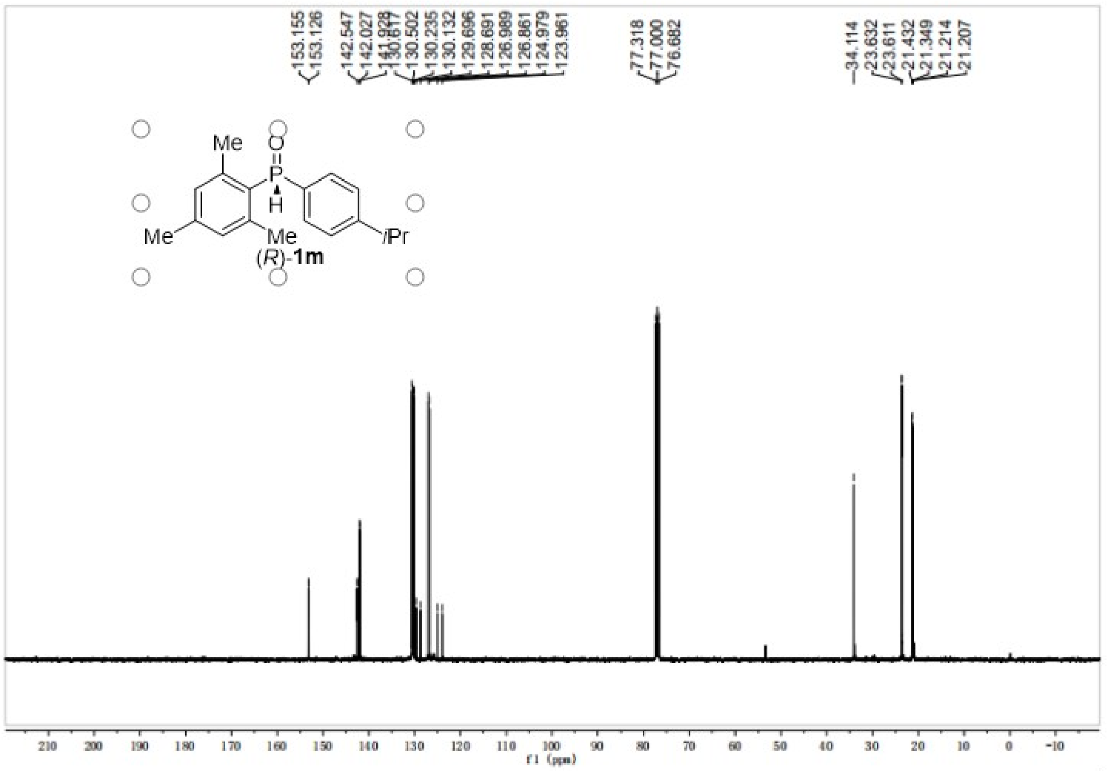
(R)-1m

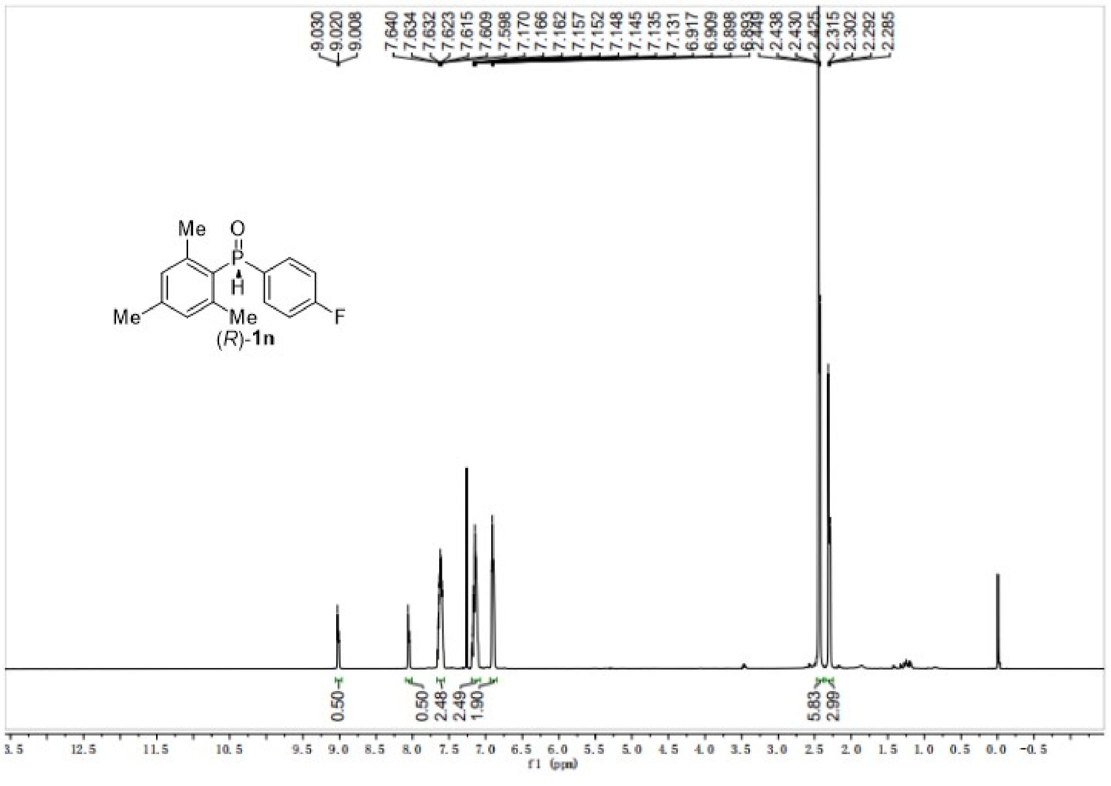
(R)-1n

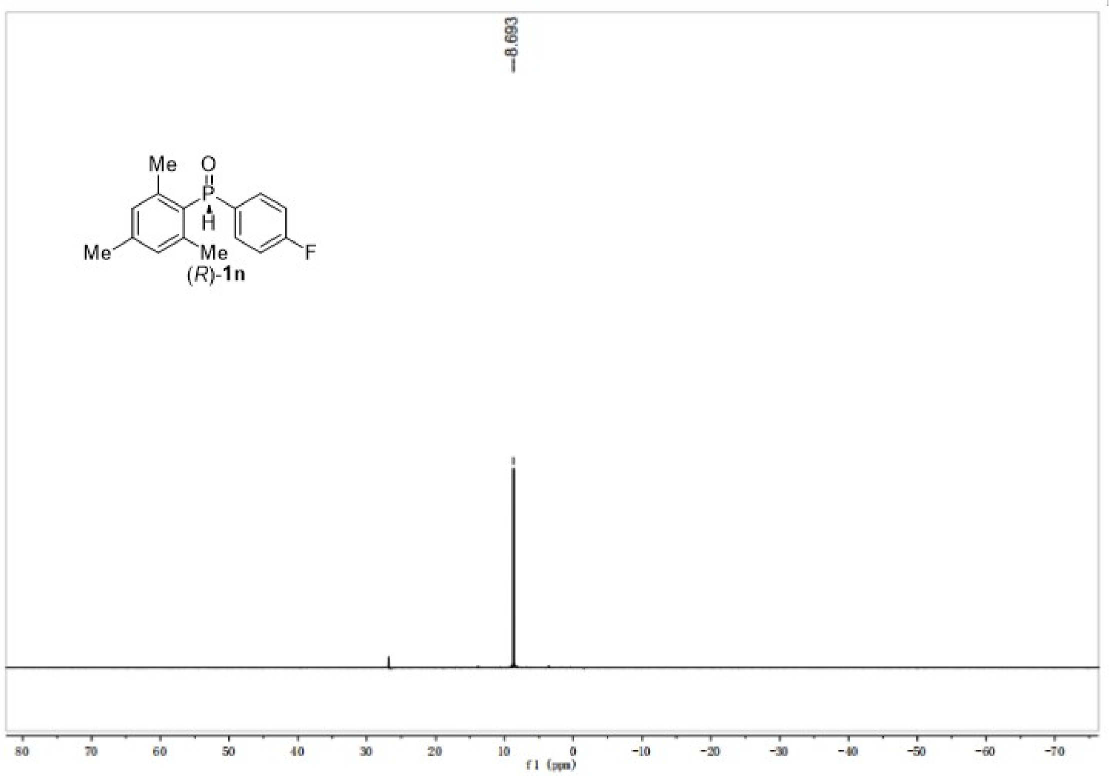
-8.693
Me
O
P
H
Me
Me
F
(R)-1n
80
70
60
50
40
30
20
10
0
-10
-20
-30
-40
-50
-60
-70
f1 (ppm)

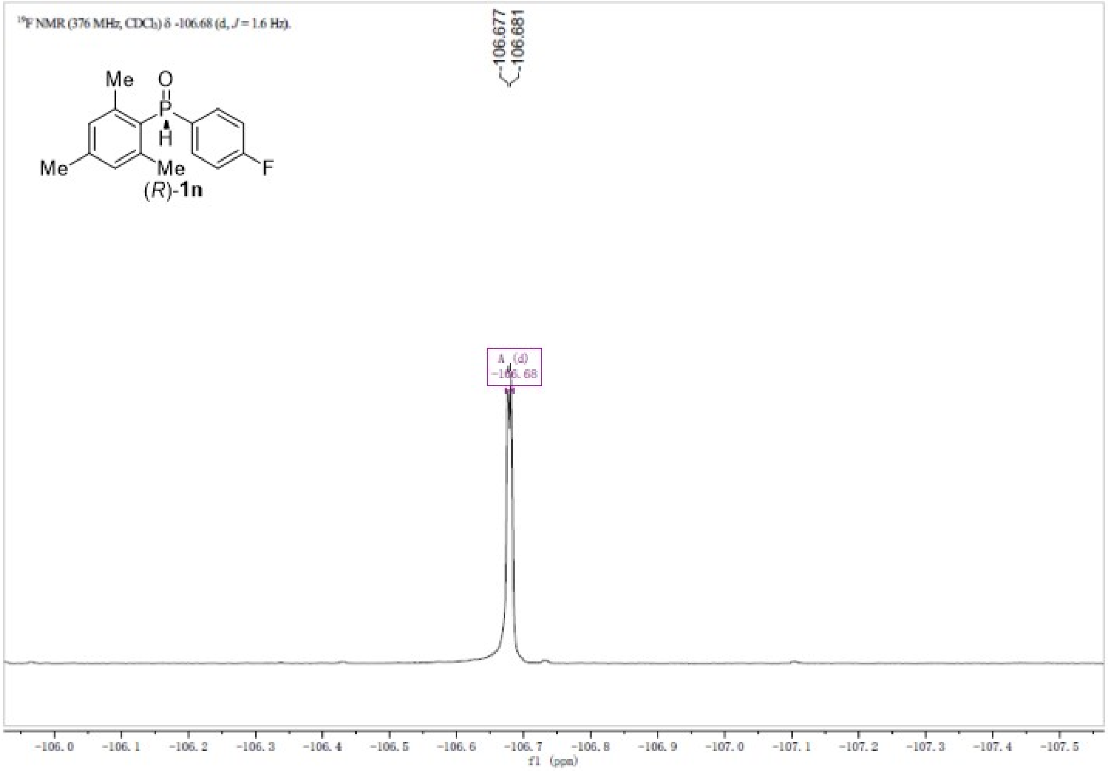
19F NMR (376 MHz, CDCl3) δ -106.68 (d, J = 1.6 Hz).
-106.677
-106.681
Me
O
P
H
Me
Me
F
(R)-1n
A (d)
-106.68
-106.0
-106.1
-106.2
-106.3
-106.4
-106.5
-106.6
-106.7
-106.8
-106.9
-107.0
-107.1
-107.2
-107.3
-107.4
-107.5
f1 (ppm)

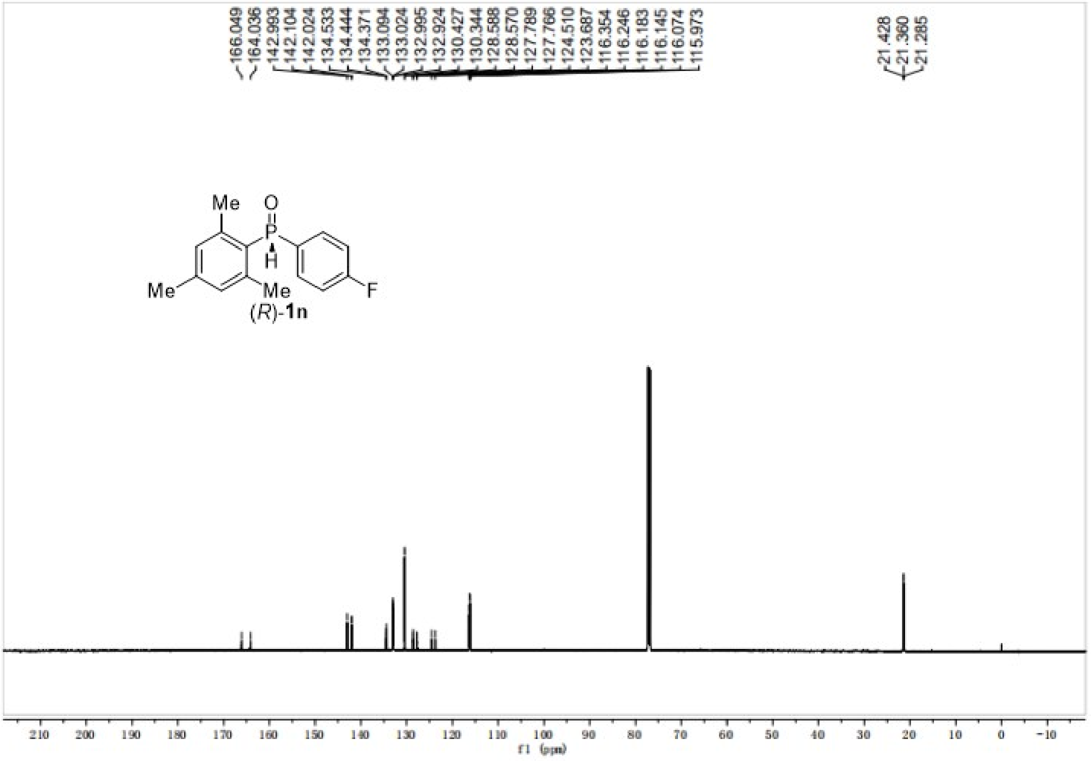
166.049
164.036
142.993
142.104
142.024
134.533
134.444
134.371
133.094
133.024
132.995
132.924
130.427
130.344
128.588
128.570
127.789
127.766
124.510
123.687
116.354
116.246
116.183
116.145
116.074
115.973
21.428
21.360
21.285
Me
O
P
H
Me
Me
F
(R)-1n
f1 (ppm)

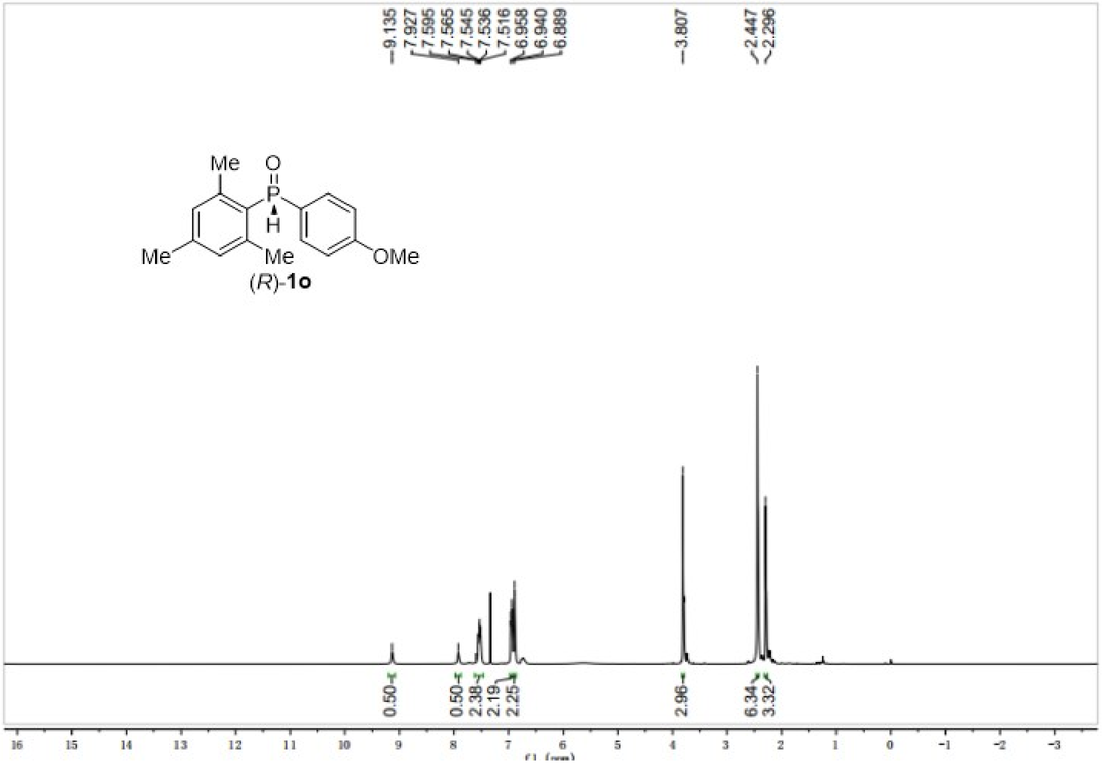
9.135
7.927
7.595
7.565
7.545
7.536
7.516
6.958
6.940
6.889
3.807
2.447
2.296
Me
O
P
H
Me
Me
OMe
(R)-1o
0.50
0.50
2.38
2.19
2.25
2.96
6.34
3.32

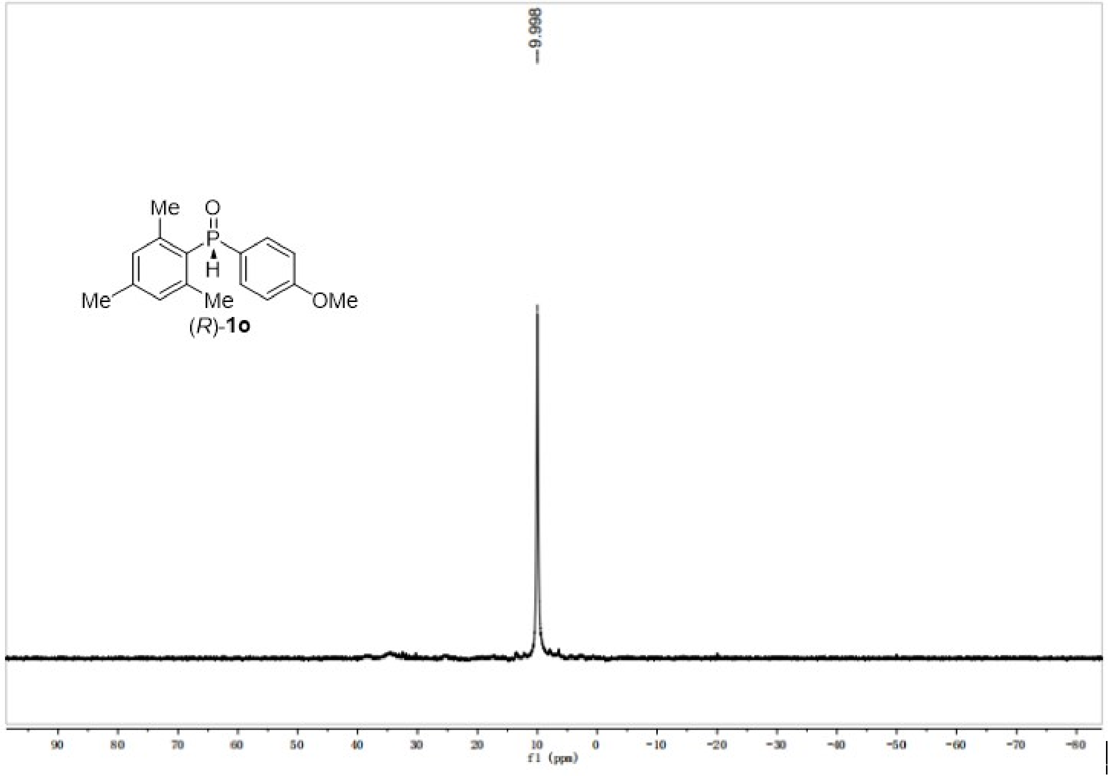

-9.998
Me
O
P
H
Me
Me
OMe
(R)-1o
f1 (ppm)

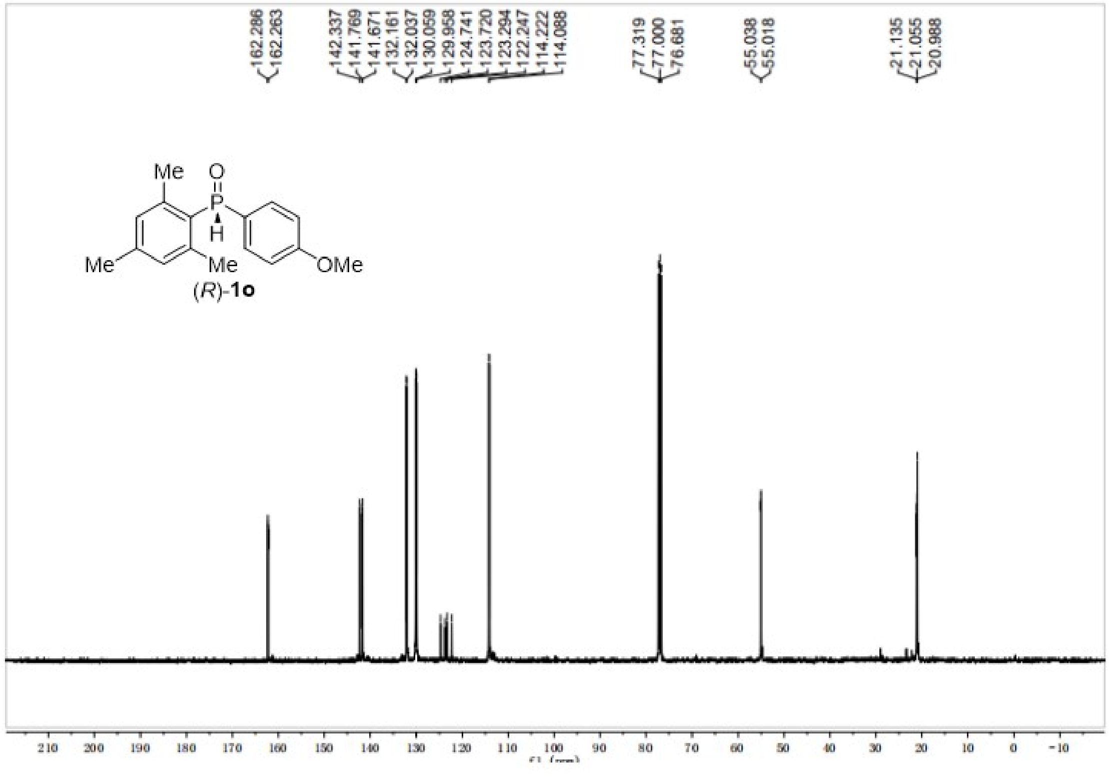

162.286
162.263
142.337
141.769
141.671
132.161
132.037
130.059
129.958
124.741
123.720
123.294
122.247
114.222
114.088
77.319
77.000
76.681
55.038
55.018
21.135
21.055
20.988
Me
O
P
H
Me
Me
OMe
(R)-1o

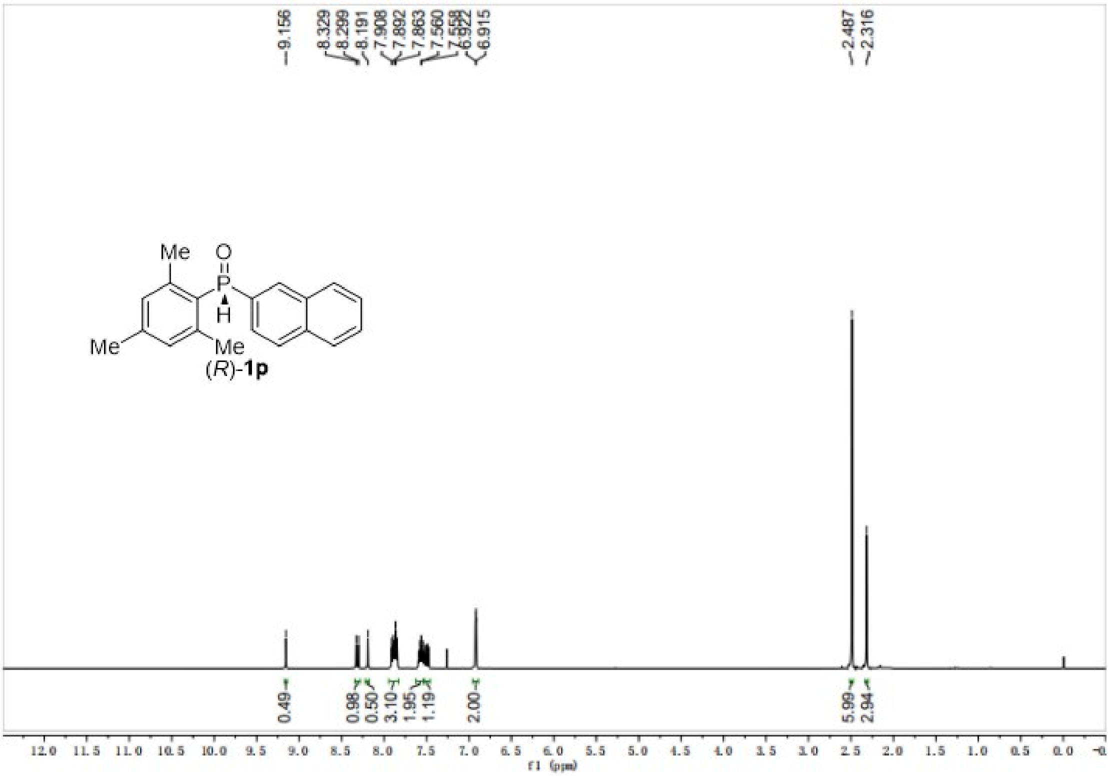
9.156
8.329
8.299
8.191
7.908
7.892
7.863
7.560
6.922
6.915
2.487
2.316
Me
O
P
H
Me
Me
(R)-1p
0.49
0.98
0.50
3.10
1.95
1.19
2.00
5.99
2.94
f1 (ppm)

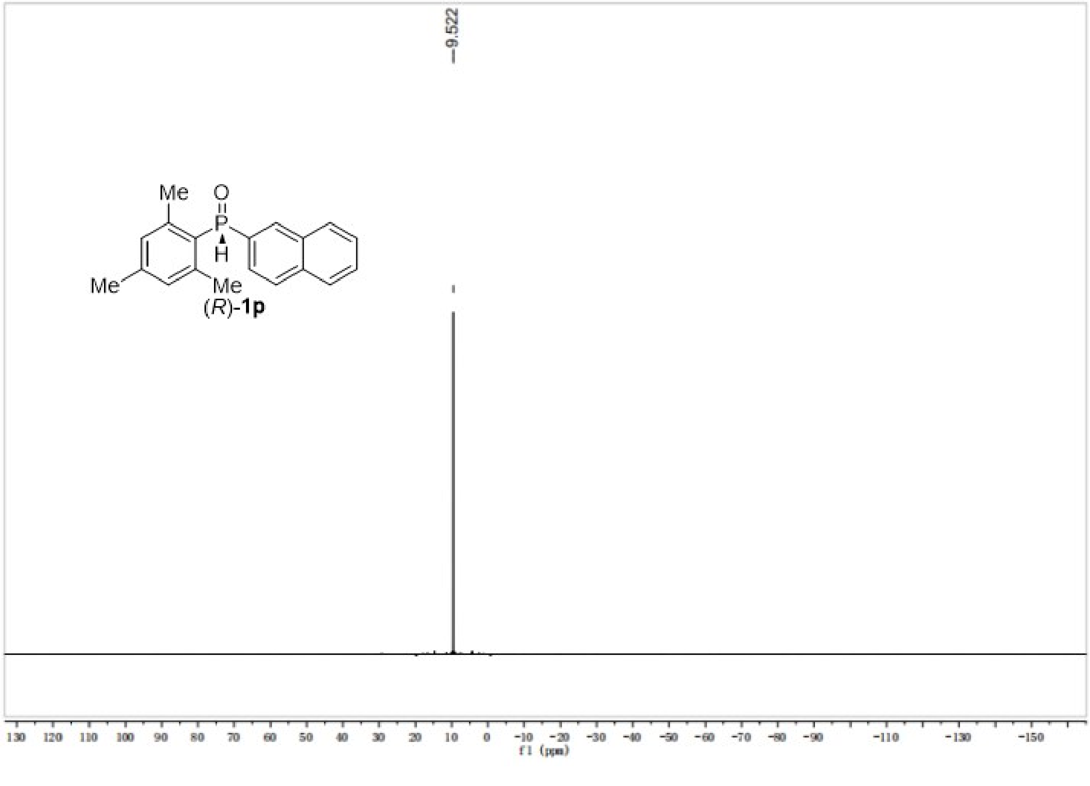
9.522
Me
O
P
H
Me
Me
(R)-1p
f1 (ppm)

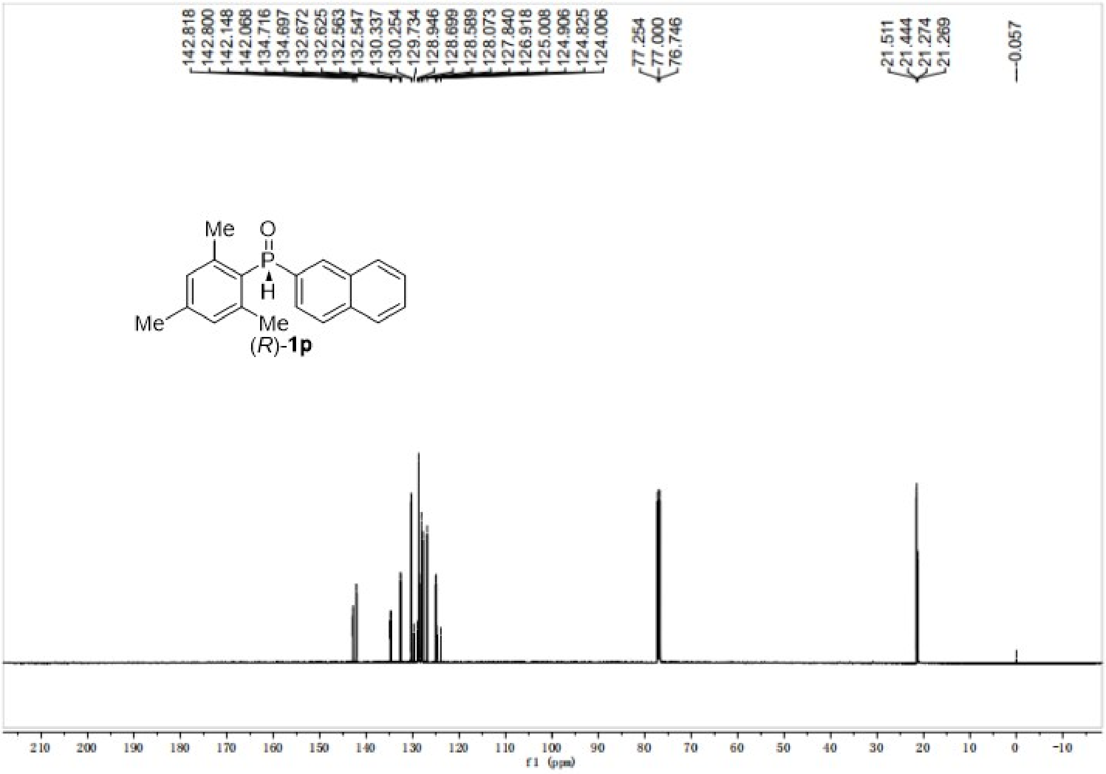

(R)-**1p**

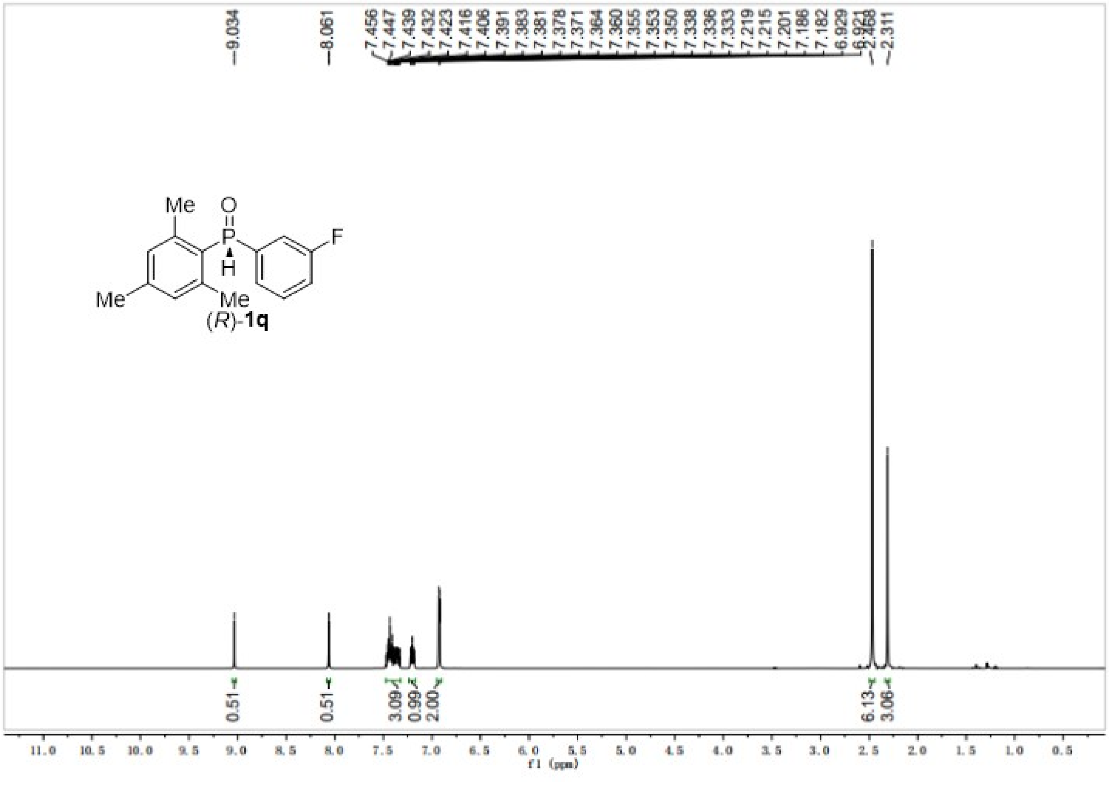

(R)-**1q**

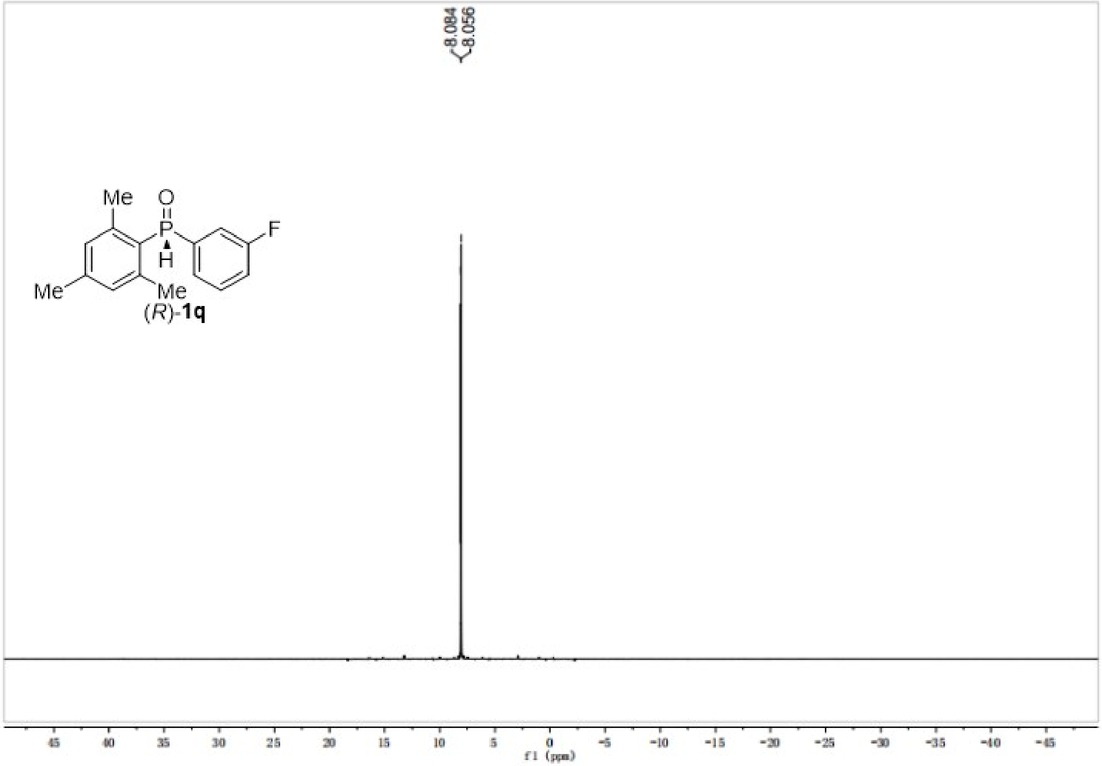
8.084
8.056
Me
O
P
H
F
Me
Me
(R)-1q
45
40
35
30
25
20
15
10
5
0
-5
-10
-15
-20
-25
-30
-35
-40
-45
f1 (ppm)

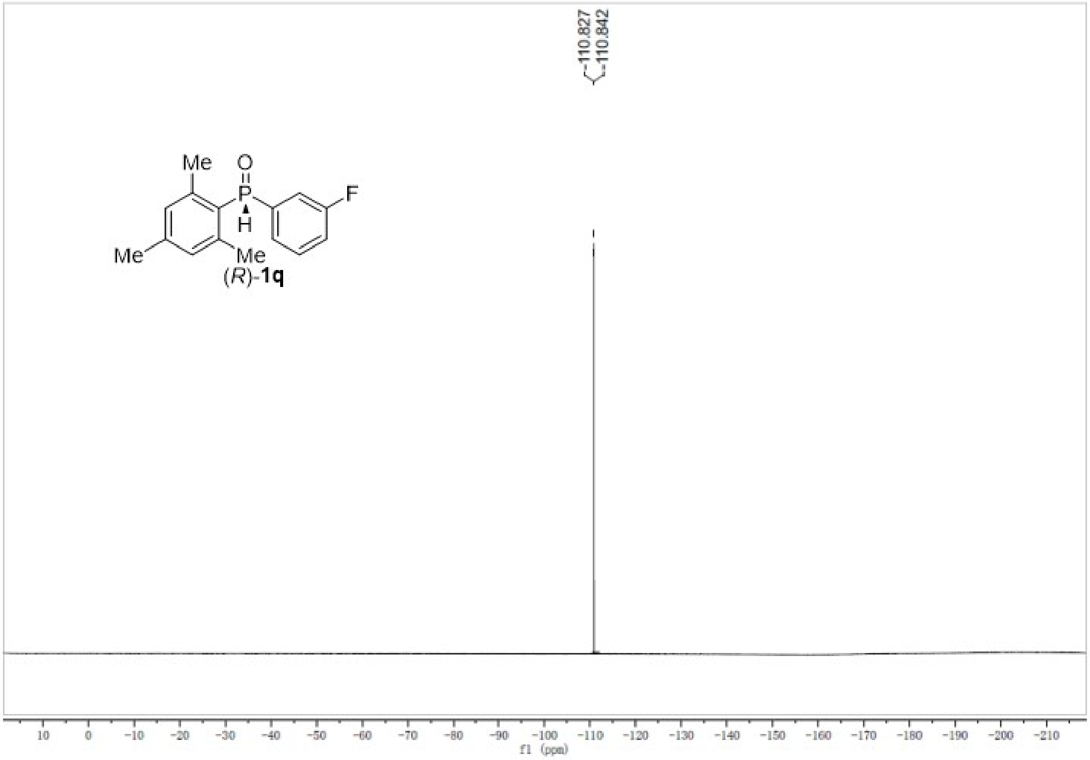
-110.827
-110.842
Me
O
P
H
F
Me
Me
(R)-1q
10
0
-10
-20
-30
-40
-50
-60
-70
-80
-90
-100
-110
-120
-130
-140
-150
-160
-170
-180
-190
-200
-210
f1 (ppm)

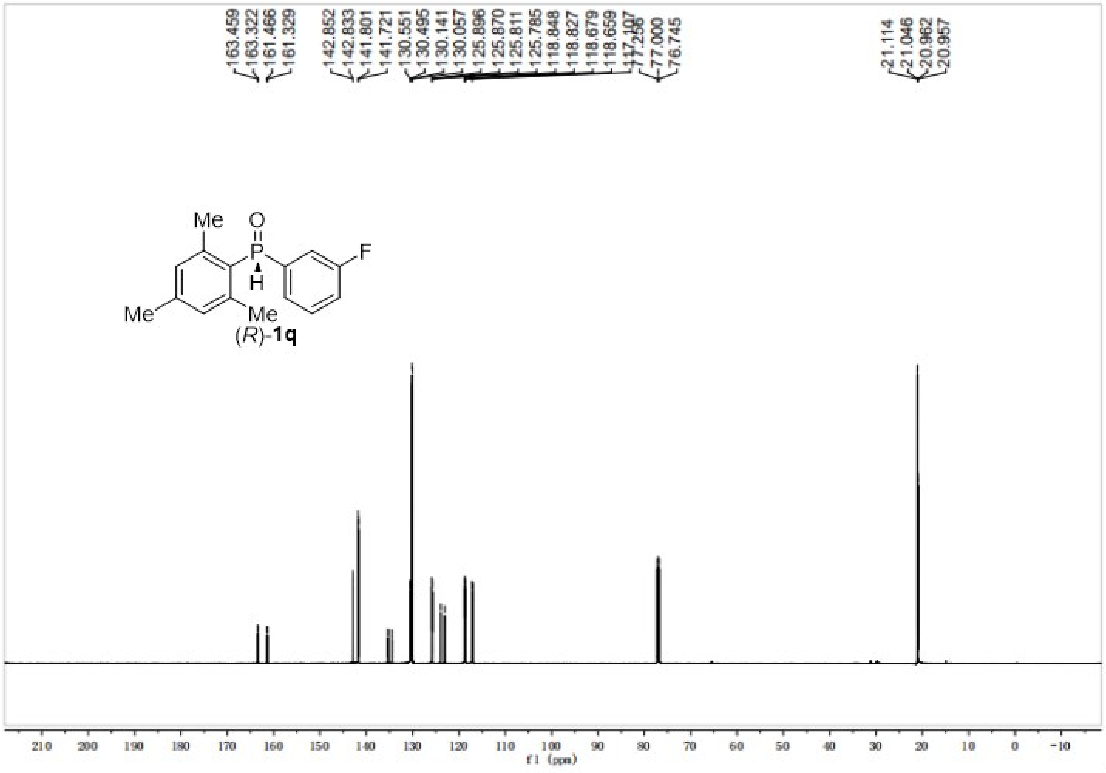

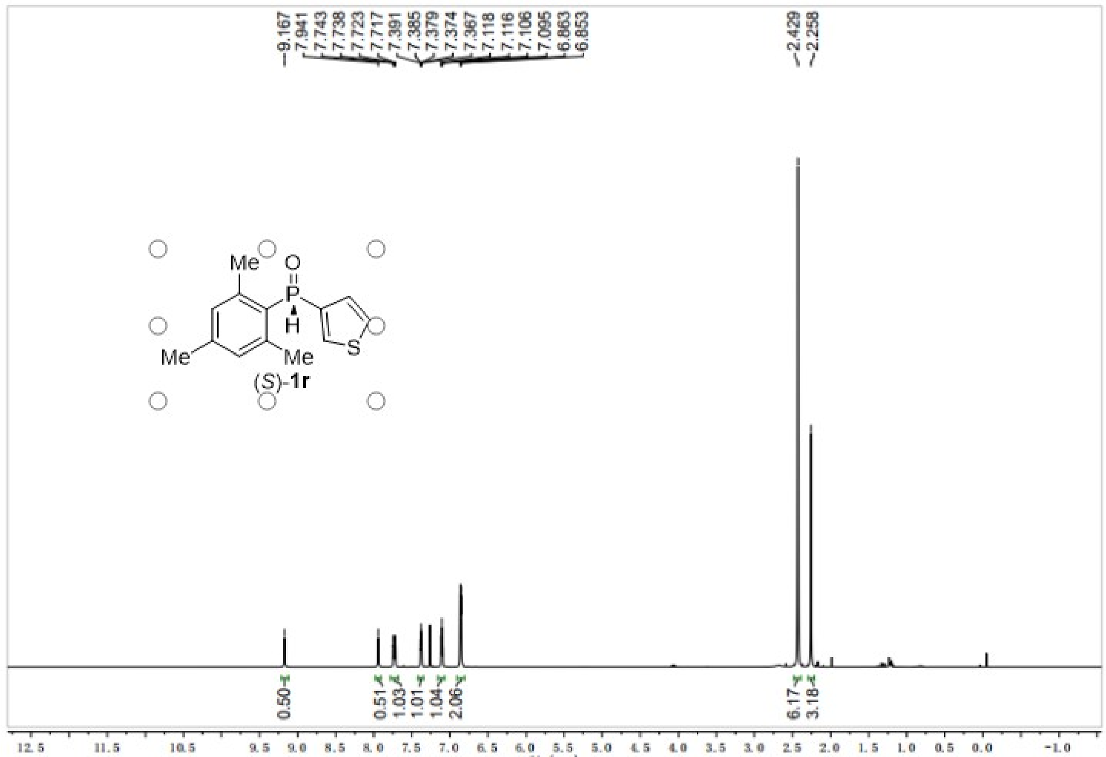

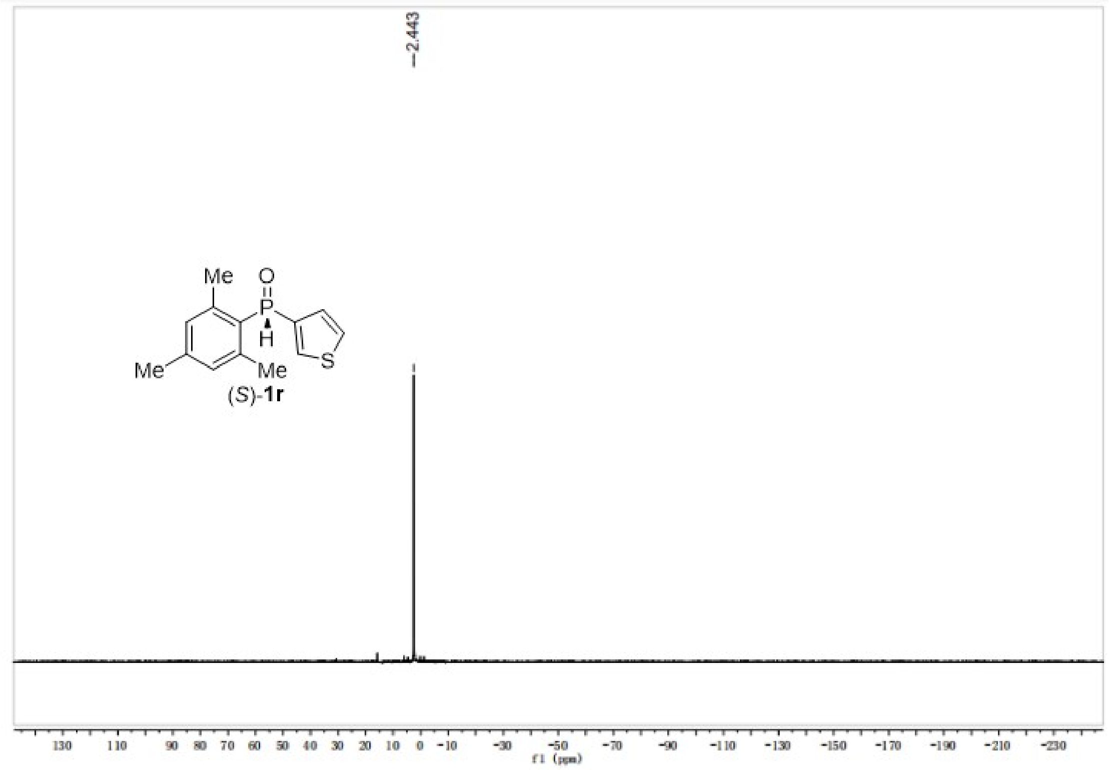

-2.443
Me
O
P
H
Me
Me
S
(S)-1r
130
110
90
80
70
60
50
40
30
20
10
0
-10
-30
-50
-70
-90
-110
-130
-150
-170
-190
-210
-230
f1 (ppm)

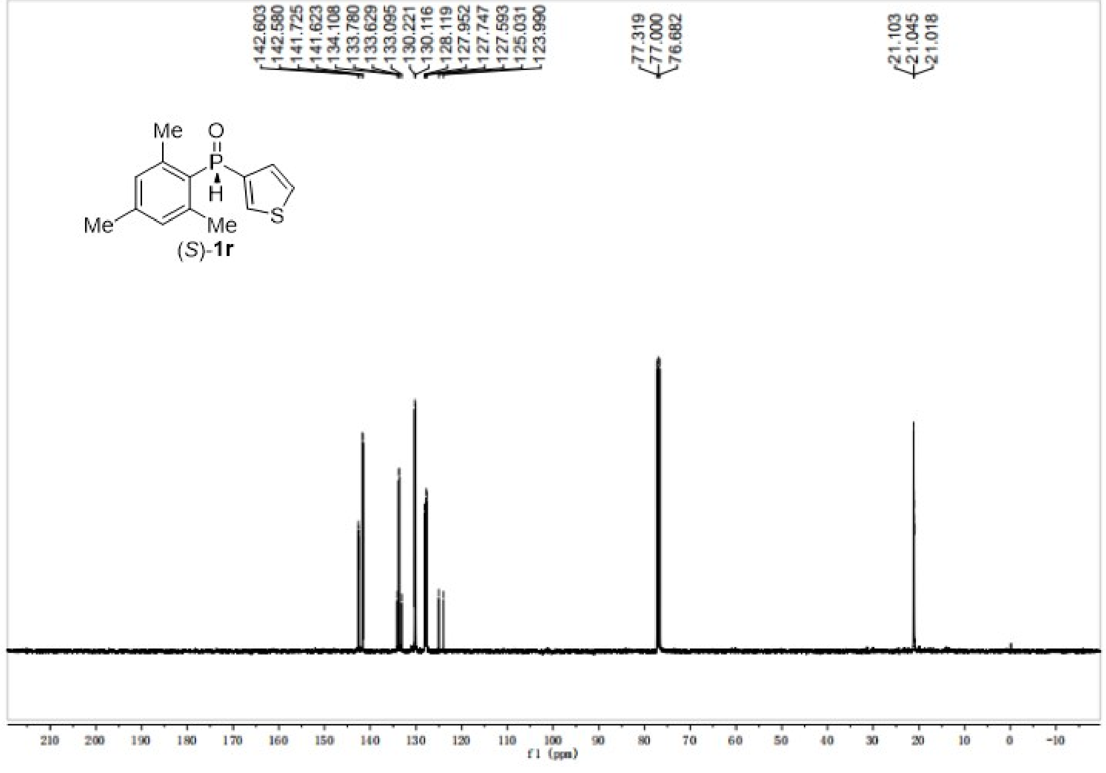

142.603
142.580
141.725
141.623
134.108
133.780
133.629
133.095
130.221
130.116
128.119
127.952
127.747
127.593
125.031
123.990
77.319
77.000
76.682
21.103
21.045
21.018
Me
O
P
H
Me
Me
S
(S)-1r
210
200
190
180
170
160
150
140
130
120
110
100
90
80
70
60
50
40
30
20
10
0
-10
f1 (ppm)

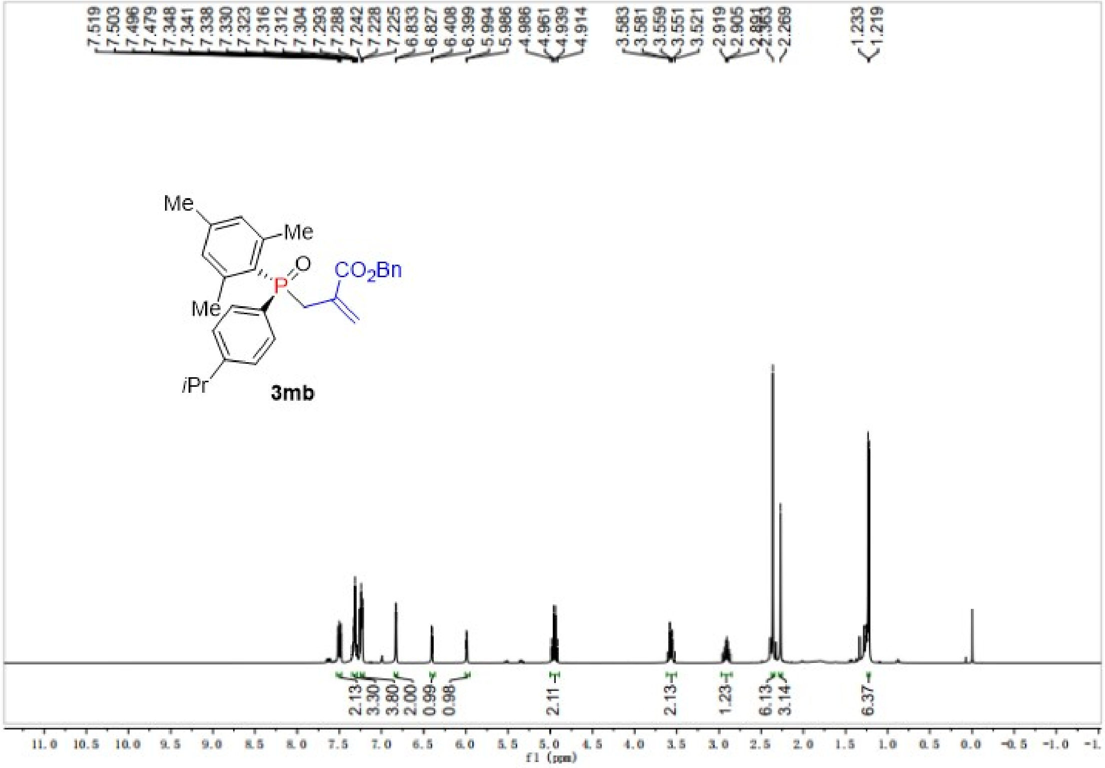
Me
Me
O
CO2Bn
P
Me
iPr
3mb
f1 (ppm)

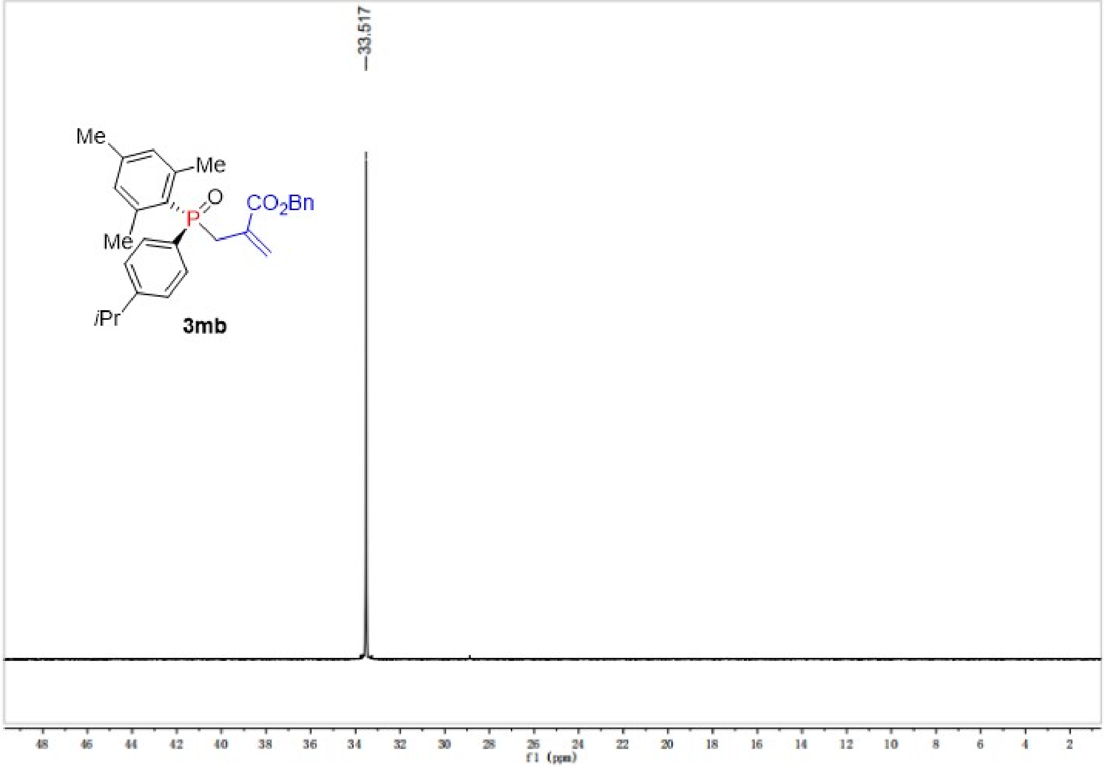
—33.517
Me
Me
O
CO2Bn
P
Me
iPr
3mb
f1 (ppm)

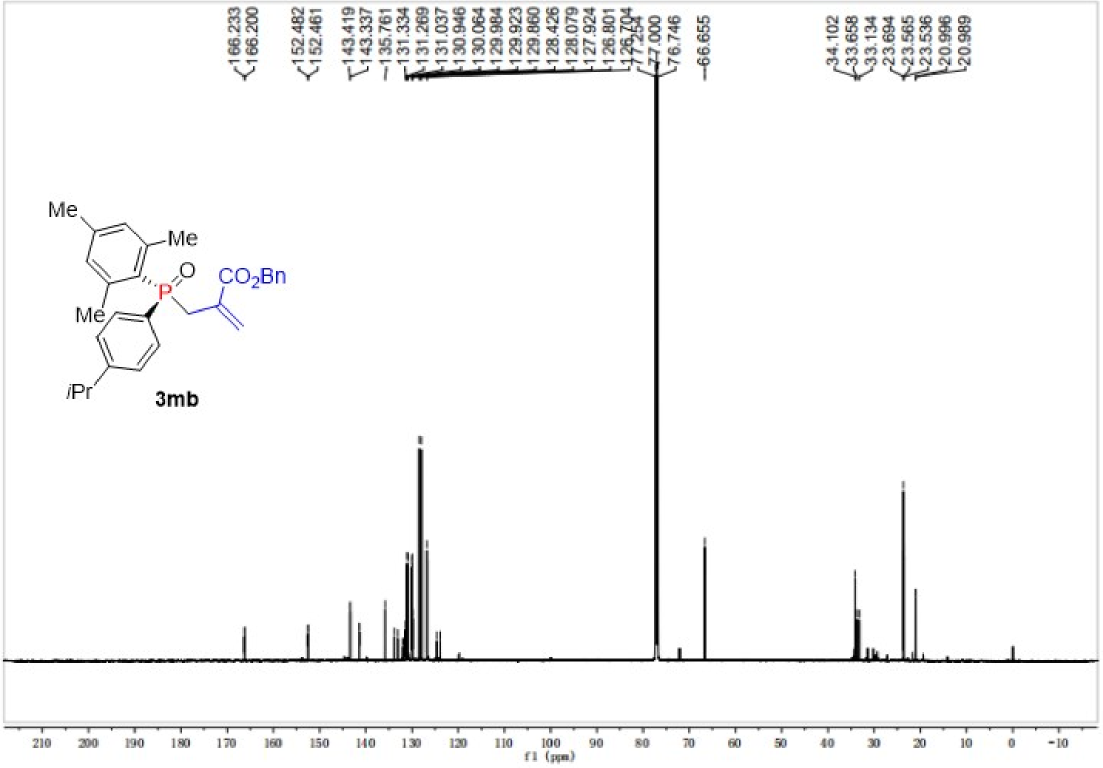
166.233
166.200
152.482
152.461
143.419
143.337
135.761
131.334
131.269
131.037
130.946
130.064
129.984
129.923
129.860
128.426
128.079
127.924
126.801
126.704
77.254
77.000
76.746
66.655
34.102
33.658
33.134
23.694
23.565
23.536
20.996
20.989
Me
Me
O
P
CO2Bn
Me
iPr
3mb
f1 (ppm)

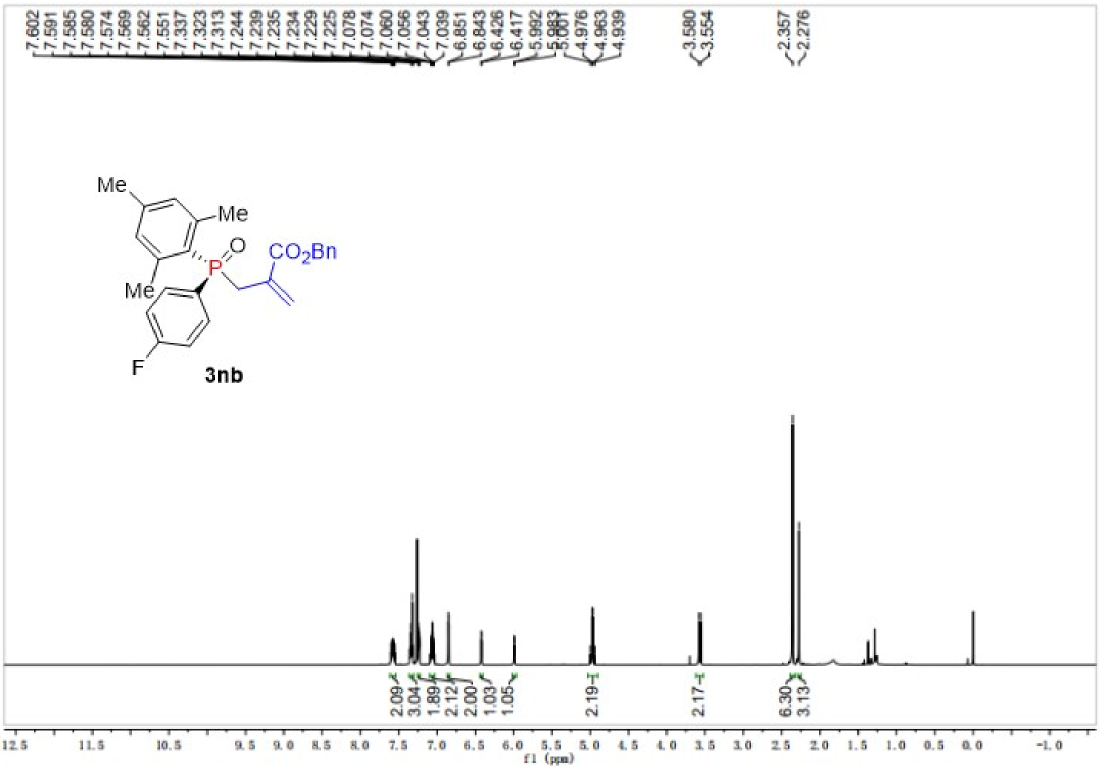
7.602
7.591
7.585
7.580
7.574
7.569
7.562
7.551
7.337
7.323
7.313
7.244
7.239
7.235
7.234
7.229
7.225
7.078
7.074
7.060
7.056
7.043
7.039
6.851
6.843
6.426
6.417
5.992
5.983
5.001
4.976
4.963
4.939
3.580
3.554
2.357
2.276
Me
Me
O
P
CO2Bn
Me
F
3nb
2.09
3.04
1.89
2.12
2.00
1.03
1.05
2.19
2.17
6.30
3.13
f1 (ppm)

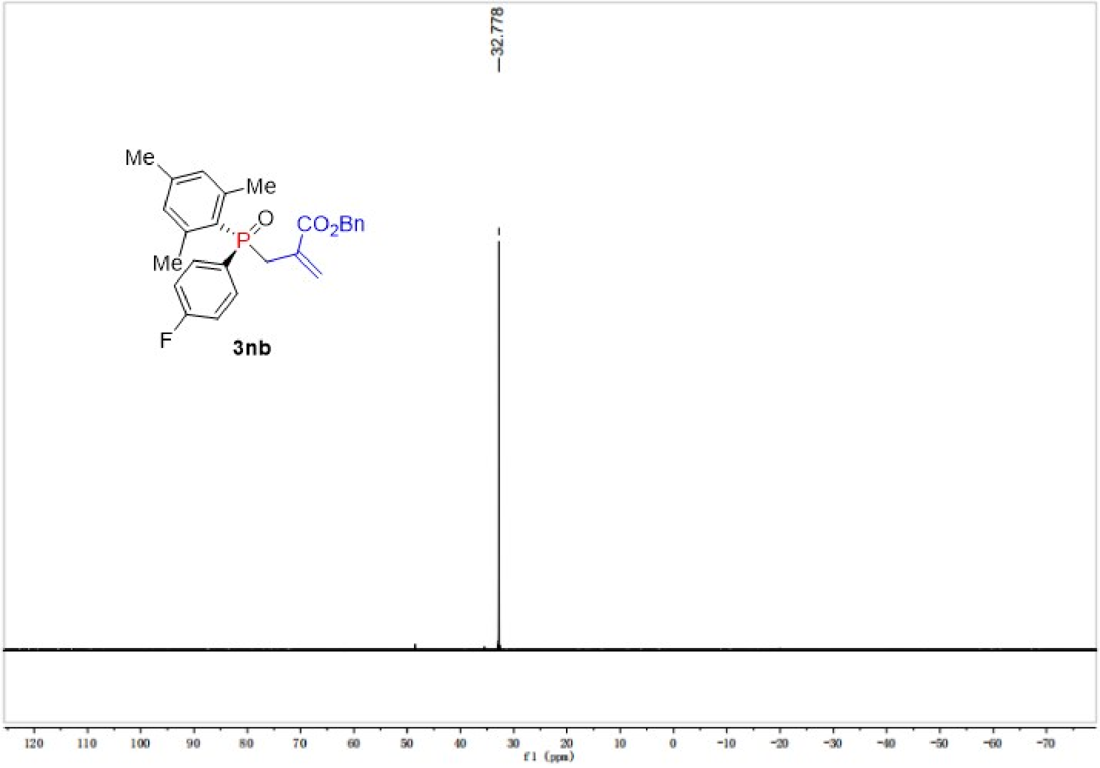
-32.778
Me
Me
Me
O
P
CO2Bn
F
3nb
f1 (ppm)

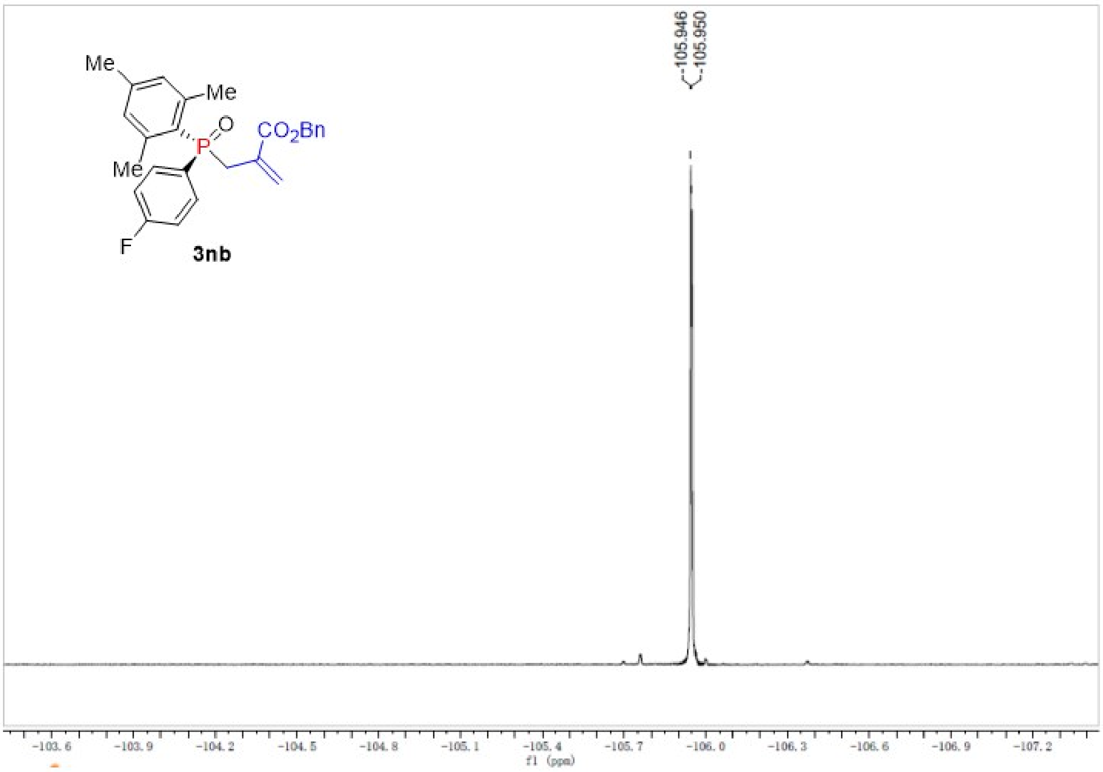
-105.946
-105.950
Me
Me
Me
O
P
CO2Bn
F
3nb
f1 (ppm)

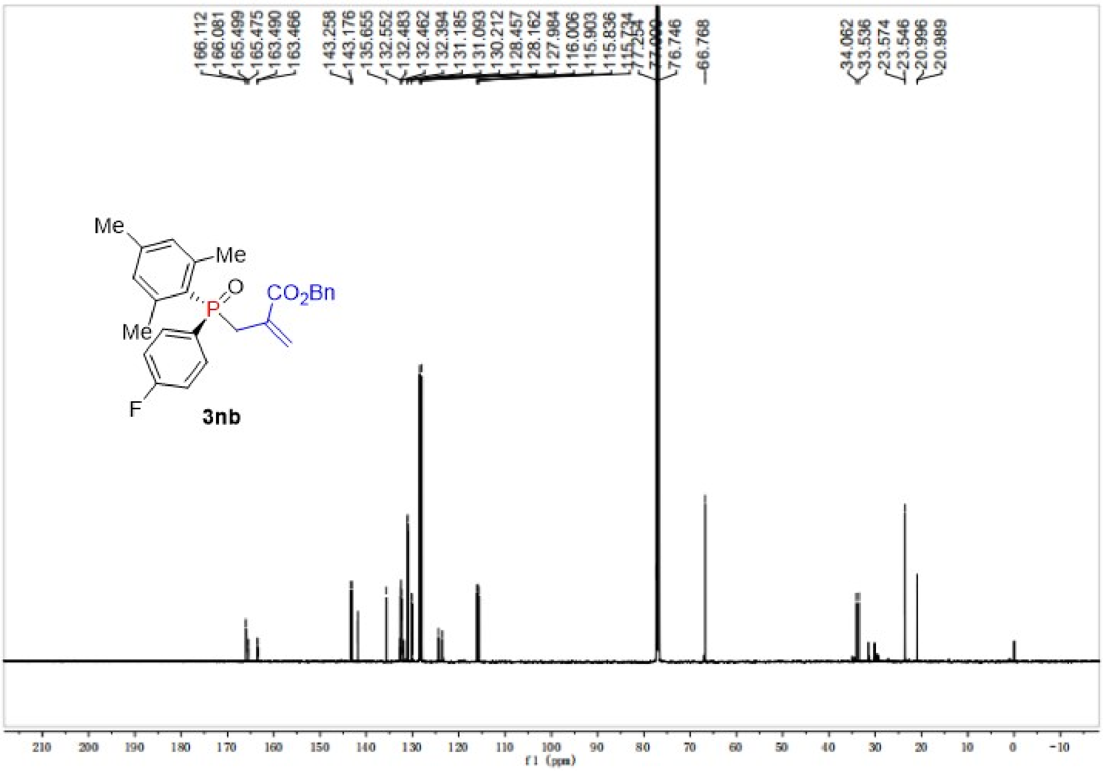

3nb
CO2Bn
f1 (ppm)

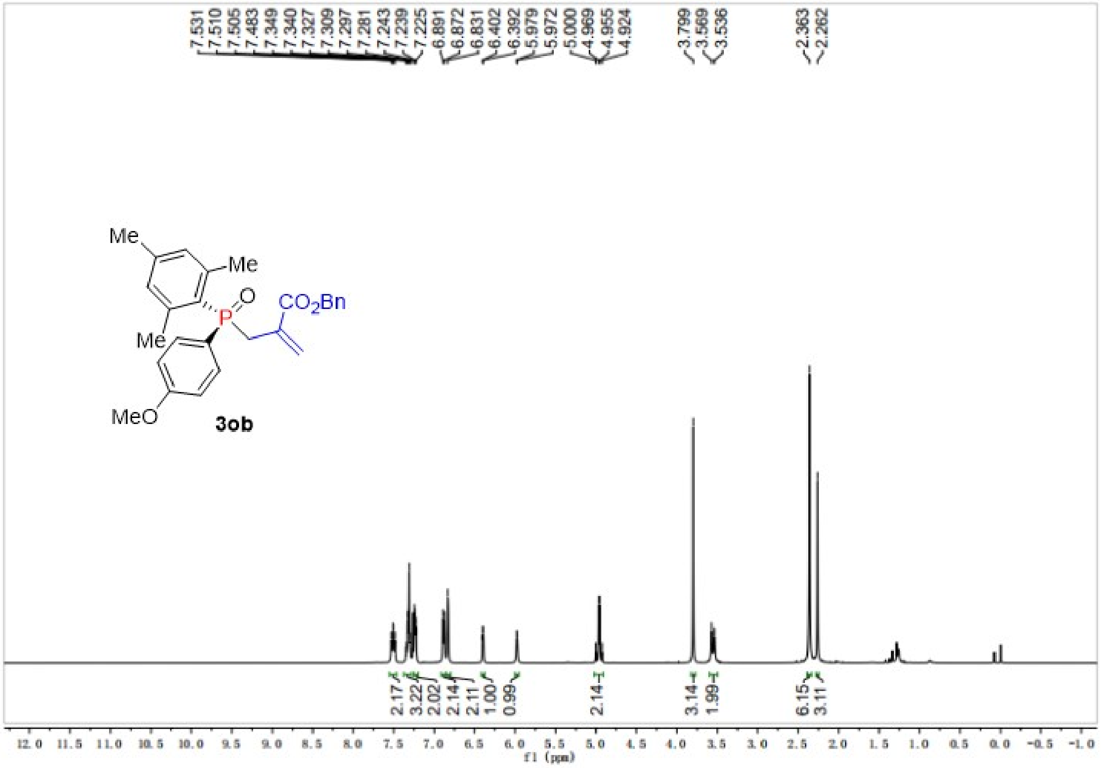

3ob
CO2Bn
MeO
f1 (ppm)

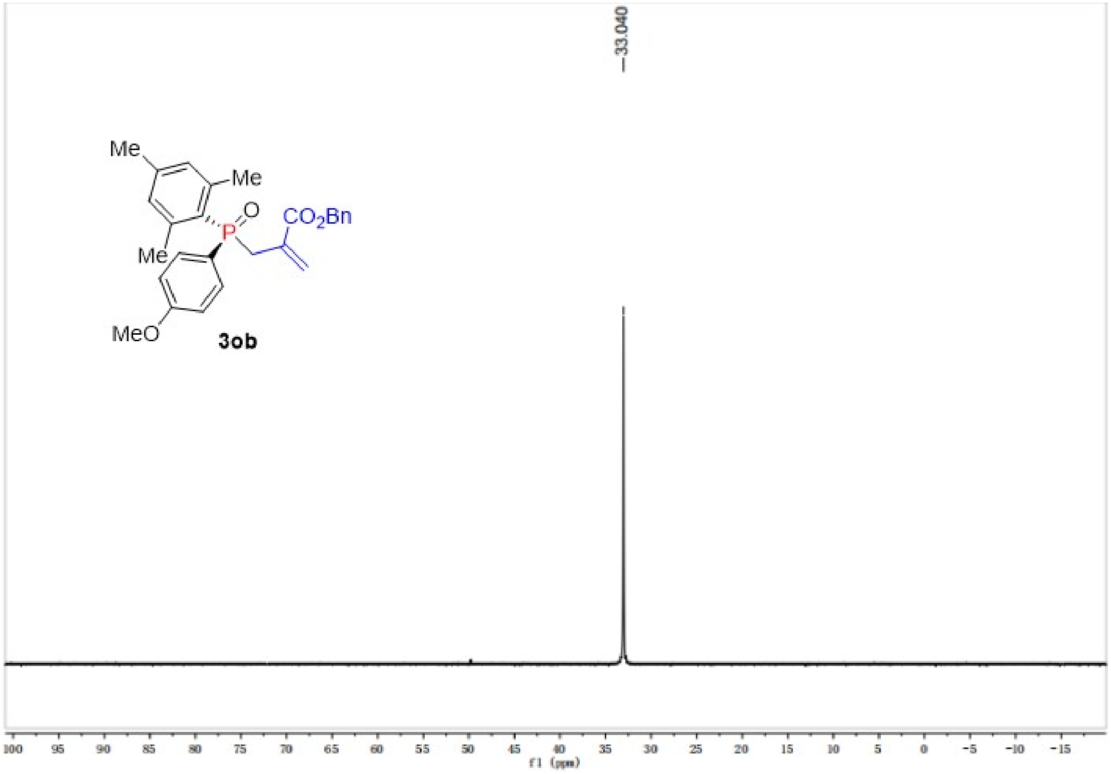

33.040
Me
Me
Me
O
P
CO2Bn
MeO
3ob
f1 (ppm)

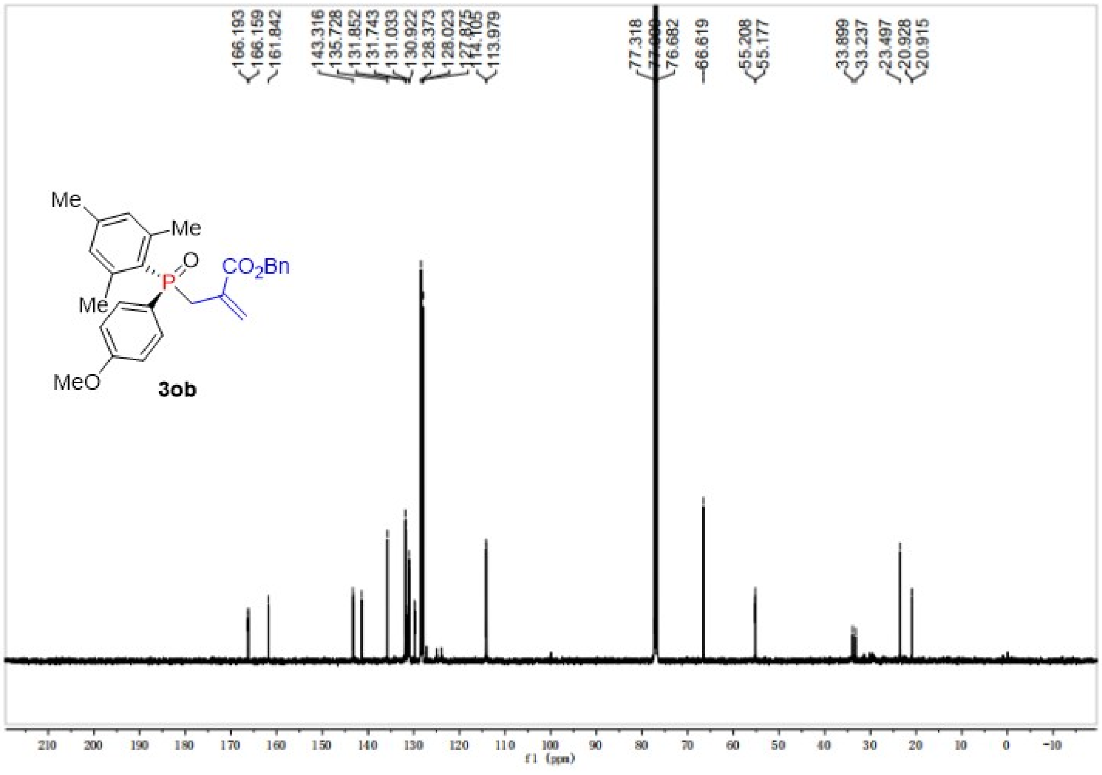

166.193
166.159
161.842
143.316
135.728
131.852
131.743
131.033
130.922
128.373
128.023
127.875
114.105
113.979
77.318
76.682
66.619
55.208
55.177
33.899
33.237
23.497
20.928
20.915
Me
Me
Me
O
P
CO2Bn
MeO
3ob
f1 (ppm)

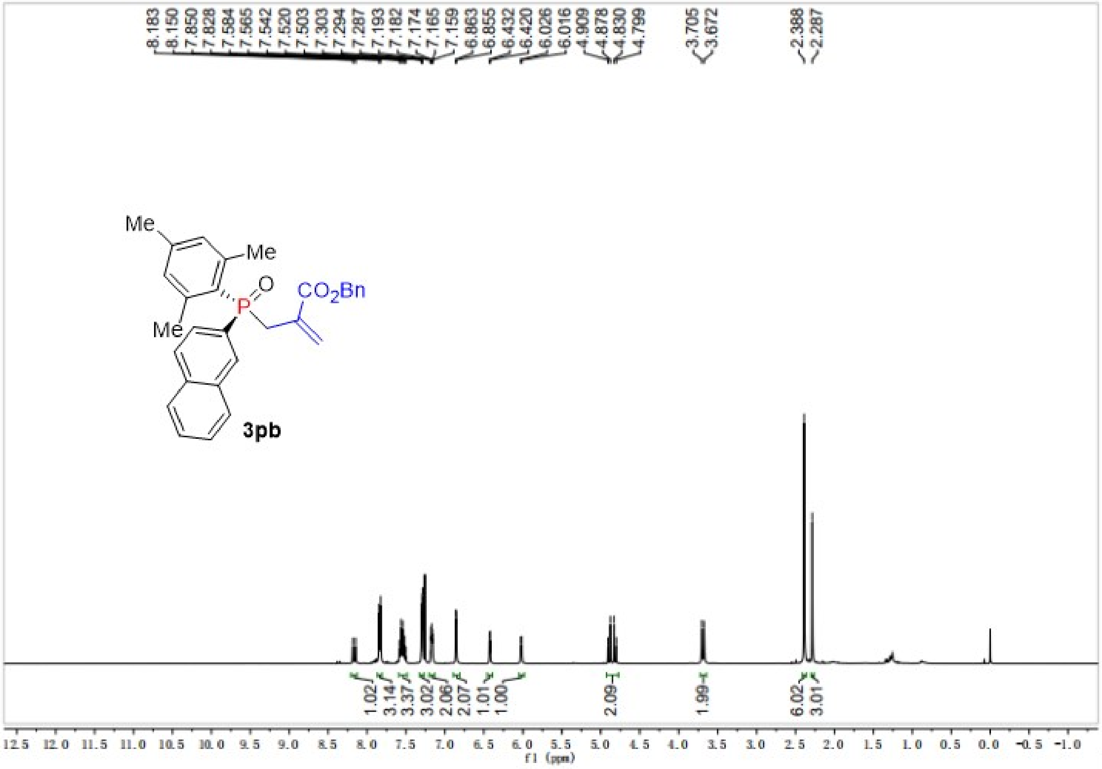
8.183
8.150
7.850
7.828
7.584
7.565
7.542
7.520
7.503
7.303
7.294
7.287
7.193
7.182
7.174
7.165
7.159
6.863
6.855
6.432
6.420
6.026
6.016
4.909
4.878
4.830
4.799
3.705
3.672
2.388
2.287
Me
Me
Me
O
P
CO2Bn
3pb
1.02
3.14
3.37
3.02
2.06
2.07
1.01
1.00
2.09
1.99
6.02
3.01
f1 (ppm)

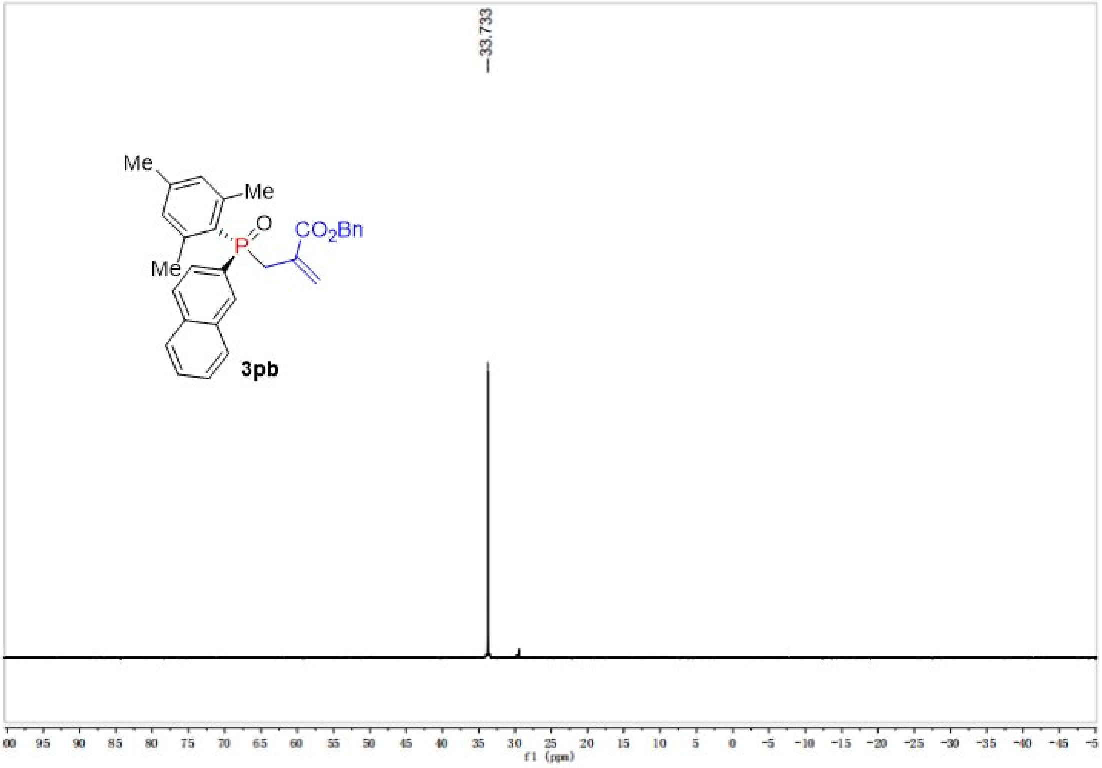
33.733
Me
Me
Me
O
P
CO2Bn
3pb
f1 (ppm)

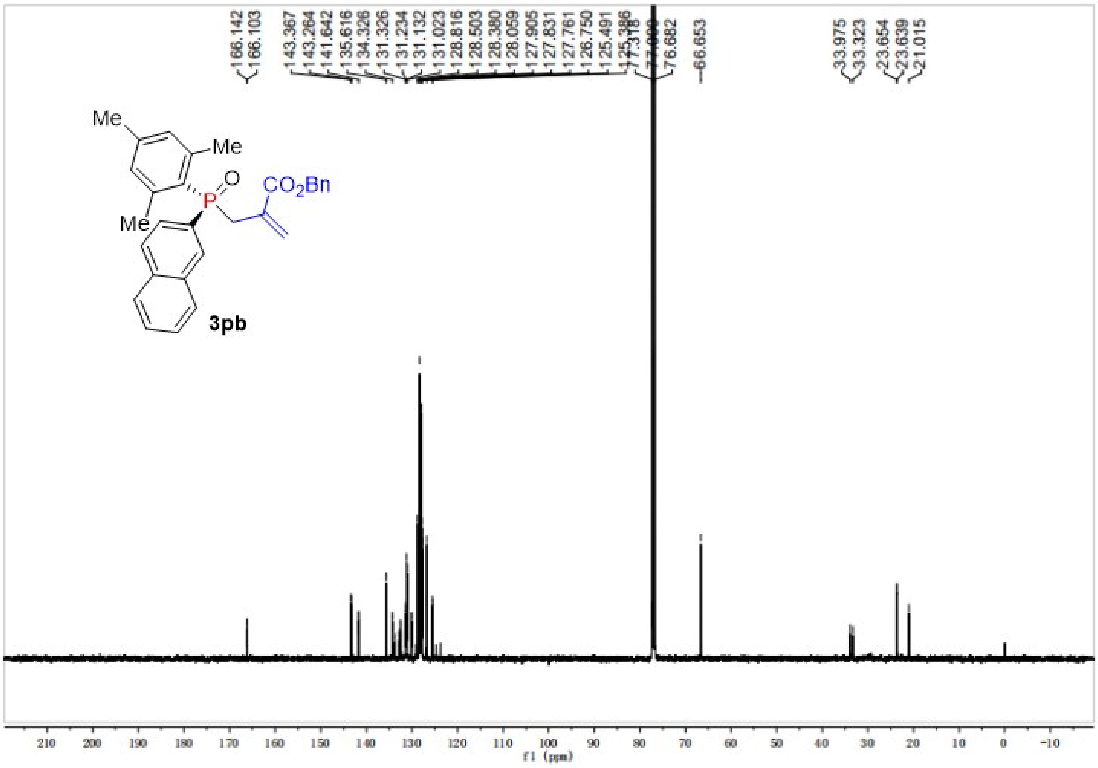
3pb
f1 (ppm)

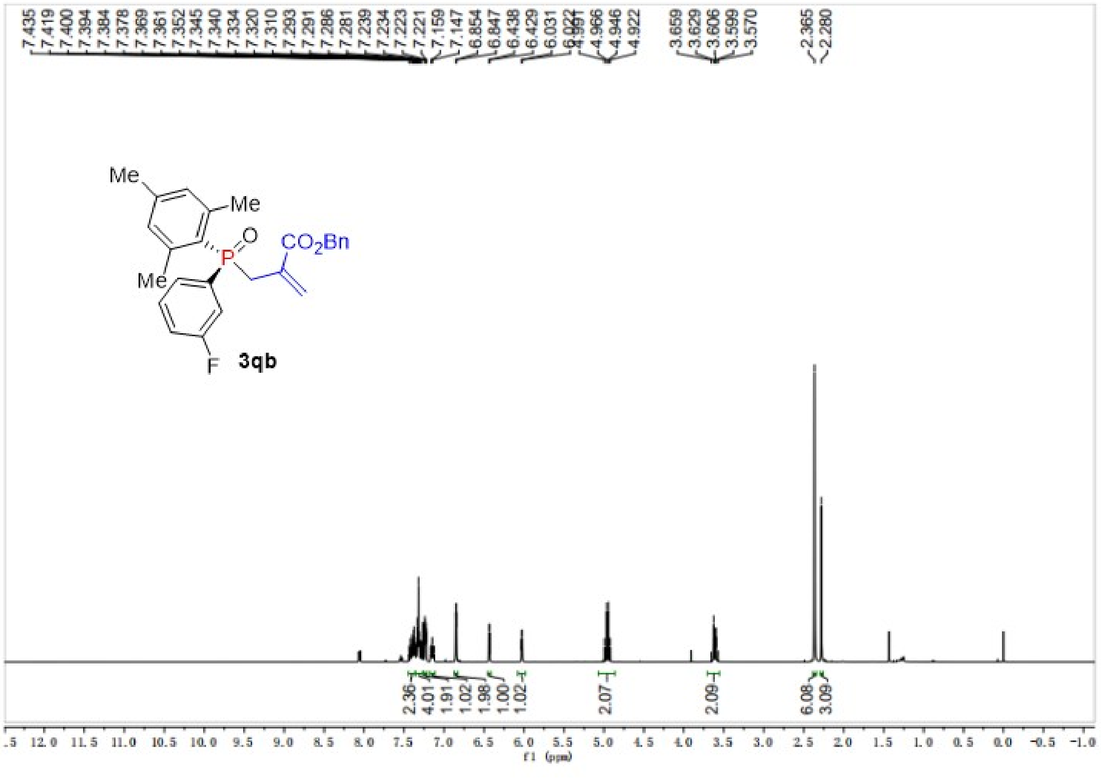
3qb
f1 (ppm)

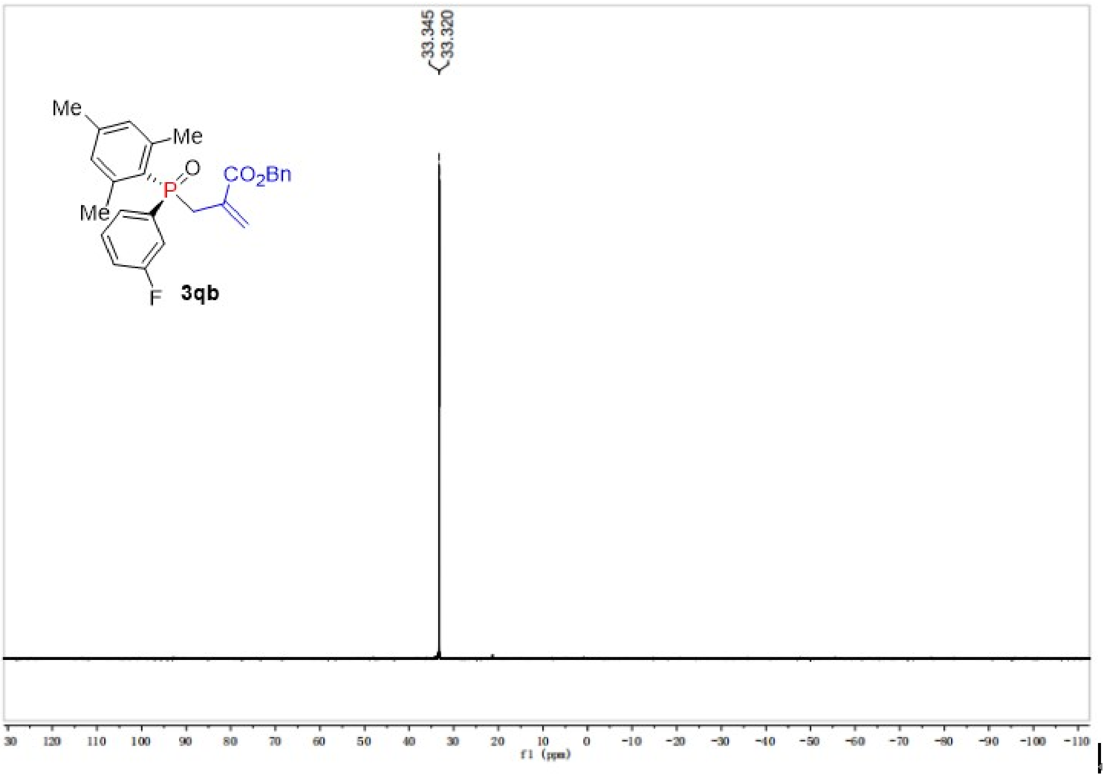
33.345
33.320
Me
Me
O
CO2Bn
P
Me
F
3qb
f1 (ppm)

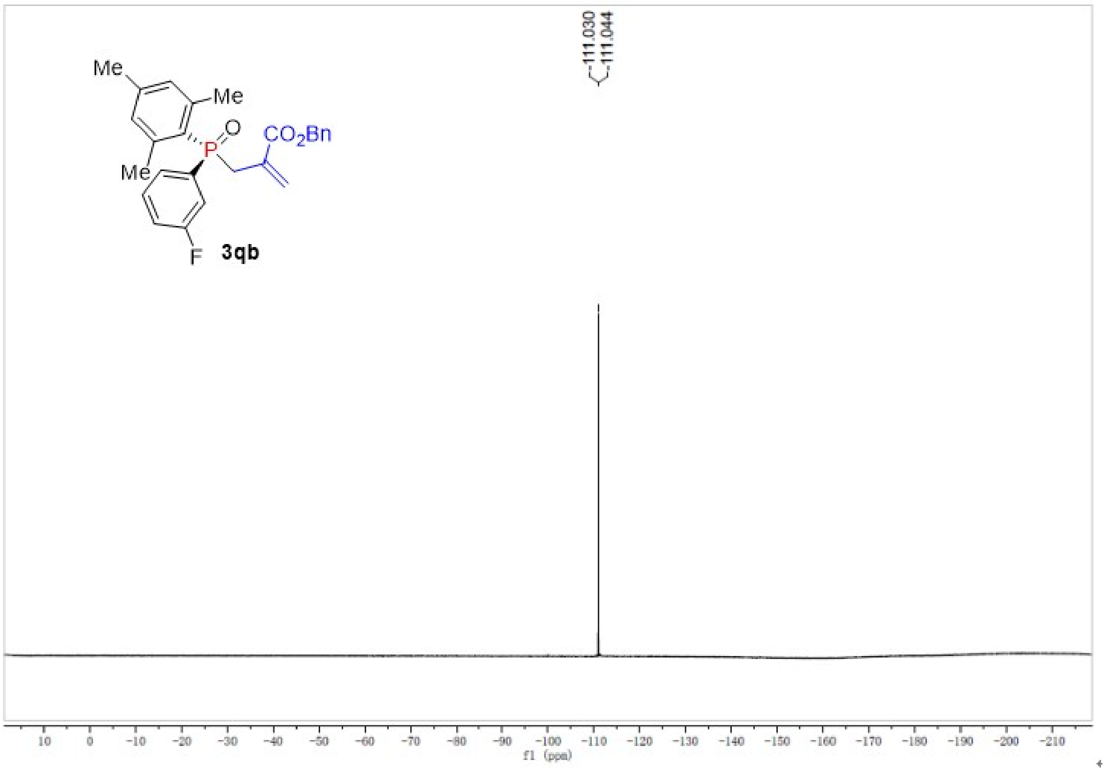
-111.030
-111.044
Me
Me
O
CO2Bn
P
Me
F
3qb
f1 (ppm)

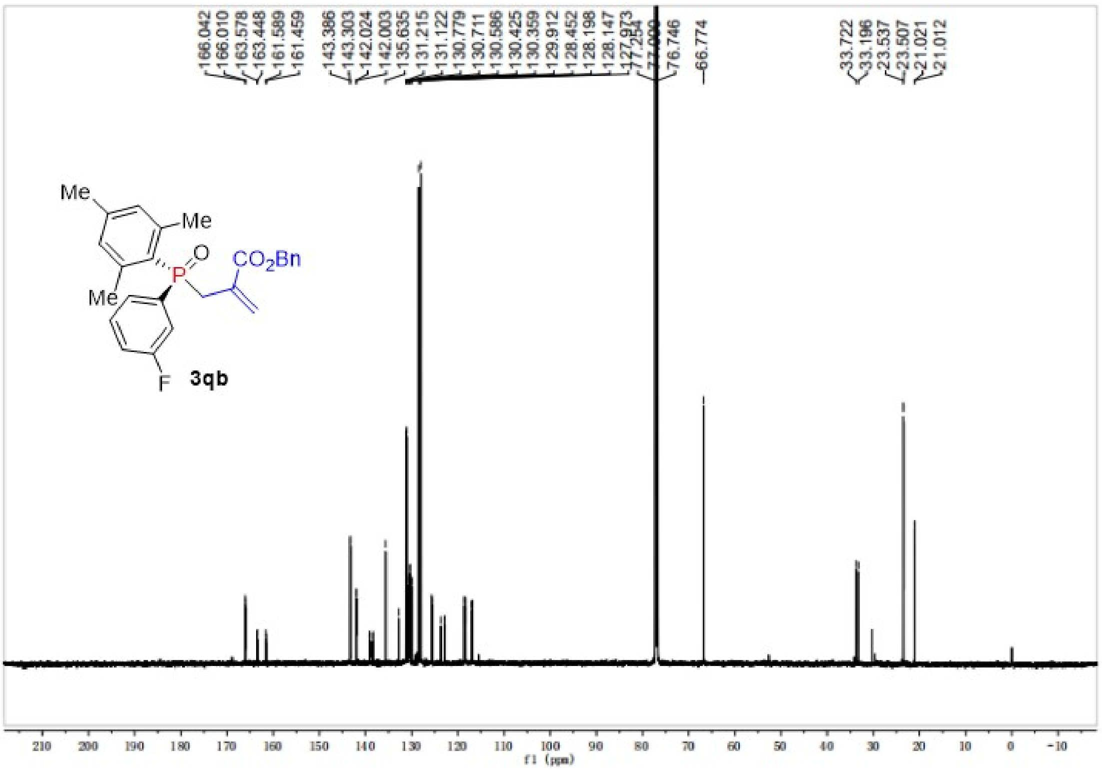

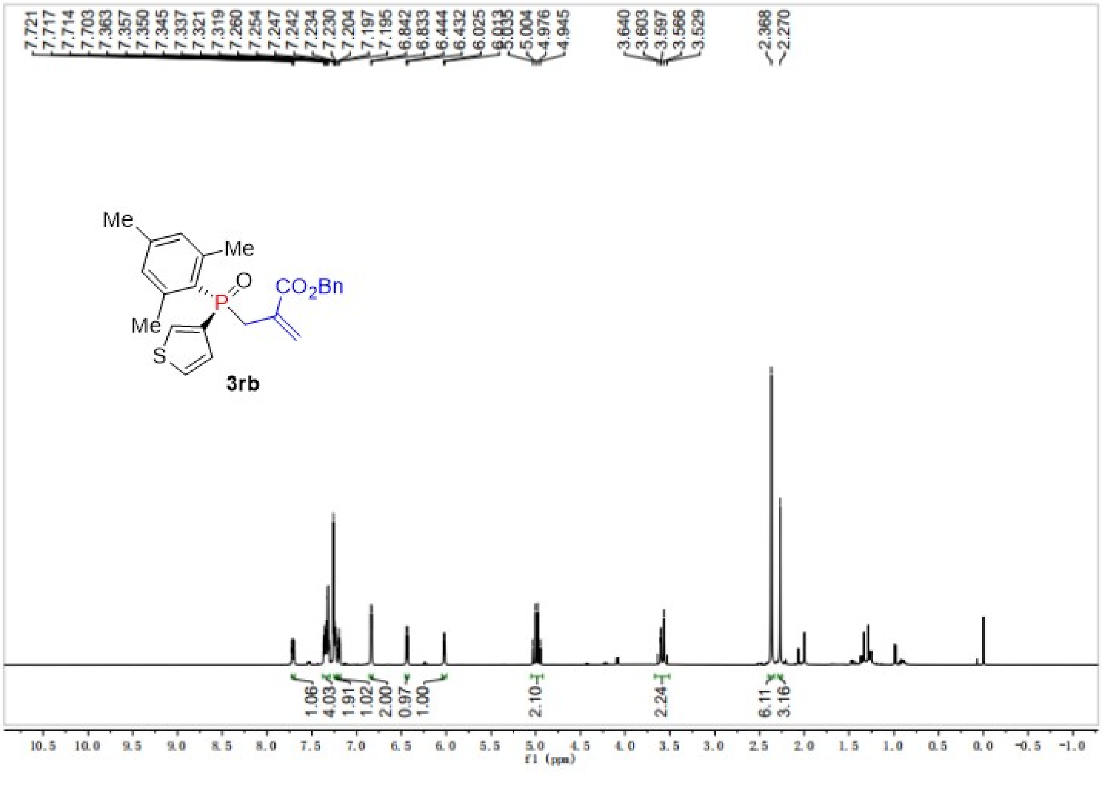

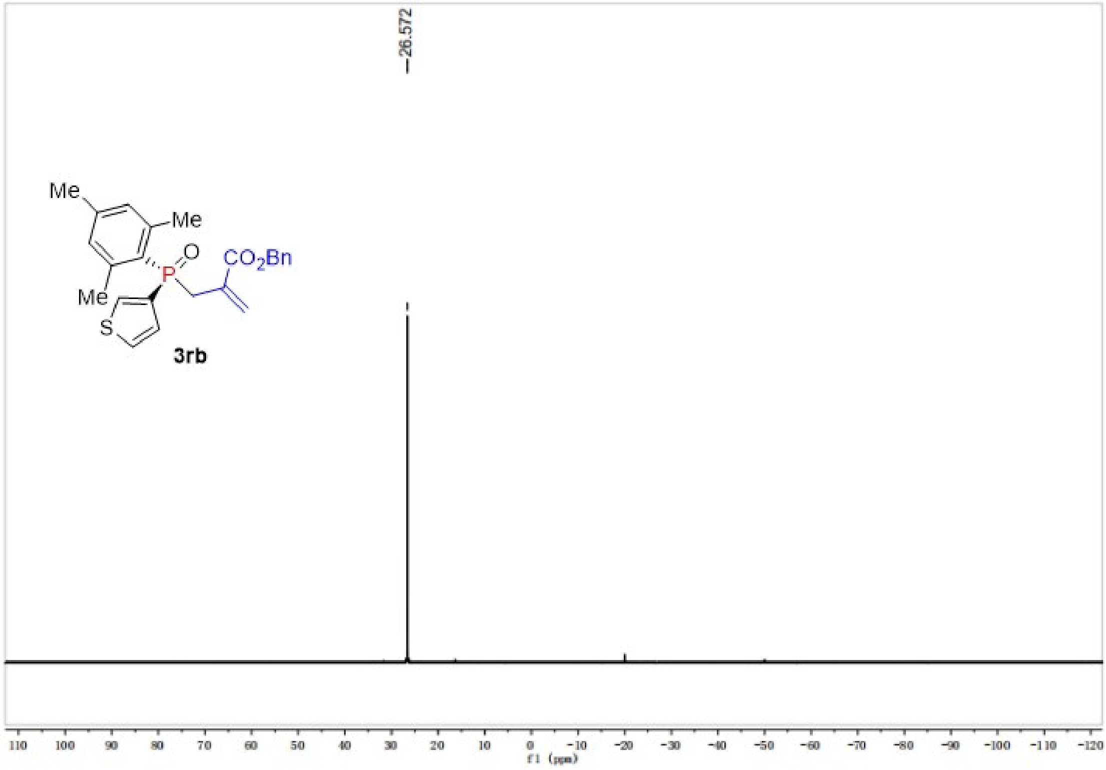

26.572
Me
Me
O
P
CO2Bn
Me
S
3rb
f1 (ppm)

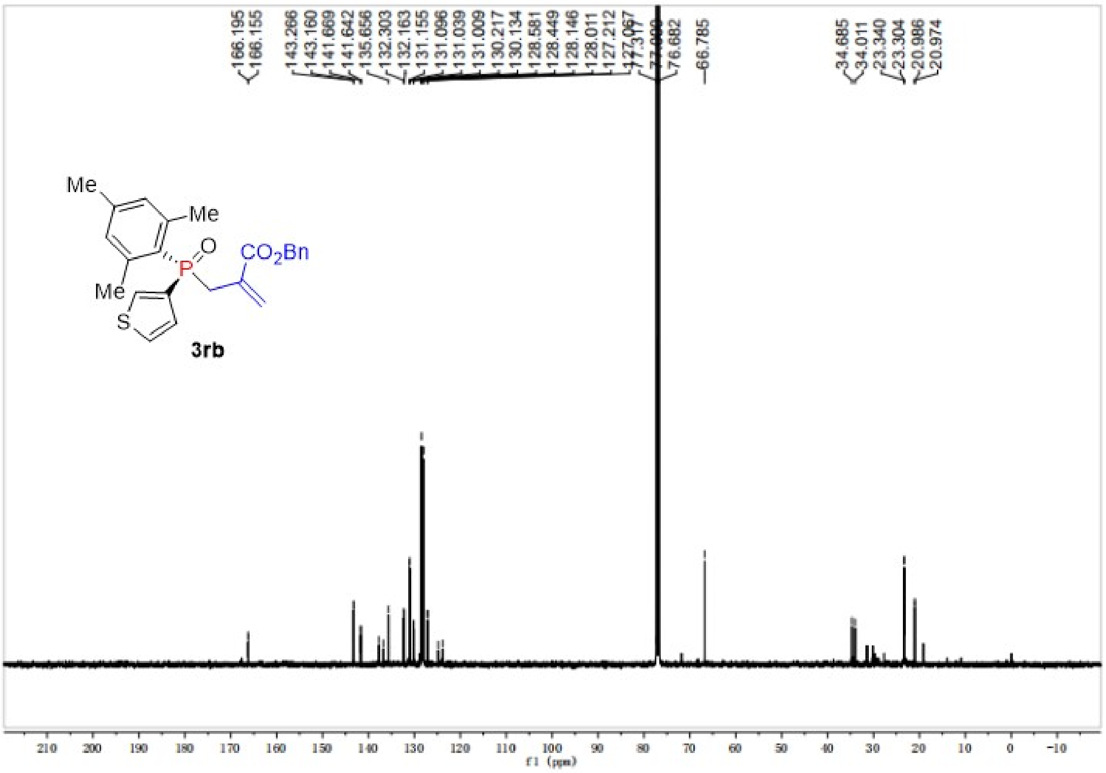

166.195
166.155
143.266
143.160
141.669
141.642
135.656
132.303
132.163
131.155
131.096
131.039
131.009
130.217
130.134
128.581
128.449
128.146
128.011
127.212
127.067
77.317
76.682
66.785
34.685
34.011
23.340
23.304
20.986
20.974
Me
Me
O
P
CO2Bn
Me
S
3rb
f1 (ppm)

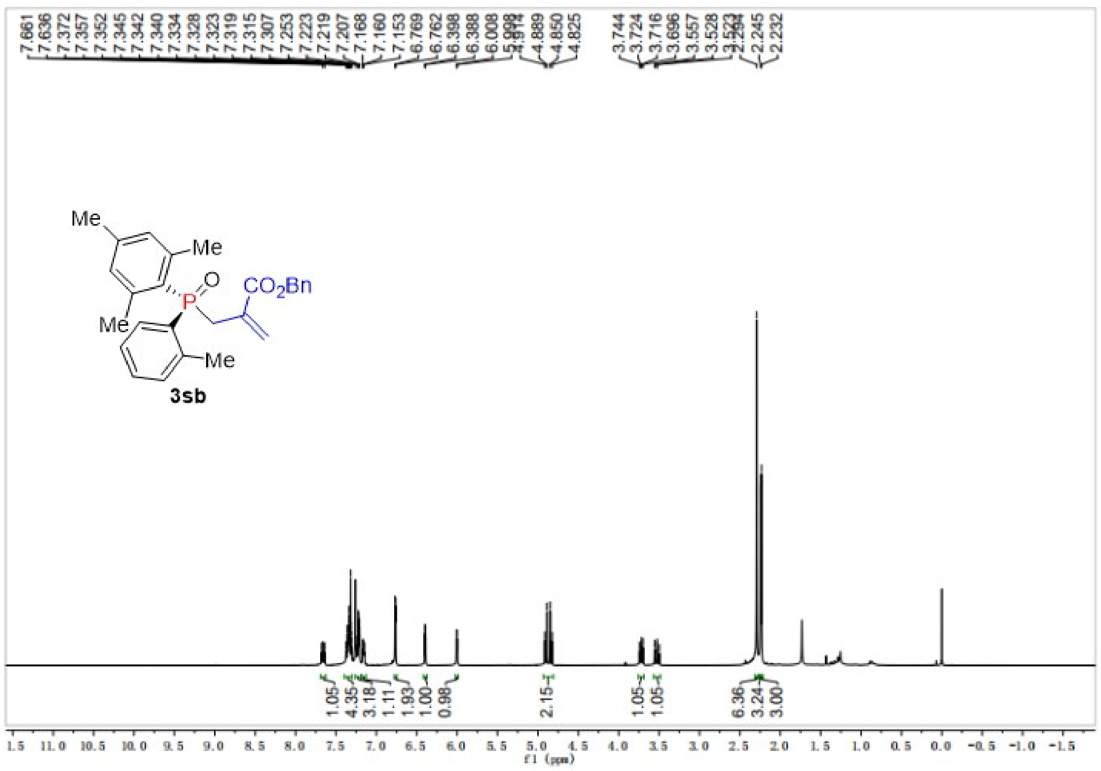

Me
Me
O
CO2Bn
P
Me
Me
3sb
f1 (ppm)

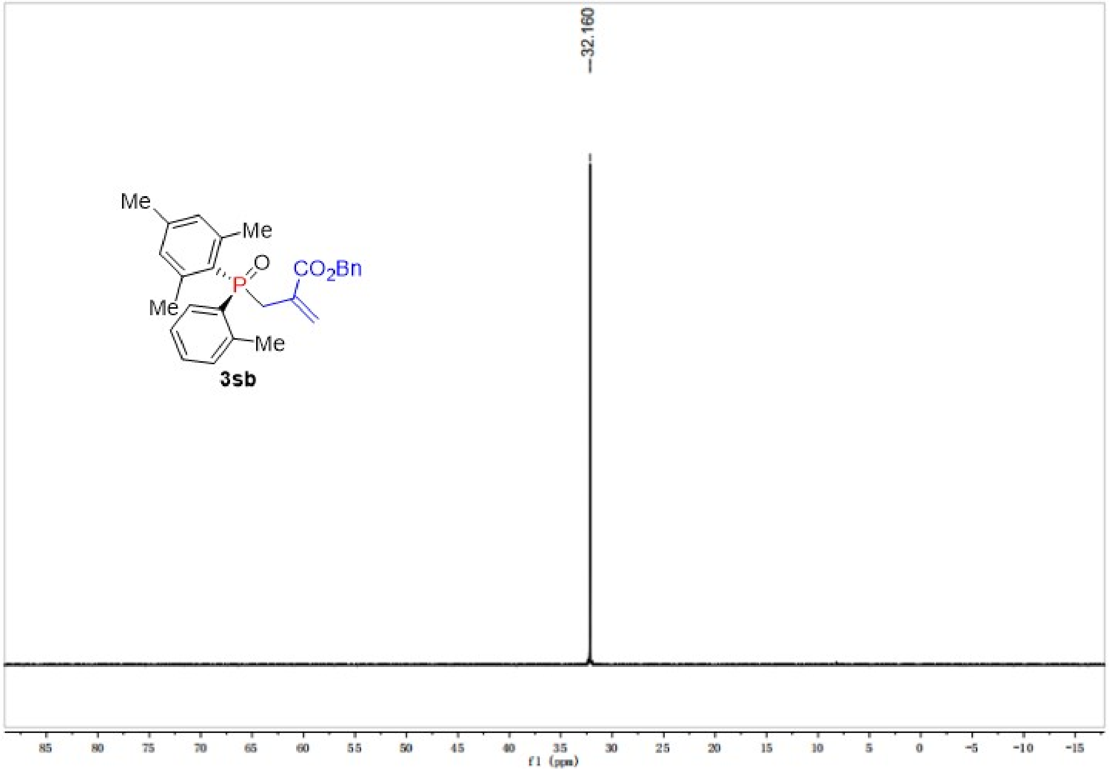

32.160
Me
Me
O
CO2Bn
P
Me
Me
3sb
f1 (ppm)

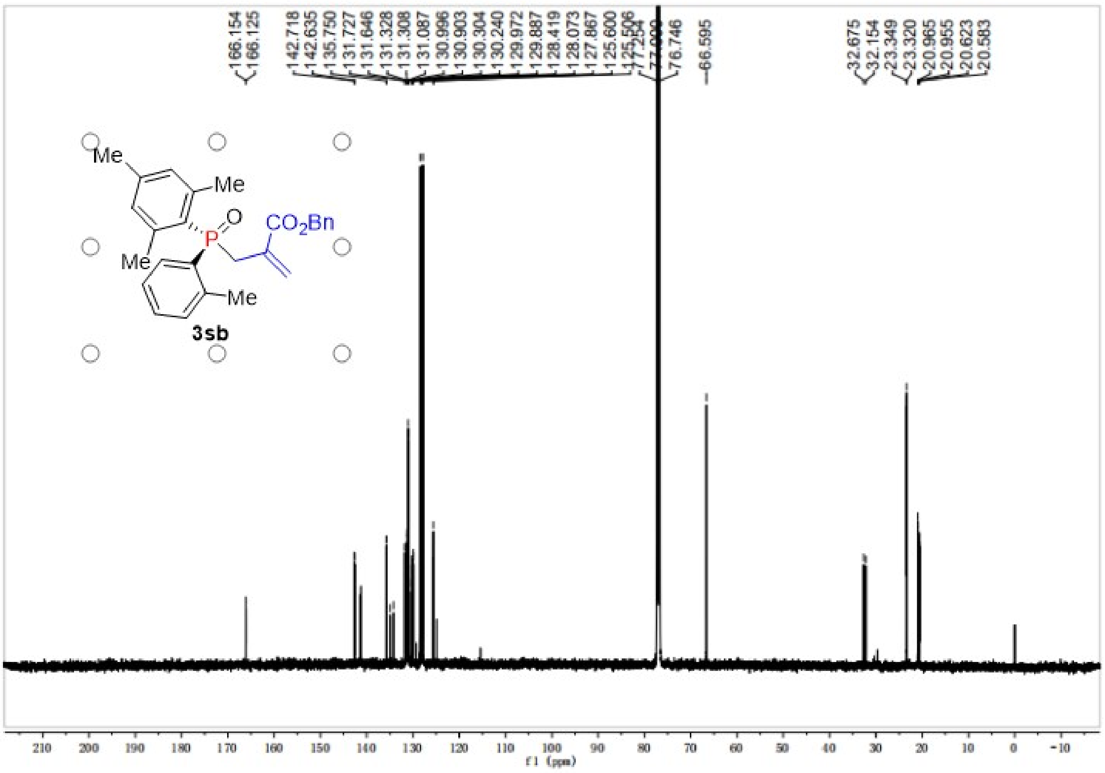
166.154
166.125
142.718
142.635
135.750
131.727
131.646
131.328
131.308
131.087
130.996
130.903
130.304
130.240
129.972
129.887
128.419
128.073
127.867
125.600
125.506
77.254
77.000
76.746
66.595
32.675
32.154
23.349
23.320
20.965
20.955
20.623
20.583
Me
Me
Me
Me
P
O
CO_2Bn
3sb
f1 (ppm)

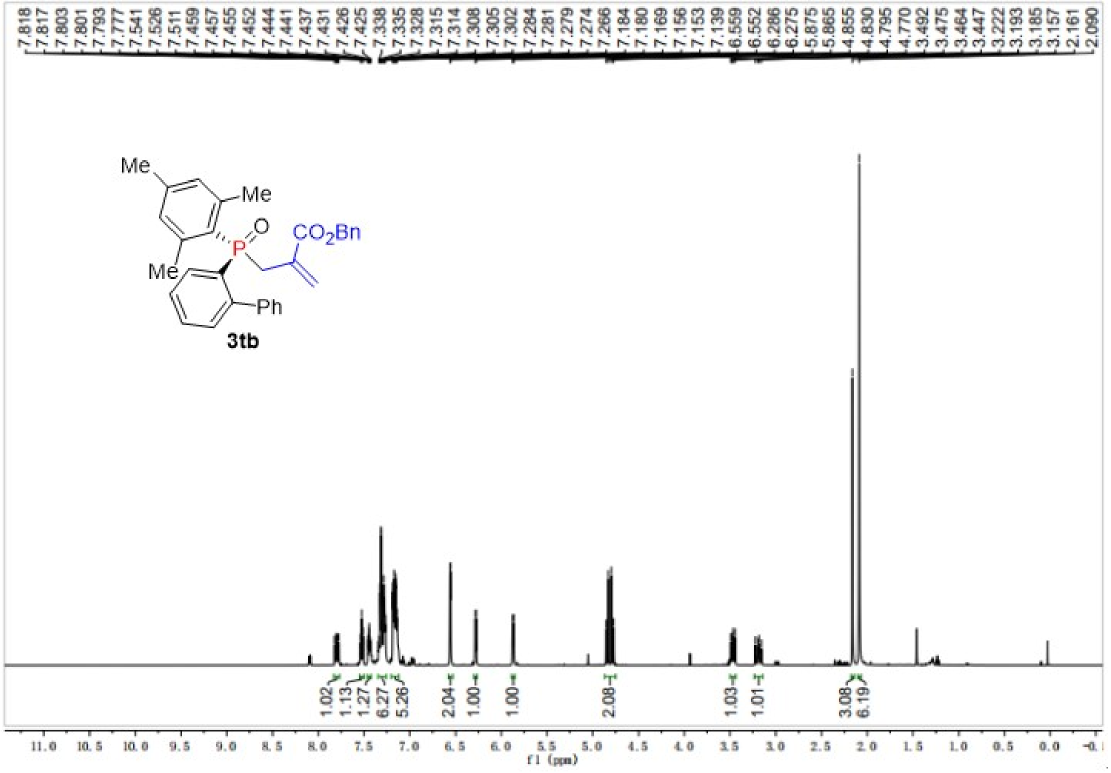
7.818
7.817
7.803
7.801
7.793
7.777
7.541
7.526
7.511
7.459
7.457
7.455
7.452
7.444
7.441
7.437
7.431
7.426
7.425
7.338
7.335
7.328
7.315
7.314
7.308
7.305
7.302
7.284
7.281
7.279
7.274
7.266
7.184
7.180
7.169
7.156
7.153
7.139
6.559
6.552
6.286
6.275
5.875
5.865
4.855
4.830
4.795
4.770
3.492
3.475
3.464
3.447
3.222
3.193
3.185
3.157
2.161
2.090
Me
Me
Me
P
O
CO_2Bn
Ph
3tb
1.02
1.13
1.27
6.27
5.26
2.04
1.00
1.00
2.08
1.03
1.01
3.08
6.19
f1 (ppm)

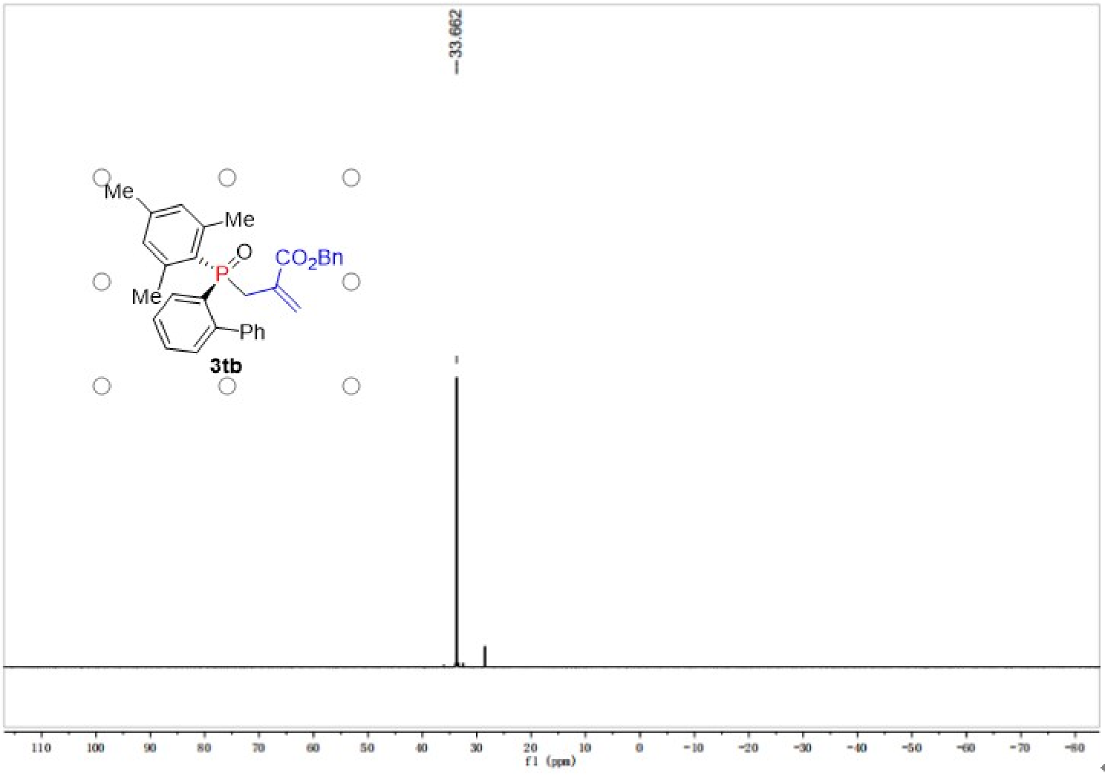

33.662
Me
Me
Me
O
P
CO2Bn
Ph
3tb
f1 (ppm)

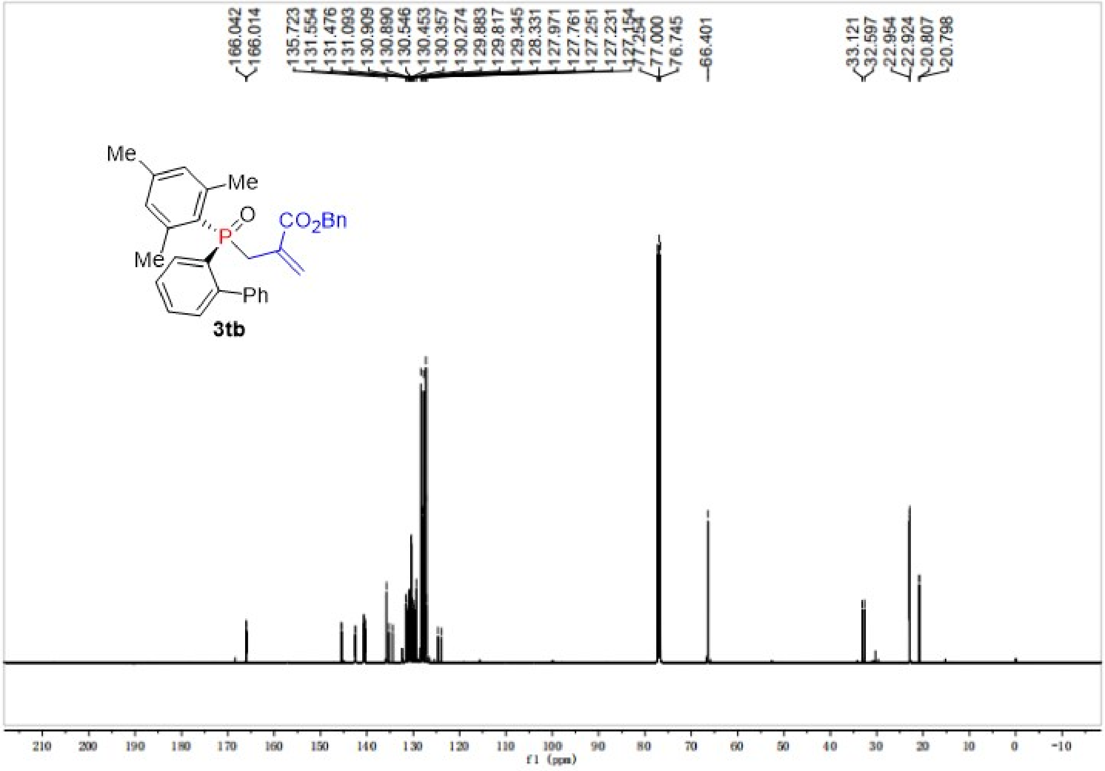

166.042
166.014
135.723
131.554
131.476
131.093
130.909
130.890
130.546
130.453
130.357
130.274
129.883
129.817
129.345
128.331
127.971
127.761
127.251
127.231
127.154
77.254
77.000
76.745
66.401
33.121
32.597
22.954
22.924
20.807
20.798
Me
Me
Me
O
P
CO2Bn
Ph
3tb
f1 (ppm)

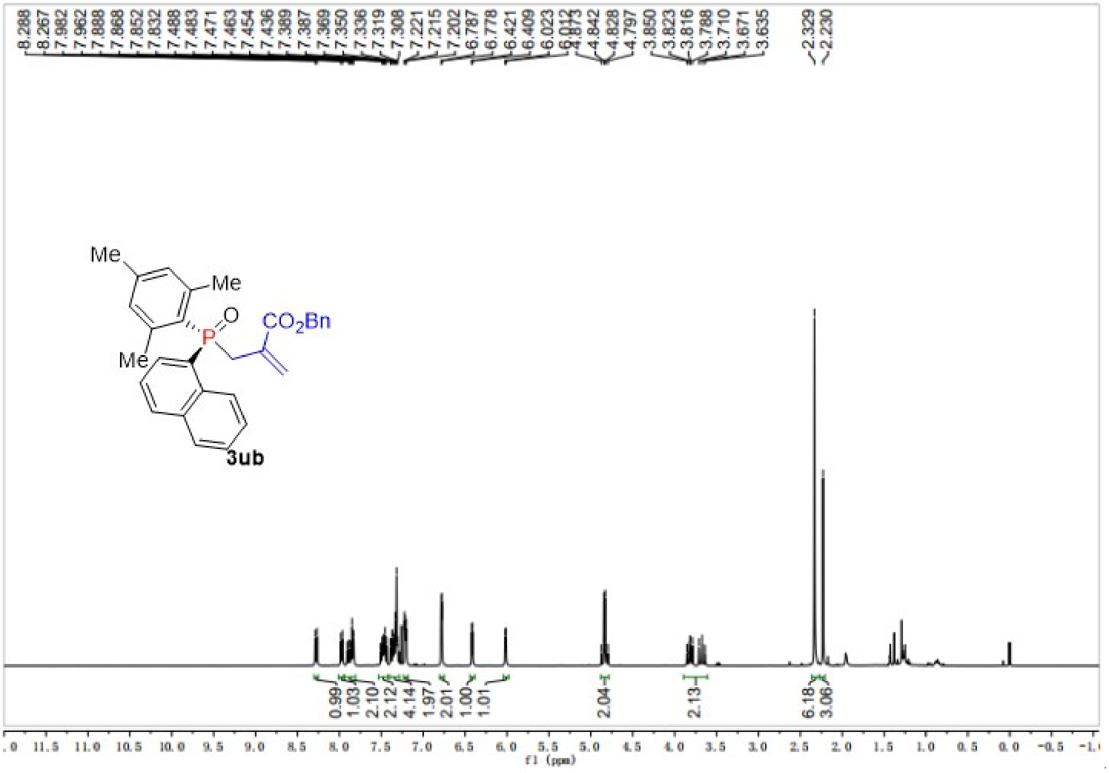
8.288
8.267
7.982
7.962
7.888
7.868
7.852
7.832
7.488
7.483
7.471
7.463
7.454
7.436
7.389
7.387
7.369
7.350
7.336
7.319
7.308
7.221
7.215
7.202
6.787
6.778
6.421
6.409
6.023
6.012
4.873
4.842
4.828
4.797
3.850
3.823
3.816
3.788
3.710
3.671
3.635
2.329
2.230
Me
Me
Me
O
P
CO2Bn
3ub
0.99
1.03
2.10
2.12
4.14
1.97
2.01
1.00
1.01
2.04
2.13
6.18
3.06
f1 (ppm)

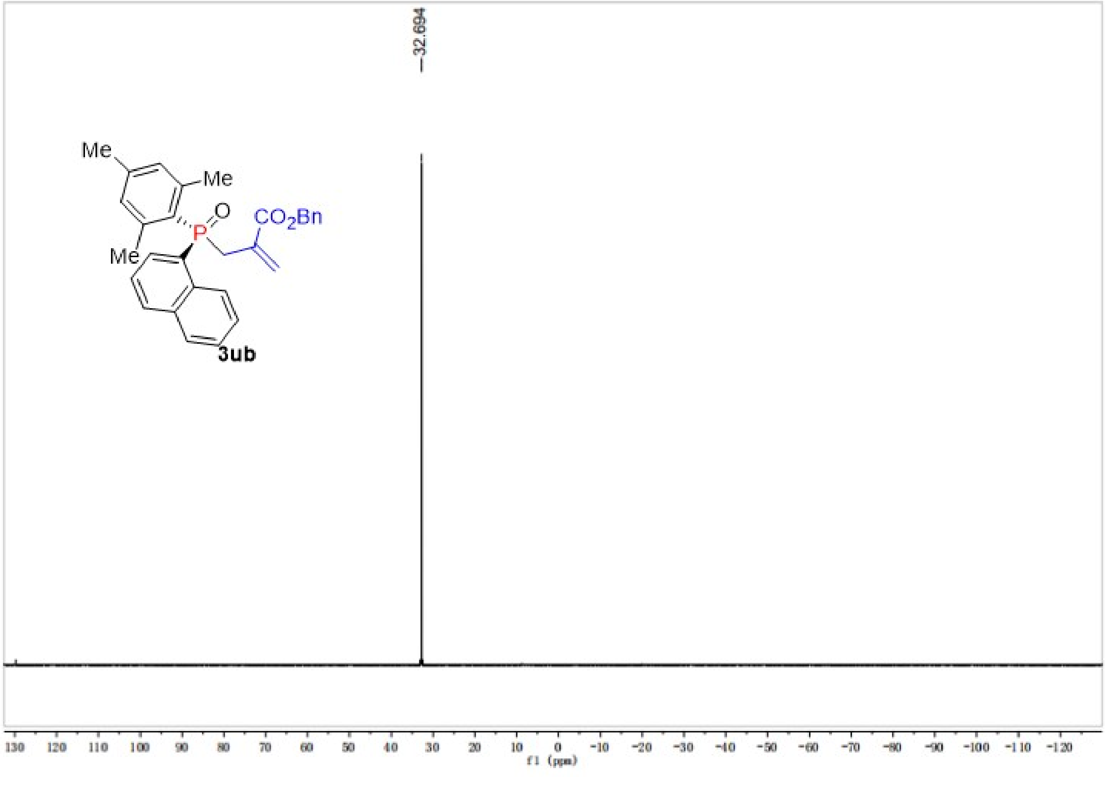
32.694
Me
Me
Me
O
P
CO2Bn
3ub
f1 (ppm)

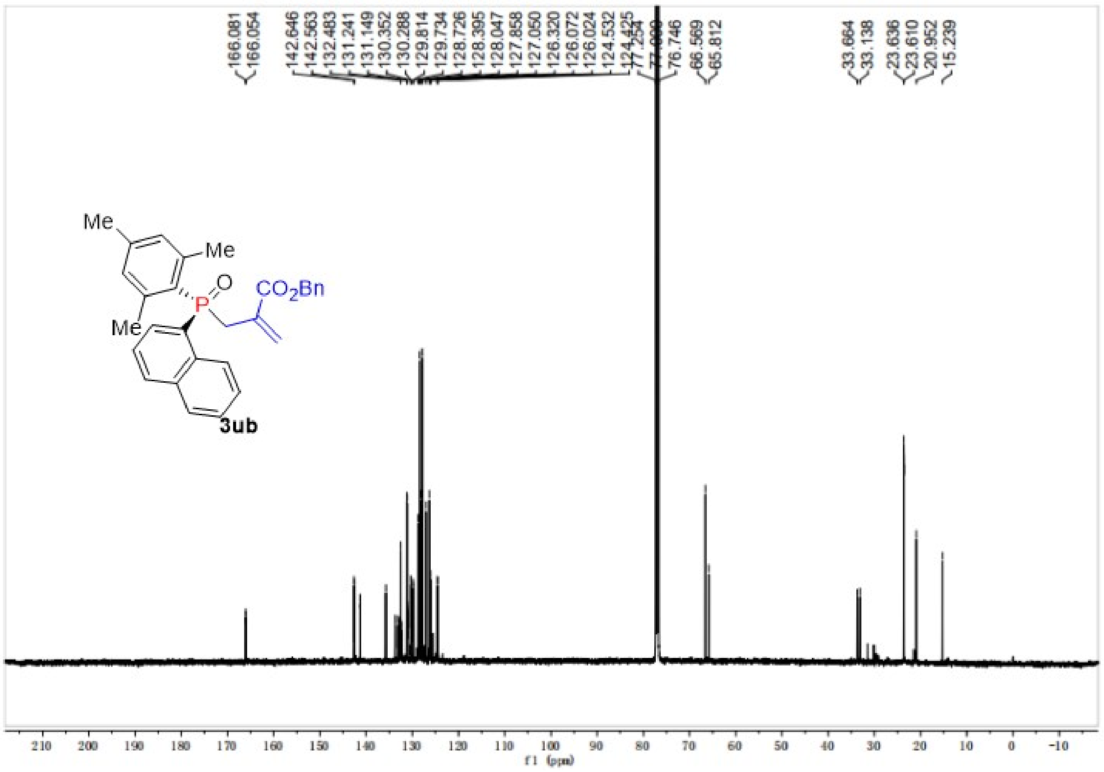

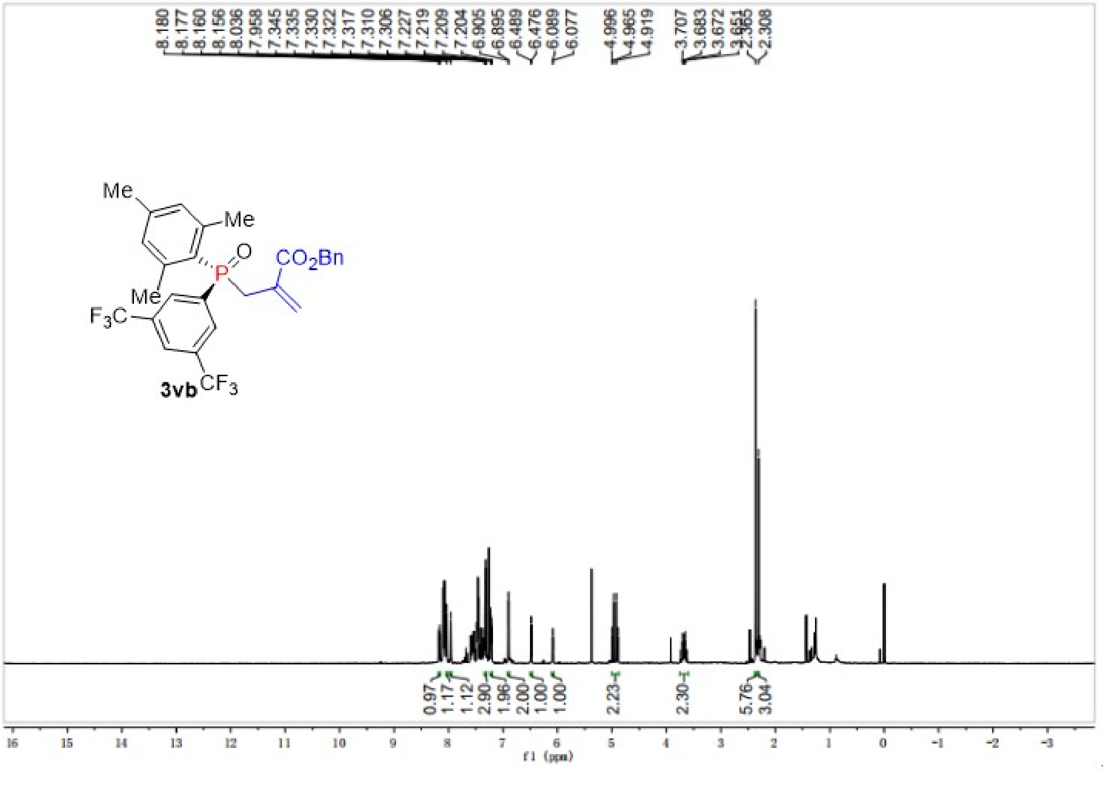

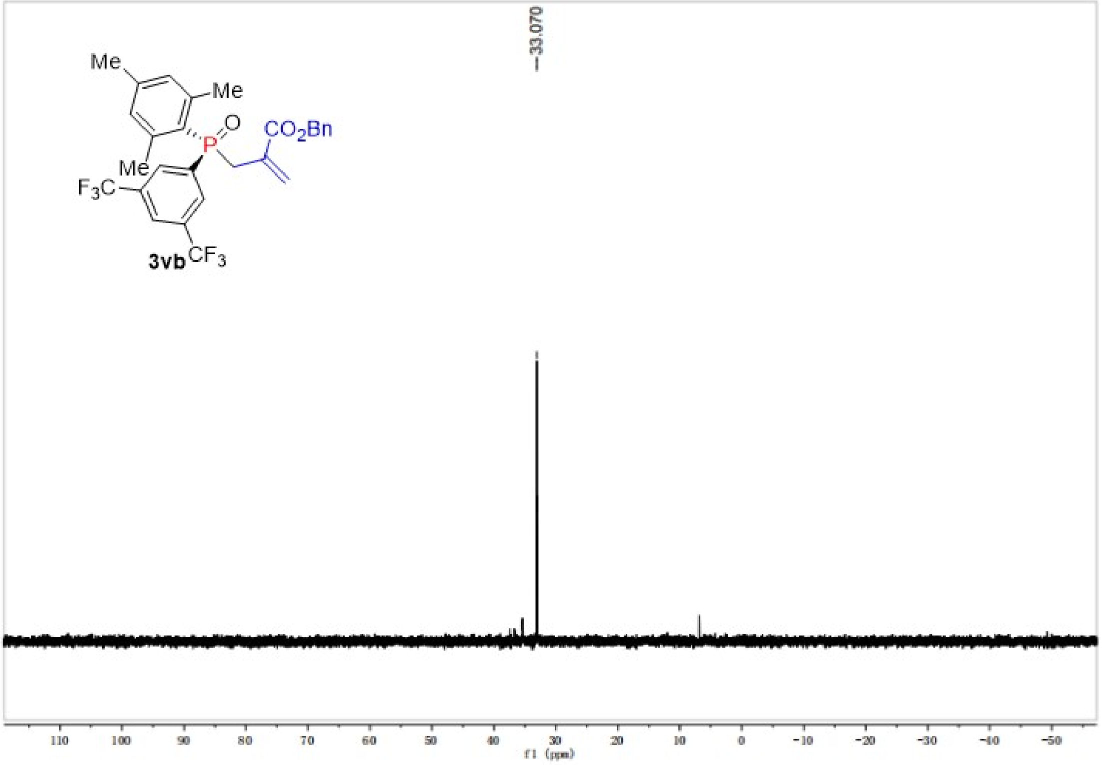
33.070
Me
Me
O
P
CO2Bn
Me
F3C
3vb
CF3
110 100 90 80 70 60 50 40 30 20 10 0 −10 −20 −30 −40 −50
f1 (ppm)

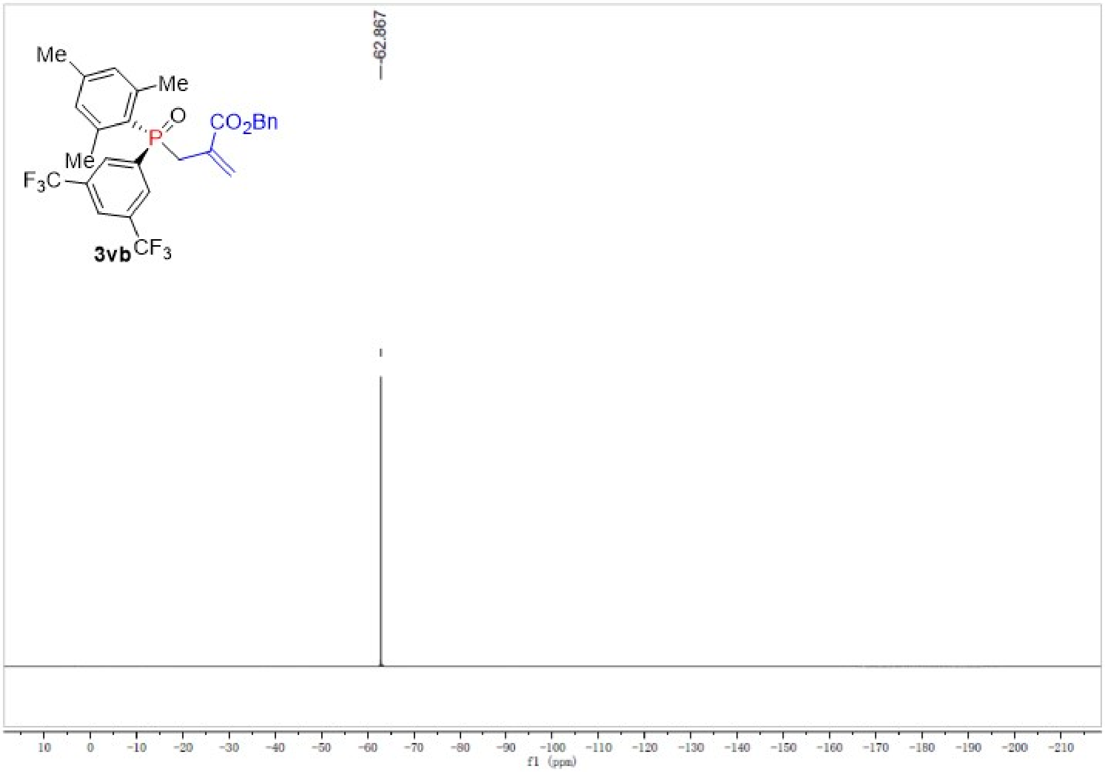
-62.867
Me
Me
O
P
CO2Bn
Me
F3C
3vb
CF3
10 0 −10 −20 −30 −40 −50 −60 −70 −80 −90 −100 −110 −120 −130 −140 −150 −160 −170 −180 −190 −200 −210
f1 (ppm)

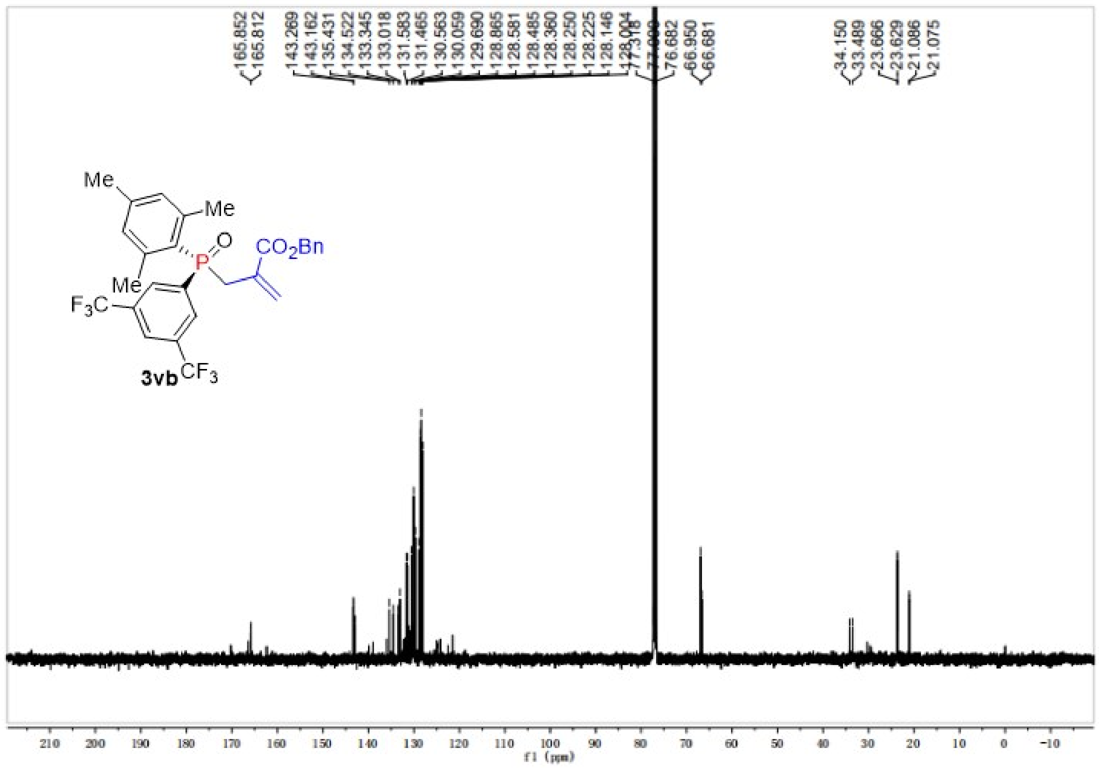
3vb
f1 (ppm)

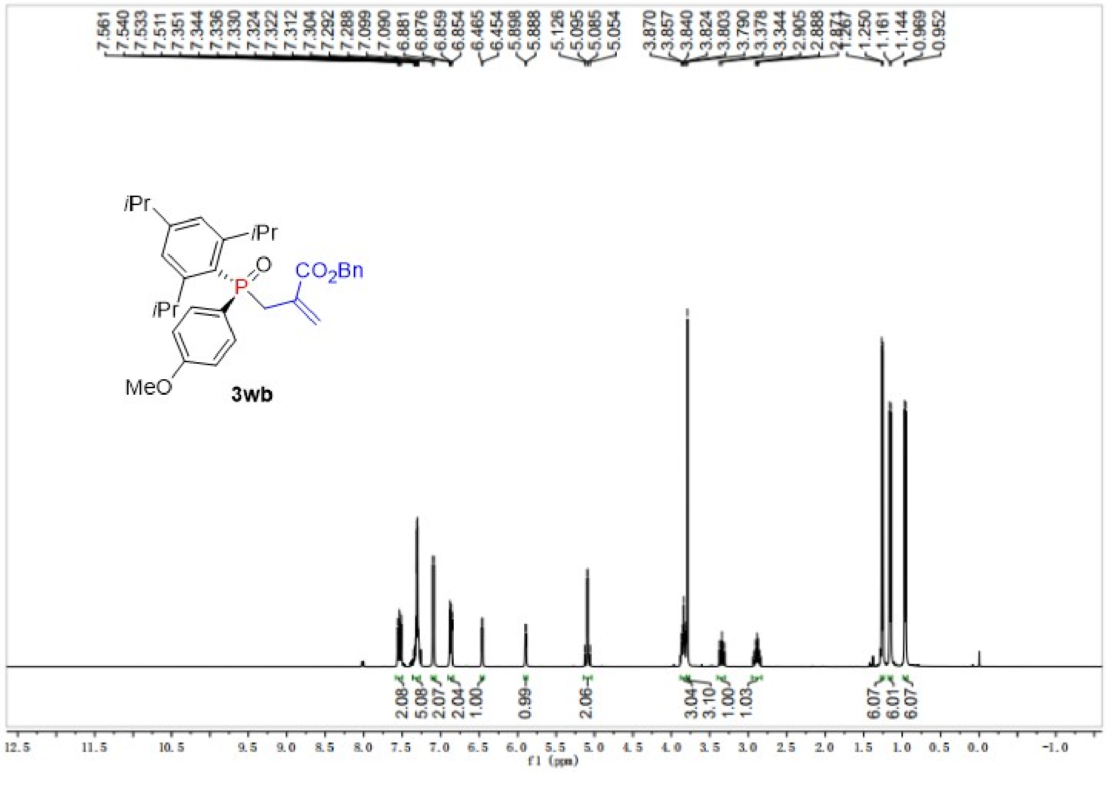
3wb
f1 (ppm)

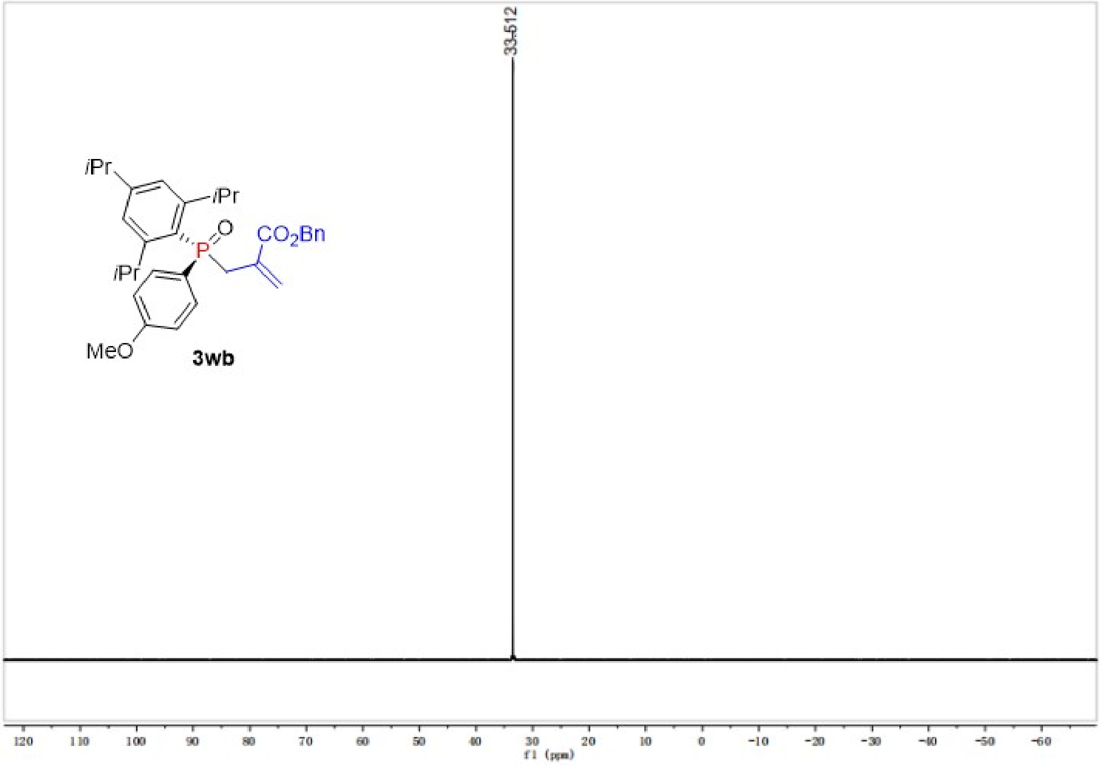

33.512
iPr
iPr
iPr
O
P
CO2Bn
MeO
3wb
f1 (ppm)

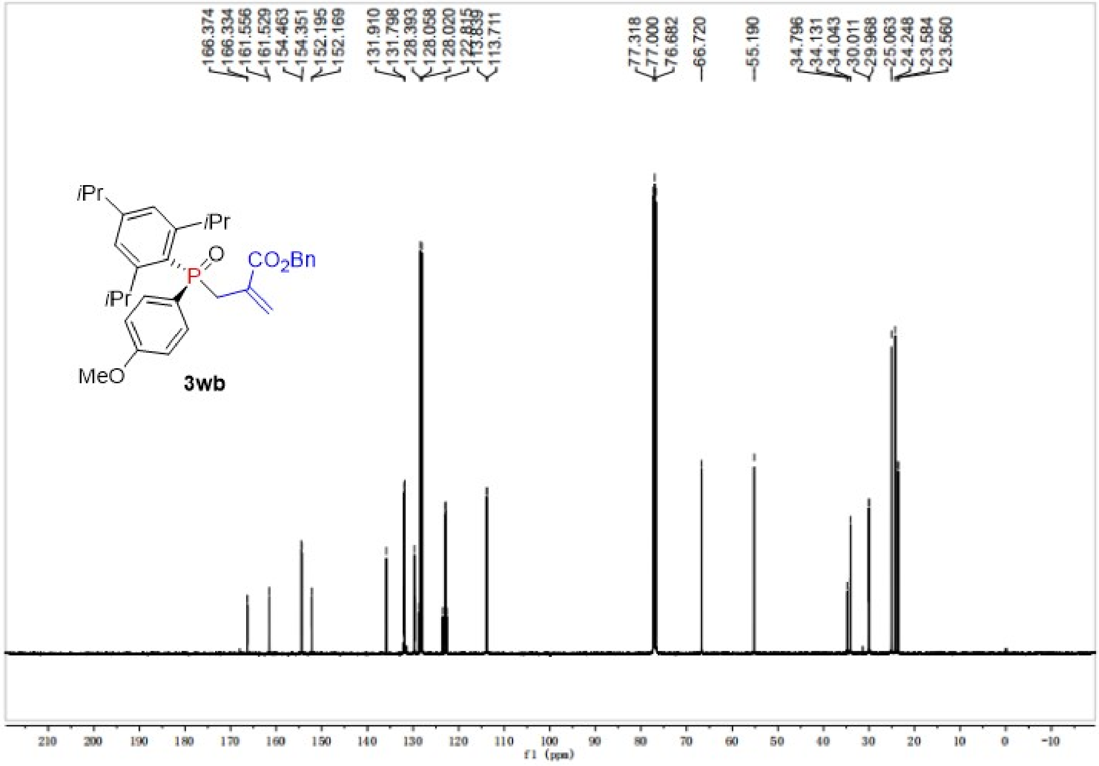

166.374
166.334
161.556
161.529
154.463
154.351
152.195
152.169
131.910
131.798
128.393
128.058
128.020
122.815
113.839
113.711
77.318
77.000
76.682
66.720
55.190
34.796
34.131
34.043
30.011
29.968
25.063
24.248
23.584
23.560
iPr
iPr
iPr
O
P
CO2Bn
MeO
3wb
f1 (ppm)

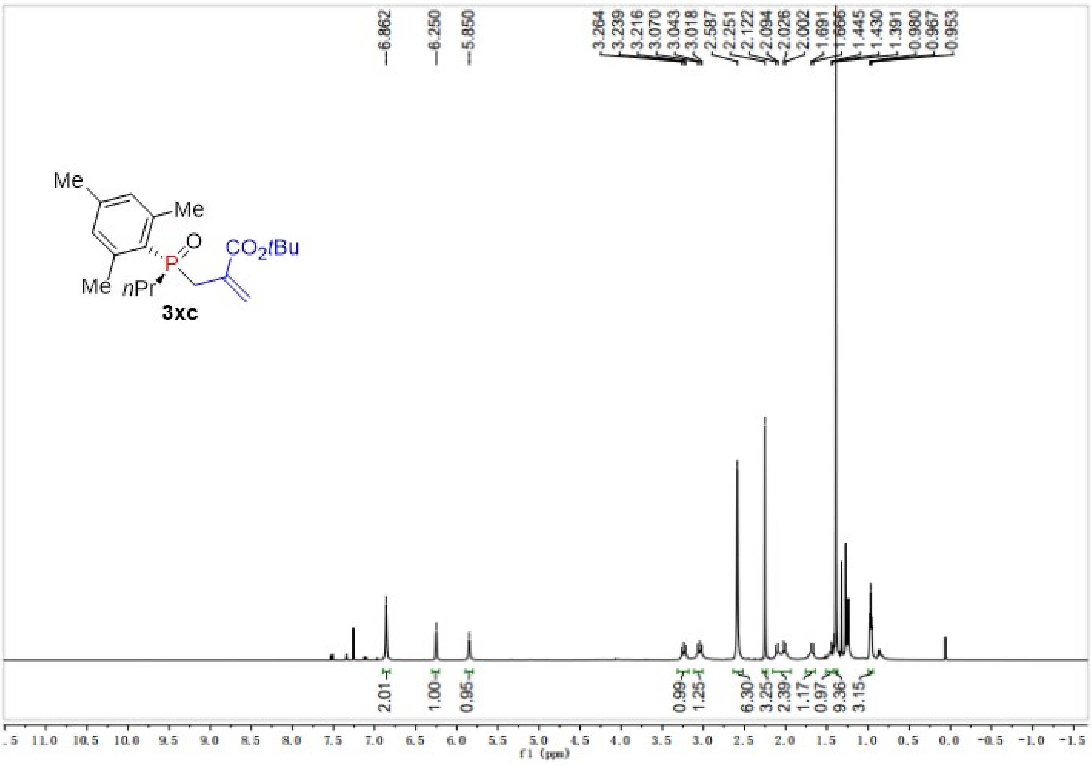

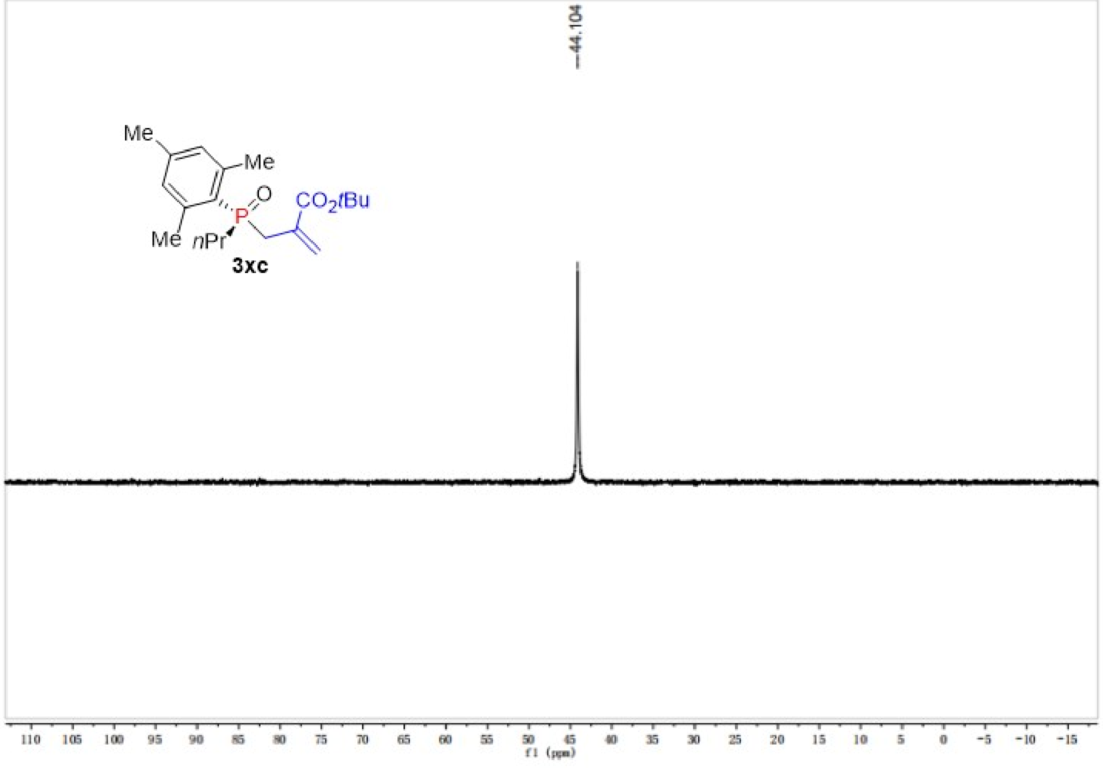

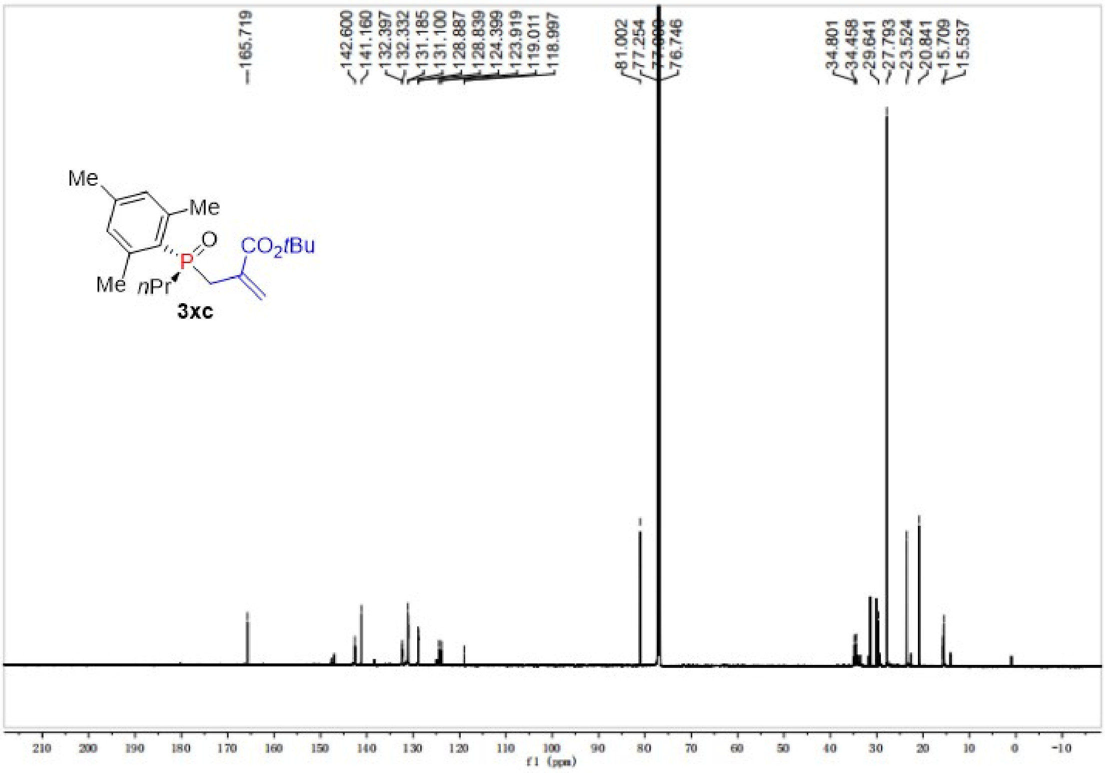
Me
Me
O
CO2tBu
P
Me nPr
3xc
165.719
142.600
141.160
132.397
132.332
131.185
131.100
128.887
128.839
124.399
123.919
119.011
118.997
81.002
77.254
76.746
34.801
34.458
29.641
27.793
23.524
20.841
15.709
15.537
f1 (ppm)

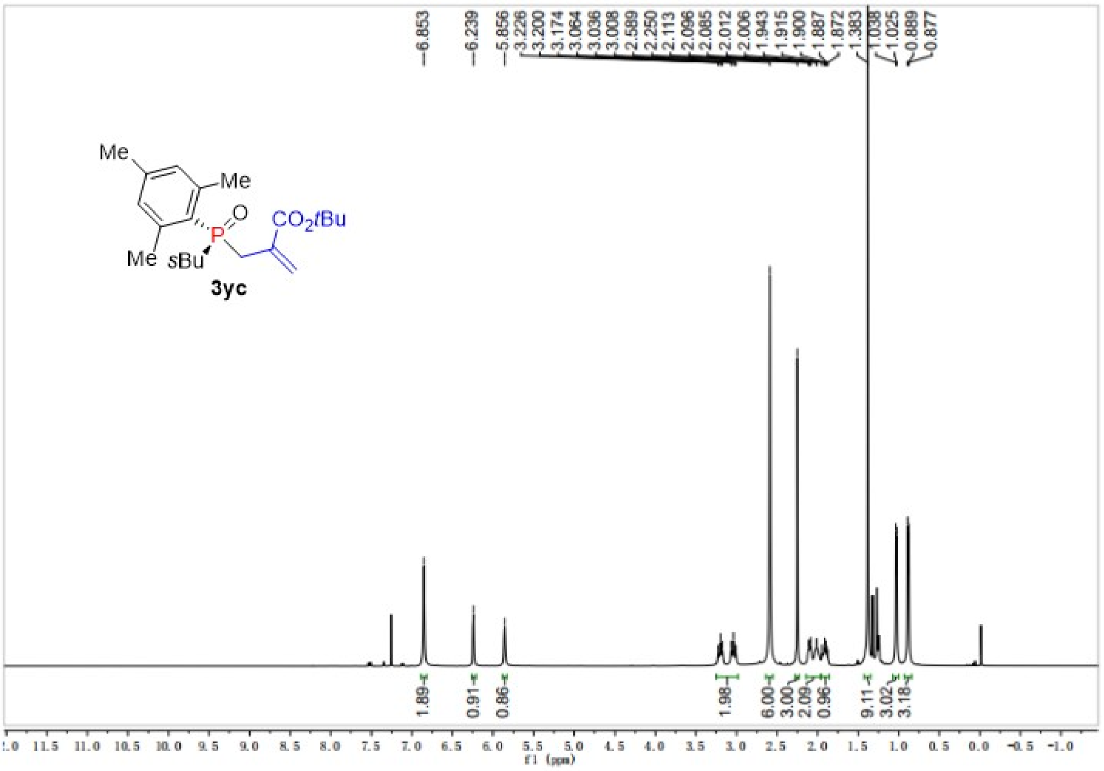
Me
Me
O
CO2tBu
P
Me sBu
3yc
6.853
6.239
5.856
3.226
3.200
3.174
3.064
3.036
3.008
2.589
2.250
2.113
2.096
2.085
2.012
2.006
1.943
1.915
1.900
1.887
1.872
1.383
1.038
1.025
0.889
0.877
1.89
0.91
0.86
1.98
6.00
3.00
2.09
0.96
9.11
3.02
3.18
f1 (ppm)

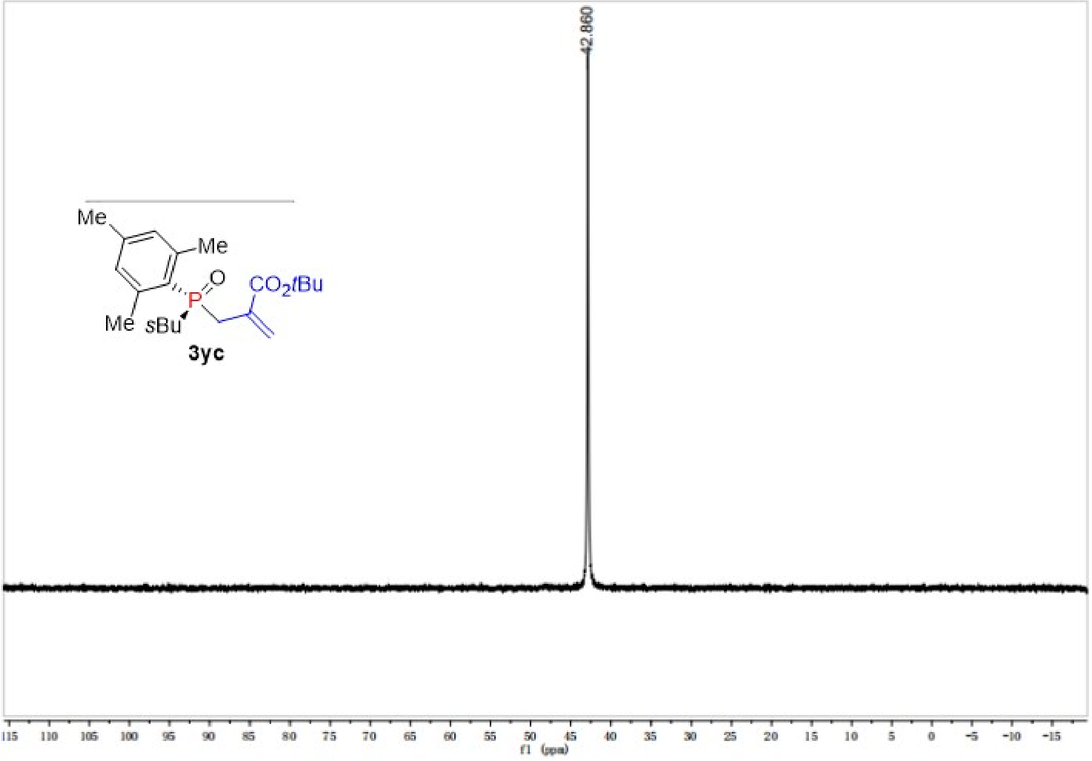

42.860
Me
Me
O
CO_2tBu
P
Me
sBu
3yc
f1 (ppm)

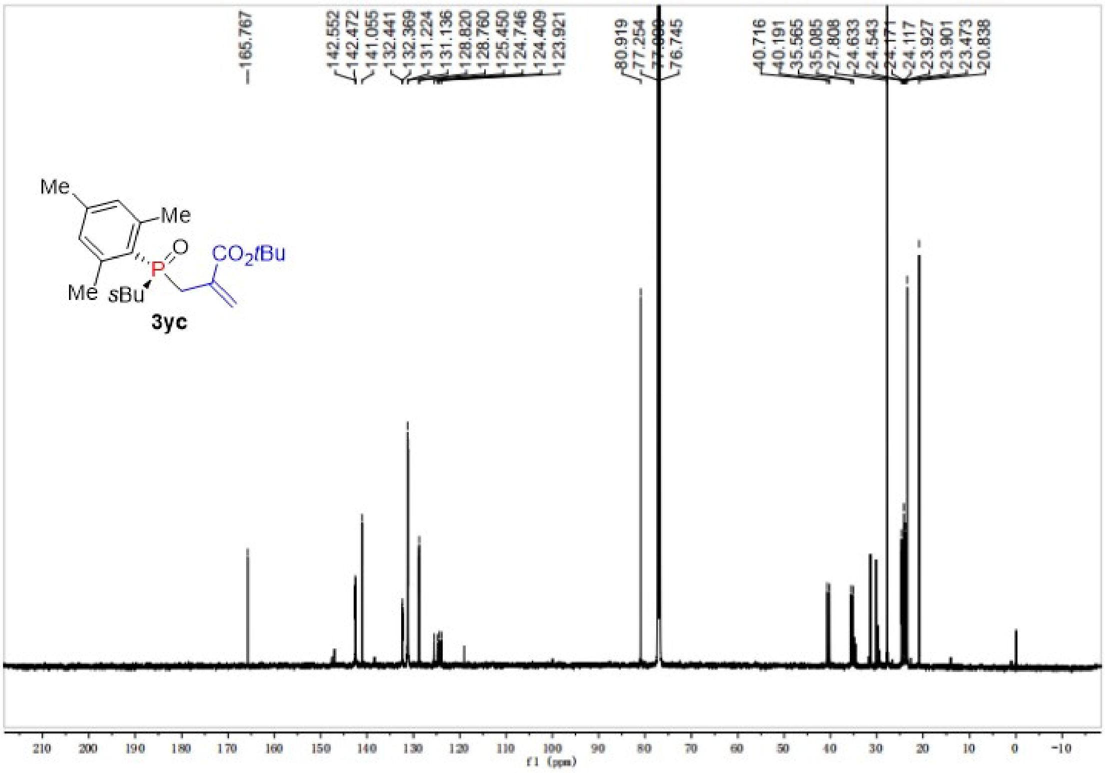

165.767
142.552
142.472
141.055
132.441
132.369
131.224
131.136
128.820
128.760
125.450
124.746
124.409
123.921
80.919
77.254
76.745
40.716
40.191
35.565
35.085
27.808
24.633
24.543
24.171
24.117
23.927
23.901
23.473
20.838
Me
Me
O
CO_2tBu
P
Me
sBu
3yc
f1 (ppm)

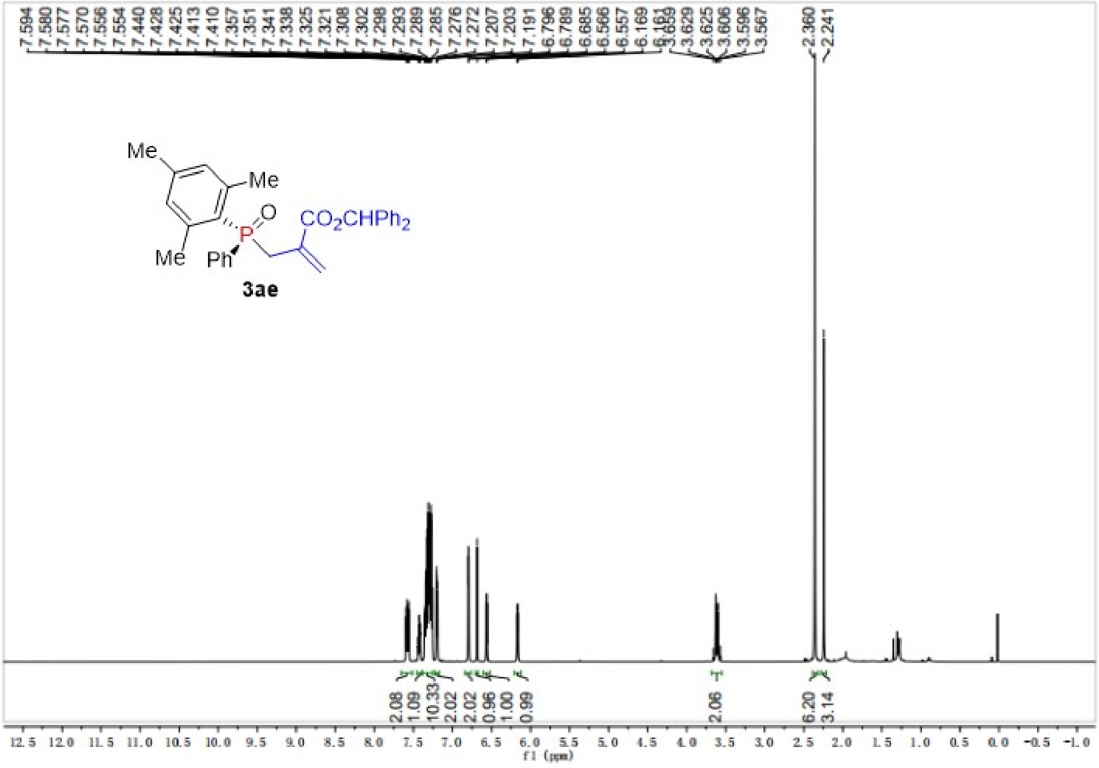

3ae

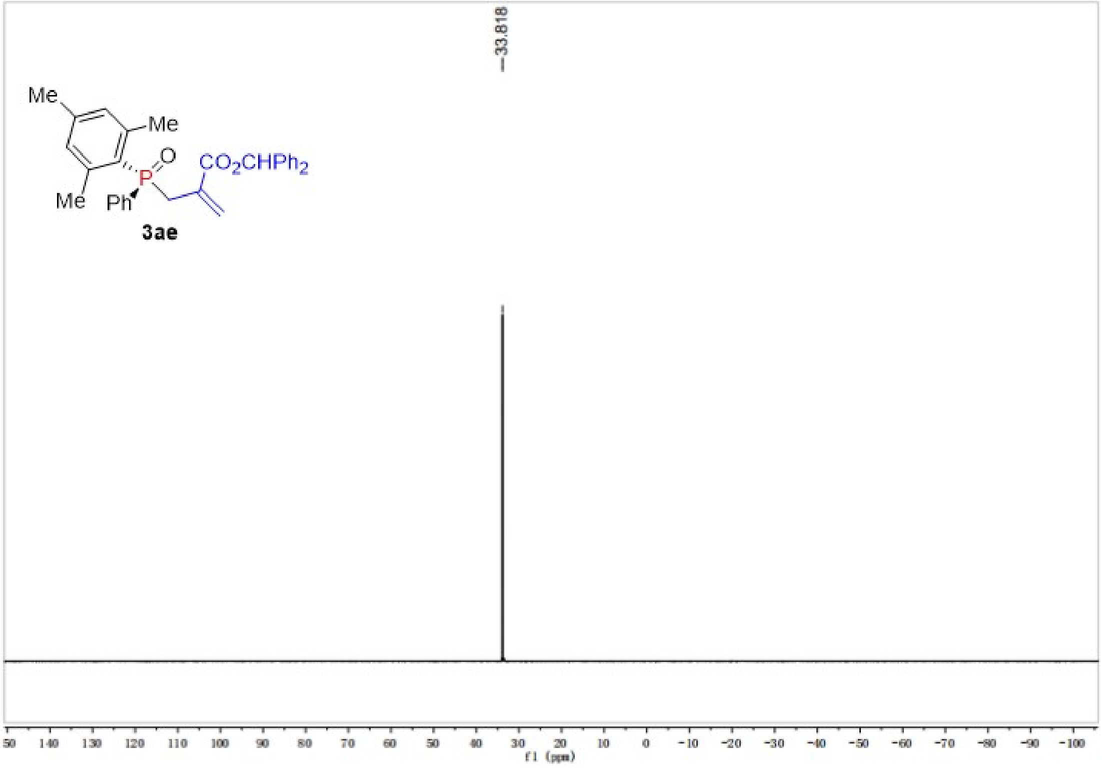

3ae
-33.818

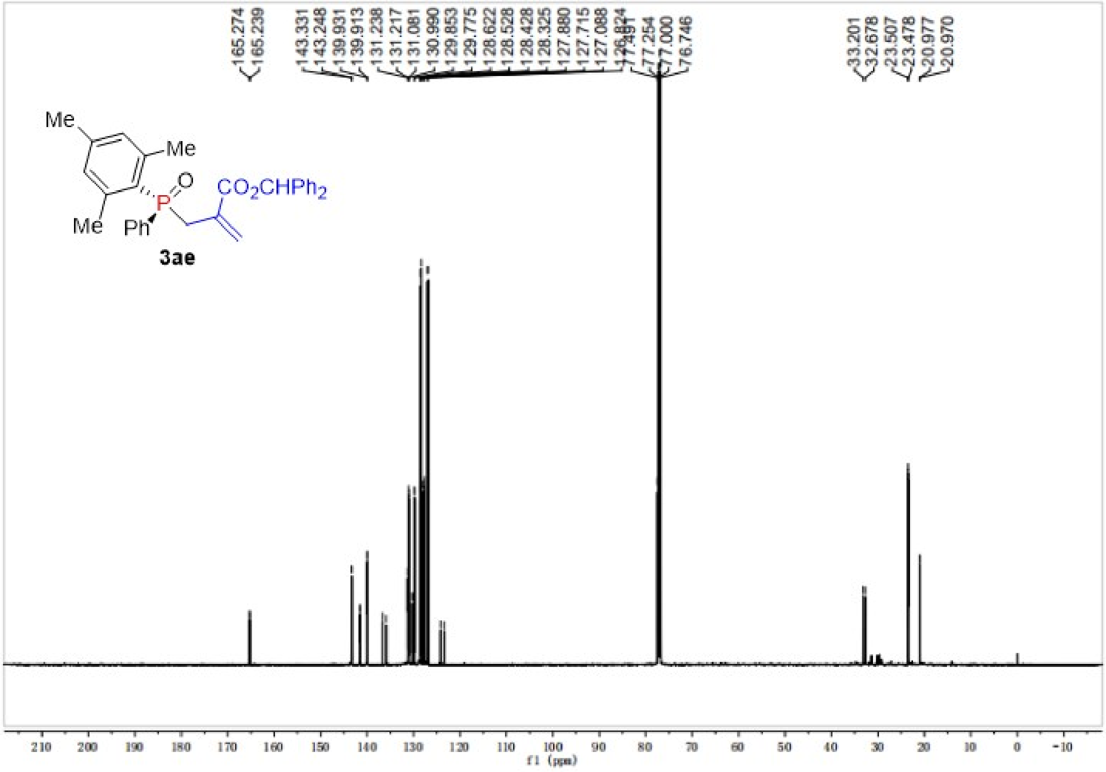
165.274
165.239
143.331
143.248
139.931
139.913
131.238
131.217
131.081
130.990
129.853
129.775
128.622
128.528
128.428
128.325
127.880
127.715
127.088
77.254
77.000
76.746
33.201
32.678
23.507
23.478
20.977
20.970
Me
Me
Me
O
P
Ph
CO2CHPh2
3ae
f1 (ppm)

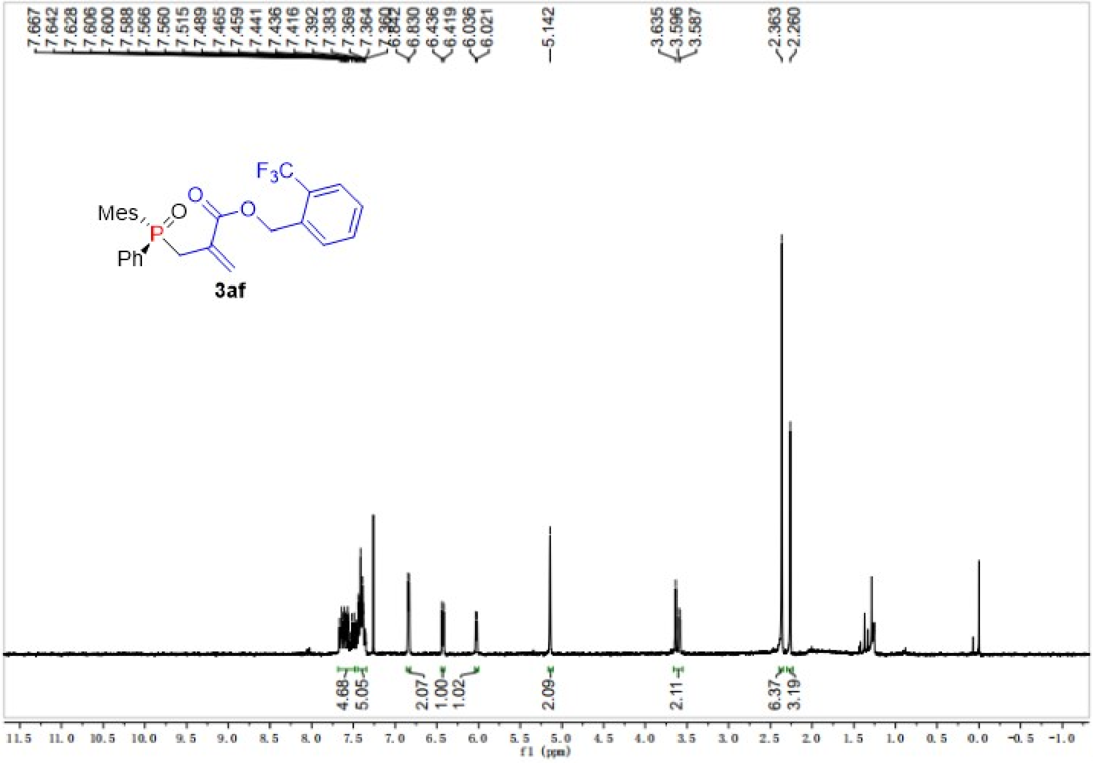
7.667
7.642
7.628
7.606
7.600
7.588
7.566
7.560
7.515
7.489
7.465
7.459
7.441
7.436
7.416
7.392
7.383
7.369
7.364
6.830
6.436
6.419
6.036
6.021
5.142
3.635
3.596
3.587
2.363
2.260
F3C
Mes
O
P
Ph
O
O
3af
4.68
5.05
2.07
1.00
1.02
2.09
2.11
6.37
3.19
f1 (ppm)

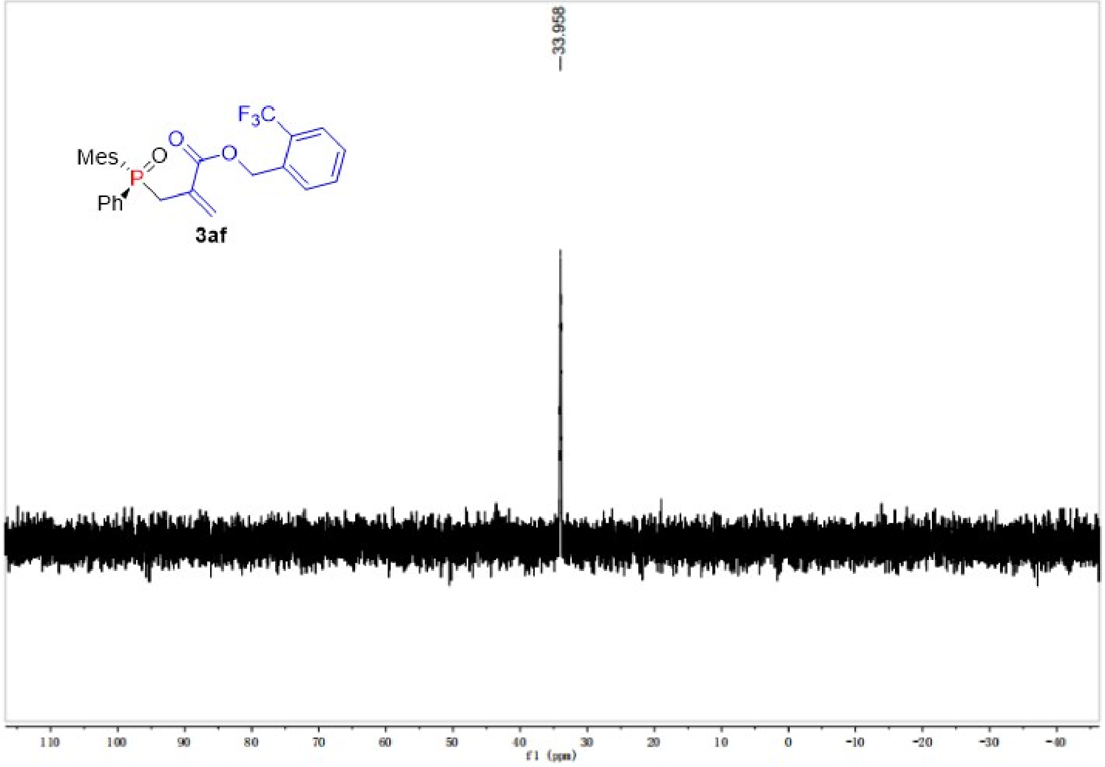

33.958
Mes
P
O
Ph
O
O
F3C
3af
110 100 90 80 70 60 50 40 30 20 10 0 -10 -20 -30 -40
f1 (ppm)

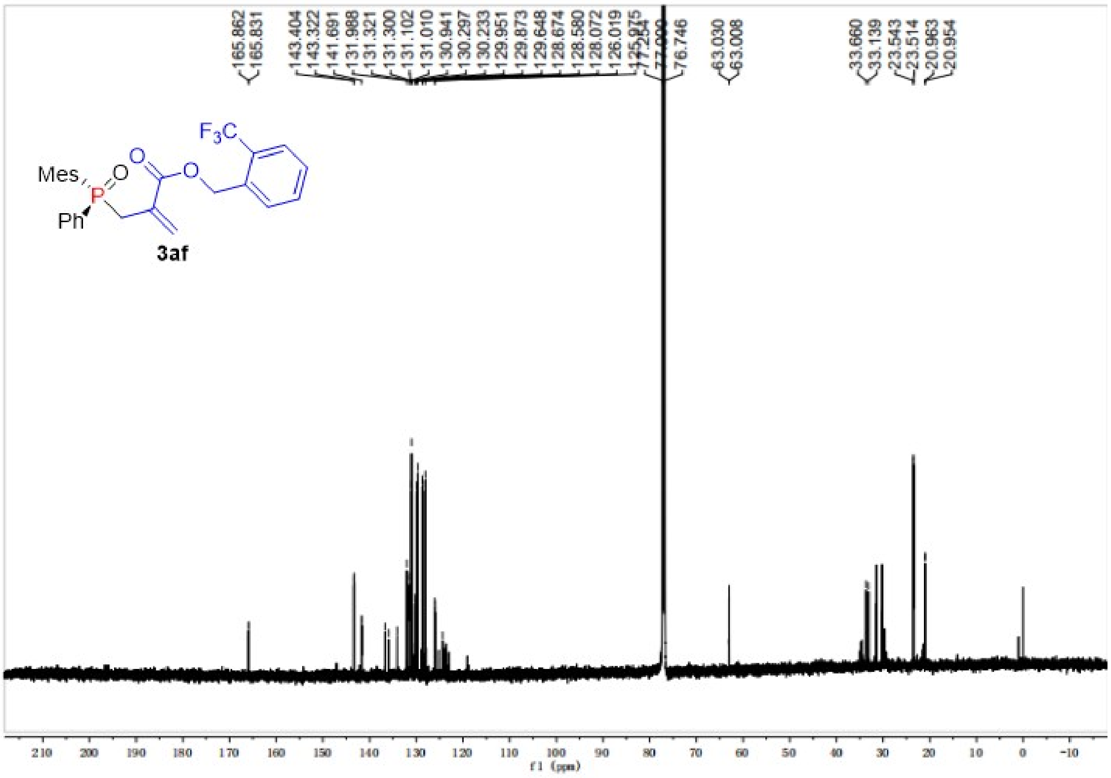

165.862
165.831
143.404
143.322
141.691
131.988
131.321
131.300
131.102
131.010
130.941
130.297
130.233
129.951
129.873
129.648
128.674
128.580
128.072
126.019
125.975
77.254
77.000
76.746
63.030
63.008
33.660
33.139
23.543
23.514
20.963
20.954
Mes
P
O
Ph
O
O
F3C
3af
210 200 190 180 170 160 150 140 130 120 110 100 90 80 70 60 50 40 30 20 10 0 -10
f1 (ppm)

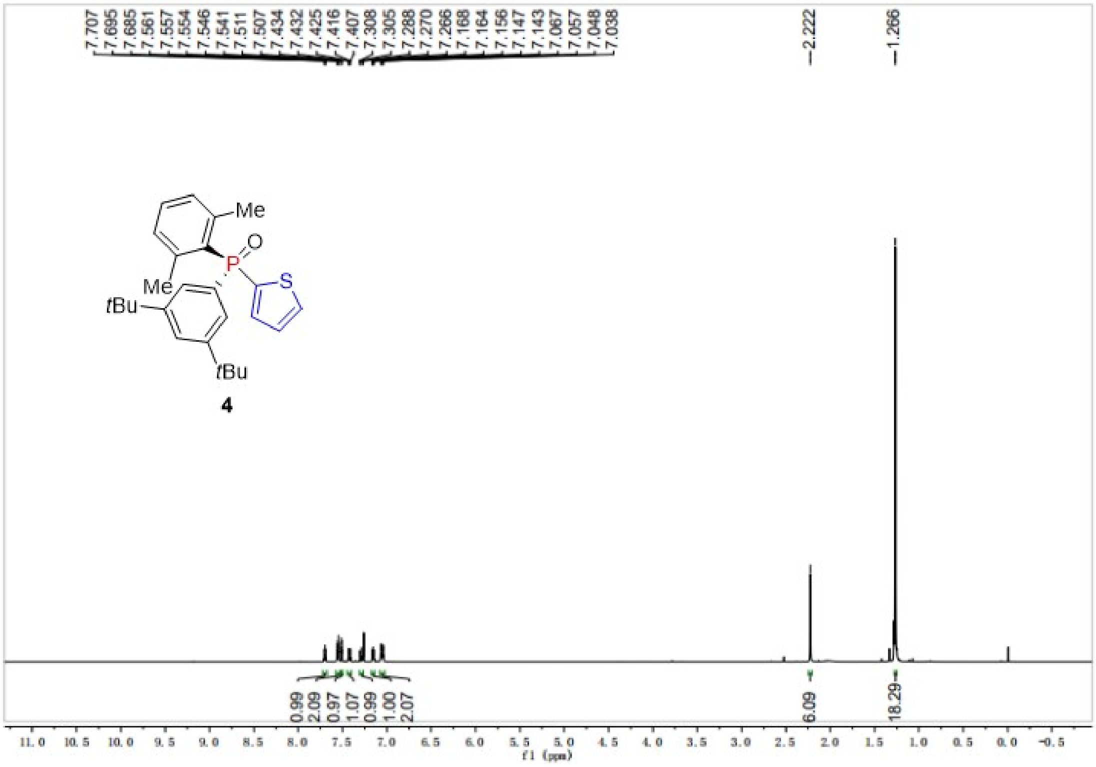
7.707
7.695
7.685
7.561
7.557
7.554
7.546
7.541
7.511
7.507
7.434
7.432
7.425
7.416
7.407
7.308
7.305
7.288
7.270
7.266
7.168
7.164
7.156
7.147
7.143
7.067
7.057
7.048
7.038
2.222
1.266
Me
P
O
S
Me
tBu
tBu
4
0.99
2.09
0.97
1.07
0.99
1.00
2.07
6.09
18.29
f1 (ppm)

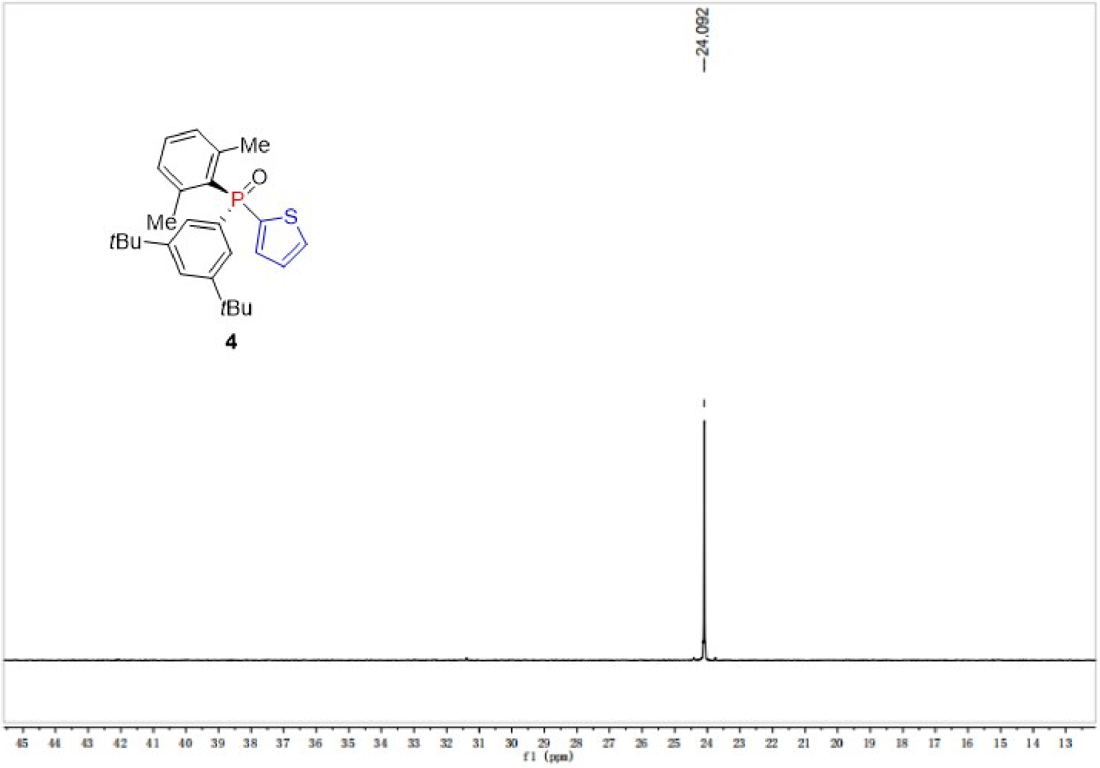
24.092
Me
P
O
S
Me
tBu
tBu
4
f1 (ppm)

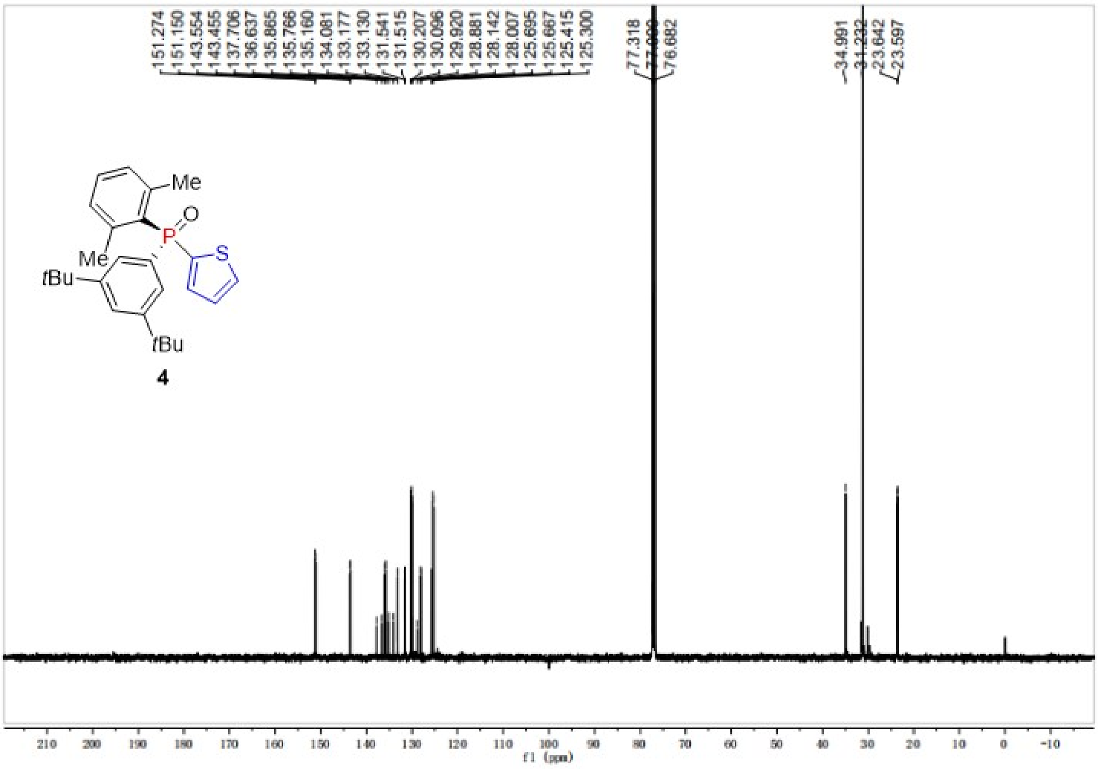

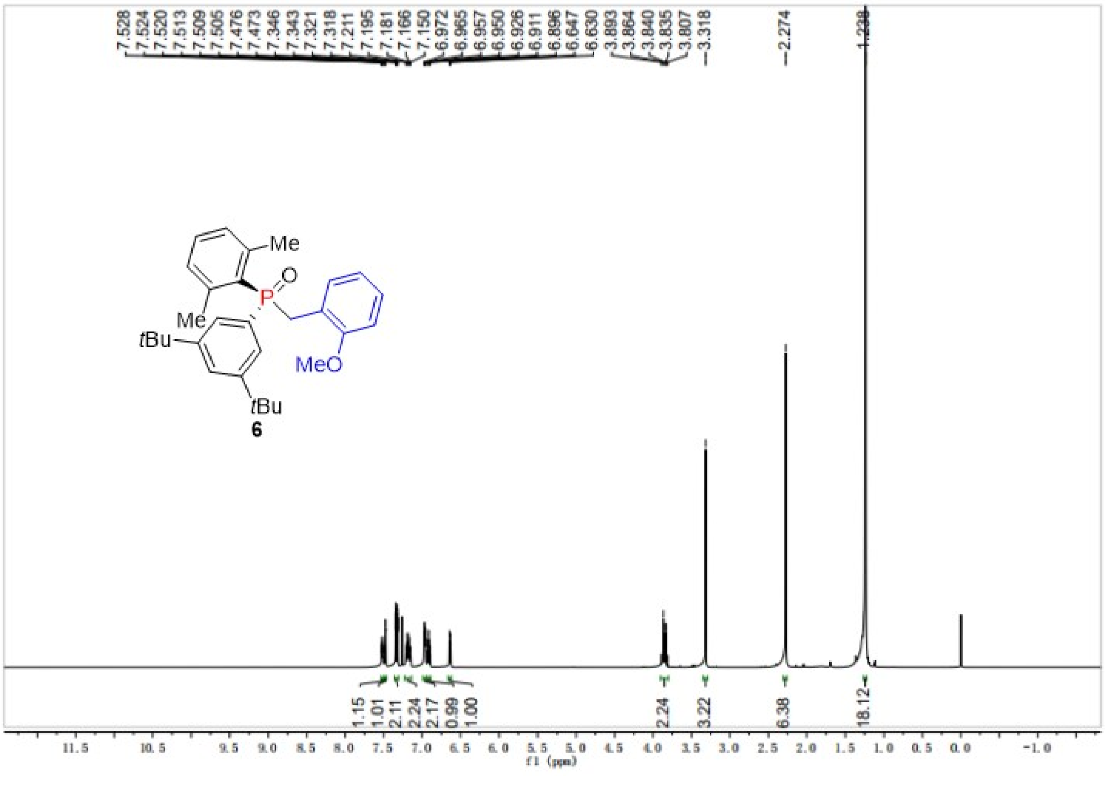

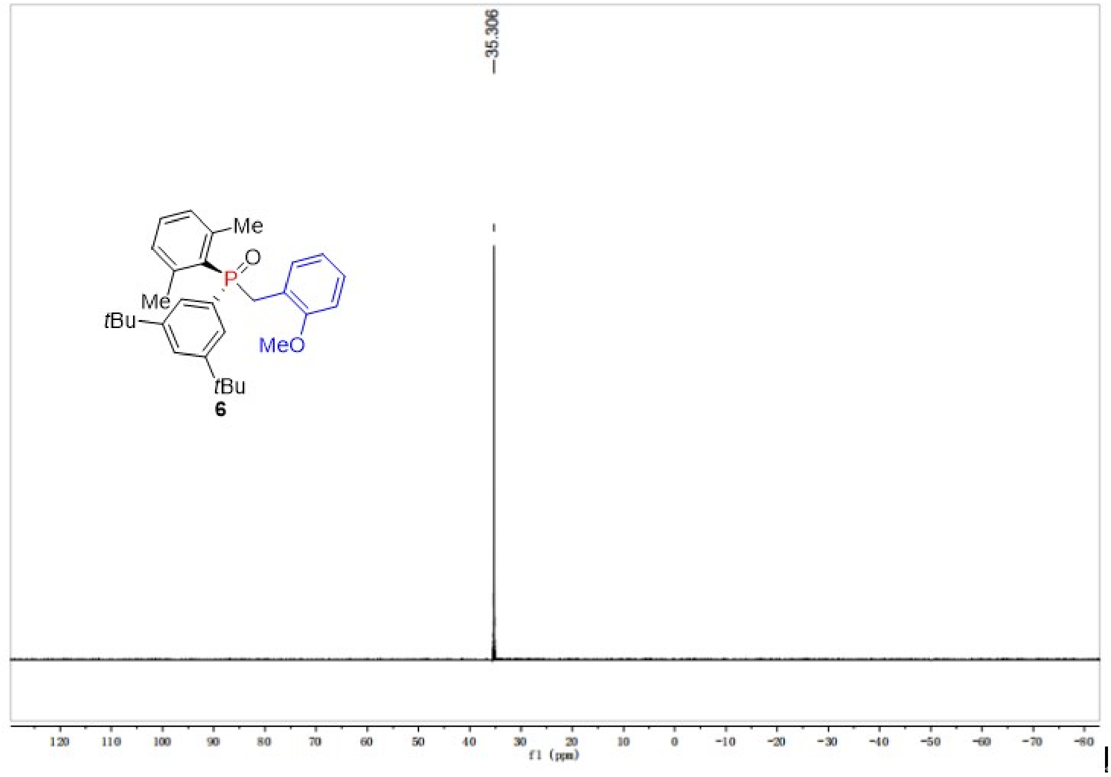
—35.306
Me
O
P
Me
tBu
MeO
tBu
6
f1 (ppm)

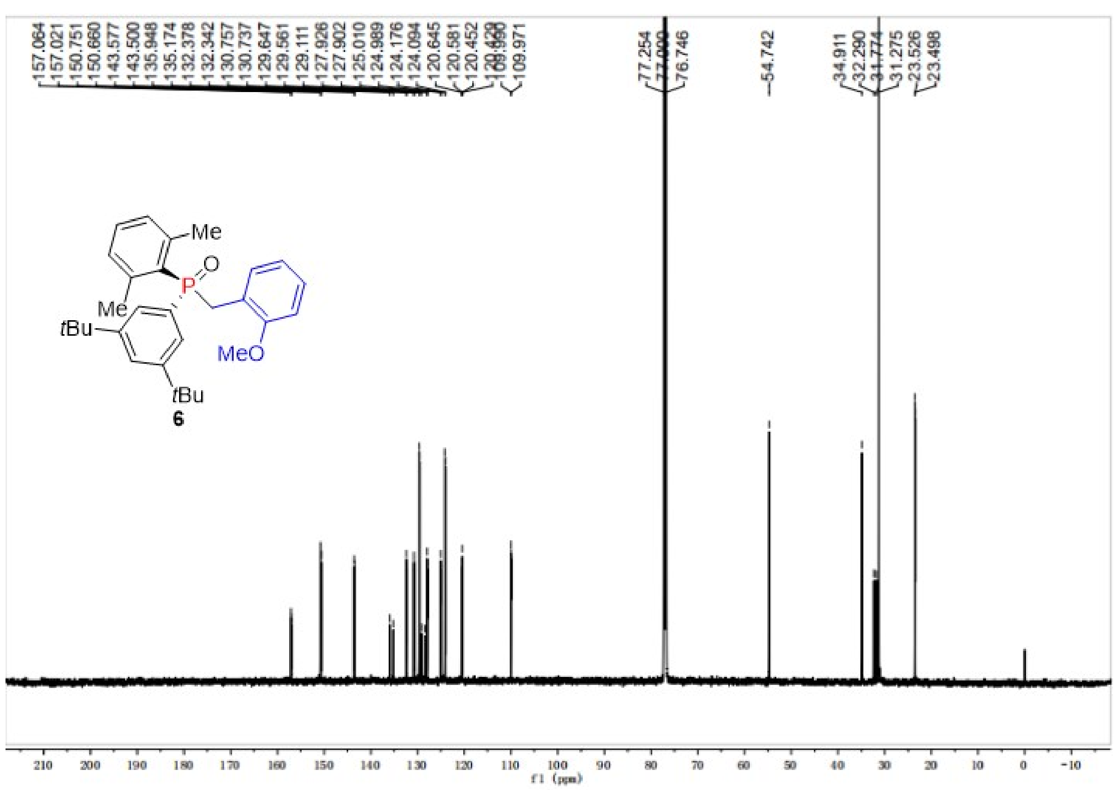
157.064
157.021
150.751
150.660
143.577
143.500
135.948
135.174
132.378
132.342
130.757
130.737
129.647
129.561
129.111
127.926
127.902
125.010
124.989
124.176
124.094
120.645
120.581
120.452
109.971
77.254
76.746
—54.742
34.911
32.290
31.774
31.275
23.526
23.498
Me
O
P
Me
tBu
MeO
tBu
6
f1 (ppm)

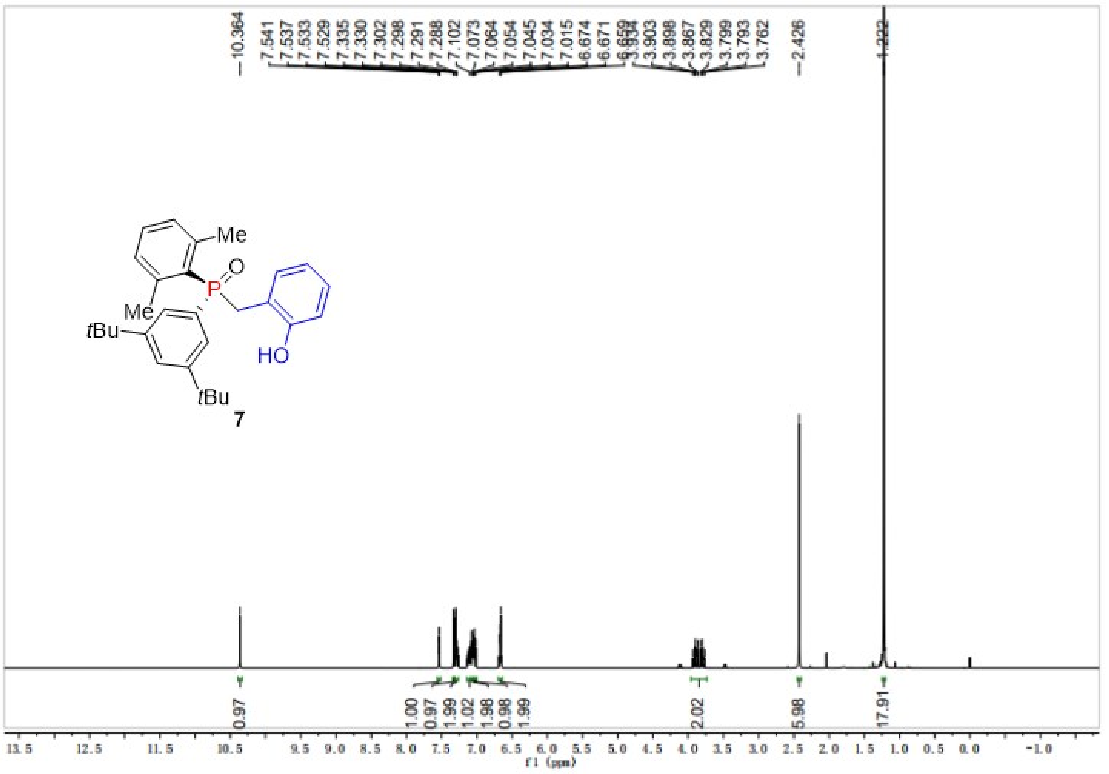
10.364
7.541
7.537
7.533
7.529
7.335
7.330
7.302
7.298
7.291
7.288
7.102
7.073
7.064
7.054
7.045
7.034
7.015
6.674
6.671
3.903
3.898
3.867
3.829
3.799
3.793
3.762
2.426
1.222
Me
O
P
Me
tBu
HO
tBu
7
0.97
1.00
0.97
1.99
1.02
1.98
0.98
1.99
2.02
5.98
17.91
f1 (ppm)

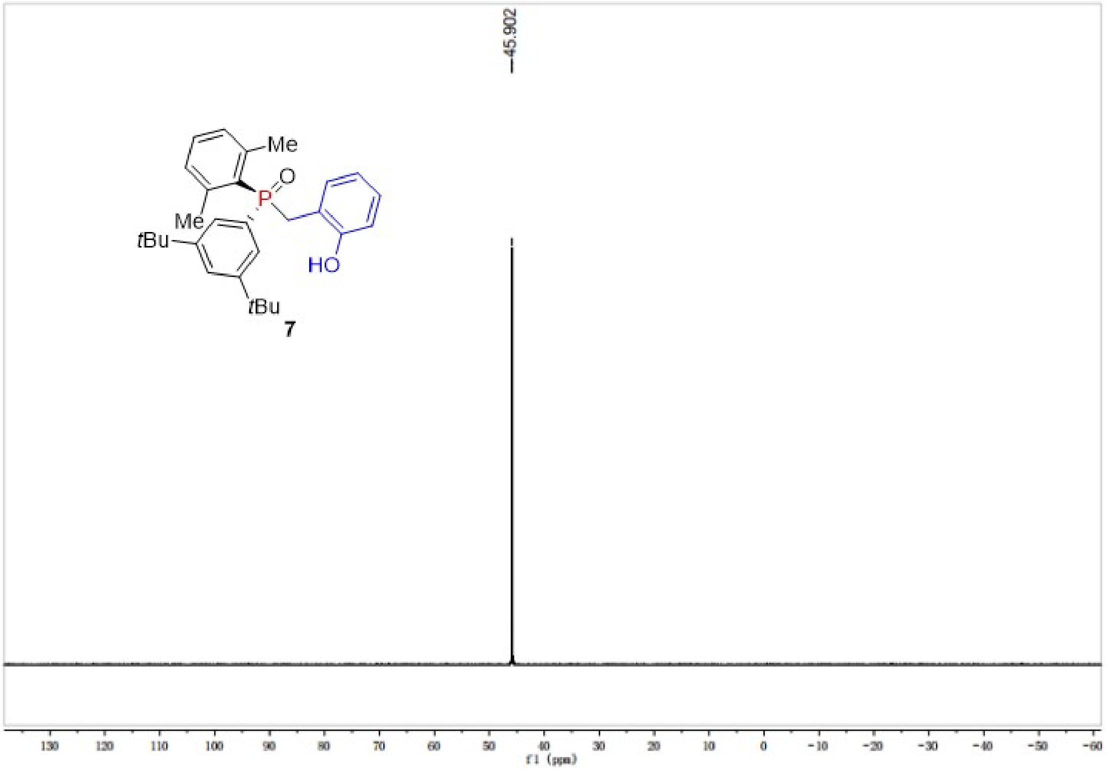
45.902
Me
O
P
Me
tBu
HO
tBu
7
f1 (ppm)

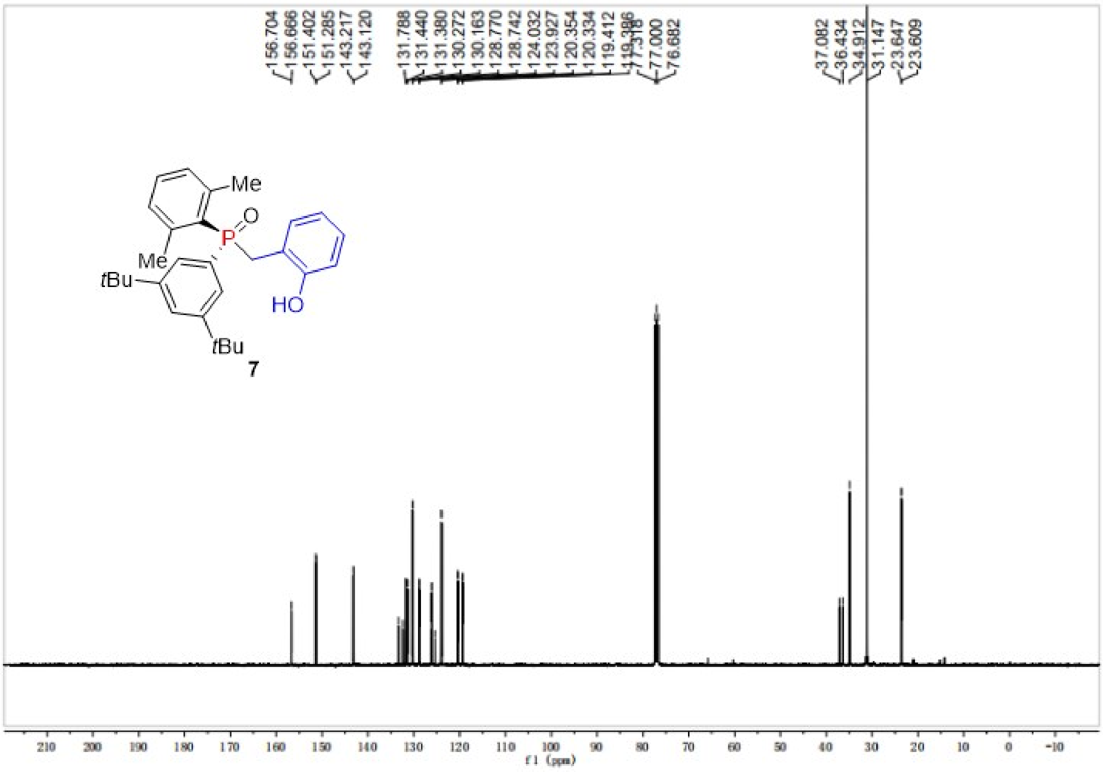
156.704
156.666
151.402
151.285
143.217
143.120
131.788
131.440
131.380
130.272
130.163
128.770
128.742
124.032
123.927
120.354
120.334
119.412
119.386
77.318
77.000
76.682
37.082
36.434
34.912
31.147
23.647
23.609
Me
O
P
Me
tBu
HO
tBu
7
f1 (ppm)

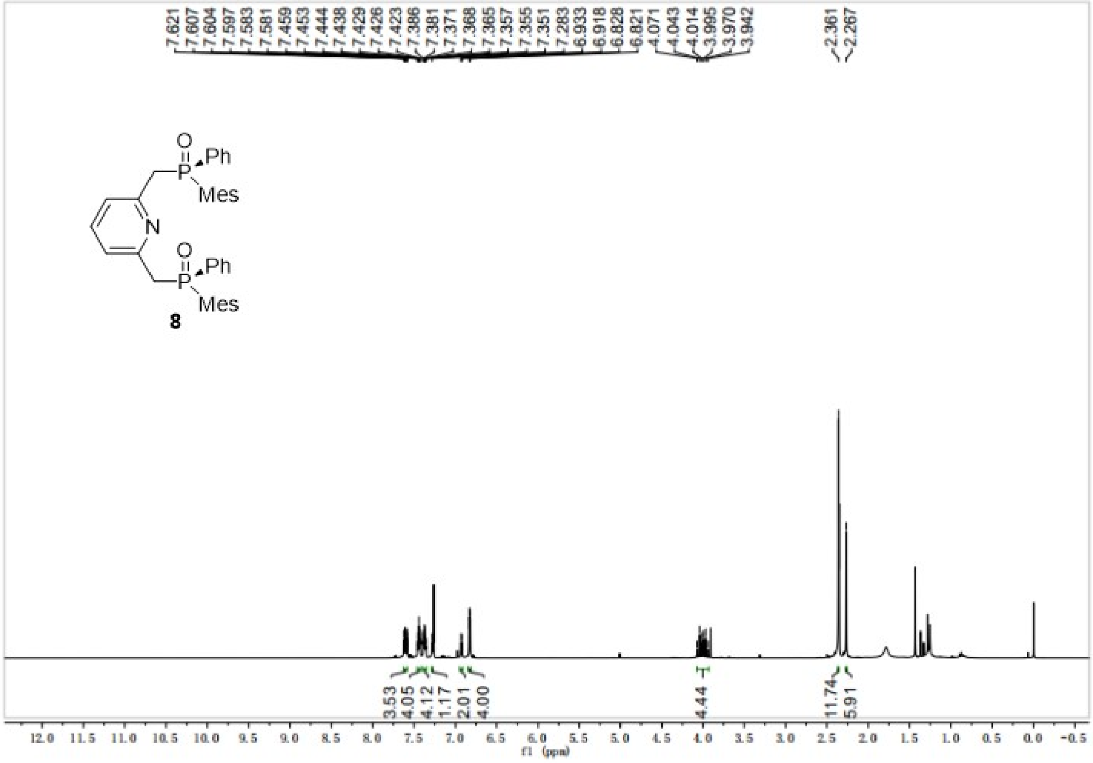
7.621
7.607
7.604
7.597
7.583
7.581
7.459
7.453
7.444
7.438
7.429
7.426
7.423
7.386
7.381
7.371
7.368
7.365
7.357
7.355
7.351
7.283
6.933
6.918
6.828
6.821
4.071
4.043
4.014
3.995
3.970
3.942
2.361
2.267
O
P
Ph
Mes
N
O
P
Ph
Mes
8
3.53
4.05
4.12
1.17
2.01
4.00
4.44
11.74
5.91
f1 (ppm)

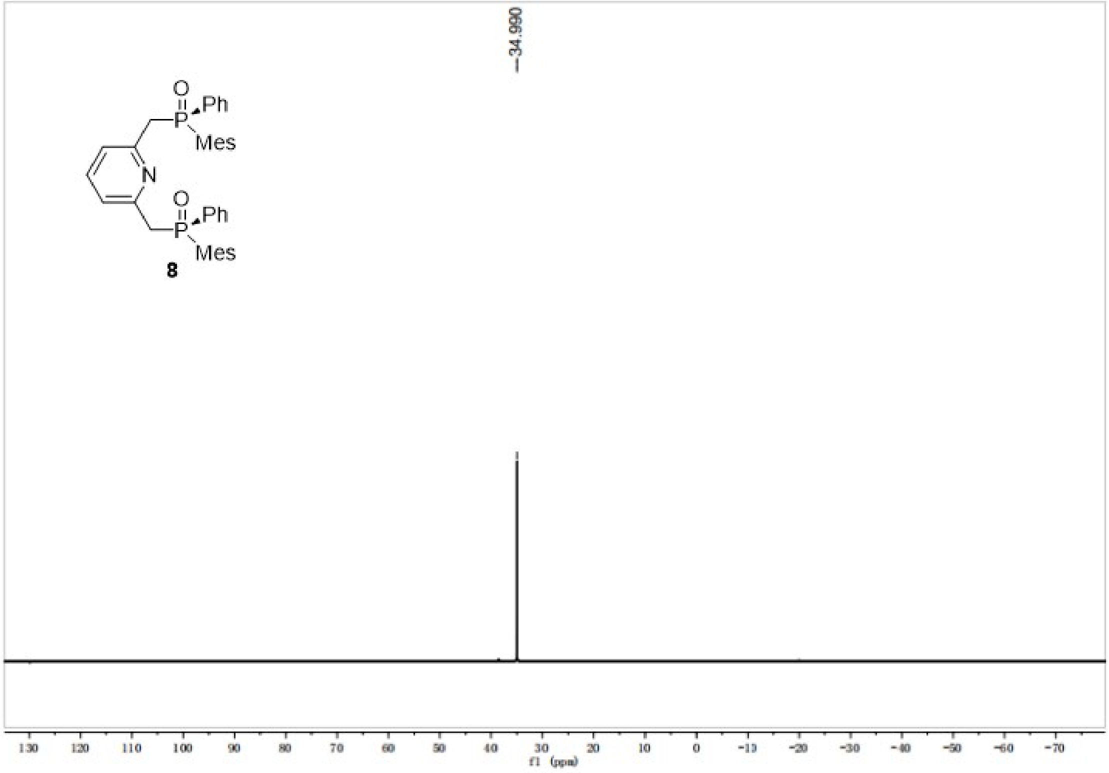

34.990
Ph
Mes
N
8
f1 (ppm)

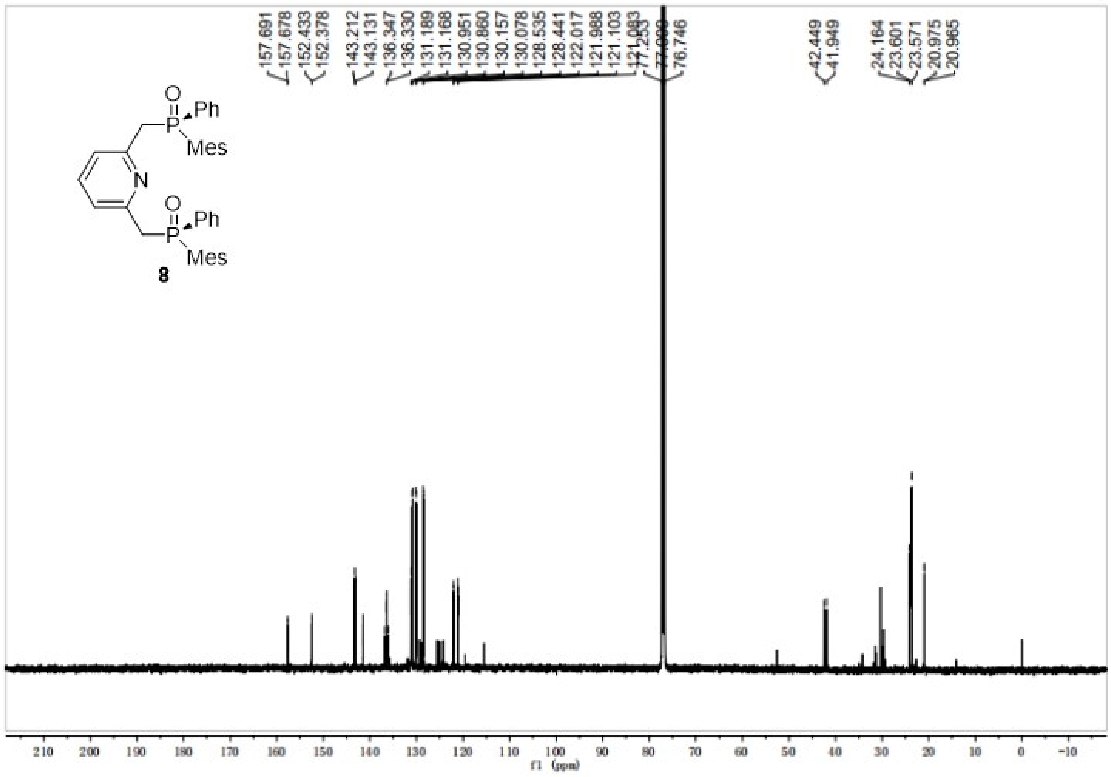

157.691
157.678
152.433
152.378
143.212
143.131
136.347
136.330
131.189
131.168
130.951
130.860
130.157
130.078
128.535
128.441
122.017
121.988
121.103
121.083
77.253
76.746
42.449
41.949
24.164
23.601
23.571
20.975
20.965
Ph
Mes
N
8
f1 (ppm)

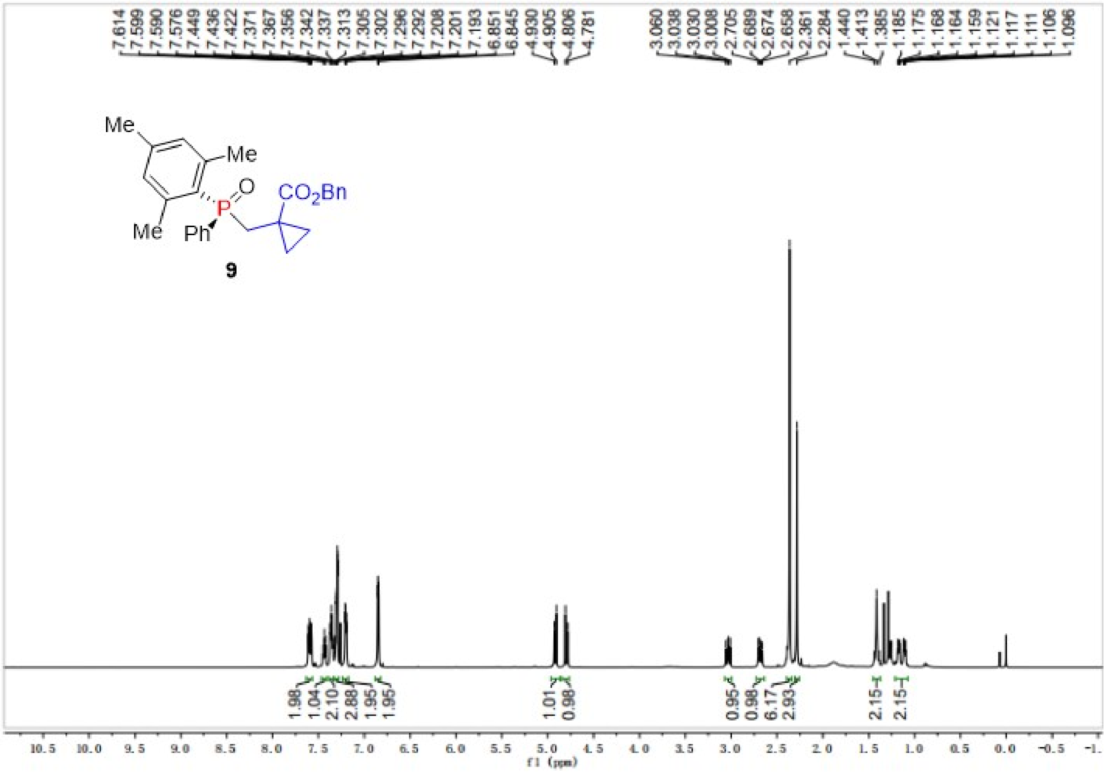
7.614
7.599
7.590
7.576
7.449
7.436
7.422
7.371
7.367
7.356
7.342
7.337
7.313
7.305
7.302
7.296
7.292
7.208
7.201
7.193
6.851
6.845
4.930
4.905
4.806
4.781
3.060
3.038
3.030
3.008
2.705
2.689
2.674
2.658
2.361
2.284
1.440
1.413
1.385
1.185
1.175
1.168
1.164
1.159
1.121
1.117
1.111
1.106
1.096
Me
Me
O
CO2Bn
P
Me
Ph
9
1.98
1.04
2.10
2.88
1.95
1.95
1.01
0.98
0.95
0.98
6.17
2.93
2.15
2.15
f1 (ppm)

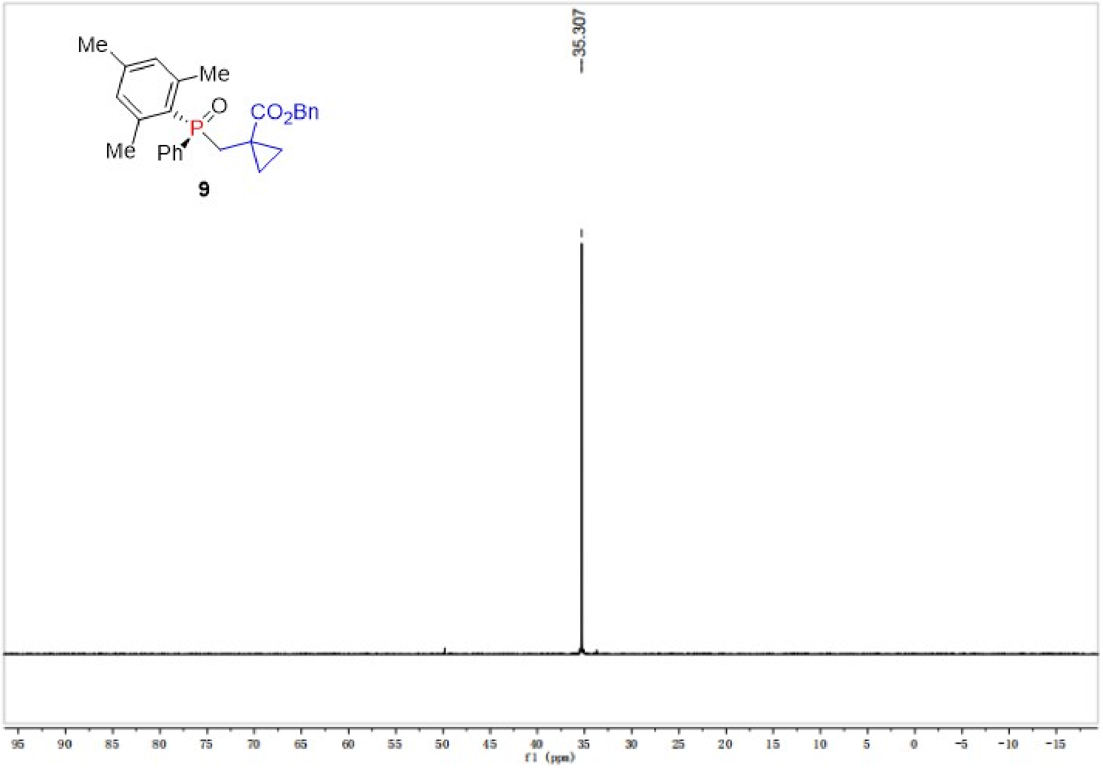
—35.307
Me
Me
O
CO2Bn
P
Me
Ph
9
f1 (ppm)

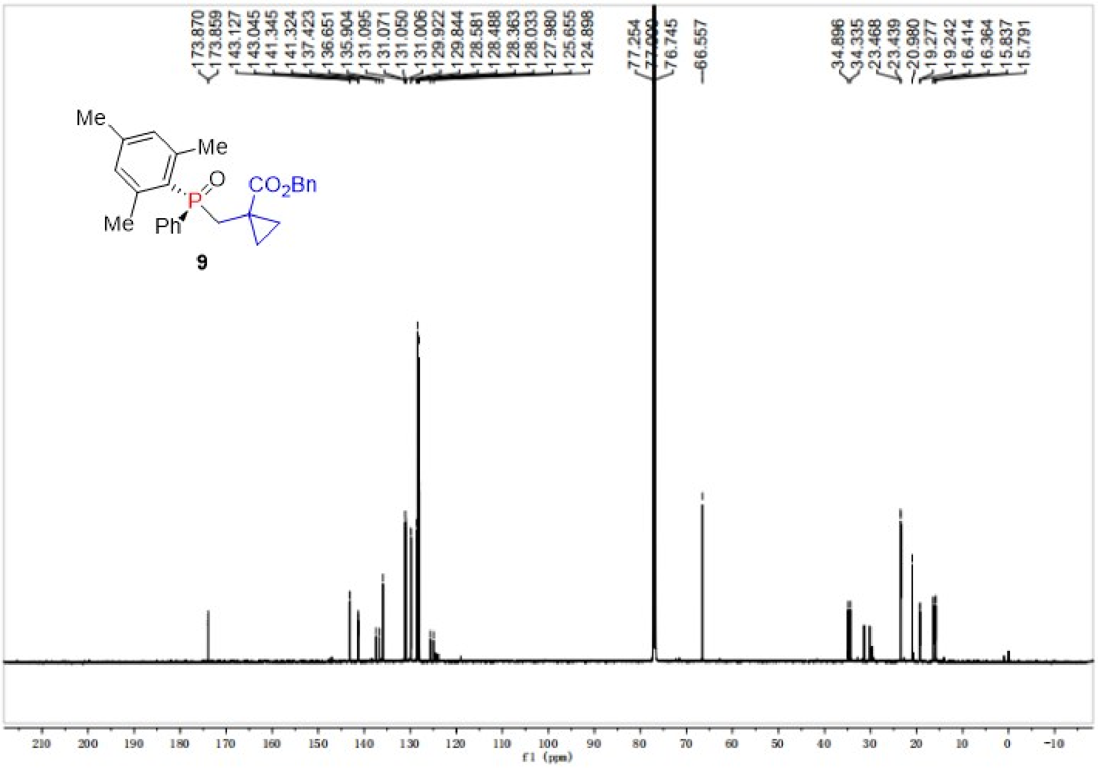

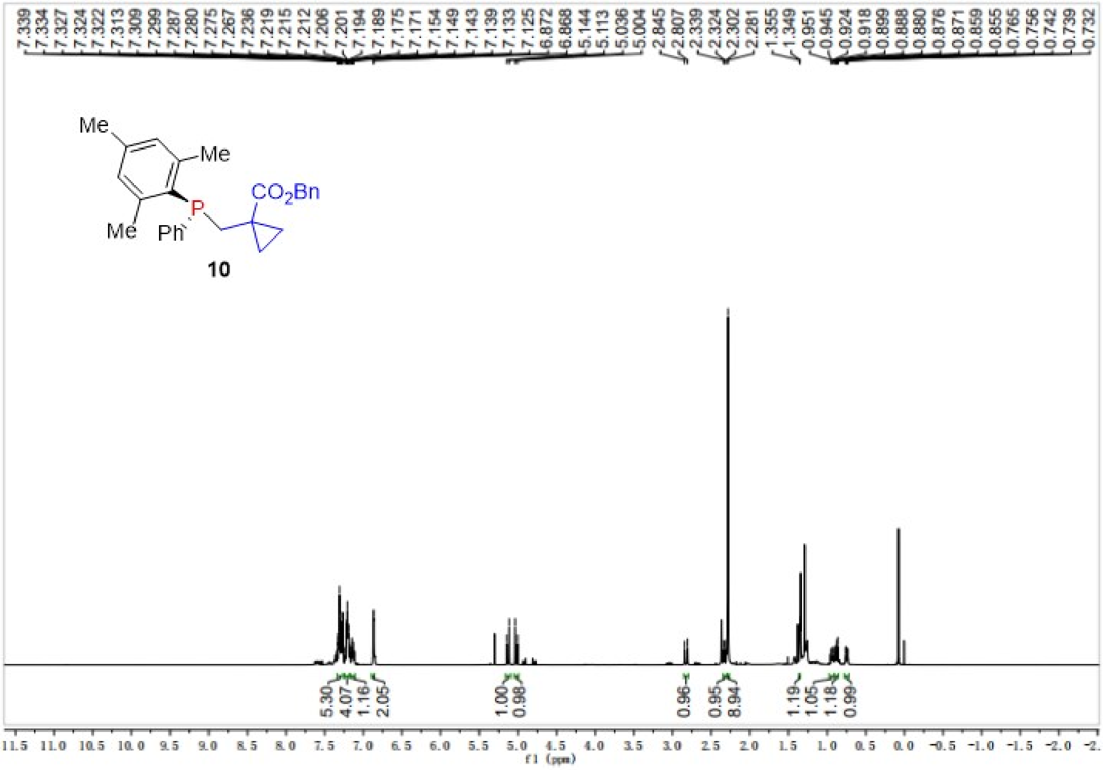

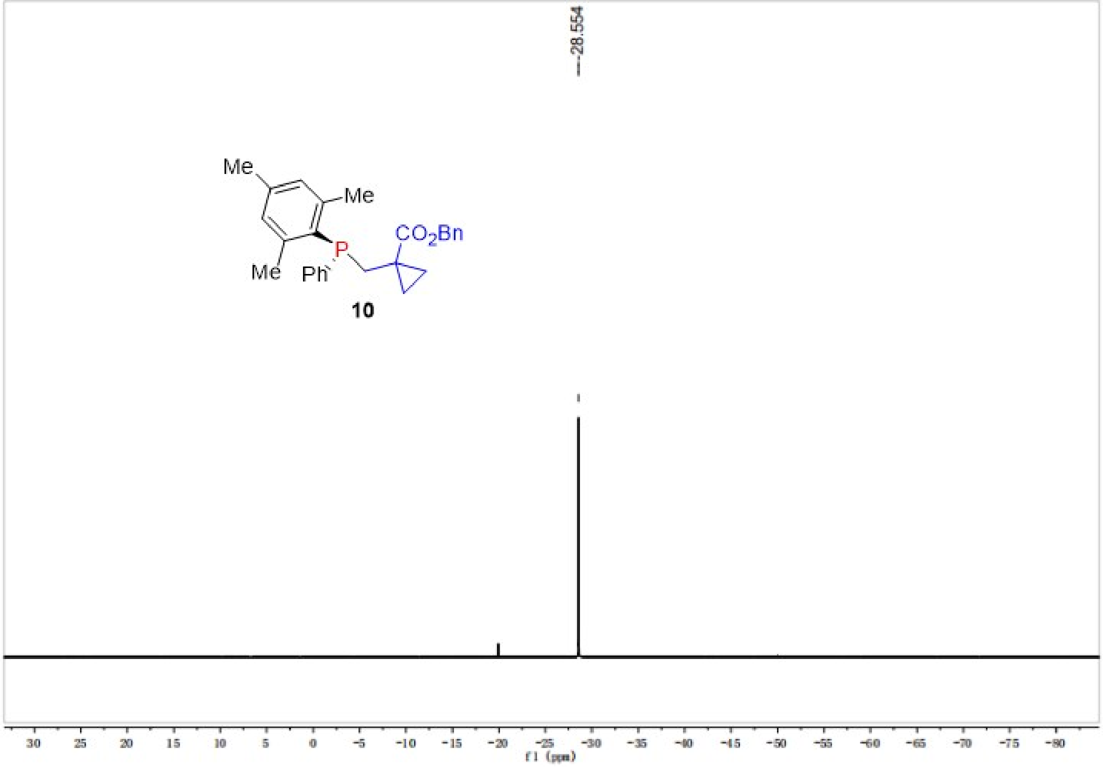

-28.554
Me
Me
CO2Bn
P
Me
Ph
10
f1 (ppm)

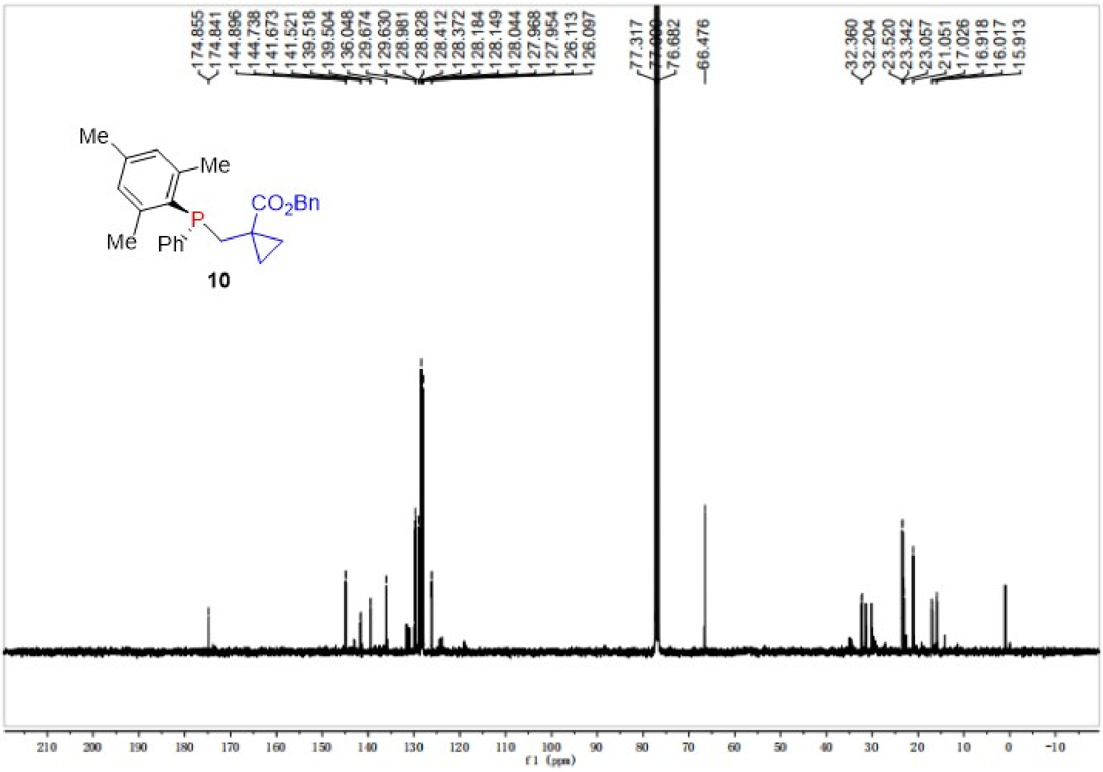

174.855
174.841
144.896
144.738
141.673
141.521
139.518
139.504
136.048
129.674
129.630
128.981
128.828
128.412
128.372
128.184
128.149
128.044
127.968
127.954
126.113
126.097
77.317
76.682
66.476
32.360
32.204
23.520
23.342
23.057
21.051
17.026
16.918
16.017
15.913
Me
Me
CO2Bn
P
Me
Ph
10
f1 (ppm)

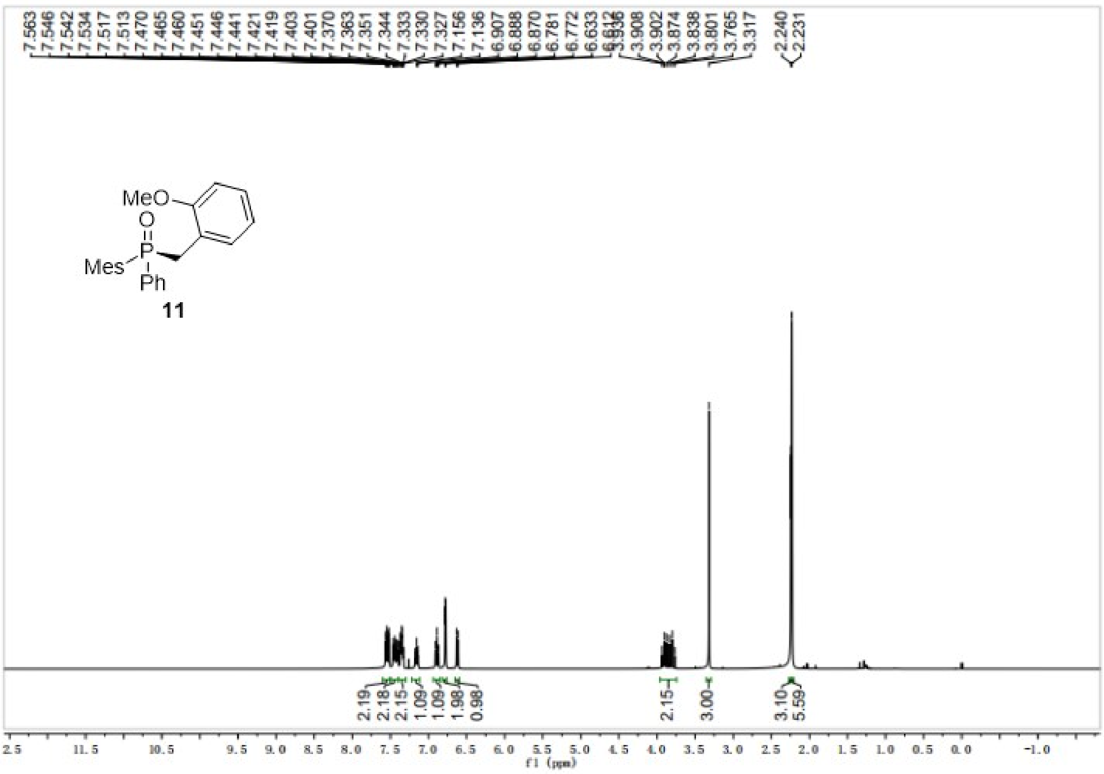

MeO
O
P
Mes
Ph
11
7.563
7.546
7.542
7.534
7.517
7.513
7.470
7.465
7.460
7.451
7.446
7.441
7.421
7.419
7.403
7.401
7.370
7.363
7.351
7.344
7.333
7.330
7.327
7.156
7.136
6.907
6.888
6.870
6.781
6.772
6.633
6.612
3.936
3.908
3.902
3.874
3.838
3.801
3.765
3.317
2.240
2.231
2.19
2.18
2.15
1.09
1.09
1.98
0.98
2.15
3.00
3.10
5.59
f1 (ppm)

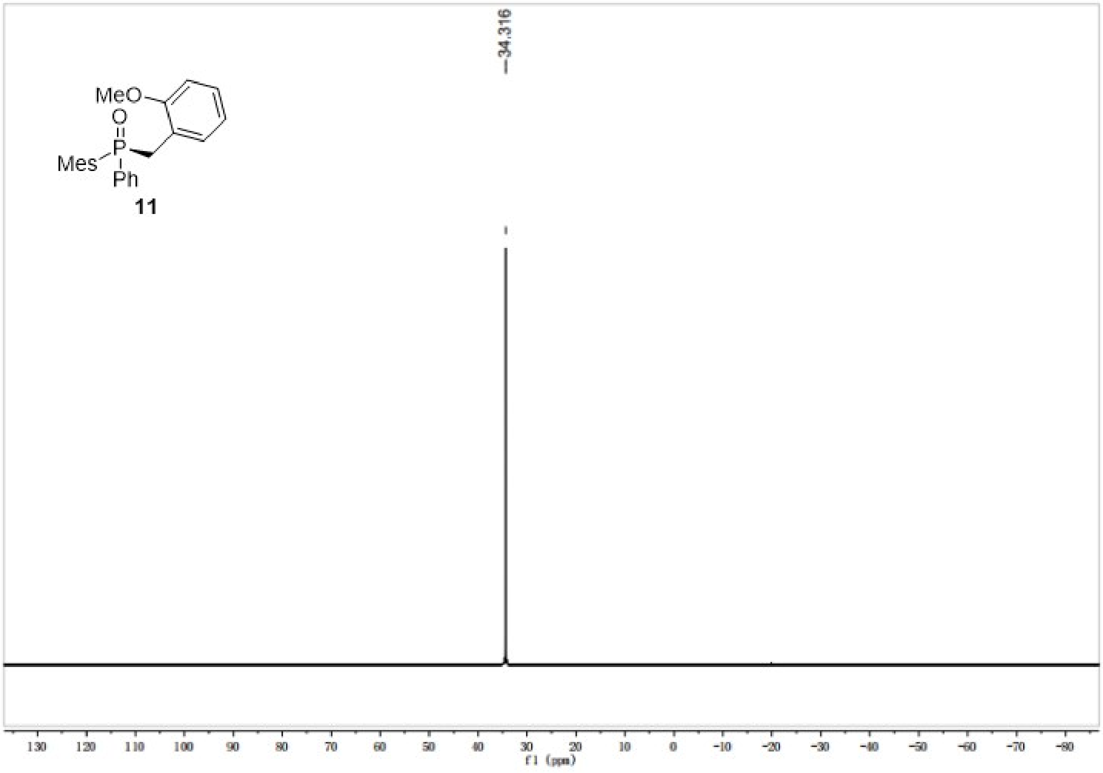

34.316
MeO
O
P
Mes
Ph
11
f1 (ppm)

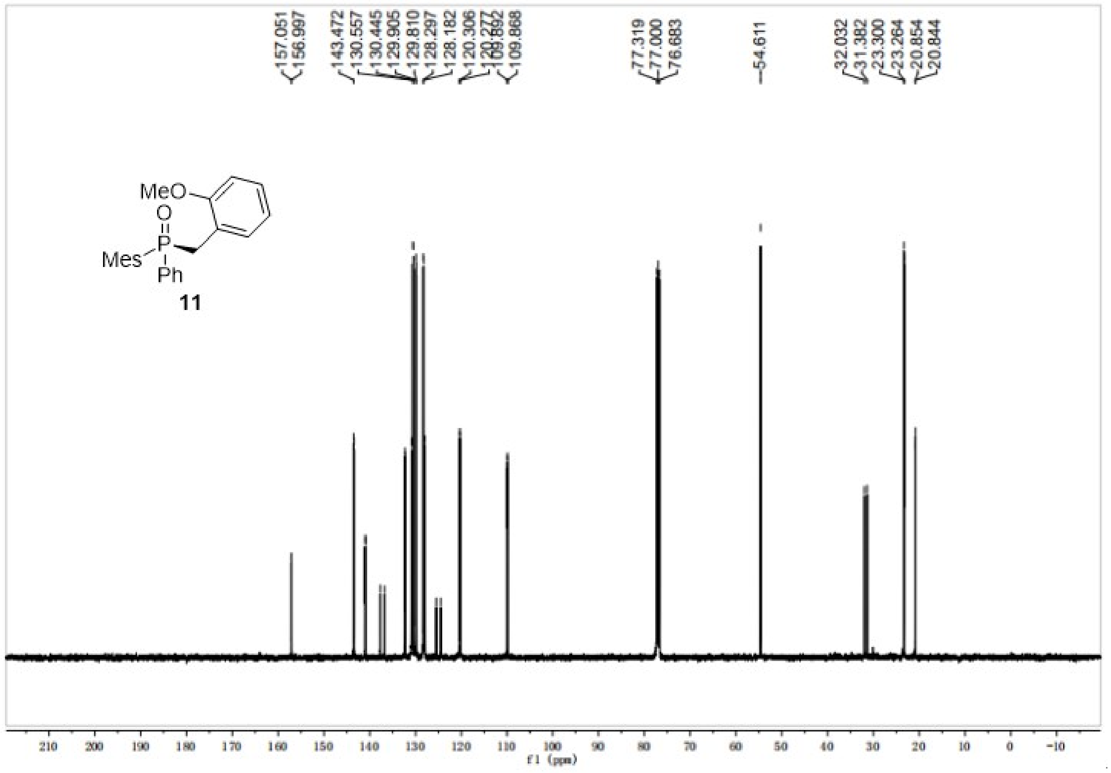
157.051
156.997
143.472
130.557
130.445
129.905
129.810
128.297
128.182
120.306
120.277
109.892
109.868
77.319
77.000
76.683
54.611
32.032
31.382
23.300
23.264
20.854
20.844
MeO
O
P
Mes
Ph
11
f1 (ppm)
